# Large-Scale Profiling of Signaling Pathways Reveals a Distinct Demarcation between Normal and Extended Liver Resection

**DOI:** 10.3390/cells9051149

**Published:** 2020-05-07

**Authors:** Pieter Borger, Anton Buzdin, Maksim Sorokin, Ekaterina Kachaylo, Bostjan Humar, Rolf Graf, Pierre-Alien Clavien

**Affiliations:** 1 Swiss Hepato-Pancreatico-Biliary and Transplantation Center, Department of Surgery & Transplantation, University Hospital Zurich, Rämistrasse 100, CH-8091 Zürich, Switzerland; Ekaterina.kachaylo@usz.ch (E.K.); bostjan.humar@usz.ch (B.H.); rolf.graf@usz.ch (R.G.); pierre-alain.clavien@usz.ch (P.-A.C.); 2 Shemyakin-Ovchinnikov Institute of Bioorganic Chemistry, 117198 Moscow, Russia; buzdin@ponkc.com (A.B.); sorokin.maks@gmail.com (M.S.); 3 I.M. Sechenov First Moscow State Medical University, 119991 Moscow, Russia; 4 OmicsWay Corp., Walnut, CA 91798, USA

**Keywords:** normal liver resection, extended liver resection, liver failure, small-for-size syndrome, RNAseq, intracellular signaling pathways, Oncofinder

## Abstract

Despite numerous studies addressing normal liver regeneration, we still lack comprehensive understanding of the biological processes underlying failed liver regeneration. Therefore, we analyzed the activity of 271 intracellular signaling pathways (ISPs) by genome wide profiling of differentially expressed RNAs in murine liver tissue biopsies after normal hepatectomy (nHx; 68% of liver removed) and extended hepatectomy (eHx; 86% of liver removed). Comprehensive, genome-wide transcriptome profiling using RNAseq was performed in liver tissue obtained from mice (sham, nHx, and eHx) harvested 1, 8, 16, 32, and 48 h after operation (n = 3 per group) and the OncoFinder toolkit was used for an unsupervised, unbiased identification of intracellular signaling pathways (ISP) activity. We observed that the normal regenerative process requires a transient activation and silencing of approximately two dozen of ISPs. After nHx, the *Akt Pathway* represented with 13 branches, the *Chromatin Pathway* and the *DDR Pathways* dominated. After eHx, the *ATM main pathway* and two of its branches (*Cell Survival*; *G2_M Checkpoint Arrest*) dominated, as well as the *Hypoxia Pathways*. Further, 14 ISPs demonstrated a strong inverse regulation, with the *Hedgehog* and the *Brca1 Main Pathways* as chief activators after nHx, and the *ATM Pathway*(*G2_M Checkpoint Arrest*) as the dominating constraining response after eHx.

## 1. Introduction

The liver has the exceptional ability to regenerate after injury or surgical resection of tissue. It is widely recognized that this regenerating process is governed by an intricate interplay of hundreds of genes, which together form dozens of interwoven networks of signaling pathways. These pathways regulate biological processes, such as cell cycle arrest, cell cycle progression, apoptosis, inflammation, angiogenesis, etc. The entire process of liver regeneration is broadly defined by three distinct phases: an initiation phase, a proliferation phase, and a termination phase. Traditionally, it is thought that during the initiation phase, key signaling pathways induced by TNF-α and IL-6 drive cell proliferation in several hepatic cell types [1]. Then, growth factors and cytokines guide the progression of liver regeneration through expression of several cell-cycle-related proteins mainly by PI3K/AKT, wnt/β-catenin, Ras/MAPK, and JAK/STAT signaling pathways [2]. Equally important are pathways that control the speed of proliferation and determine the terminal point of liver regeneration [3]. Previously, we demonstrated that expression and activation of p21, an inhibitor of a subset of cyclin dependent kinases including cdc2, is a pivotal factor in arrested liver regeneration [4]. P21 is a fundamental cell cycle control checkpoint regulator, in particular, as it is inhibiting the G2-M phase progression. As such, it is downstream of many signaling networks, including those induced by p53, injury, growth factors (TGF-β), and inflammatory cytokines [3].

Limits to physiological liver regeneration are experienced in the clinic. Following extended hepatectomy that leaves behind a marginal liver remnant, liver failure may develop, a syndrome known as the small-for-size syndrome (SFSS). The SFSS is typified by metabolic liver dysfunction and represents the most frequent cause of postoperative death due to liver surgery [1]. Extended resection however is the most frequent intervention against highly prevalent liver tumors, which can be cured through complete removal only. The SFSS is currently untreatable and hence creates a medical conflict in that it limits the surgical cure of liver cancer [1,4]. Despite numerous studies addressing normal liver regeneration, we still lack comprehensive understanding of the biological processes underlying normal and failed liver regeneration. Such knowledge is required to categorize the biological processes of failed liver regeneration as observed in the SFSS.

With the development of reliable surgical mouse models, which reflect normal and extended liver hepatectomy in humans, and the advent of appropriate bioinformatics tools to analyze genome wide gene expression profiling, comprehensive categorization has now become feasible. To identify the intracellular signaling pathways (ISP) involved in normal and extended liver regeneration, we applied the OncoFinder algorithm for the functional annotation of the primary next generation sequencing genome-wide expression data. The advantage of OncoFinder over alternative tools, such as Metacore and Ingenuity Pathway Analysis (IPA), is that it calculates the pathway activation strength (PAS), so that differences between the two surgical procedures can be quantified [5,6].

Here, we analyzed the expression profiles of >20,000 genes and determined how they interact together in 271 IPA-defined intracellular signaling pathways. Our analyses of more than 12 million data points demonstrate that liver regeneration is a highly orchestrated flowing process, in which many signaling pathways operate simultaneously, either additively or inversely. We also show that the distinct stages of normal and failed regeneration are clearly discernable by a discrete activation and deactivation of a small number of signaling pathways. The five time points we studies in our nHx and eHx models were clearly demarcated by only a limited number of pathways, which may reflect normal and abnormal regeneration processes—the latter leading to liver failure and/or delayed regeneration.

## 2. Materials and Methods

### 2.1. Animals

All animal experiments were performed in accordance with Swiss Federal Animal Regulations and approved by the Veterinary Office of Zurich. Animals aged 10–12 weeks were kept on a 12-h day/night cycle with free access to food and water. C57Bl/6 mice were obtained from Harlan (Horst, The Netherlands).

### 2.2. Animal Surgery

**Figure 1:**
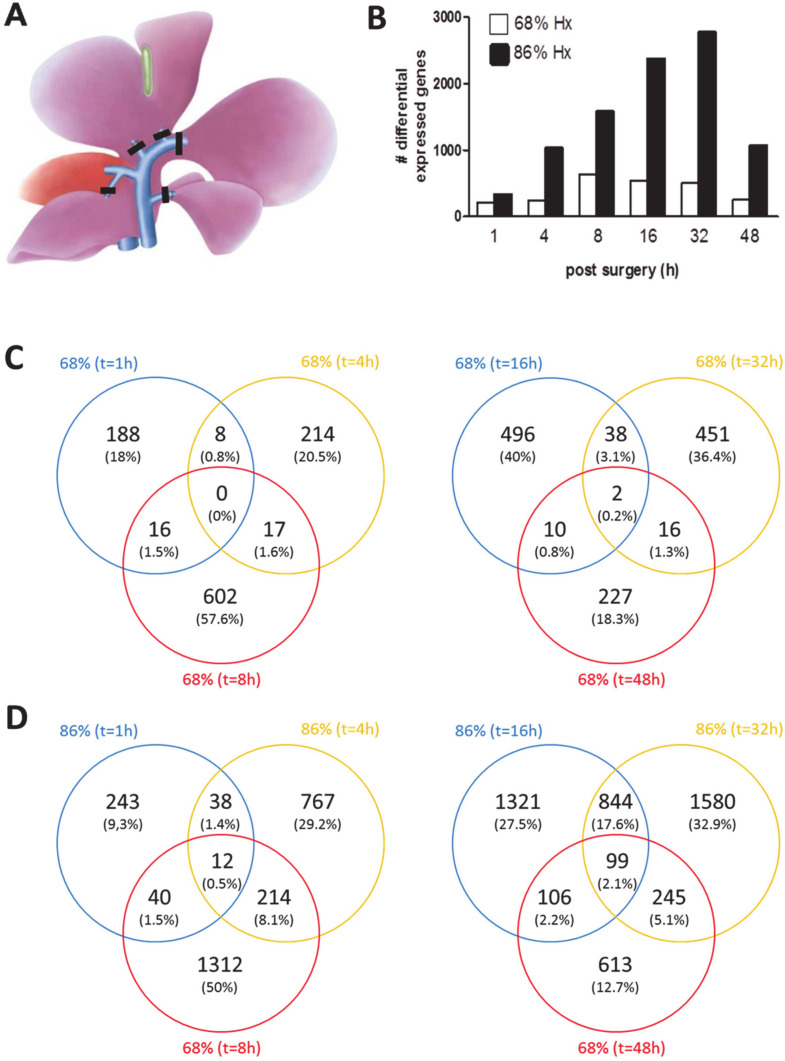
Candidate gene expression in nHx and eHx. (**A**) Schematic representation of the surgical procedure for extended resection of mouse liver. Segmental portal vein branches are ligated separately before parenchymal transection. (**B**) Candidate gene expression at different time points after nHx and eHx (for details on genes and expression levels see Supplementary dataset S1). (**C**) During the early phase after 68% hepatectomy (1–8 h post OP), no common genes were detected, whereas only 2 genes are common during the late phase (16–48 h post OP). (**D**) During the early regeneration phase of 86% hepatectomy (1–8 h post OP), 12 common genes were detected), whereas 99 genes are common genes were detected during the late phase (16–48 h post OP).

Animal surgery was performed as described by our team [4]. In short, for normal hepatectomy (68% hepatectomy), a midline incision was performed, and the liver was freed from ligaments. The pedicle of the left lobe was ligated (silk, 6/0), and resected. After cholecystectomy (Prolene, 8/0; Ethicon, Neuchatel, Switzerland), the middle lobe was ligated in 2 steps (silk 6/0) and resected. For extended hepatectomy (extended 86% hepatectomy [eHx]), all segmental portal vessels of the caudate, right anterior, left, and middle lobes were ligated individually (Prolene 8/0). The parenchyma was transected with silk 6/0 ligatures afterward. Vascular and biliary structures of the right posterior lobe were preserved by this technique and visually controlled (Figure 1A).

### 2.3. Liver and RNA Isolation

To study uncompromised liver regeneration, normal hepatectomy (68%) was performed, whereas extended hepatectomy (86%) was completed to induce experimental SFSS. Following 68% hepatectomy, injury is negligible, with ALT <100 U/L 1 day post-operation and serum bilirubin not elevated. After 86% hepatectomy, liver starts to gain weight very slowly, if at all, with its strongest gains toward 48 h. This time point coincides with the hepatocellular mitotic peak, which follows cell cycle entry around 16–20 h post 68% hepatectomy [4]. Mice were sacrificed at 5 time points after hepatectomy (1, 8, 16, 32, and 48 h) and livers were harvested. Immediately, liver tissue (30 mg) was homogenized and total RNA was isolated using TRIzol (Life technologies) following on-column DNase treatment (QIAGEN, Hombrechtikon, Switzerland). To exclude the possible confounding effect of laparotomy, sham operations were performed to set the baseline in further analyses.

### 2.4. cDNA Library Construction and RNA Deep Sequencing

Total liver RNA was prepared by DNA Column Clean-up (Qiagen, Basel, Switzerland) and purified, enriched, and fragmented following the protocol of Illumina TruSeq [7]. RNA deep sequencing using poly-A selection was performed using the Illumina Hiseq 2500. A QC report was generated and two-group analyses (comparing all procedures normalized to sham) were performed by the Functional Genomics Center, Zürich, to retrieve differential gene expression data (|log2FC = 1|; *p*-value ≤ 0.05). Venn diagrams were constructed using Venny 2.1 (http://bioinfogp.cnb.csic.es/tools/venny/). All data were deposited to the European Nucleotide Archive (ENA; accession number: PRJEB15593)

### 2.5. Functional Annotation of Gene Expression Data

For the functional annotation of the primary gene expression data, we applied our original algorithm termed OncoFinder [5,8]. It enables calculation of the Pathway Activation Strength (PAS), a value that serves as a qualitative measure of pathway activation. Briefly, the enclosing algorithm utilizes the following formula to evaluate pathway activation:

(1) $$PAS_{p}=\sum_{n}ARR_{np}\cdot BTIF_{n}\cdot lg\left(CNR_{n}\right).$$

Here, the case-to-normal ratio (CNRn) is the ratio of expression levels for a gene n in the sample under investigation to the same average value for the control group of samples. The Boolean flag of BTIF (beyond tolerance interval flag) equals to one for genes with significantly altered expression, and to zero for non-significantly affected genes. The applied significance criteria were as follows: differential gene had to meet simultaneously the two conditions, first, gene expression level for the sample must lie outside the tolerance interval (*p* < 0.05), and second, the value of CNR must differ from 1 considerably, thus being inferior of 0.66 or exceeding 1.5. The discrete value of ARR (activator/repressor role) reflects the functional role of a protein n in the pathway [5,8].

### 2.6. Source Datasets

The intracellular signaling pathways (ISPs) knowledge base developed by SABiosciences was used to determine structures of ISPs (https://www.qiagen.com/us/shop/genes-and-pathways/pathway-central/), which were used for OncoFinder, as described previously [5,8]. The OncoFinder software correctly identified ISPs known to be involved in liver regeneration.

### 2.7. Statistical Tests

The PAS values for each normal sample were obtained using the whole set of these normal samples as a reference. Distribution of PAS values was estimated, assuming its Gaussian behavior. Then, for each pathway of each sample, a probability that its PAS value comes from this estimated distribution was calculated. Additionally, p-values and false discovery rate (FDR) for each pathway of the entire group of samples were calculated using Wilcoxon rank-sum test and Benjamin-Hochberg method, respectively. Principal component analyses were performed using MADE4 package [9]. Hierarchical clustering heatmaps with Pearson distance and average linkage were generated using heatmap.2 function from “gplots” package [10]. Pearson tau correlation matrix was calculated in R 3.1.1 using a function of standard library “cor” with the default settings. Correlation diagram was built using a function “corrplot” from the package “corrplot” sorted with respect to hierarchical clustering. Similarities between the pathways according to the content of similar genes were calculated using Jaccard coefficient. The Jaccard coefficient measures similarity between finite sample sets, and is defined as the size of the intersection divided by the size of the union of the sample sets.

### 2.8. miRNA Target Prediction

Focusing on differentially expressed pre-miRNAs present in our datasets, we predicted their putative mRNA targets considering only experimentally validated miRNA–mRNA interactions using the Ingenuity Pathway Analysis (IPA) suite (Qiagen, Redwood city, CA, USA), which specifically uses TarBase [11], miRecords [12], and Ingenuity Expert Findings data sets to implement this task. Among all miRNA-targeted mRNAs, only genes having at least 10 reads (read count ≥10) were considered true targets for differentially expressed miRNAs in liver cells.

## 3. Results

### 3.1. Transcriptional Landscape of nHx and eHx Changes Over Time

Differential expression analyses revealed a total of 11,546 differentially expressed candidate genes (|log_2_FC = 1|; *p*-value ≤ 0.05), i.e., genes that are uniquely expressed after either nHx or eHx. As demonstrated in Figure 1B, the expression of candidate genes fluctuated over time and heavily depended on the surgical procedure, with the most dramatic effects observed after eHx. One hour after surgery, 212 and 333 candidate genes were detected for nHx and eHx, respectively. After 8 h, 635 (nHx) and 1578 (eHx) candidate genes were present. Sixteen hours after surgery, 546 (nHx) and 2374 (eHx) candidate genes were detected. After 32 h, 507 (nHx) and 2772 (eHx) genes were affected, whereas 255 (nHx) and 1063 (eHx) candidate genes were present 48 h post-surgery.

After both nHx and eHx, we observed a distinctive and sequential activation of unique candidate genes, with only a minor overlap of genes over time (Figure 1C,D). We did not observe common genes in 68% hepatectomy during the early phase after surgery (1–8 h post OP) and only 2 common genes (Cd46, Fam102b) during the late phase (16–48 h), indicating that a very distinct set of candidate genes is successively regulating the normal regeneration process. During extended hepatectomy, we observed 12 common genes (4930518I15Rik, Cdk20, Zfp235, Tcta, Coro1a, Sh2b2, Ptpre, Csf3r, 1810033B17Rik, Slfn4, Foxs1, and Il6) during the early phase after extended hepatectomy (1–8 h post OP). During the late phase after extended hepatectomy (16–48 h post OP), we determined 99 common candidate genes (Supplementary Data S1). Most of the reported functions of these genes, except II6 (which codes for interleukin-6), are completely uninformative in the context of liver regeneration. For instance, the *Tcta* gene, which is short for “T cell leukemia translocation altered gene”, is conserved in organisms ranging from Drosophila to humans and encodes a predicted M(r) 12,000 protein without strong homology to any previously reported proteins and expressed ubiquitously in all normal tissues [13]. To put these data in a sensible biological context, we comprehensively analyzed them using OncoFinder [5,8].

### 3.2. Building ISPs Activation Profiles

**Figure 2:**
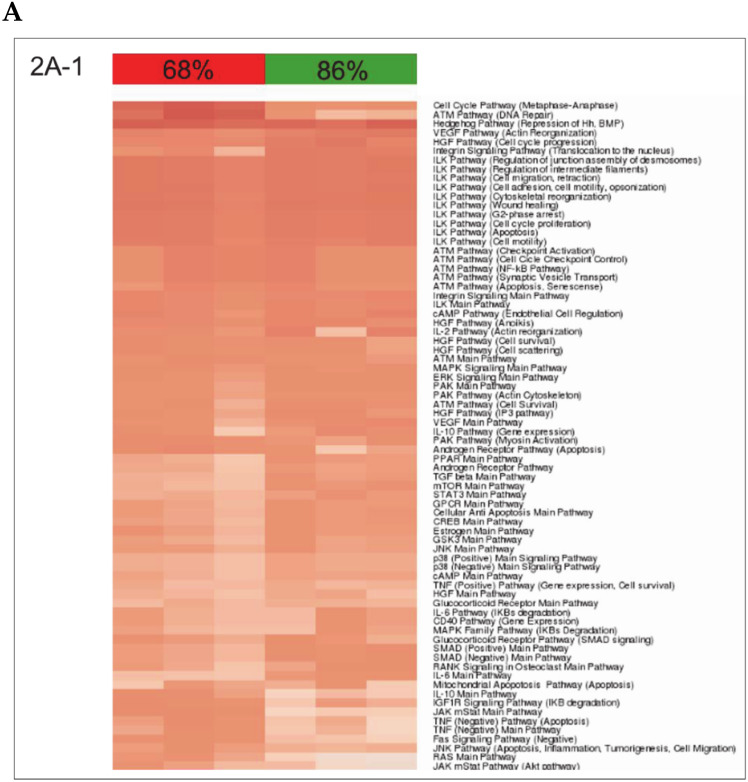

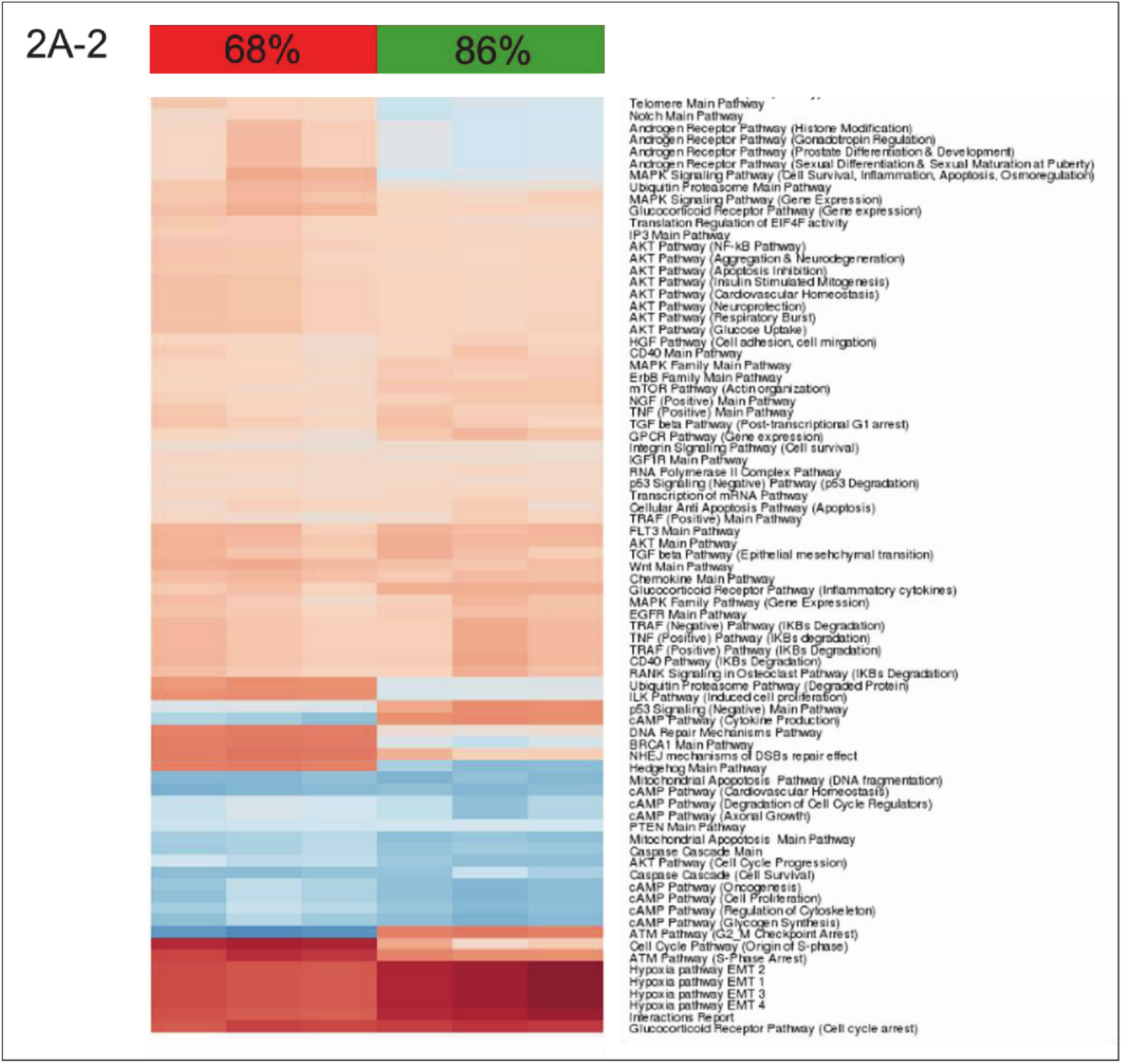

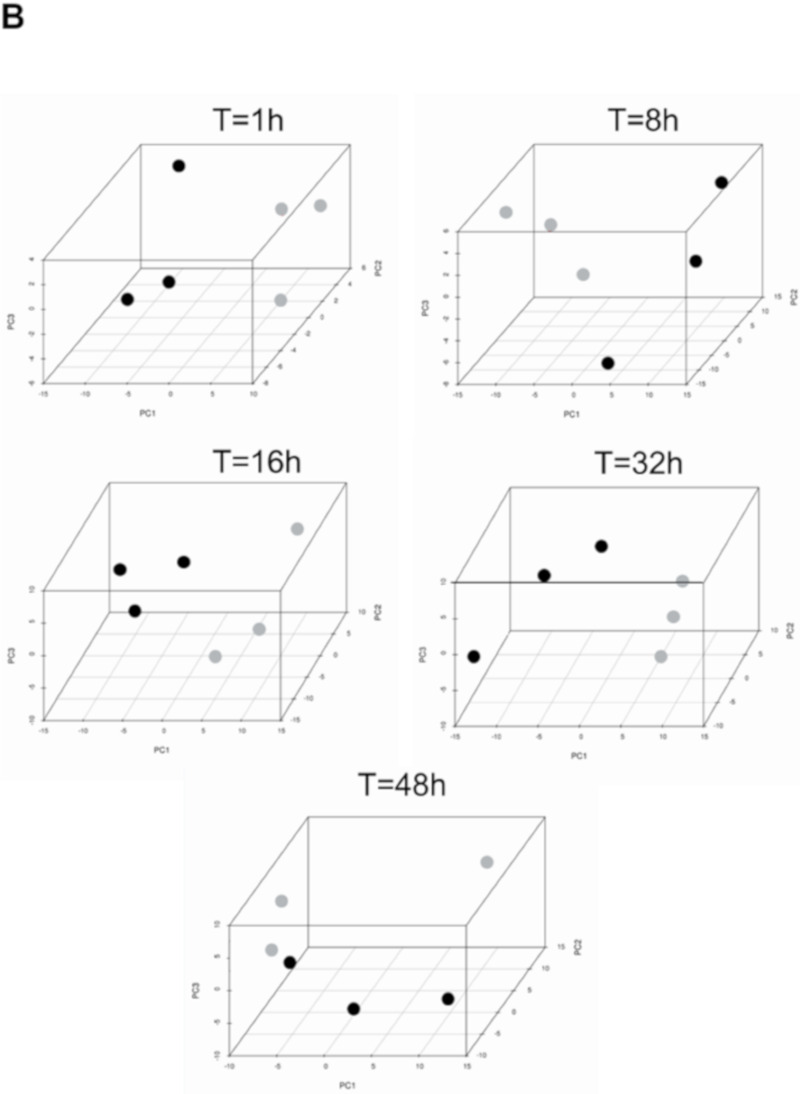

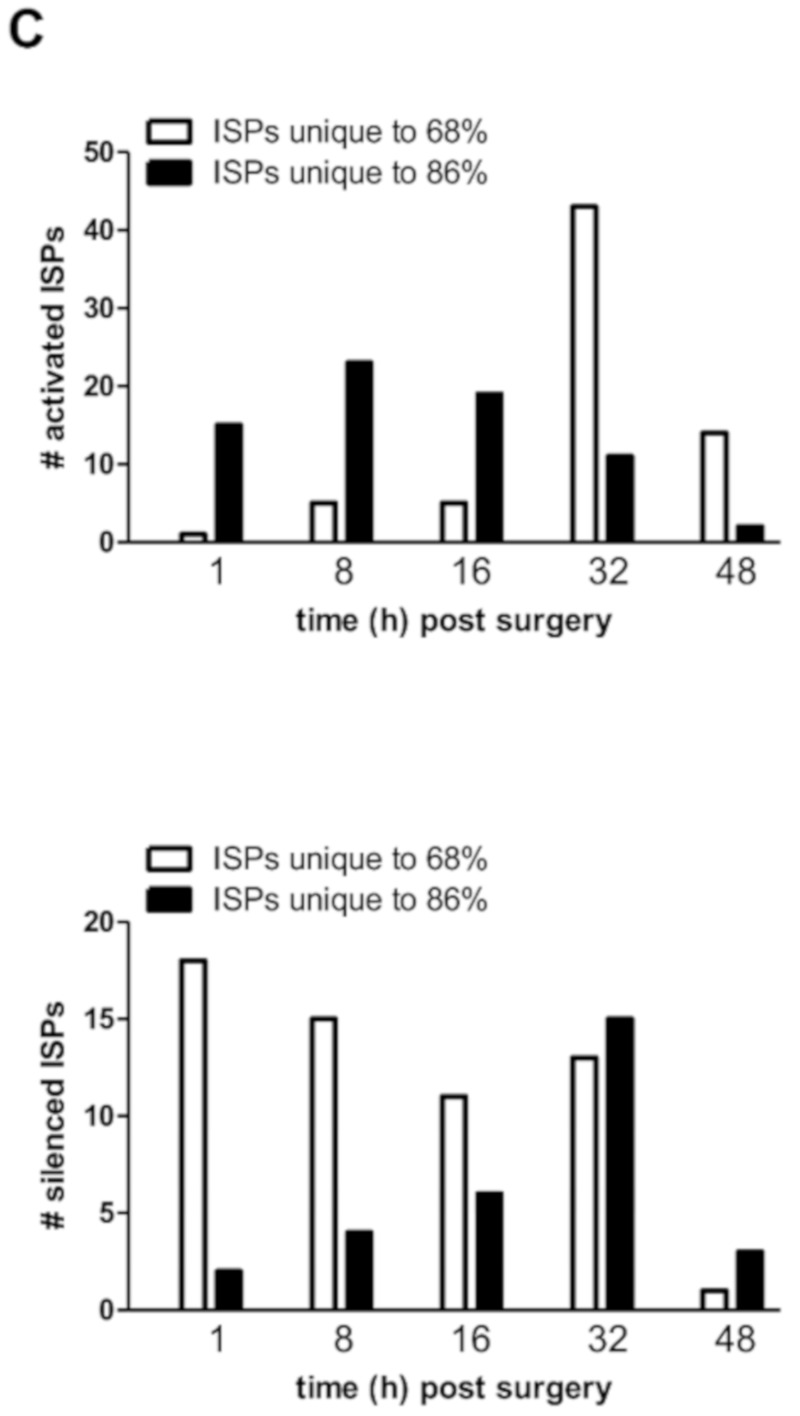
Modulation of intracellular signaling pathways (ISPs) after nHx and eHx. (**A**) Hierarchical clustering heatmap 32 h post-surgery based on the analysis of 271 intracellular signaling pathway activation profiles. Color key represents PAS score for a given pathway in a given sample. (**B**) Results of the principal component analysis. Groups were analyzed according to their PAS score signatures calculated for 271 intracellular signaling pathways. Black dots denote nHx samples; grey denote eHx samples. (**C**) Number of unique, activated ISPs after nHx and eHx at 1, 8, 16, 32, and 48 h post-surgery (top); Number of silenced ISPs after nHx and eHx at 1, 8, 16, 32, and 48 h post-surgery (bottom).

The normalized gene expression levels of our experimental groups were processed using the OncoFinder algorithm to calculate individual regulatory pathway activation strengths (PAS) profiles of 271 ISPs [5,8]. The PAS data, together with corresponding P values, are shown in the Supplementary Data S2. Using these data, we built hierarchical clustering heat maps with Euclidian distance and average linkage for all samples investigated for all time points (Supplementary Data S3). The most distinctive ISP differences between nHx and eHx were observed 32 h post-surgery (Figure 2A-1,2; summarized in Figure 2C). The two procedures can be clearly discerned based on PAS values, indicating that the both nHx and eHxe associated with multiple common changes in signaling pathway activities—changes that are specific and unique to one of both procedures. Principal Component Analyses confirmed this observation, demonstrating that both procedures formed clear distinctive groups, particularly 16 h and 32 h after surgery (Figure 2B).

### 3.3. Common and Unique ISPs after nHx and eHx

To identify the most significant ISPs involved in normal and delayed liver regeneration, we systematically compared all corresponding PAS values calculated for nHx and eHx over time (Table 1; Supplementary Data S4). One hour post-surgery, 102/271 (38%) pathways displayed *increased* activities of which the major part (86/102; 84%) overlapped, the latter indicating common intracellular activities. In the nHx group, one unique ISP was activated (Table 1; Supplementary Data S4). In sharp contrast, 15 signaling pathways were activated 1 h post extended hepatectomy. Further, we identified 23/271 (9%) pathways with decreased activities (summarized in Table 2). Thirteen of those were exclusively observed in the nHx group, two were only found in the eHx group, whereas eight were common to both surgical procedures (Supplementary Data S4, p1-4). Eight hours post-surgery, 141/271 (42%) pathways displayed increased activities (Supplementary Data S4, p5–10). Of those, five ISPs were uniquely upregulated after nHx, whereas the activities of 23 unique ISPs were increased after eHx (Table 1). Further, 97/271 (36%) ISPs presented with *diminished* activities. We observed that 15/97 were exclusively downregulated after nHx, whereas 4/97 were unique for the eHx procedure (Table 2). Sixteen hours post-surgery, 130/271 (48%) pathways presented with *increased* activities (Supplementary Data S4, p10 –14). Again, only five ISPs were uniquely activated after nHx, whereas 19 distinctive ISPs were upregulated after extended hepatectomy (Table 1). In addition, 60/271 (22%) ISPs presented with *diminished* activities. Of those, 11 were exclusively downregulated after nHx, whereas six were distinctively down after the extended procedure (Table 2).

Thirty-two hours post-surgery, 185/271 (69%) pathways displayed *increased* activities of which 131 were shared between the two surgical procedures (Supplementary Data S4, p15–20). We observed 43 and 11 demarcating ISPs after nHx and eHx, respectively (Table 1). In addition, we observed that 41/271 (15%) ISPs presented with *lower* activities (Table 2). Of those, 13 were unique for the normal procedure, whereas 15 were only found after extended surgery.

After 48 h, 172/271 (64%) ISPs presented with augmented activities (Supplementary Data S4, p20–25). Fourteen ISPs were uniquely activated after nHx, whereas the activities of two distinctive ISPs were increased after eHx (Table 1). In addition, 45/271 (17%) ISPs presented with *diminished* activities. Of those, only one ISP was exclusively down after nHx, whereas three were distinctively lower after the extended procedure (Table 2). Taken together, we identified 68 activated and 53 silenced signaling pathways specific for nHx. Likewise, 70 activated and 30 silenced signaling pathways were specific for eHx. Overall, nHx is associated with an early silencing and a late activation of a very distinct set of ISPs (for all pathways see Supplementary Data S4), whereas eHx is accompanied with an early activation and late silencing of its own specific assortment of ISPs (Figure 2C).

### 3.4. Inversely Regulated ISPs after nHx and eHx

Additional ISPs, which may potentially determine the outcome of liver regeneration, are those that are inversely regulated after the two surgical procedures. These pathways are activated after 68% hepatectomy and silenced after 86%, or vice versa. We identified a total of 33 common pathways with opposite signs for the calculated PAS values (Table 3 and Supplementary Data S4, p29–34). Among those, 14 ISPs presented with a differential PAS ≥ 0.1, indicating strong inverse regulation after 68% and 86% hepatectomy (highlighted in Table 3). During the early phase after nHx, the *ATM Pathway* (*G2_M checkpoint arrest*) and the *CD40 Pathway* (*cytokine expression*) are markedly silenced (PAS values are −0.115072046 and −0.106856601, respectively), whereas the *Circadian Main Pathway* is activated (PAS: 0.119250412). After 32 h, normal liver regeneration is predominantly dictated by the activation of the *Hedgehog Main Pathway* (PAS=0.261337874) and the *BRCA1 Main Pathway* (PAS: 0.292340978). The *ATM Pathway* (*G2_M checkpoint arrest*) is the most silenced pathway 48 h after surgery (PAS = −0.651298809). During the early phase after extended hepatectomy, the *Cytokine Main Pathway*, the *ErbB Family Main Pathway*, and the *Mitochondrial Apoptosis Pathway* are the most activated ISPs (PAS values are 0.110941123, 0.124348982, and 0.181980954, respectively), followed by the *IL10 Pathway* 16 h after surgery (PAS = 0.109013662). In contrast to nHx, the *Hedgehog Main Pathway* is markedly silenced (PAS = −0.141280757). Likewise, 48 h after eHx, the *ATM Pathway* (*G2_M checkpoint arrest*) is most significantly activated (PAS=0.282117965), as well as the *cAMP Pathway* (Cytokine Production) and the p*53 Signaling (Negative) Main Pathways* (PAS values are 0.140344644 and 0.110310831, respectively).

### 3.5. Target Prediction for Differentially Expressed miRNAs

**Figure 3:**
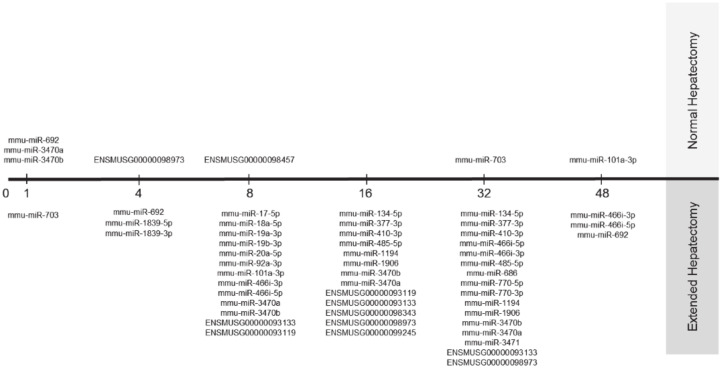
Changes of miRNA expression patterns over time after nHx and eHx. Two novel miRNAs are expressed after nHx, whereas five novel miRNA were identified after eHx (ENSMUS classification indicated).

**Figure 4:**
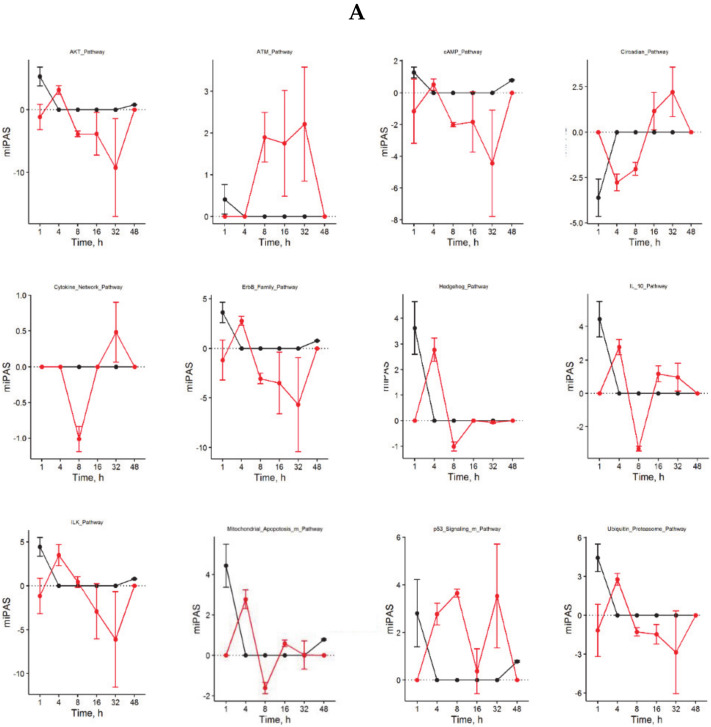

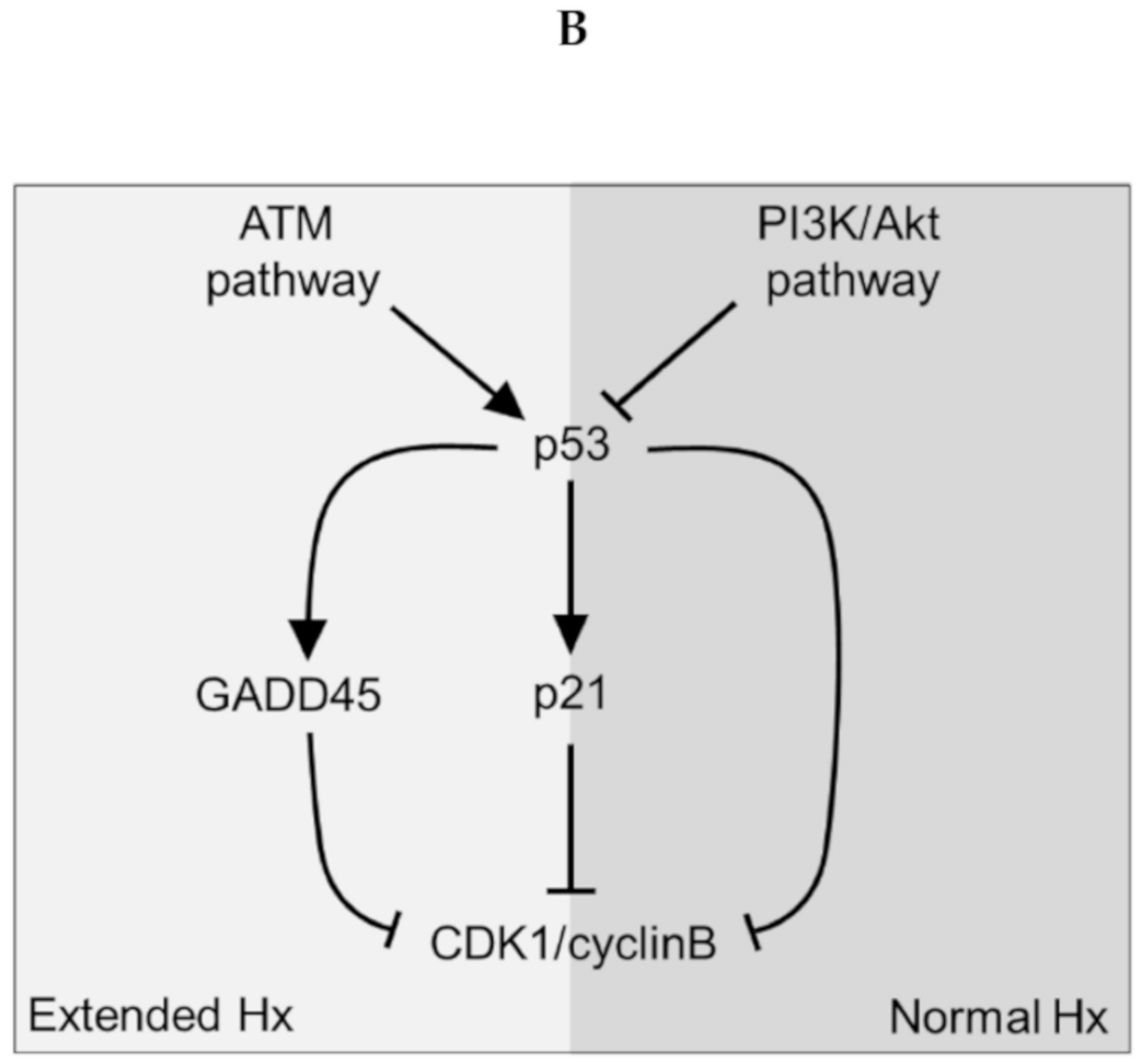
IPA functional analysis on the set mRNAs targeted by the recognized miRNAs. (**A**) Plotted PAS values for miRNA-affected ISPs after nHx (black) and eHx (red). (**B**) Schematic presentation how the *ATM-* and the *PI3K/Akt Signaling Pathway* are integrated at the molecular level. After nHx, p53 is inhibited by the *PI3K/Akt Signaling Pathway* resulting in lower expression of the cell cycle inhibitor p21, so that the cell cycle block (CDK1/cyclin B) is released and proliferation is induced. Overstimulation of the *ATM Signaling Pathway* bypasses the PI3K/Akt pathway to further activate the p53-p21 axis 48 h after eHx only (see Table 3). Hence, the *ATM Signaling Pathway* induces an additional eHx-specific proliferation block via GADD45, the inhibitor of CDK1/cyclinB. Arrows: stimulation; Blunt arrows: inhibition.

Using IPA software, we identified experimentally validated targets for differentially expressed pre-miRNAs (Supplementary Data S5, p1–16). After removal of possible miRNA-targeted mRNAs transcripts not expressed in the liver, we found 40 miRNAs of which 25 targeted 359 mRNAs, whereas the remaining 15 miRNAs presented as novel miRNAs without experimentally evaluated molecular targets. The distribution and changes of miRNA expression patterns over time after nHx and eHx are presented in Figure 3. Seven of the identified miRNAs were present after nHx (5 known, 2 novel; ENSMUS classification indicated in Figure 3), whereas 30 miRNAs were observed after eHx (25 known, 5 novel; ENSMUS classification indicated in Figure 3). Since the high number of pre-miRNA present after eHx submits a possible leading role in the biological processes involved in failed/delayed liver regeneration, we determined how the corresponding miRNA interact with the ISPs presented in Table 3. The plots presented in Figure 4A demonstrate how these miRNA affect the activity of the ISP earlier identified as the key cellular processes induced by nHx and eHx. Overall, the miRNAs would predominantly exert a negative effect on the PAS values of eHx-induced signaling, with an optimum at 32 h post-surgery. Only the *ATM Pathway* and the *p53 Main Pathway* showed clear positive PAS values, also peaking at 32 h (Figure 4A; Supplementary Data S5, p17–24).

## 4. Discussion

Failed liver regeneration after major resection has been a subject of intense scrutiny, as it posits a severe restraint to liver surgery. Resection beyond 70 percent of the total liver mass is critical, since it often leads to the small for size syndrome (SFSS), a pathology characterized by hyper-bilirubinemia, diminished (or failed) liver function, and, as such, is the most frequent cause of death due to liver surgery. Earlier, we demonstrated that p21 activation mediates a transient barrier to the progression and completion of the cell cycle, thereby inhibiting liver regeneration and deteriorating survival [4]. To comprehend the molecular background of SFSS, however, the total of regulatory processes operating in the liver during normal and failed/delayed regeneration should be integrated, a challenge, which can only be overcome using bioinformatics approaches. This study is the first systematic and comprehensive analysis of liver-expressed genes to profile intracellular signaling pathways (ISPs) after nHx and eHx. Our study submits that relative to eHx, nHx requires a lower level of gene activation with an optimal expression of candidate genes around 8 h post-surgery. Thereafter, gene activation rapidly returns to control levels (Figure 1B). In contrast, eHx is associated with a 3- to 4-fold overshoot of differentially regulated candidate genes, which is most pronounced 32 h post-surgery. Forty-eight hours after the extended procedure, the liver seems to have mastered the affliction and gene expression levels start to go down. Relative to the normal procedure, however, there is still an enormous overshoot of genetic activity. These facts indicate that normal liver regeneration requires a stringent control over gene expression, which seems to be transiently, but severely, disturbed after extended hepatectomy.

How all the liver-expressed genes interact together and affect biological relevant processes was studied using OncoFinder, software designed to quantify pathway activation strengths according to gene expression levels [5,8]. We observed that normal liver regeneration is an orchestration of the sequential silencing and activation of several dozen cooperating ISPs (Figure 2C). Importantly, the early phase of normal liver regeneration is defined by a predominant silencing, rather than an activation of ISPs. Then, during the late phase of normal liver regeneration (32–48 h), whole arrays of ISPs are being activated simultaneously but transiently. The opposite was observed after extended hepatectomy, which resulted in an immediate activation of a plethora of signaling cascades. Therefore, delayed (or failed) liver regeneration may result from a hyper-activation, rather than hypo-activation (or silencing) of ISPs. The earlier described activation of p21 by Lehmann et al. [4] may represent the ensuing biological response, since it would transiently stall the cell cycle, so that (epi)genetic salvage programs can be initiated.

A closer look at the identified ISPs revealed that both procedures recruit pathways that traditionally have been linked to liver regeneration. The most pronounced common pathways activated after both nHx and eHx include the *Hedgehog Pathway* (Figure 2A-1) and four branches of the *Hypoxia Pathway* involved in epithelial-mesenchymal transition (Figure 2A-2). In addition, we identified the *IL10 Pathway* as an important ISP after both normal and extended hepatectomies. A pleiotropic cytokine with important immuno-regulatory functions, IL-10 may act to silence the initial inflammatory response through counteracting the expression of inflammatory cytokines such as TNF-α, interleukin (IL)-6, and IL-1 by activated macrophages [14]. The most distinct common pathways silenced after both nHx and eHx are the seven branches of the *cAMP Pathway*. In the liver, cAMP-responsive signaling plays an important role, particularly in regulating hepatocyte proliferation as observed after nHx [15]. In addition, silencing of the cAMP-induced pathway may prompt hepatic cells to become less committed and prepare them for cell cycle entry [16]. Most PAS values presented in Figure 2A-1 demonstrate only minor differences between the two surgical procedures. Still, small but significant differences in PAS values may lead to significant biological effects, as they represent the additive activation strengths of the components of the pathway as a whole [5,8]. More pronounced differences also reflect stronger involvement in the pathological processes observed after delayed liver regeneration.

Among the unique ISPs activated after nHx, the *Akt Pathway* represented with 13 branches, the *Chromatin Pathway* and the *DDR Pathway*—with 3 branches each—dominated (Table 1A). The *Akt Pathways*, which are triggered essentially by growth factors, induce the progression of G1→M and G2→M transitions via silencing the activity of p53 and p21, two proteins crucial to cell cycle entry. The *chromatin-* and *DDR Pathways* signify the importance of epigenetic configurations after nHx. Crucial for a normal regeneration process seems to be the sequential silencing of a score of ISPs, including most branches of the *ILK Pathway* during the late phase, which are not (yet) detected after extended hepatectomy (Table 2). Indeed, the integrin-linked kinase (ILK) is a protein involved in transmitting extracellular matrix signals and associated with the termination of liver regeneration [17].

Among the unique ISPs activated after extended hepatectomy, the *ATM Main Pathway* and two of its branches (*Cell Survival; G2_M Checkpoint Arrest*) dominated. Both pathways may be induced by severe (DNA) damage inflicted by extended hepatectomy, dramatically increased blood pressure, or both, which retain the cells in the G2 phase. In addition, five branches of the *HIF1-Alpha Main Pathway* (*Gene Expression Pathway; NOS Pathway; Pyruvate Pathway; VEGF Pathway*) prevailed. These pathways, which are closely associated with the *VEGF Main Pathway*, may reflect the immediate early hypoxic conditions after extended liver hepatectomy. Both nHx and eHx rapidly induced hypoxia-associated ISPs, an observation, which may reflect the overshoot of oxygen-poor blood delivered from the portal vein, and probably required for the early phase of liver regeneration through Hif2a [18]. Interestingly, the hypoxia-induced pathways are swiftly down-regulated after 32 h, but only following nHx. Hypoxia may thus be an important fine-tuning mechanism to induce liver regeneration, functioning within a very narrow window. Prolonged hypoxia-associated signaling may stall the normal regeneration process after eHx, however, as it may inhibit G1/S transitions through regulation of p27 expression [19]. Indeed, loss of p27 leads to accelerated DNA synthesis in hepatocytes DNA replication after partial hepatectomy in mice [20].

Fourteen ISPs demonstrated a strong inverse regulation after nHx and eHx (Table 3; PAS ≥ 0.1, highlighted). Together with the surgery-specific pathways discussed above, they may represent the most relevant pathways determining a healthy/normal or diseased/delayed regeneration process. Here, the *ATM Pathway* (*G2_M checkpoint arrest*) is of particular interest. Earlier, we observed that liver dysfunction after eHx resulted from a deficiency in cell cycle progression caused by a transient activation of p21 just before hepatocyte division, rather than from parenchymal injury [4]. Oncofinder identified the *ATM Pathway* (*G2_M checkpoint arrest*) with p53 and p21 as dominant cell cycle checkpoints, to be the most differentially activated pathway—thus confirming our earlier data. Therefore, a deficiency in cell-cycle progression after extended hepatectomy and concomitant liver failure might be overcome by counteracting this pathway. Our data suggest this can be achieved by stimulation of the Akt-associated pathways, which are explicitly activated early after nHx but not after eHx, and interfere with the ATM pathway at the level of p53, the activator of p21. The pre-miRNA identified in the eHx samples also indicated a strong reciprocal regulation of these pathways. Our comprehensive ISP analyses thus reveals potential intervention points to treat failed liver regeneration.

Taken together, a timely—but transiently—activation and silencing of approximately two dozen of ISPs warrant a normal regenerative process, culminating in full recovery of small liver remnants. The most affected ISPs are revolving around Akt-mediated signaling—which is tightly associated with several pathways, including the *JNK Pathway*, the *NF-κB,* and the *ERK Pathway*—and the *ATM Pathway* via the p53-p21 axis (Figure 4B). Together, they govern the G1/S Checkpoint and the G2/M DNA Damage Checkpoint, in which p21 and p53 are major executors. The miRNAs found exclusively after hepatectomy may interfere with Akt-signaling, thereby silencing the downstream cell cycle programs. Indeed, 20 of the identified miRNAs specific for extended hepatectomy target the transcripts of 18 genes operating directly or indirectly in the Akt pathway. One miRNA, mmu-miR692, is expressed immediately after normal regeneration and 48 h after extended hepatectomy, suggesting that it may prepare for a smooth regeneration process—as suggested by the predominant ISP silencing during these stages. Alternatively, the observed overload of pathway activities (Figure 1) after 86% may reflect a metabolic overload as a major cause of liver failure. Metabolic changes after hepatectomy are thought to provide regenerative triggers, but might also serve to satisfy energy demands. Liver is the major glucose provider, and hypoglycaemia inevitably develops when liver mass is lost. Indeed, hypoglycaemia is an essential regenerative signal [21]. Moreover, hypoglycemia is thought to trigger a systemic response leading to a redistribution of lipids from the periphery into the regenerating liver [22]. This could mean that delay or inhibition of proliferation is the consequence of hepatocyte functional impairment and not that of the cell cycle, persee. Indeed, mesenchymal stem cells have been reported to ameliorate hepatic dysfunction and improve liver regeneration after extended resection by paracrine, metabolic mechanisms [23]. Additional analyses into the metabolic pathways underlying liver failure are currently being addressed in our laboratory. In summary, our comprehensive analyses not only recapitulated current knowledge on normal liver regeneration processes by retrieving Hedgehog-, Hypoxia-, and p21-associated pathways from thousands of data points, they also disclosed novel genes and surprising connections not earlier reported. In particular, the involvement of the *CD40-*, the *BRCA1*, and the *Ubiquitin-Proteasome Pathways* are unexpectedly involved in the normal regeneration process, whereas the *IL10-*, the *cAMP*-, and the *ATM Pathways* are defining the ceased regeneration process. The importance of miRNAs governing the molecular principles underlying liver regeneration should also be envisaged. Liver-specific signaling networks are tightly controlled by miRNAs that regulate key hepatic functions during liver injury and disease. Several miRNA affect hepatocyte proliferation, including miR-21, miR-29a, and miR-382, which target genes at important signaling crossroads, such as the crucial PI3K/AKT-signaling mediator PTEN [24]. Still, miRNA data should be interpreted cautiously, because their expression depends on the genetic background, as well as on inter- and intra-species variability. The involvement of one—or a few—specific miRNA is not only very hard to establish, but also rather unlikely to be driving biological processes in which thousands of genes interact. In reality, extended arrays of differentially expressed miRNA determine the ultimate biological response.

## 5. Conclusions

Our analyses recapitulated current knowledge on liver regeneration processes by retrieving the Hedgehog-pathway the Hypoxia-pathways, and unveiled several novel p21-connected networks not earlier associated with liver regeneration. Overall, nHx is associated with an early silencing and a late activation of a very distinct set of ISPs, whereas an early activation and late silencing of its own specific assortment of ISPs characterize eHx.

## Figures and Tables

**Table 1:**
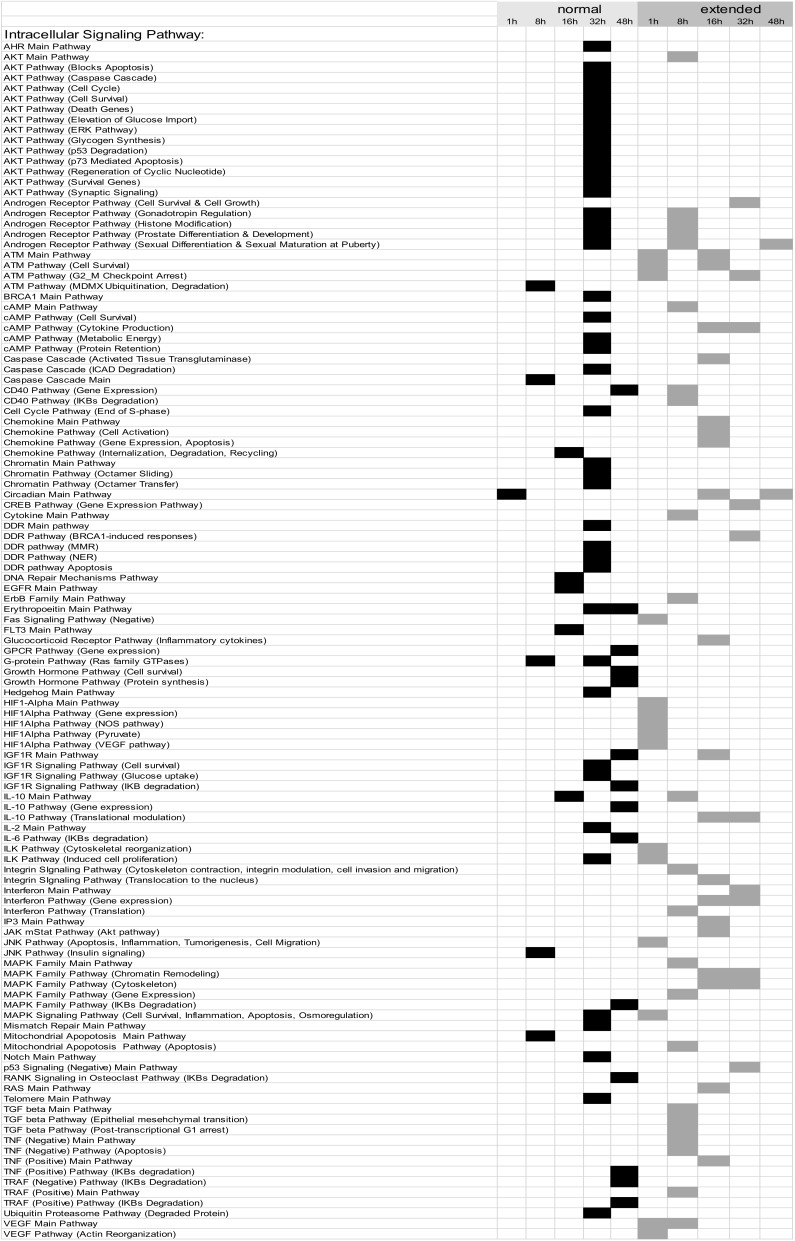
Physical properties of the test fuels.

**Table 2:**
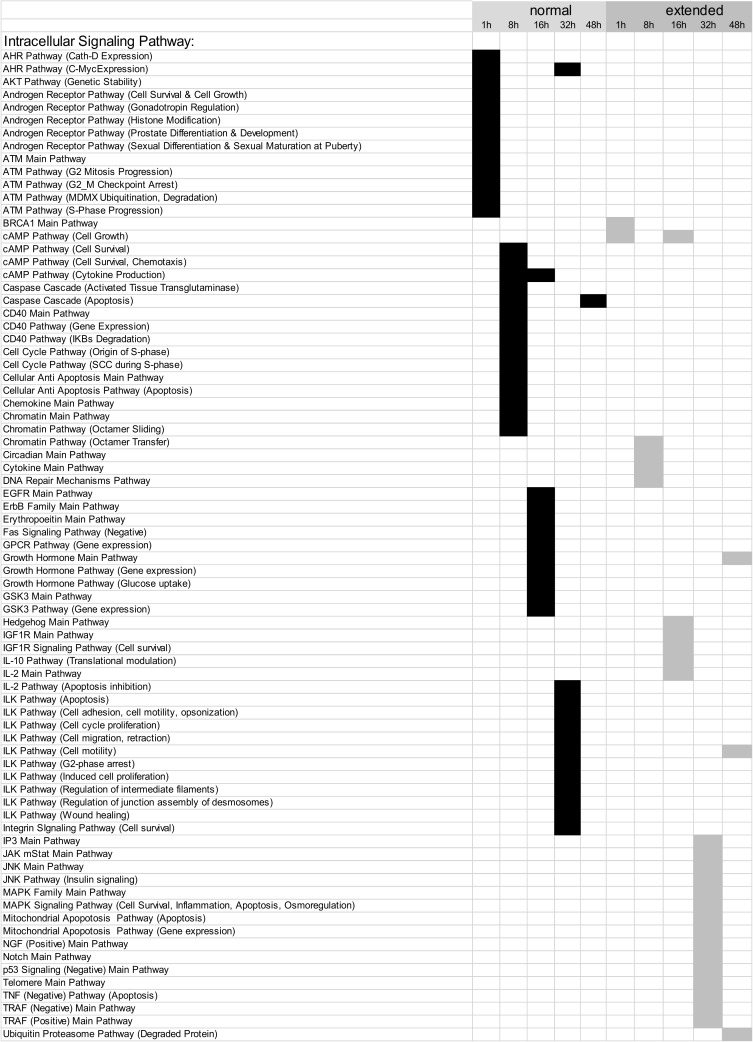
Physical properties of the test fuels.

**Table 3:**
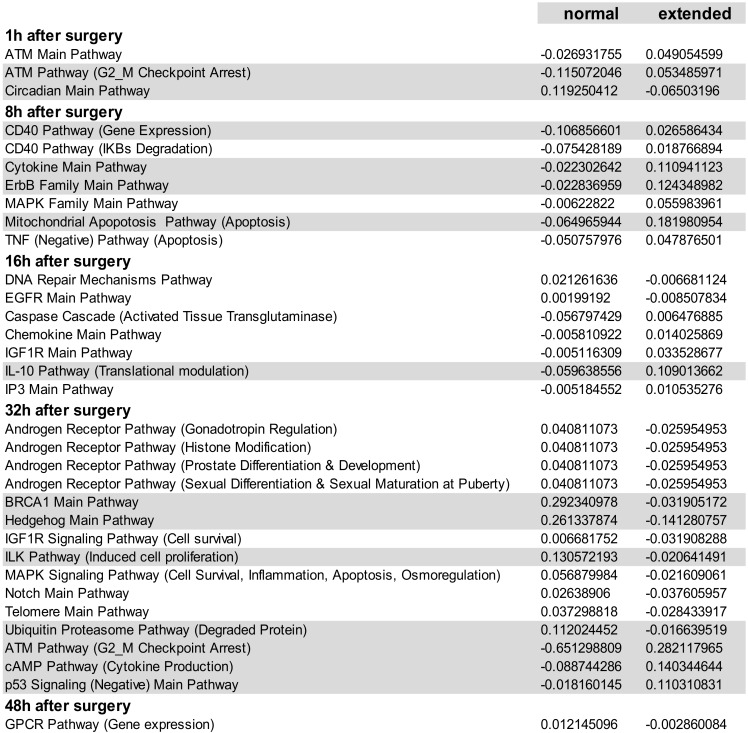
Physical properties of the test fuels.

## Supplementary Materials

The supplementary data are available online at https://www.mdpi.com/2073-4409/9/5/1149/s1.
