# Supplementary material for: Large-Scale Profiling of Signaling Pathways Reveals a Distinct Demarcation between Normal and Extended Liver Resection
Source: Cells. 2020 May 7;9(5):1149. doi: 10.3390/cells9051149 (PMC7290735; doi:10.3390/cells9051149)

Supplemental Data S-1

99 common genes in 86% hepatectomy during late phase (16-32h post OP):

610318N02Rik	Cdh5	Fam47e	Gprc5a
8430419L09Rik	Chic1	Fam71f2	Gstz1
A130051J06Rik	Chpt1	Fggy	Hcfc1r1
Acat3	Clip4	Firre	Iqgap1
Acrbp	Cmah	Fmn2	Kalrn
Adh5	Crp	Fmnl2	Khnyn
Adi1	Cyp8b1	Fxyd1	Kynu
Aldh6a1	Dgat2	Gar1	L3hypdh
Anks6	Dlgap2	Gm11789	Lbp
Ano6	Dpf3	Gm15614	Lif
C6	Egln3	Gm15939	Lrrc70
Capn8	Eif5a2	Gm21750	Mcc
Ccs	Erbb3	Gm5526	Mcee
Cd83	Fam46c	Gpr133	Mpdz

Mpst
Myc
Naprt1
Nlrp12
Parvb
Pde4d
Plxdc1
Prune
Rgl3
Ripk3
Rnd1
Rogdi
Sephs2
Sf3b3
Slc10a3
Slc27a2
Slc29a1
Slc2a5
Slc35e2
Slc35g2
Snai3
Sntb2
Snx32
Sowahb
Spag8
Sptlc2
St8sia3os
Svip
Tax1bp3
Tc2n
Tmem150a
Tmem229a
Tmem65
Toporsos
Tppp
Tspan2
Tspan33
Unc119
Zap70
Zfp773
Zfp937
Zfp953
Zfp964

Supplemental S-2

Pathway	M_68_1h_1	M_68_1h_2	M_68_1h_3	p-value_Mean
**AHR Main Pathway**	0	0	0	1
**AHR Pathway (AHR Degradation)**	0	0	0	1
**AHR Pathway (Cath-D Expression)**	0	0	0	1
**AHR Pathway (C-MycExpression)**	0	0	0	1
**AHR Pathway (PS2 Gene Expression)**	0	0	0	1
**AKT Main Pathway**	0.01599702	0.01322475	0.0168663	0.049534613
**AKT Pathway (Aggregation & Neurodegeneration)**	0.0210164	0.01867118	0.0191526	0.049534613
**AKT Pathway (Apoptosis Inhibition)**	0.02019223	0.01793897	0.0184015	0.049534613
**AKT Pathway (Blocks Apoptosis)**	0	0	0	1
**AKT Pathway (Cardiovascular Homeostasis)**	0.02288453	0.02033084	0.020855	0.049534613
**AKT Pathway (Caspase Cascade)**	0	0	0	1
**AKT Pathway (Cell Cycle)**	0	0	0	1
**AKT Pathway (Cell Cycle Progression)**	0.04926568	0.03249248	0.0181728	0.049534613
**AKT Pathway (Cell Survival)**	0	0	0	1
**AKT Pathway (Death Genes)**	0	0	0	1
**AKT Pathway (Elevation of Glucose Import)**	0	0	0	1
**AKT Pathway (ERK Pathway)**	0	0	0	1
**AKT Pathway (Genetic Stability)**	0	0	0	1
**AKT Pathway (Glucose Uptake)**	0.02340463	0.0207929	0.021329	0.049534613
**AKT Pathway (Glycogen Synthesis)**	0	0	0	1
**AKT Pathway (Insulin Stimulated Mitogenesis)**	0.02288453	0.02033084	0.020855	0.049534613
**AKT Pathway (JNK Pathway)**	0	0	0	1
**AKT Pathway (Neuroprotection)**	0.02288453	0.02033084	0.020855	0.049534613
**AKT Pathway (NF-kB Pathway)**	0.02145424	0.01906016	0.0195516	0.049534613
**AKT Pathway (p53 Degradation)**	0	0	0	1
**AKT Pathway (p73 Mediated Apoptosis)**	0	0	0	1
**AKT Pathway (Protein Synthesis)**	0	0	0	1
**AKT Pathway (Regeneration of Cyclic Nucleotide)**	0	0	0	1
**AKT Pathway (Respiratory Burst)**	0.02288453	0.02033084	0.020855	0.049534613
**AKT Pathway (Survival Genes)**	0	0	0	1
**AKT Pathway (Synaptic Signaling)**	0	0	0	1
**AKT Pathway (Translation)**	0	0	0	1
**Androgen Receptor Pathway**	0.01953019	0.01347931	0.0274531	0.049534613
**Androgen Receptor Pathway (Apoptosis)**	0	0	0	1
**Androgen Receptor Pathway (Degradation)**	0.02301891	0.01609388	0.0503994	0.049534613
**Androgen Receptor Pathway (Cell Survival & Cell Growth)**	0	0	0	1
**Androgen Receptor Pathway (Gonadotropin Regulation)**	0.09083207	0.06874262	0.1457075	0.049534613
**Androgen Receptor Pathway (Histone Modification)**	0.09083207	0.06874262	0.1457075	0.049534613
**Androgen Receptor Pathway (Prostate Differentiation & Development)**	0.09083207	0.06874262	0.1457075	0.049534613
**Androgen Receptor Pathway (Sexual Differentiation & Sexual Maturation at Puberty)**	0.09083207	0.06874262	0.1457075	0.049534613
**ATM Main Pathway**	-0.0398318	-0.0262705	-0.0146929	0.049534613
**ATM Pathway (Apoptosis)**	0	0	0	1
**ATM Pathway (Apoptosis, Senescense)**	0	0	0	1

**ATM Pathway (Cell Cicle Checkpoint Control)**	0	0	0	1
**ATM Pathway (Cell Survival)**	0	0	0	1
**ATM Pathway (Checkpoint Activation)**	0	0	0	1
**ATM Pathway (DNA Repair)**	0	0	0	1
**ATM Pathway (G2_M Checkpoint Arrest)**	-0.1701905	-0.1122468	-0.0627789	0.049534613
**ATM Pathway (G2 Mitosis Progression)**	0	0	0	1
**ATM Pathway (MDMX Ubiquitination, Degradation)**	0	0	0	1
**ATM Pathway (NF-kB Pathway)**	0	0	0	1
**ATM Pathway (Synaptic Vesicle Transport)**	0	0	0	1
**ATM Pathway (S-Phase Arrest)**	0	0	0	1
**ATM Pathway (S-Phase Progression)**	0	0	0	1
**DDR Pathway (BRCA1-induced responses)**	-0.1185748	-0.0691444	-0.0944495	0.049534613
**BRCA1 Main Pathway**	0.03915031	0.0292528	0.015938	0.049534613
**cAMP Main Pathway**	0.00663511	0.004523	0.0075243	0.049534613
**cAMP Pathway (Axonal Growth)**	0	0	0	1
**cAMP Pathway (Cardiovascular Homeostasis)**	0	0	0	1
**cAMP Pathway (Cell Growth)**	0	0	0	1
**cAMP Pathway (Cell Proliferation)**	0	0	0	1
**cAMP Pathway (Cell Survival)**	0	0	0	1
**cAMP Pathway (Cell Survival, Chemotaxis)**	0	0	0	1
**cAMP Pathway (Cytokine Production)**	0	0	0	1
**cAMP Pathway (Degradation of Cell Cycle Regulators)**	0	0	0	1
**cAMP Pathway (Endothelial Cell Regulation)**	0	0	0	1
**cAMP Pathway (Glycogen Synthesis)**	0	0	0	1
**cAMP Pathway (Glycolysis)**	0	0	0	1
**cAMP Pathway (Metabolic Energy)**	0	0	0	1
**cAMP Pathway (Myocardial Contraction)**	0	0	0	1
**cAMP Pathway (Oncogenesis)**	0	0	0	1
**cAMP Pathway (Protein Retention)**	0	0	0	1
**cAMP Pathway (Regulation of Cytoskeleton)**	0	0	0	1
**Caspase Cascade Main**	-0.0133437	-0.0091643	-0.014811	0.049534613
**Caspase Cascade (Activated Tissue Transglutaminase)**	0	0	0	1
**Caspase Cascade (Apoptosis)**	0	0	0	1
**Caspase Cascade (Cell Survival)**	0	0	0	1
**Caspase Cascade (ICAD Degradation)**	0	0	0	1
**CD40 Main Pathway**	0.02011994	0.01712993	0.0129543	0.049534613
**CD40 Pathway (Cell Survival)**	0	0	0	1
**CD40 Pathway (Gene Expression)**	0.08886307	0.0756572	0.0572148	0.049534613
**CD40 Pathway (IKBs Degradation)**	0.06272687	0.05340508	0.0403869	0.049534613
**Cellular Anti Apoptosis Main Pathway**	0.00857431	0.00730778	0.0111567	0.049534613
**Cellular Anti Apoptosis Pathway (Apoptosis)**	0	0	0	1
**Cellular Anti Apoptosis Pathway (Depolarization)**	0	0	0	1
**Chemokine Main Pathway**	0.01968196	0.0192395	0.030282	0.049534613
**Chemokine Pathway (Cell Activation)**	0	0	0	1
**Chemokine Pathway (Gene Expression, Apoptosis)**	0	0	0	1
**Chemokine Pathway (Internalization, Degradation, Recycling)**	0	0	0	1
**Chromatin Main Pathway**	0	0	0	1
**Chromatin Pathway (Octamer Sliding)**	0	0	0	1
**Chromatin Pathway (Octamer Transfer)**	0	0	0	1
**Circadian Main Pathway**	0.14738504	0.09705678	0.1133094	0.049534613
**CREB Main Pathway**	0.01170681	0.00746464	0.0146237	0.049534613

**CREB Pathway (Gene Expression Pathway)**	0	0	0	1
**Cytokine Main Pathway**	0.03153499	0.02974881	0.0738732	0.049534613
**DDR pathway Apoptosis**	0	0	0	1
**DDR Main pathway**	0	0	0	1
**DNA Repair Mechanisms Pathway**	0	0	0	1
**EGFR Main Pathway**	0.04771066	0.03939992	0.0788285	0.049534613
**ErbB Family Main Pathway**	0.05333454	0.04558723	0.1010988	0.049534613
**ErbB Family Pathway (Anti-Apoptosis)**	0	0	0	1
**ERK Signaling Main Pathway**	0.01302759	0.00752665	0.0175347	0.049534613
**Erythropoeitin Main Pathway**	0.00772405	0.00259649	0.0164176	0.049534613
**Estrogen Main Pathway**	0.01288068	0.00778455	0.0187924	0.049534613
**Fas Signaling Pathway (Negative)**	0	0	0	1
**Fas Signaling Pathway (Positive)**	0	0	0	1
**FLT3 Main Pathway**	0	0	0	1
**Glucocorticoid Receptor Main Pathway**	0.02242316	0.01848836	0.0412283	0.049534613
**Glucocorticoid Receptor Pathway (Cell cycle arrest)**	-0.7488383	-0.4938857	-0.276227	0.049534613
**Glucocorticoid Receptor Pathway (Cell cycle progression)**	0	0	0	1
**Glucocorticoid Receptor Pathway (Gene expression)**	0	0	0	1
**Glucocorticoid Receptor Pathway (Inflammatory cytokines)**	0.04222638	0.03424232	0.0659327	0.049534613
**Glucocorticoid Receptor Pathway (SMAD signaling)**	0	0	0	1
**GPCR Main Pathway**	0.0136611	0.01289919	0.0183866	0.049534613
**GPCR Pathway (Gene expression)**	0.04140874	0.03133855	0.0664255	0.049534613
**Growth Hormone Main Pathway**	-0.0144817	-0.0248131	-0.0280019	0.049534613
**Growth Hormone Pathway (Cell survival)**	0	0	0	1
**Growth Hormone Pathway (Gene expression)**	0	0	0	1
**Growth Hormone Pathway (Glucose uptake)**	-0.0124394	-0.0213138	-0.0240529	0.049534613
**Growth Hormone Pathway (Protein synthesis)**	0	0	0	1
**GSK3 Main Pathway**	0.00299823	0.00246148	0.0018969	0.049534613
**GSK3 Pathway (Degradation)**	0	0	0	1
**GSK3 Pathway (Gene expression)**	0	0	0	1
**GSK3 Pathway (Translation)**	0	0	0	1
**G-protein Pathway (Ras family GTPases)**	0	0	0	1
**Hedgehog Main Pathway**	0	0	0	1
**Hedgehog Pathway (Repression of Hh, BMP)**	0.03471176	0.05192009	0.0696135	0.049534613
**Hedgehog Pathway (Activation of BMP, Ptc, WNT)**	0	0	0	1
**HGF Main Pathway**	0.02010848	0.01405902	0.044027	0.049534613
**HGF Pathway (Anoikis)**	0.05831458	0.04077116	0.1276784	0.049534613
**HGF Pathway (Cell adhesion, cell mirgation)**	0	0	0	1
**HGF Pathway (Cell cycle progression)**	0.19438195	0.13590388	0.4255948	0.049534613
**HGF Pathway (Cell polarity, cell motility)**	0	0	0	1
**HGF Pathway (Cell scattering)**	0	0	0	1
**HGF Pathway (Cell survival)**	0	0	0	1
**HGF Pathway (IP3 pathway)**	0	0	0	1
**HGF Pathway (PKC pathway)**	0	0	0	1
**HIF1-Alpha Main Pathway**	0	0	0	1
**HIF1Alpha Pathway (Gene expression)**	0	0	0	1
**HIF1Alpha Pathway (HIF1alpha degradation)**	0	0	0	1
**HIF1Alpha Pathway (NOS pathway)**	0	0	0	1
**HIF1Alpha Pathway (p53 Hypoxia pathway)**	0	0	0	1
**HIF1Alpha Pathway (Pyruvate)**	0	0	0	1
**HIF1Alpha Pathway (VEGF pathway)**	0	0	0	1

**Hypoxia pathway EMT 1**	0	0	0	1
**Hypoxia pathway EMT 2**	0	0	0	1
**Hypoxia pathway EMT 3**	0	0	0	1
**Hypoxia pathway EMT 4**	0	0	0	1
**IGF1R Main Pathway**	0.0171324	0.01660555	0.0171123	0.049534613
**IGF1R Signaling Pathway (Cell survival)**	0	0	0	1
**IGF1R Signaling Pathway (Glucose uptake)**	0	0	0	1
**IGF1R Signaling Pathway (Glycogen synthesis)**	0	0	0	1
**IGF1R Signaling Pathway (IKB degradation)**	0.07109045	0.06052576	0.0457719	0.049534613
**IGF1R Signaling Pathway (Protein synthesis)**	0	0	0	1
**ILK Main Pathway**	0.01643962	0.01416557	0.0209413	0.049534613
**ILK Pathway (Apoptosis)**	-0.0099375	-0.0077984	-0.0140781	0.049534613
**ILK Pathway (Cell adhesion, cell motility, opsonization)**	-0.0114601	-0.0089933	-0.0162352	0.049534613
**ILK Pathway (Cell cycle proliferation)**	-0.0102975	-0.0080809	-0.0145882	0.049534613
**ILK Pathway (Cell migration, retraction)**	-0.0112782	-0.0088505	-0.0159775	0.049534613
**ILK Pathway (Cell motility)**	-0.0075992	-0.0059634	-0.0107656	0.049534613
**ILK Pathway (Cytoskeletal reorganization)**	-0.0028766	0.00334211	-0.0045044	0.51269076
**ILK Pathway (G2-phase arrest)**	-0.0102975	-0.0080809	-0.0145882	0.049534613
**ILK Pathway (Induced cell proliferation)**	0.05030293	0.02506318	0.0639677	0.049534613
**ILK Pathway (Regulation of intermediate filaments)**	-0.0113685	-0.0089213	-0.0161053	0.049534613
**ILK Pathway (Regulation of junction assembly of desmosomes)**	-0.0113685	-0.0089213	-0.0161053	0.049534613
**ILK Pathway (Wound healing)**	-0.0113685	-0.0089213	-0.0161053	0.049534613
**IL-10 Main Pathway**	0.06412176	0.02464008	0.0778665	0.049534613
**IL-10 Pathway (Gene expression)**	0.0666473	0.0567429	0.0429111	0.049534613
**IL-10 Pathway (Stability determination)**	0	0	0	1
**IL-10 Pathway (Translational modulation)**	-0.0970276	-0.1662479	-0.1876126	0.049534613
**IL-2 Main Pathway**	0.02652038	0.00654049	0.0250987	0.126630458
**IL-2 Pathway (Actin reorganization)**	0.07463665	0.12788299	0.1443174	0.049534613
**IL-2 Pathway (Apoptosis)**	0	0	0	1
**IL-2 Pathway (Apoptosis inhibition)**	0	0	0	1
**IL-2 Pathway (Protein synthesis)**	0.08820695	0.15113444	0.1705569	0.049534613
**IL-6 Main Pathway**	0.02947368	0.01524629	0.0282143	0.049534613
**Integrin SIgnaling Main Pathway**	0.0087927	0.00413794	0.017293	0.049534613
**Integrin SIgnaling Pathway (Cell survival)**	0	0	0	1
**Integrin SIgnaling Pathway (Cytoskeleton contraction integrin modulation cell invasion and migration)**	0.0472821	0.0330577	0.1035231	0.049534613
**Integrin SIgnaling Pathway (Focal adhesion and stress fibers)**	0	0	0	1
**Integrin SIgnaling Pathway (Translocation to the nucleus)**	0	0	0	1
**Interactions Report**	0	0	0	1
**Interferon Main Pathway**	0.02513993	0.02436684	0.0251104	0.049534613
**Interferon Pathway (Gene expression)**	0	0	0	1
**Interferon Pathway (Transcription)**	0	0	0	1
**Interferon Pathway (Translation)**	0	0	0	1
**IP3 Main Pathway**	0.00900189	0.00313701	0.010614	0.049534613
**IP3 Pathway (Gene expression)**	0	0	0	1
**JAK mStat Main Pathway**	0.04832145	0.02940443	0.0840417	0.049534613
**JAK mStat Pathway (Akt pathway)**	0	0	0	1
**JAK mStat Pathway (JAK degradation)**	0	0	0	1
**JNK Main Pathway**	-0.0038477	-0.0042006	-0.0062217	0.049534613
**JNK Pathway (Apoptosis, Inflammation, Tumorigenesis, Cell Migration)**	0	0	0	1
**JNK Pathway (Insulin signaling)**	-0.201839	-0.1722508	-0.2312606	0.049534613

**MAPK Family Main Pathway**	0.01089325	0.00950548	0.013233	0.049534613
**MAPK Family Pathway (Chromatin Remodeling)**	0	0	0	1
**MAPK Family Pathway (Cytoskeleton)**	0	0	0	1
**MAPK Family Pathway (Gene Expression)**	0.02734248	0.02327914	0.0176046	0.049534613
**MAPK Family Pathway (IKBs Degradation)**	0.08202744	0.06983742	0.0528137	0.049534613
**MAPK Family Pathway (Translation)**	0	0	0	1
**MAPK Signaling Main Pathway**	0.01707853	0.01178464	0.0273834	0.049534613
**MAPK Signaling Pathway (Cell Survival, Inflammation, Apoptosis, Osmoregulation)**	0	0	0	1
**MAPK Signaling Pathway (Gene Expression)**	0.02107756	0.01473657	0.0461488	0.049534613
**DDR pathway (MMR)**	0	0	0	1
**Mismatch Repair Main Pathway**	0	0	0	1
**Mitochondrial Apopotosis Main Pathway**	-0.0100454	-0.0085616	-0.0130709	0.049534613
**Mitochondrial Apopotosis Pathway (Apoptosis)**	0	0	0	1
**Mitochondrial Apopotosis Pathway (Depolarization)**	0	0	0	1
**Mitochondrial Apopotosis Pathway (DNA fragmentation)**	0	0	0	1
**Mitochondrial Apopotosis Pathway (Gene expression)**	0	0	0	1
**mTOR Main Pathway**	0.00840453	0.01011649	0.0083071	0.049534613
**mTOR Pathway (Actin organization)**	0.03712983	0.05931262	0.0502882	0.049534613
**mTOR Pathway (Akt signaling)**	0	0	0	1
**mTOR Pathway (Scanning)**	0	0	0	1
**mTOR Pathway (Translation on)**	0	0	0	1
**mTOR Pathway (VEGF pathway)**	0	0	0	1
**NGF (Negative) Main Pathway**	0	0	0	1
**NGF (Negative) Pathway (Apoptosis)**	0	0	0	1
**NGF (Positive) Main Pathway**	0.03821535	0.02427087	0.0392986	0.049534613
**NHEJ mechanisms of DSBs repair effect**	0	0	0	1
**Notch Main Pathway**	0	0	0	1
**DDR Pathway (NER)**	0	0	0	1
**p38 (Negative) Main Signaling Pathway**	0.01737052	0.01348705	0.023943	0.049534613
**p38 (Positive) Main Signaling Pathway**	0.01733521	0.01345963	0.0238943	0.049534613
**p53 Signaling (Negative) Main Pathway**	0.02367253	0.03060733	0.0881163	0.049534613
**p53 Signaling (Negative) Pathway (p53 Degradation)**	0	0	0	1
**PAK Main Pathway**	0.00940196	0.00830729	0.0155968	0.049534613
**PAK Pathway (Actin Cytoskeleton)**	0.0147936	0.01307118	0.024541	0.049534613
**PAK Pathway (Myosin Activation)**	0	0	0	1
**PPAR Main Pathway**	0.00704213	0.00600192	0.0091631	0.049534613
**PTEN Main Pathway**	0	0	0	1
**RANK Signaling in Osteoclast Main Pathway**	0.03857252	0.02919207	0.0618758	0.049534613
**RANK Signaling in Osteoclast Pathway (IKBs Degradation)**	0.05331784	0.04539432	0.0343289	0.049534613
**RAS Main Pathway**	0.00666701	0.00143889	0.010125	0.049534613
**Telomere Main Pathway**	0.05810856	0.02895229	0.0738937	0.049534613
**RNA Polymerase II Complex Pathway**	0	0	0	1
**Cell Cycle Pathway (SCC during S-phase)**	0	0	0	1
**SMAD (Negative) Main Pathway**	0.0375439	0.0282891	0.0542274	0.049534613
**SMAD (Negative) Pathway (Degradation)**	0	0	0	1
**SMAD (Positive) Main Pathway**	0.0375439	0.0282891	0.0542274	0.049534613
**SMAD (Positive) Pathway (Degradation)**	0	0	0	1
**Cell Cycle Pathway (Metaphase-Anaphase)**	0	0	0	1
**Cell Cycle Pathway (Origin of S-phase)**	0	0	0	1
**STAT3 Main Pathway**	0.0176139	0.0110135	0.0278497	0.049534613

**TGF beta Main Pathway**	0.02015169	0.0154186	0.0318126	0.049534613
**TGF beta Pathway (Epithelial mesehchymal transition)**	0	0	0	1
**TGF beta Pathway (Post-transcriptional G1 arrest)**	0	0	0	1
**TGF beta Pathway (SnON degradation)**	0.17494375	0.12231349	0.3830353	0.049534613
**TGF beta Pathway (Tumorigenesis)**	0.24991965	0.17473356	0.5471933	0.049534613
**TGF beta Pathway (Tumor suppression)**	0.24991965	0.17473356	0.5471933	0.049534613
**TNF (Negative) Main Pathway**	0	0	0	1
**TNF (Negative) Pathway (Apoptosis)**	0	0	0	1
**TNF (Positive) Main Pathway**	0.03570281	0.02496194	0.0781705	0.049534613
**TNF (Positive) Pathway (Gene expression, Cell survival)**	0.09385981	0.07103404	0.1505644	0.049534613
**TNF (Positive) Pathway (IKBs degradation)**	0.05924204	0.05043814	0.0381432	0.049534613
**TRAF (Negative) Main Pathway**	0	0	0	1
**TRAF (Negative) Pathway (IKBs Degradation)**	0.05924204	0.05043814	0.0381432	0.049534613
**TRAF (Positive) Main Pathway**	0.03527034	0.02793243	0.0792755	0.049534613
**TRAF (Positive) Pathway (IKBs Degradation)**	0.05924204	0.05043814	0.0381432	0.049534613
**Transcription of mRNA Pathway**	0	0	0	1
**Cell Cycle Pathway (End of S-phase)**	0	0	0	1
**Translation Regulation of EIF4F activity**	0	0	0	1
**Ubiquitin Proteasome Main Pathway**	0	0	0	1
**Ubiquitin Proteasome Pathway (Degraded Protein)**	0	0	0	1
**VEGF Main Pathway**	0	0	0	1
**VEGF Pathway (Actin Reorganization)**	0	0	0	1
**VEGF Pathway (Nitric Oxide Production)**	0	0	0	1
**Wnt Main Pathway**	0.02447694	0.0130864	0.0294049	0.049534613
**Wnt Pathway (Ctnn-b Degradation)**	0	0	0	1
**IL-6 Pathway (IKBs degradation)**	0.08886307	0.0756572	0.0572148	0.049534613

Borger et al
Supplemental Data

Pathway	M_86_32h_1	M_86_32h_2	M_86_32h_3	p-
AHR Main Pathway	0	0	0	
AHR Pathway (AHR Degradation)	0	0	0	
AHR Pathway (Cath-D Expression)	0	0	0	
AHR Pathway (C-MycExpression)	0	0	0	
AHR Pathway (PS2 Gene Expression)	0	0	0	
AKT Main Pathway	0.060424	0.071181	0.061002	
AKT Pathway (Aggregation & Neurodegeneration)	0.027091	0.030477	0.032081	
AKT Pathway (Apoptosis Inhibition)	0.026029	0.029282	0.030823	
AKT Pathway (Blocks Apoptosis)	0	0	0	
AKT Pathway (Cardiovascular Homeostasis)	0.029499	0.033186	0.034932	
AKT Pathway (Caspase Cascade)	0	0	0	
AKT Pathway (Cell Cycle)	0	0	0	
AKT Pathway (Cell Cycle Progression)	-0.12165	-0.09055	-0.11137	
AKT Pathway (Cell Survival)	0	0	0	
AKT Pathway (Death Genes)	0	0	0	
AKT Pathway (Elevation of Glucose Import)	0	0	0	
AKT Pathway (ERK Pathway)	0	0	0	
AKT Pathway (Genetic Stability)	0	0	0	
AKT Pathway (Glucose Uptake)	0.03017	0.033941	0.035726	
AKT Pathway (Glycogen Synthesis)	0	0	0	
AKT Pathway (Insulin Stimulated Mitogenesis)	0.029499	0.033186	0.034932	
AKT Pathway (JNK Pathway)	0	0	0	
AKT Pathway (Neuroprotection)	0.029499	0.033186	0.034932	
AKT Pathway (NF-kB Pathway)	0.027655	0.031112	0.032749	
AKT Pathway (p53 Degradation)	0	0	0	
AKT Pathway (p73 Mediated Apoptosis)	0	0	0	
AKT Pathway (Protein Synthesis)	0	0	0	
AKT Pathway (Regeneration of Cyclic Nucleotide)	0	0	0	
AKT Pathway (Respiratory Burst)	0.029499	0.033186	0.034932	
AKT Pathway (Survival Genes)	0	0	0	
AKT Pathway (Synaptic Signaling)	0	0	0	
AKT Pathway (Translation)	0	0	0	
Androgen Receptor Pathway	0.08491	0.09619	0.090212	
Androgen Receptor Pathway (Apoptosis)	0.046287	0.104754	0.08255	
Androgen Receptor Pathway (Degradation)	0.045988	0.069977	0.054133	
Androgen Receptor Pathway (Cell Survival & Cell Growth)	0.059659	0.057729	0.048361	
Androgen Receptor Pathway (Gonadotropin Regulation)	-0.03578	-0.01244	-0.02965	
Androgen Receptor Pathway (Histone Modification)	-0.03578	-0.01244	-0.02965	
Androgen Receptor Pathway (Prostate Differentiation & Development)	-0.03578	-0.01244	-0.02965	
Androgen Receptor Pathway (Sexual Differentiation & Sexual Maturation at Puberty)	-0.03578	-0.01244	-0.02965	
ATM Main Pathway	0.120154	0.100467	0.092802	
ATM Pathway (Apoptosis)	0	0	0	
ATM Pathway (Apoptosis, Senescense)	0.102388	0.195777	0.114126	
ATM Pathway (Cell Cicle Checkpoint Control)	0.122866	0.234933	0.136952	
ATM Pathway (Cell Survival)	0.122831	0.133878	0.104691	
ATM Pathway (Checkpoint Activation)	0.122866	0.234933	0.136952	
ATM Pathway (DNA Repair)	0.061433	0.117466	0.068476	
ATM Pathway (G2_M Checkpoint Arrest)	0.345887	0.246709	0.253758	
ATM Pathway (G2 Mitosis Progression)	0	0	0	

**ATM Pathway (MDMX Ubiquitination, Degradation)**	0	0	0
**ATM Pathway (NF-kB Pathway)**	0.122866	0.234933	0.136952
**ATM Pathway (Synaptic Vesicle Transport)**	0.122866	0.234933	0.136952
**ATM Pathway (S-Phase Arrest)**	0.122866	0.234933	0.136952
**ATM Pathway (S-Phase Progression)**	0	0	0
**DDR Pathway (BRCA1-induced responses)**	0.152817	0.091193	0.148008
**BRCA1 Main Pathway**	-0.04919	-0.02	-0.02652
**cAMP Main Pathway**	0.082057	0.085698	0.084975
**cAMP Pathway (Axonal Growth)**	-0.10942	-0.04915	-0.0668
**cAMP Pathway (Cardiovascular Homeostasis)**	-0.23448	-0.10531	-0.14315
**cAMP Pathway (Cell Growth)**	0	0	0
**cAMP Pathway (Cell Proliferation)**	-0.25251	-0.11341	-0.15416
**cAMP Pathway (Cell Survival)**	-0.0248	0.031946	0.010576
**cAMP Pathway (Cell Survival, Chemotaxis)**	0	0	0
**cAMP Pathway (Cytokine Production)**	0.175447	0.119072	0.126515
**cAMP Pathway (Degradation of Cell Cycle Regulators)**	-0.10589	-0.04756	-0.06465
**cAMP Pathway (Endothelial Cell Regulation)**	0.157386	0.190101	0.231713
**cAMP Pathway (Glycogen Synthesis)**	-0.29842	-0.13403	-0.18219
**cAMP Pathway (Glycolysis)**	-0.08291	0.03422	-0.05398
**cAMP Pathway (Metabolic Energy)**	0	0	0
**cAMP Pathway (Myocardial Contraction)**	0.009528	0.016436	0.007179
**cAMP Pathway (Oncogenesis)**	-0.23448	-0.10531	-0.14315
**cAMP Pathway (Protein Retention)**	-0.00042	0.175815	0.090615
**cAMP Pathway (Regulation of Cytoskeleton)**	-0.20517	-0.09215	-0.12526
**Caspase Cascade Main**	-0.08774	-0.09824	-0.09366
**Caspase Cascade (Activated Tissue Transglutaminase)**	0.009822	0.010252	0.003712
**Caspase Cascade (Apoptosis)**	0	0	0
**Caspase Cascade (Cell Survival)**	-0.04339	-0.09821	-0.07739
**Caspase Cascade (ICAD Degradation)**	0	0	0
**CD40 Main Pathway**	0.049554	0.028879	0.03687
**CD40 Pathway (Cell Survival)**	0	0	0
**CD40 Pathway (Gene Expression)**	0.11652	0.058092	0.089044
**CD40 Pathway (IKBs Degradation)**	0.08225	0.041006	0.062854
**Cellular Anti Apoptosis Main Pathway**	0.092676	0.103181	0.098285
**Cellular Anti Apoptosis Pathway (Apoptosis)**	0.041012	0.027319	0.016389
**Cellular Anti Apoptosis Pathway (Depolarization)**	0	0	0
**Chemokine Main Pathway**	0.057938	0.043987	0.051538
**Chemokine Pathway (Cell Activation)**	0.042349	0.028741	0.030538
**Chemokine Pathway (Gene Expression, Apoptosis)**	0.053397	0.036239	0.038505
**Chemokine Pathway (Internalization, Degradation, Recycling)**	0	0	0
**Chromatin Main Pathway**	0	0	0
**Chromatin Pathway (Octamer Sliding)**	0	0	0
**Chromatin Pathway (Octamer Transfer)**	0	0	0
**Circadian Main Pathway**	0	0	0
**CREB Main Pathway**	0.092702	0.103743	0.097046
**CREB Pathway (Gene Expression Pathway)**	0.042832	0.041446	0.03472
**Cytokine Main Pathway**	0	0	0
**DDR pathway Apoptosis**	0	0	0
**DDR Main pathway**	0	0	0
**DNA Repair Mechanisms Pathway**	0.001557	0.008155	0.009464
**EGFR Main Pathway**	0.054547	0.040641	0.052893

**ErbB Family Main Pathway**	0.03893	0.044505	0.044717
**ErbB Family Pathway (Anti-Apoptosis)**	0	0	0
**ERK Signaling Main Pathway**	0.121017	0.128844	0.122809
**Erythropoeitin Main Pathway**	-0.00737	-0.00124	-0.00686
**Estrogen Main Pathway**	0.094038	0.109419	0.094906
**Fas Signaling Pathway (Negative)**	0.050066	0.056246	0.038913
**Fas Signaling Pathway (Positive)**	0	0	0
**FLT3 Main Pathway**	0.056749	0.063925	0.064677
**Glucocorticoid Receptor Main Pathway**	0.071938	0.072846	0.067562
**Glucocorticoid Receptor Pathway (Cell cycle arrest)**	1.081263	0.927807	0.98317
**Glucocorticoid Receptor Pathway (Cell cycle progression)**	0	0	0
**Glucocorticoid Receptor Pathway (Gene expression)**	0.032084	0.031087	0.027553
**Glucocorticoid Receptor Pathway (Inflammatory cytokines)**	0.072744	0.070875	0.071041
**Glucocorticoid Receptor Pathway (SMAD signaling)**	0.098788	0.127857	0.07354
**GPCR Main Pathway**	0.095239	0.10642	0.095506
**GPCR Pathway (Gene expression)**	0.063189	0.046279	0.04865
**Growth Hormone Main Pathway**	-0.00976	-0.00839	-0.01434
**Growth Hormone Pathway (Cell survival)**	0	0	0
**Growth Hormone Pathway (Gene expression)**	-0.01521	-0.01307	-0.02234
**Growth Hormone Pathway (Glucose uptake)**	-0.00838	-0.00721	-0.01232
**Growth Hormone Pathway (Protein synthesis)**	0	0	0
**GSK3 Main Pathway**	0.083098	0.111157	0.088229
**GSK3 Pathway (Degradation)**	0	0	0
**GSK3 Pathway (Gene expression)**	0.044417	-0.01452	0.020726
**GSK3 Pathway (Translation)**	0	0	0
**G-protein Pathway (Ras family GTPases)**	0	0	0
**Hedgehog Main Pathway**	-0.19376	-0.07686	-0.15323
**Hedgehog Pathway (Repression of Hh, BMP)**	0.387358	0.478421	0.540323
**Hedgehog Pathway (Activation of BMP, Ptc, WNT)**	0	0	0
**HGF Main Pathway**	0.082662	0.076748	0.056066
**HGF Pathway (Anoikis)**	0.19946	0.185189	0.127918
**HGF Pathway (Cell adhesion, cell mirgation)**	0.037744	0.035044	0.032507
**HGF Pathway (Cell cycle progression)**	0.33418	0.279023	0.209415
**HGF Pathway (Cell polarity, cell motility)**	0	0	0
**HGF Pathway (Cell scattering)**	0.1294	0.132368	0.084904
**HGF Pathway (Cell survival)**	0.1294	0.132368	0.084904
**HGF Pathway (IP3 pathway)**	0.14881	0.152223	0.09764
**HGF Pathway (PKC pathway)**	0	0	0
**HIF1-Alpha Main Pathway**	0	0	0
**HIF1Alpha Pathway (Gene expression)**	0	0	0
**HIF1Alpha Pathway (HIF1alpha degradation)**	0	0	0
**HIF1Alpha Pathway (NOS pathway)**	0	0	0
**HIF1Alpha Pathway (p53 Hypoxia pathway)**	0	0	0
**HIF1Alpha Pathway (Pyruvate)**	0	0	0
**HIF1Alpha Pathway (VEGF pathway)**	0	0	0
**Hypoxia pathway EMT 1**	1.509285	1.336039	1.719183
**Hypoxia pathway EMT 2**	1.509285	1.336039	1.719183
**Hypoxia pathway EMT 3**	1.509285	1.336039	1.719183
**Hypoxia pathway EMT 4**	1.509285	1.336039	1.719183
**IGF1R Main Pathway**	0.009621	0.009586	0.011457
**IGF1R Signaling Pathway (Cell survival)**	-0.03673	-0.0264	-0.03259

**IGF1R Signaling Pathway (Glucose uptake)**	0	0	0
**IGF1R Signaling Pathway (Glycogen synthesis)**	0	0	0
**IGF1R Signaling Pathway (IKB degradation)**	0.093216	0.046473	0.071235
**IGF1R Signaling Pathway (Protein synthesis)**	0.112141	0	0.126904
**ILK Main Pathway**	0.154363	0.18115	0.178657
**ILK Pathway (Apoptosis)**	0.248492	0.266625	0.280078
**ILK Pathway (Cell adhesion, cell motility, opsonization)**	0.272465	0.294643	0.305122
**ILK Pathway (Cell cycle proliferation)**	0.244824	0.264752	0.274167
**ILK Pathway (Cell migration, retraction)**	0.268141	0.289966	0.300278
**ILK Pathway (Cell motility)**	0.229961	0.247452	0.269804
**ILK Pathway (Cytoskeletal reorganization)**	0.283726	0.298371	0.316153
**ILK Pathway (G2-phase arrest)**	0.244824	0.264752	0.274167
**ILK Pathway (Induced cell proliferation)**	-0.01925	-0.02662	-0.01606
**ILK Pathway (Regulation of intermediate filaments)**	0.270286	0.292286	0.302681
**ILK Pathway (Regulation of junction assembly of desmosomes)**	0.270286	0.292286	0.302681
**ILK Pathway (Wound healing)**	0.282744	0.308054	0.319175
**IL-10 Main Pathway**	0.05902	0.034396	0.043913
**IL-10 Pathway (Gene expression)**	0.164148	0.095663	0.122133
**IL-10 Pathway (Stability determination)**	0	0	0
**IL-10 Pathway (Translational modulation)**	0.122813	0.08335	0.08856
**IL-2 Main Pathway**	0.001408	0.018905	0.015148
**IL-2 Pathway (Actin reorganization)**	0.055123	0.226592	0.239233
**IL-2 Pathway (Apoptosis)**	0	0	0
**IL-2 Pathway (Apoptosis inhibition)**	0	0	0
**IL-2 Pathway (Protein synthesis)**	0	0	0
**IL-6 Main Pathway**	0.116788	0.096952	0.110098
**Integrin SIgnaling Main Pathway**	0.168492	0.177336	0.182812
**Integrin SIgnaling Pathway (Cell survival)**	0.01421	0.013193	0.012238
**Integrin SIgnaling Pathway (Cytoskeleton contraction integrin modulation cell invasion and migration)**	0	0	0
**Integrin SIgnaling Pathway (Focal adhesion and stress fibers)**	0	0	0
**Integrin SIgnaling Pathway (Translocation to the nucleus)**	0.275278	0.298325	0.245147
**Interactions Report**	1.358357	1.202435	1.547265
**Interferon Main Pathway**	0.025538	0.00906	0.02342
**Interferon Pathway (Gene expression)**	0.061407	0.041675	0.04428
**Interferon Pathway (Transcription)**	0	0	0
**Interferon Pathway (Translation)**	0.186901	0	0.211507
**IP3 Main Pathway**	0.026184	0.031556	0.026505
**IP3 Pathway (Gene expression)**	0	0	0
**JAK mStat Main Pathway**	0.041421	0.03046	0.039761
**JAK mStat Pathway (Akt pathway)**	0.024713	0.057966	0.014309
**JAK mStat Pathway (JAK degradation)**	0	0	0
**JNK Main Pathway**	0.084376	0.102984	0.087283
**JNK Pathway (Apoptosis, Inflammation, Tumorigenesis, Cell Migration)**	0.066154	0.071333	0.073173
**JNK Pathway (Insulin signaling)**	0	0	0
**MAPK Family Main Pathway**	0.044061	0.04307	0.042304
**MAPK Family Pathway (Chromatin Remodeling)**	0.042349	0.028741	0.030538
**MAPK Family Pathway (Cytoskeleton)**	0.081875	0.055567	0.05904
**MAPK Family Pathway (Gene Expression)**	0.067343	0.039246	0.050106
**MAPK Family Pathway (IKBs Degradation)**	0.107557	0.053623	0.082194
**MAPK Family Pathway (Translation)**	0	0	0

**MAPK Signaling Main Pathway**	0.113417	0.135182	0.123783
**MAPK Signaling Pathway (Cell Survival, Inflammation, Apoptosis, Osmoregulation)**	-0.02015	-0.02787	-0.01681
**MAPK Signaling Pathway (Gene Expression)**	0.023928	0.031585	0.035839
**DDR pathway (MMR)**	0	0	0
**Mismatch Repair Main Pathway**	0	0	0
**Mitochondrial Apopotosis Main Pathway**	-0.09588	-0.12088	-0.12038
**Mitochondrial Apopotosis Pathway (Apoptosis)**	0.109365	0.07285	0.043703
**Mitochondrial Apopotosis Pathway (Depolarization)**	0	0	0
**Mitochondrial Apopotosis Pathway (DNA fragmentation)**	-0.11572	-0.26189	-0.20638
**Mitochondrial Apopotosis Pathway (Gene expression)**	0	0	0
**mTOR Main Pathway**	0.095974	0.104567	0.106931
**mTOR Pathway (Actin organization)**	0.047897	0.049332	0.04629
**mTOR Pathway (Akt signaling)**	0	0	0
**mTOR Pathway (Scanning)**	0.0623	0	0.070502
**mTOR Pathway (Translation on)**	0.03115	0	0.035251
**mTOR Pathway (VEGF pathway)**	0	0	0
**NGF (Negative) Main Pathway**	0	0	0
**NGF (Negative) Pathway (Apoptosis)**	0	0	0
**NGF (Positive) Main Pathway**	0.046316	0.036655	0.046427
**NHEJ mechanisms of DSBs repair effect**	0.036137	0.069098	0.04028
**Notch Main Pathway**	-0.03695	-0.04311	-0.03275
**DDR Pathway (NER)**	0	0	0
**p38 (Negative) Main Signaling Pathway**	0.076694	0.084565	0.076289
**p38 (Positive) Main Signaling Pathway**	0.076538	0.084393	0.076134
**p53 Signaling (Negative) Main Pathway**	0.148255	0.076654	0.106024
**p53 Signaling (Negative) Pathway (p53 Degradation)**	0.02038	0.022439	0.020923
**PAK Main Pathway**	0.122325	0.12987	0.13986
**PAK Pathway (Actin Cytoskeleton)**	0.108031	0.108341	0.116224
**PAK Pathway (Myosin Activation)**	0.083898	0.098266	0.123438
**PPAR Main Pathway**	0.092556	0.100331	0.090991
**PTEN Main Pathway**	-0.02979	-0.02988	-0.03499
**RANK Signaling in Osteoclast Main Pathway**	0.124038	0.083654	0.122852
**RANK Signaling in Osteoclast Pathway (IKBs Degradation)**	0.069912	0.034855	0.053426
**RAS Main Pathway**	0.018408	0.037666	0.016147
**Telomere Main Pathway**	-0.02118	-0.04051	-0.02361
**RNA Polymerase II Complex Pathway**	0.026897	0.022147	0.022554
**Cell Cycle Pathway (SCC during S-phase)**	0	0	0
**SMAD (Negative) Main Pathway**	0.117697	0.120776	0.137763
**SMAD (Negative) Pathway (Degradation)**	0	0	0
**SMAD (Positive) Main Pathway**	0.117697	0.120776	0.137763
**SMAD (Positive) Pathway (Degradation)**	0	0	0
**Cell Cycle Pathway (Metaphase-Anaphase)**	0.102339	0.129445	0.132727
**Cell Cycle Pathway (Origin of S-phase)**	0.021278	0.079555	0.04297
**STAT3 Main Pathway**	0.107979	0.088025	0.092858
**TGF beta Main Pathway**	0.086893	0.100158	0.095158
**TGF beta Pathway (Epithelial mesehchymal transition)**	0.054593	0.070658	0.04064
**TGF beta Pathway (Post-transcriptional G1 arrest)**	0.041491	0.0537	0.030887
**TGF beta Pathway (SnON degradation)**	0	0	0
**TGF beta Pathway (Tumorigenesis)**	0	0	0
**TGF beta Pathway (Tumor suppression)**	0	0	0

**TNF (Negative) Main Pathway**	0.069072	0.046011	0.027602
**TNF (Negative) Pathway (Apoptosis)**	0.077198	0.051424	0.030849
**TNF (Positive) Main Pathway**	0.035356	0.049911	0.038263
**TNF (Positive) Pathway (Gene expression, Cell survival)**	0.063418	0.076974	0.068593
**TNF (Positive) Pathway (IKBs degradation)**	0.07768	0.038728	0.059362
**TRAF (Negative) Main Pathway**	0	0	0
**TRAF (Negative) Pathway (IKBs Degradation)**	0.07768	0.038728	0.059362
**TRAF (Positive) Main Pathway**	0.041321	0.017141	0.030511
**TRAF (Positive) Pathway (IKBs Degradation)**	0.07768	0.038728	0.059362
**Transcription of mRNA Pathway**	0.03138	0.025838	0.026313
**Cell Cycle Pathway (End of S-phase)**	0	0	0
**Translation Regulation of EIF4F activity**	0.021169	0.027398	0.015758
**Ubiquitin Proteasome Main Pathway**	0.001187	0.007084	0.005111
**Ubiquitin Proteasome Pathway (Degraded Protein)**	-0.01328	-0.01936	-0.01728
**VEGF Main Pathway**	0.15227	0.116694	0.16149
**VEGF Pathway (Actin Reorganization)**	0.299781	0.229741	0.317933
**VEGF Pathway (Nitric Oxide Production)**	0	0	0
**Wnt Main Pathway**	0.04592	0.052567	0.057212
**Wnt Pathway (Ctnn-b Degradation)**	0	0	0
**IL-6 Pathway (IKBs degradation)**	0.11652	0.058092	0.089044

**Pathway**	**M_68_4h_1**	**M_68_4h_2**	**M_68_4h_3**
**AHR Main Pathway**	0	0	0
**AHR Pathway (AHR Degradation)**	0	0	0
**AHR Pathway (Cath-D Expression)**	0	0	0
**AHR Pathway (C-MycExpression)**	0	0	0
**AHR Pathway (PS2 Gene Expression)**	0	0	0
**AKT Main Pathway**	0.020815	0.023276	0.02706
**AKT Pathway (Aggregation & Neurodegeneration)**	-0.02665	-0.0164	-0.0122
**AKT Pathway (Apoptosis Inhibition)**	-0.0256	-0.01576	-0.01172
**AKT Pathway (Blocks Apoptosis)**	0	0	0
**AKT Pathway (Cardiovascular Homeostasis)**	-0.02902	-0.01786	-0.01328
**AKT Pathway (Caspase Cascade)**	0	0	0
**AKT Pathway (Cell Cycle)**	0	0	0
**AKT Pathway (Cell Cycle Progression)**	0	0	0
**AKT Pathway (Cell Survival)**	0	0	0
**AKT Pathway (Death Genes)**	0	0	0
**AKT Pathway (Elevation of Glucose Import)**	0	0	0
**AKT Pathway (ERK Pathway)**	0	0	0
**AKT Pathway (Genetic Stability)**	0	0	0
**AKT Pathway (Glucose Uptake)**	-0.02968	-0.01826	-0.01358
**AKT Pathway (Glycogen Synthesis)**	0	0	0
**AKT Pathway (Insulin Stimulated Mitogenesis)**	-0.02902	-0.01786	-0.01328
**AKT Pathway (JNK Pathway)**	0	0	0
**AKT Pathway (Neuroprotection)**	-0.02902	-0.01786	-0.01328
**AKT Pathway (NF-kB Pathway)**	-0.0272	-0.01674	-0.01245
**AKT Pathway (p53 Degradation)**	0	0	0
**AKT Pathway (p73 Mediated Apoptosis)**	0	0	0
**AKT Pathway (Protein Synthesis)**	0	0	0

AKT Pathway (Regeneration of Cyclic Nucleotide)	0	0	0
AKT Pathway (Respiratory Burst)	-0.02902	-0.01786	-0.01328
AKT Pathway (Survival Genes)	0.04833	0.02739	0.052597
AKT Pathway (Synaptic Signaling)	0	0	0
AKT Pathway (Translation)	0	0	0
Androgen Receptor Pathway	0.01708	0.021868	0.027979
Androgen Receptor Pathway (Apoptosis)	0	0	0
Androgen Receptor Pathway (Degradation)	0.021474	0.03173	0.056072
Androgen Receptor Pathway (Cell Survival & Cell Growth)	0	0	0
Androgen Receptor Pathway (Gonadotropin Regulation)	0.069916	0.080618	0.092352
Androgen Receptor Pathway (Histone Modification)	0.069916	0.080618	0.092352
Androgen Receptor Pathway (Prostate Differentiation & Development)	0.069916	0.080618	0.092352
Androgen Receptor Pathway (Sexual Differentiation & Sexual Maturation at Puberty)	0.069916	0.080618	0.092352
ATM Main Pathway	0.066931	0.064665	0.077161
ATM Pathway (Apoptosis)	0	0	0
ATM Pathway (Apoptosis, Senescense)	0	0	0
ATM Pathway (Cell Cicle Checkpoint Control)	0	0	0
ATM Pathway (Cell Survival)	0.086994	0.049302	0.094675
ATM Pathway (Checkpoint Activation)	0	0	0
ATM Pathway (DNA Repair)	0	0	0
ATM Pathway (G2_M Checkpoint Arrest)	0.16735	0.209067	0.200585
ATM Pathway (G2 Mitosis Progression)	0	0	0
ATM Pathway (MDMX Ubiquitination, Degradation)	0	0	0
ATM Pathway (NF-kB Pathway)	0	0	0
ATM Pathway (Synaptic Vesicle Transport)	0	0	0
ATM Pathway (S-Phase Arrest)	0	0	0
ATM Pathway (S-Phase Progression)	0	0	0
DDR Pathway (BRCA1-induced responses)	-0.10153	-0.14218	-0.14119
BRCA1 Main Pathway	-0.01428	-0.01673	-0.01753
cAMP Main Pathway	0.008067	0.011234	0.013025
cAMP Pathway (Axonal Growth)	-0.05562	-0.04862	-0.04636
cAMP Pathway (Cardiovascular Homeostasis)	-0.11919	-0.1042	-0.09934
cAMP Pathway (Cell Growth)	0	0	0
cAMP Pathway (Cell Proliferation)	-0.12836	-0.11221	-0.10698
cAMP Pathway (Cell Survival)	-0.0298	-0.02605	-0.02484
cAMP Pathway (Cell Survival, Chemotaxis)	0	0	0
cAMP Pathway (Cytokine Production)	0	0	0
cAMP Pathway (Degradation of Cell Cycle Regulators)	-0.05383	-0.04706	-0.04486
cAMP Pathway (Endothelial Cell Regulation)	-0.0329	-0.04308	-0.04374
cAMP Pathway (Glycogen Synthesis)	-0.1517	-0.13261	-0.12644
cAMP Pathway (Glycolysis)	-0.07585	-0.06631	-0.06322
cAMP Pathway (Metabolic Energy)	0	0	0
cAMP Pathway (Myocardial Contraction)	-0.01005	-0.00879	-0.00838
cAMP Pathway (Oncogenesis)	-0.11919	-0.1042	-0.09934
cAMP Pathway (Protein Retention)	-0.10429	-0.09117	-0.08693
cAMP Pathway (Regulation of Cytoskeleton)	-0.10429	-0.09117	-0.08693
Caspase Cascade Main	-0.00943	-0.01401	-0.01567
Caspase Cascade (Activated Tissue Transglutaminase)	0.006538	0.005469	0.003575
Caspase Cascade (Apoptosis)	0	0	0
Caspase Cascade (Cell Survival)	-0.05956	-0.0677	-0.06681
Caspase Cascade (ICAD Degradation)	0	0	0

**CD40 Main Pathway**	0	0	0
**CD40 Pathway (Cell Survival)**	0	0	0
**CD40 Pathway (Gene Expression)**	0	0	0
**CD40 Pathway (IKBs Degradation)**	0	0	0
**Cellular Anti Apoptosis Main Pathway**	0.021195	0.028302	0.033658
**Cellular Anti Apoptosis Pathway (Apoptosis)**	0.049736	0.045182	0.044588
**Cellular Anti Apoptosis Pathway (Depolarization)**	0	0	0
**Chemokine Main Pathway**	0.042268	0.042301	0.043082
**Chemokine Pathway (Cell Activation)**	0	0	0
**Chemokine Pathway (Gene Expression, Apoptosis)**	0	0	0
**Chemokine Pathway (Internalization, Degradation, Recycling)**	-0.07605	-0.06234	-0.09533
**Chromatin Main Pathway**	0	0	0
**Chromatin Pathway (Octamer Sliding)**	0	0	0
**Chromatin Pathway (Octamer Transfer)**	0	0	0
**Circadian Main Pathway**	-0.16094	-0.18488	-0.20166
**CREB Main Pathway**	0.013349	0.016045	0.019792
**CREB Pathway (Gene Expression Pathway)**	0.000308	-0.00821	-0.00514
**Cytokine Main Pathway**	-0.02207	-0.02848	-0.03294
**DDR pathway Apoptosis**	0	0	0
**DDR Main pathway**	0	0	0
**DNA Repair Mechanisms Pathway**	-0.01405	-0.01756	-0.01684
**EGFR Main Pathway**	0.051604	0.05945	0.077336
**ErbB Family Main Pathway**	0.054947	0.057097	0.06051
**ErbB Family Pathway (Anti-Apoptosis)**	0	0	0
**ERK Signaling Main Pathway**	0.035811	0.039313	0.042743
**Erythropoeitin Main Pathway**	-0.008	-0.01399	-0.01407
**Estrogen Main Pathway**	0.019806	0.020866	0.02878
**Fas Signaling Pathway (Negative)**	0	0	0
**Fas Signaling Pathway (Positive)**	0	0	0
**FLT3 Main Pathway**	0.01919	0.010875	0.020884
**Glucocorticoid Receptor Main Pathway**	0.018241	0.015673	0.020325
**Glucocorticoid Receptor Pathway (Cell cycle arrest)**	0	0	0
**Glucocorticoid Receptor Pathway (Cell cycle progression)**	0.323654	0.336335	0.492746
**Glucocorticoid Receptor Pathway (Gene expression)**	0.016011	0.009074	0.017425
**Glucocorticoid Receptor Pathway (Inflammatory cytokines)**	0.017297	0.01714	0.017298
**Glucocorticoid Receptor Pathway (SMAD signaling)**	0	0	0
**GPCR Main Pathway**	0.02094	0.022435	0.028967
**GPCR Pathway (Gene expression)**	0.063159	0.063464	0.079067
**Growth Hormone Main Pathway**	-0.05017	-0.04703	-0.03481
**Growth Hormone Pathway (Cell survival)**	0	0	0
**Growth Hormone Pathway (Gene expression)**	0.057901	0.060533	0.070127
**Growth Hormone Pathway (Glucose uptake)**	-0.0431	-0.0404	-0.0299
**Growth Hormone Pathway (Protein synthesis)**	0	0	0
**GSK3 Main Pathway**	0.006023	0.009691	0.013838
**GSK3 Pathway (Degradation)**	0	0	0
**GSK3 Pathway (Gene expression)**	0	0	0
**GSK3 Pathway (Translation)**	0	0	0
**G-protein Pathway (Ras family GTPases)**	0.10936	0.12977	0.214078
**Hedgehog Main Pathway**	-0.06114	-0.07437	-0.04755
**Hedgehog Pathway (Repression of Hh, BMP)**	0.066251	0.065439	0.104282
**Hedgehog Pathway (Activation of BMP, Ptc, WNT)**	0	0	0

**HGF Main Pathway**	0.061001	0.062351	0.063259
**HGF Pathway (Anoikis)**	0.085467	0.091784	0.126406
**HGF Pathway (Cell adhesion, cell mirgation)**	0.085722	0.083468	0.053479
**HGF Pathway (Cell cycle progression)**	0.284889	0.305946	0.421354
**HGF Pathway (Cell polarity, cell motility)**	0	0	0
**HGF Pathway (Cell scattering)**	0	0	0
**HGF Pathway (Cell survival)**	0	0	0
**HGF Pathway (IP3 pathway)**	0	0	0
**HGF Pathway (PKC pathway)**	0	0	0
**HIF1-Alpha Main Pathway**	0.055292	0.051927	0.072521
**HIF1Alpha Pathway (Gene expression)**	0.141326	0.105881	0.134967
**HIF1Alpha Pathway (HIF1alpha degradation)**	0.067915	0.070724	0.050365
**HIF1Alpha Pathway (NOS pathway)**	0.163068	0.12217	0.155731
**HIF1Alpha Pathway (p53 Hypoxia pathway)**	0.058213	0.06062	0.04317
**HIF1Alpha Pathway (Pyruvate)**	0.141326	0.105881	0.134967
**HIF1Alpha Pathway (VEGF pathway)**	0.132493	0.099263	0.126532
**Hypoxia pathway EMT 1**	0.090554	0.094298	0.067153
**Hypoxia pathway EMT 2**	0.090554	0.094298	0.067153
**Hypoxia pathway EMT 3**	0.090554	0.094298	0.067153
**Hypoxia pathway EMT 4**	0.090554	0.094298	0.067153
**IGF1R Main Pathway**	0.015893	0.011965	0.019413
**IGF1R Signaling Pathway (Cell survival)**	0.033459	0.018962	0.036414
**IGF1R Signaling Pathway (Glucose uptake)**	0	0	0
**IGF1R Signaling Pathway (Glycogen synthesis)**	0	0	0
**IGF1R Signaling Pathway (IKB degradation)**	0	0	0
**IGF1R Signaling Pathway (Protein synthesis)**	0	0	0
**ILK Main Pathway**	0.068918	0.071644	0.082418
**ILK Pathway (Apoptosis)**	0.09406	0.093366	0.088418
**ILK Pathway (Cell adhesion, cell motility, opsonization)**	0.099907	0.098346	0.089643
**ILK Pathway (Cell cycle proliferation)**	0.089771	0.088369	0.080549
**ILK Pathway (Cell migration, retraction)**	0.098321	0.096785	0.08822
**ILK Pathway (Cell motility)**	0.082165	0.083622	0.076734
**ILK Pathway (Cytoskeletal reorganization)**	0.116867	0.11468	0.113455
**ILK Pathway (G2-phase arrest)**	0.089771	0.088369	0.080549
**ILK Pathway (Induced cell proliferation)**	0.191665	0.178978	0.222532
**ILK Pathway (Regulation of intermediate filaments)**	0.109122	0.109536	0.099614
**ILK Pathway (Regulation of junction assembly of desmosomes)**	0.099108	0.097559	0.088926
**ILK Pathway (Wound healing)**	0.099108	0.097559	0.088926
**IL-10 Main Pathway**	0.05028	0.062797	0.047357
**IL-10 Pathway (Gene expression)**	0.109185	0.11326	0.12938
**IL-10 Pathway (Stability determination)**	1.746967	1.812161	2.070086
**IL-10 Pathway (Translational modulation)**	0.171324	0.185209	0.198081
**IL-2 Main Pathway**	0.006802	0.01421	0.030067
**IL-2 Pathway (Actin reorganization)**	0.450109	0.442614	0.411356
**IL-2 Pathway (Apoptosis)**	0	0	0
**IL-2 Pathway (Apoptosis inhibition)**	0	0	0
**IL-2 Pathway (Protein synthesis)**	0.531947	0.523089	0.486148
**IL-6 Main Pathway**	0.02613	0.031138	0.041214
**Integrin SIgnaling Main Pathway**	0.06532	0.072489	0.064511
**Integrin SIgnaling Pathway (Cell survival)**	-0.00349	0.002838	-0.01883
**Integrin SIgnaling Pathway (Cytoskeleton contraction integrin modulation cell**	0.034722	0.047591	0.064333

**invasion and migration)**			
**Integrin SIgnaling Pathway (Focal adhesion and stress fibers)**	-0.00277	0.029683	-0.0077
**Integrin SIgnaling Pathway (Translocation to the nucleus)**	0	0	0
**Interactions Report**	0.162997	0.169737	0.120876
**Interferon Main Pathway**	0.022675	0.015227	0.026707
**Interferon Pathway (Gene expression)**	0	0	0
**Interferon Pathway (Transcription)**	0	0	0
**Interferon Pathway (Translation)**	0	0	0
**IP3 Main Pathway**	0.003895	0.000262	0.002889
**IP3 Pathway (Gene expression)**	0.054371	0.030814	0.059172
**JAK mStat Main Pathway**	0.038166	0.053672	0.055276
**JAK mStat Pathway (Akt pathway)**	-0.0164	-0.0123	-0.00956
**JAK mStat Pathway (JAK degradation)**	0	0	0
**JNK Main Pathway**	0.021239	0.021254	0.024202
**JNK Pathway (Apoptosis, Inflammation, Tumorigenesis, Cell Migration)**	0.083212	0.070011	0.085223
**JNK Pathway (Insulin signaling)**	-0.06142	-0.07178	-0.08404
**MAPK Family Main Pathway**	0.025864	0.024597	0.030346
**MAPK Family Pathway (Chromatin Remodeling)**	0	0	0
**MAPK Family Pathway (Cytoskeleton)**	0.125026	0.100734	0.123862
**MAPK Family Pathway (Gene Expression)**	0.033459	0.018962	0.036414
**MAPK Family Pathway (IKBs Degradation)**	0	0	0
**MAPK Family Pathway (Translation)**	0.187943	0.229454	0.315164
**MAPK Signaling Main Pathway**	0.047831	0.051156	0.057009
**MAPK Signaling Pathway (Cell Survival, Inflammation, Apoptosis, Osmoregulation)**	0.202741	0.193139	0.240365
**MAPK Signaling Pathway (Gene Expression)**	0.089723	0.076726	0.107134
**DDR pathway (MMR)**	-0.05996	-0.07161	-0.12078
**Mismatch Repair Main Pathway**	-0.03712	-0.04433	-0.07477
**Mitochondrial Apopotosis Main Pathway**	-0.013	-0.01961	-0.03383
**Mitochondrial Apopotosis Pathway (Apoptosis)**	0	0	0
**Mitochondrial Apopotosis Pathway (Depolarization)**	0	0	0
**Mitochondrial Apopotosis Pathway (DNA fragmentation)**	-0.15882	-0.18053	-0.17816
**Mitochondrial Apopotosis Pathway (Gene expression)**	0	0	0
**mTOR Main Pathway**	0.027284	0.028647	0.035934
**mTOR Pathway (Actin organization)**	0.056245	0.051723	0.070794
**mTOR Pathway (Akt signaling)**	0	0	0
**mTOR Pathway (Scanning)**	0	0	0
**mTOR Pathway (Translation on)**	0	0	0
**mTOR Pathway (VEGF pathway)**	0.162997	0.169737	0.120876
**NGF (Negative) Main Pathway**	0	0	0
**NGF (Negative) Pathway (Apoptosis)**	0	0	0
**NGF (Positive) Main Pathway**	0.057031	0.067076	0.072852
**NHEJ mechanisms of DSBs repair effect**	0	0	0
**Notch Main Pathway**	0	0	0
**DDR Pathway (NER)**	0	0	0
**p38 (Negative) Main Signaling Pathway**	0.03148	0.034787	0.045768
**p38 (Positive) Main Signaling Pathway**	0.031416	0.034717	0.045675
**p53 Signaling (Negative) Main Pathway**	0.07621	0.082976	0.094226
**p53 Signaling (Negative) Pathway (p53 Degradation)**	0	0	0
**PAK Main Pathway**	0.030789	0.035651	0.037526
**PAK Pathway (Actin Cytoskeleton)**	0.024558	0.029039	0.029045
**PAK Pathway (Myosin Activation)**	0.026855	0.034534	0.030924

**PPAR Main Pathway**	0.040901	0.046519	0.050552
**PTEN Main Pathway**	0	0	0
**RANK Signaling in Osteoclast Main Pathway**	0.049791	0.054358	0.064297
**RANK Signaling in Osteoclast Pathway (IKBs Degradation)**	0	0	0
**RAS Main Pathway**	0.015209	0.016477	0.017816
**Telomere Main Pathway**	0.041548	0.067783	0.069973
**RNA Polymerase II Complex Pathway**	0	0	0
**Cell Cycle Pathway (SCC during S-phase)**	0	0	0
**SMAD (Negative) Main Pathway**	0.124387	0.134865	0.159739
**SMAD (Negative) Pathway (Degradation)**	0.011753	0.014179	0.017958
**SMAD (Positive) Main Pathway**	0.124387	0.134865	0.159739
**SMAD (Positive) Pathway (Degradation)**	0.011753	0.014179	0.017958
**Cell Cycle Pathway (Metaphase-Anaphase)**	0	0	0
**Cell Cycle Pathway (Origin of S-phase)**	0	0	0
**STAT3 Main Pathway**	0.009488	0.016137	0.026291
**TGF beta Main Pathway**	0.015453	0.019597	0.025801
**TGF beta Pathway (Epithelial mesehchymal transition)**	0.149546	0.18252	0.222467
**TGF beta Pathway (Post-transcriptional G1 arrest)**	0.113655	0.138715	0.169075
**TGF beta Pathway (SnON degradation)**	0.139347	0.145183	0.216042
**TGF beta Pathway (Tumorigenesis)**	0.199068	0.207404	0.308632
**TGF beta Pathway (Tumor suppression)**	0.199068	0.207404	0.308632
**TNF (Negative) Main Pathway**	0	0	0
**TNF (Negative) Pathway (Apoptosis)**	0	0	0
**TNF (Positive) Main Pathway**	0.081025	0.088607	0.119319
**TNF (Positive) Pathway (Gene expression, Cell survival)**	0.132341	0.144725	0.194888
**TNF (Positive) Pathway (IKBs degradation)**	0	0	0
**TRAF (Negative) Main Pathway**	0	0	0
**TRAF (Negative) Pathway (IKBs Degradation)**	0	0	0
**TRAF (Positive) Main Pathway**	0.017418	0.018148	0.027005
**TRAF (Positive) Pathway (IKBs Degradation)**	0	0	0
**Transcription of mRNA Pathway**	0	0	0
**Cell Cycle Pathway (End of S-phase)**	0	0	0
**Translation Regulation of EIF4F activity**	0.013912	0.016783	0.021257
**Ubiquitin Proteasome Main Pathway**	0.006854	0.004944	0.008053
**Ubiquitin Proteasome Pathway (Degraded Protein)**	0.018562	0.01339	0.021809
**VEGF Main Pathway**	0.051182	0.054175	0.069702
**VEGF Pathway (Actin Reorganization)**	0.100764	0.106657	0.137225
**VEGF Pathway (Nitric Oxide Production)**	0	0	0
**Wnt Main Pathway**	0.044438	0.043838	0.044132
**Wnt Pathway (Ctnn-b Degradation)**	0	0	0
**IL-6 Pathway (IKBs degradation)**	0	0	0

**Pathway**	**M_68_8h_1**	**M_68_8h_2**	**M_68_8h_3**	**p-value_Mean**
**AHR Main Pathway**	-0.06437	-0.10014	-0.06936	0.049535
**AHR Pathway (AHR Degradation)**	-0.12368	-0.18349	-0.13754	0.049535
**AHR Pathway (Cath-D Expression)**	-0.05593	-0.05498	-0.04877	0.049535
**AHR Pathway (C-MycExpression)**	-0.05264	-0.05174	-0.0459	0.049535
**AHR Pathway (PS2 Gene Expression)**	-0.11586	-0.18025	-0.12486	0.049535
**AKT Main Pathway**	0.00589	-0.00814	0.006719	0.512691

**AKT Pathway (Aggregation & Neurodegeneration)**	0.008879	-0.03553	0.025452	0.512691
**AKT Pathway (Apoptosis Inhibition)**	0.008531	-0.03414	0.024454	0.512691
**AKT Pathway (Blocks Apoptosis)**	0.033339	0.038397	0.047346	0.049535
**AKT Pathway (Cardiovascular Homeostasis)**	0.009668	-0.03869	0.027714	0.512691
**AKT Pathway (Caspase Cascade)**	0.004006	-0.05626	0.003268	0.827259
**AKT Pathway (Cell Cycle)**	0	0	0	1
**AKT Pathway (Cell Cycle Progression)**	-0.09389	-0.15746	-0.11465	0.049535
**AKT Pathway (Cell Survival)**	-0.57341	-0.40707	-0.45473	0.049535
**AKT Pathway (Death Genes)**	-0.02285	-0.07251	-0.01587	0.049535
**AKT Pathway (Elevation of Glucose Import)**	-0.02165	-0.06869	-0.01504	0.049535
**AKT Pathway (ERK Pathway)**	-0.01661	-0.03803	-0.01111	0.049535
**AKT Pathway (Genetic Stability)**	-0.05862	-0.10832	-0.04765	0.049535
**AKT Pathway (Glucose Uptake)**	0.009888	-0.03957	0.028344	0.512691
**AKT Pathway (Glycogen Synthesis)**	-0.07119	-0.16089	-0.10842	0.049535
**AKT Pathway (Insulin Stimulated Mitogenesis)**	0.009668	-0.03869	0.027714	0.512691
**AKT Pathway (JNK Pathway)**	-0.02285	-0.07251	-0.01587	0.049535
**AKT Pathway (Neuroprotection)**	0.009668	-0.03869	0.027714	0.512691
**AKT Pathway (NF-kB Pathway)**	0.009064	-0.03627	0.025982	0.512691
**AKT Pathway (p53 Degradation)**	0	0	0	1
**AKT Pathway (p73 Mediated Apoptosis)**	-0.02351	-0.07458	-0.01632	0.049535
**AKT Pathway (Protein Synthesis)**	0	0	0	1
**AKT Pathway (Regeneration of Cyclic Nucleotide)**	-0.07771	-0.11228	-0.06044	0.049535
**AKT Pathway (Respiratory Burst)**	0.009668	-0.03869	0.027714	0.512691
**AKT Pathway (Survival Genes)**	-0.01477	-0.03381	-0.00988	0.049535
**AKT Pathway (Synaptic Signaling)**	0.000689	-0.02454	0.000775	0.827259
**AKT Pathway (Translation)**	0	0	0	1
**Androgen Receptor Pathway**	0.029706	0.035052	0.032193	0.049535
**Androgen Receptor Pathway (Apoptosis)**	-0.02827	-0.11316	-0.02031	0.049535
**Androgen Receptor Pathway (Degradation)**	-0.03202	0.019867	-0.00427	0.827259
**Androgen Receptor Pathway (Cell Survival & Cell Growth)**	-0.04213	0.019283	0.012881	0.512691
**Androgen Receptor Pathway (Gonadotropin Regulation)**	0.017851	0.065211	0.017681	0.275234
**Androgen Receptor Pathway (Histone Modification)**	0.017851	0.065211	0.017681	0.275234
**Androgen Receptor Pathway (Prostate Differentiation & Development)**	0.017851	0.065211	0.017681	0.275234
**Androgen Receptor Pathway (Sexual Differentiation & Sexual Maturation at Puberty)**	0.017851	0.065211	0.017681	0.275234
**ATM Main Pathway**	0.158539	0.205274	0.17002	0.049535
**ATM Pathway (Apoptosis)**	-0.38847	-0.22208	-0.36657	0.049535
**ATM Pathway (Apoptosis, Senescense)**	-0.15413	0.213744	-0.11882	0.512691
**ATM Pathway (Cell Cicle Checkpoint Control)**	-0.43232	-0.23797	-0.39592	0.049535
**ATM Pathway (Cell Survival)**	-0.07079	0.003468	-0.07546	0.275234
**ATM Pathway (Checkpoint Activation)**	-0.43232	-0.23797	-0.39592	0.049535
**ATM Pathway (DNA Repair)**	-0.21796	-0.23873	-0.2009	0.049535
**ATM Pathway (G2_M Checkpoint Arrest)**	0.669616	0.920645	0.732046	0.049535
**ATM Pathway (G2 Mitosis Progression)**	0.309204	0.618077	0.316673	0.049535
**ATM Pathway (MDMX Ubiquitination, Degradation)**	0.014284	0.361211	0.033396	0.049535
**ATM Pathway (NF-kB Pathway)**	-0.43232	-0.23797	-0.39592	0.049535
**ATM Pathway (Synaptic Vesicle Transport)**	-0.43232	-0.23797	-0.39592	0.049535
**ATM Pathway (S-Phase Arrest)**	-0.43232	-0.23797	-0.39592	0.049535
**ATM Pathway (S-Phase Progression)**	-0.08885	-0.03299	-0.11643	0.049535
**DDR Pathway (BRCA1-induced responses)**	0.030069	0.047725	0.081122	0.049535
**BRCA1 Main Pathway**	-0.21046	-0.23478	-0.2302	0.049535

**cAMP Main Pathway**	0.005276	-0.00149	-0.00052	0.827259
**cAMP Pathway (Axonal Growth)**	-0.10001	-0.04486	-0.07184	0.049535
**cAMP Pathway (Cardiovascular Homeostasis)**	-0.21431	-0.09614	-0.15394	0.049535
**cAMP Pathway (Cell Growth)**	0	0	0	1
**cAMP Pathway (Cell Proliferation)**	-0.2308	-0.10353	-0.16578	0.049535
**cAMP Pathway (Cell Survival)**	-0.08297	-0.05436	-0.06034	0.049535
**cAMP Pathway (Cell Survival, Chemotaxis)**	0	0	0	1
**cAMP Pathway (Cytokine Production)**	0	0	0	1
**cAMP Pathway (Degradation of Cell Cycle Regulators)**	-0.09679	-0.04342	-0.06952	0.049535
**cAMP Pathway (Endothelial Cell Regulation)**	0.234869	0.254507	0.209269	0.049535
**cAMP Pathway (Glycogen Synthesis)**	-0.27276	-0.12236	-0.19592	0.049535
**cAMP Pathway (Glycolysis)**	-0.14183	-0.10622	-0.14715	0.049535
**cAMP Pathway (Metabolic Energy)**	-0.02827	-0.11316	-0.02031	0.049535
**cAMP Pathway (Myocardial Contraction)**	-0.0533	-0.04138	-0.04665	0.049535
**cAMP Pathway (Oncogenesis)**	-0.21431	-0.09614	-0.15394	0.049535
**cAMP Pathway (Protein Retention)**	0.097473	0.107004	0.071344	0.12663
**cAMP Pathway (Regulation of Cytoskeleton)**	-0.18752	-0.08412	-0.1347	0.049535
**Caspase Cascade Main**	-0.02707	-0.01764	-0.02151	0.049535
**Caspase Cascade (Activated Tissue Transglutaminase)**	-0.02413	-0.0259	-0.00714	0.049535
**Caspase Cascade (Apoptosis)**	-0.01179	0.007206	0.000624	0.512691
**Caspase Cascade (Cell Survival)**	-0.06442	-0.12826	-0.09231	0.049535
**Caspase Cascade (ICAD Degradation)**	-0.03425	-0.02491	-0.02437	0.049535
**CD40 Main Pathway**	-0.08786	-0.09991	-0.06242	0.049535
**CD40 Pathway (Cell Survival)**	0	0	0	1
**CD40 Pathway (Gene Expression)**	-0.10357	-0.12273	-0.09427	0.049535
**CD40 Pathway (IKBs Degradation)**	-0.07311	-0.08663	-0.06654	0.049535
**Cellular Anti Apoptosis Main Pathway**	0.023983	0.017682	0.021786	0.049535
**Cellular Anti Apoptosis Pathway (Apoptosis)**	0.075262	0.056742	0.067053	0.049535
**Cellular Anti Apoptosis Pathway (Depolarization)**	0	0	0	1
**Chemokine Main Pathway**	0.022025	0.010853	0.019053	0.049535
**Chemokine Pathway (Cell Activation)**	0.070355	0.088573	0.066667	0.049535
**Chemokine Pathway (Gene Expression, Apoptosis)**	0.017917	0.01199	0.012043	0.049535
**Chemokine Pathway (Internalization, Degradation, Recycling)**	-0.07766	-0.12433	-0.06298	0.049535
**Chromatin Main Pathway**	0.009341	-0.01124	-0.00857	0.512691
**Chromatin Pathway (Octamer Sliding)**	0.010778	-0.01297	-0.00989	0.512691
**Chromatin Pathway (Octamer Transfer)**	0.009341	-0.01124	-0.00857	0.512691
**Circadian Main Pathway**	-0.10269	-0.13854	-0.15761	0.049535
**CREB Main Pathway**	0.020874	0.011177	0.021831	0.049535
**CREB Pathway (Gene Expression Pathway)**	-0.03385	-0.0542	-0.02745	0.049535
**Cytokine Main Pathway**	-0.02238	-0.02724	-0.01729	0.049535
**DDR pathway Apoptosis**	-0.26971	-0.43357	-0.28618	0.049535
**DDR Main pathway**	-0.46187	-0.76159	-0.50624	0.049535
**DNA Repair Mechanisms Pathway**	-0.05919	-0.05571	-0.06205	0.049535
**EGFR Main Pathway**	0.003045	-0.00109	-0.00021	0.827259
**ErbB Family Main Pathway**	-0.02333	-0.0258	-0.01938	0.049535
**ErbB Family Pathway (Anti-Apoptosis)**	0	0	0	1
**ERK Signaling Main Pathway**	0.018865	0.012656	0.018812	0.049535
**Erythropoeitin Main Pathway**	-0.02739	-0.02278	-0.01518	0.049535
**Estrogen Main Pathway**	0.042054	0.032547	0.036362	0.049535
**Fas Signaling Pathway (Negative)**	-0.01176	-0.00956	-0.01375	0.275234
**Fas Signaling Pathway (Positive)**	0	0	0	1

**FLT3 Main Pathway**	-0.05564	-0.06038	-0.03312	0.049535
**Glucocorticoid Receptor Main Pathway**	0.037781	0.051127	0.049969	0.049535
**Glucocorticoid Receptor Pathway (Cell cycle arrest)**	1.317439	1.543869	1.330079	0.049535
**Glucocorticoid Receptor Pathway (Cell cycle progression)**	0.363877	0.335885	0.24129	0.049535
**Glucocorticoid Receptor Pathway (Gene expression)**	0.048789	0.067921	0.06225	0.049535
**Glucocorticoid Receptor Pathway (Inflammatory cytokines)**	0.013308	0.016366	0.024537	0.049535
**Glucocorticoid Receptor Pathway (SMAD signaling)**	0.041376	0.041969	0.039023	0.049535
**GPCR Main Pathway**	0.031291	0.016648	0.027094	0.049535
**GPCR Pathway (Gene expression)**	0.038218	0.077915	0.050367	0.049535
**Growth Hormone Main Pathway**	0.037593	0.032532	0.048963	0.049535
**Growth Hormone Pathway (Cell survival)**	0	0	0	1
**Growth Hormone Pathway (Gene expression)**	0.063391	0.069805	0.079005	0.049535
**Growth Hormone Pathway (Glucose uptake)**	0.032291	0.027944	0.042058	0.049535
**Growth Hormone Pathway (Protein synthesis)**	0	0	0	1
**GSK3 Main Pathway**	0.024287	0.01332	0.014622	0.049535
**GSK3 Pathway (Degradation)**	0.015108	0.033905	0.022315	0.049535
**GSK3 Pathway (Gene expression)**	0.031842	0.049967	0.034038	0.049535
**GSK3 Pathway (Translation)**	0.047654	0.049715	0.033323	0.049535
**G-protein Pathway (Ras family GTPases)**	0.052478	0.023949	0.035196	0.049535
**Hedgehog Main Pathway**	-0.16926	-0.20426	-0.21918	0.049535
**Hedgehog Pathway (Repression of Hh, BMP)**	0.260153	0.356839	0.235476	0.049535
**Hedgehog Pathway (Activation of BMP, Ptc, WNT)**	0	0	0	1
**HGF Main Pathway**	0.019621	0.023455	0.029713	0.049535
**HGF Pathway (Anoikis)**	0.064354	0.049013	0.059989	0.049535
**HGF Pathway (Cell adhesion, cell mirgation)**	0.041301	0.03371	0.054801	0.049535
**HGF Pathway (Cell cycle progression)**	0.349746	0.542723	0.408003	0.049535
**HGF Pathway (Cell polarity, cell motility)**	0	0	0	1
**HGF Pathway (Cell scattering)**	-0.10136	-0.18101	-0.11033	0.049535
**HGF Pathway (Cell survival)**	-0.10136	-0.18101	-0.11033	0.049535
**HGF Pathway (IP3 pathway)**	-0.11656	-0.20816	-0.12687	0.049535
**HGF Pathway (PKC pathway)**	0	0	0	1
**HIF1-Alpha Main Pathway**	-0.09567	-0.10336	-0.07394	0.049535
**HIF1Alpha Pathway (Gene expression)**	0.106653	0.139728	0.108969	0.049535
**HIF1Alpha Pathway (HIF1alpha degradation)**	0.041667	0.071171	0.065564	0.049535
**HIF1Alpha Pathway (NOS pathway)**	0.123061	0.161225	0.125733	0.049535
**HIF1Alpha Pathway (p53 Hypoxia pathway)**	0.035715	0.061004	0.056198	0.049535
**HIF1Alpha Pathway (Pyruvate)**	0.106653	0.139728	0.108969	0.049535
**HIF1Alpha Pathway (VEGF pathway)**	0.099987	0.130995	0.102158	0.049535
**Hypoxia pathway EMT 1**	0.672629	0.867643	0.862	0.049535
**Hypoxia pathway EMT 2**	0.672629	0.867643	0.862	0.049535
**Hypoxia pathway EMT 3**	0.672629	0.867643	0.862	0.049535
**Hypoxia pathway EMT 4**	0.672629	0.867643	0.862	0.049535
**IGF1R Main Pathway**	-0.01678	-0.02804	-0.01779	0.049535
**IGF1R Signaling Pathway (Cell survival)**	0.049418	0.063943	0.042063	0.049535
**IGF1R Signaling Pathway (Glucose uptake)**	0	0	0	1
**IGF1R Signaling Pathway (Glycogen synthesis)**	0.124586	0.281557	0.189735	0.049535
**IGF1R Signaling Pathway (IKB degradation)**	0.082859	0.098184	0.075413	0.049535
**IGF1R Signaling Pathway (Protein synthesis)**	0.085777	0.089488	0.059981	0.049535
**ILK Main Pathway**	0.062214	0.076096	0.069215	0.049535
**ILK Pathway (Apoptosis)**	0.03026	0.043171	0.055078	0.049535
**ILK Pathway (Cell adhesion, cell motility, opsonization)**	0.010026	0.034167	0.040286	0.049535

**ILK Pathway (Cell cycle proliferation)**	0.005936	0.018401	0.033992	0.049535
**ILK Pathway (Cell migration, retraction)**	0.004267	0.027682	0.034004	0.049535
**ILK Pathway (Cell motility)**	0.052112	0.065306	0.069816	0.049535
**ILK Pathway (Cytoskeletal reorganization)**	0.063443	0.091908	0.08869	0.049535
**ILK Pathway (G2-phase arrest)**	0.005936	0.018401	0.033992	0.049535
**ILK Pathway (Induced cell proliferation)**	0.021046	0.09348	0.066284	0.049535
**ILK Pathway (Regulation of intermediate filaments)**	0.02165	0.049245	0.051957	0.049535
**ILK Pathway (Regulation of junction assembly of desmosomes)**	0.009946	0.033894	0.039964	0.049535
**ILK Pathway (Wound healing)**	0.014039	0.041256	0.0472	0.049535
**IL-10 Main Pathway**	-0.03435	-0.01472	0.011024	0.512691
**IL-10 Pathway (Gene expression)**	0.029072	0.045698	0.066849	0.049535
**IL-10 Pathway (Stability determination)**	1.320249	2.187984	1.677377	0.049535
**IL-10 Pathway (Translational modulation)**	-0.07712	0.162547	0.088272	0.512691
**IL-2 Main Pathway**	0.027396	0.053241	0.037157	0.049535
**IL-2 Pathway (Actin reorganization)**	-0.28239	-0.2903	-0.25479	0.049535
**IL-2 Pathway (Apoptosis)**	0.011731	0.016432	0.060402	0.275234
**IL-2 Pathway (Apoptosis inhibition)**	-0.03298	-0.00849	0.032668	0.512691
**IL-2 Pathway (Protein synthesis)**	-0.33374	-0.34308	-0.30112	0.049535
**IL-6 Main Pathway**	0.03853	0.053268	0.042454	0.049535
**Integrin SIgnaling Main Pathway**	0.022691	0.029829	0.038915	0.049535
**Integrin SIgnaling Pathway (Cell survival)**	-0.07757	-0.11522	-0.06585	0.049535
**Integrin SIgnaling Pathway (Cytoskeleton contraction integrin modulation cell invasion and migration)**	0.024537	0.02991	0.025814	0.512691
**Integrin SIgnaling Pathway (Focal adhesion and stress fibers)**	-0.12471	-0.1405	-0.11233	0.049535
**Integrin SIgnaling Pathway (Translocation to the nucleus)**	0.385879	0.465976	0.353975	0.049535
**Interactions Report**	0.655367	0.866284	0.854477	0.049535
**Interferon Main Pathway**	-0.06466	-0.05336	-0.04675	0.049535
**Interferon Pathway (Gene expression)**	-0.06614	0	-0.06287	0.19043
**Interferon Pathway (Transcription)**	0	0	0	1
**Interferon Pathway (Translation)**	0	0	0	1
**IP3 Main Pathway**	-0.0094	-0.03331	-0.00701	0.049535
**IP3 Pathway (Gene expression)**	0.028517	0.042542	0.021219	0.049535
**JAK mStat Main Pathway**	-0.0392	-0.06117	-0.03212	0.049535
**JAK mStat Pathway (Akt pathway)**	-0.03983	-0.11392	-0.05652	0.049535
**JAK mStat Pathway (JAK degradation)**	-0.01309	-0.02938	-0.01934	0.049535
**JNK Main Pathway**	0.012102	-0.00476	0.016938	0.275234
**JNK Pathway (Apoptosis, Inflammation, Tumorigenesis, Cell Migration)**	0.066545	0.093052	0.092034	0.049535
**JNK Pathway (Insulin signaling)**	-0.28018	-0.41042	-0.24144	0.049535
**MAPK Family Main Pathway**	-0.0007	0.006857	0.012528	0.275234
**MAPK Family Pathway (Chromatin Remodeling)**	0.053052	0.097899	0.059982	0.049535
**MAPK Family Pathway (Cytoskeleton)**	0.116905	0.216265	0.13639	0.049535
**MAPK Family Pathway (Gene Expression)**	-0.02857	0.05823	-0.00468	0.827259
**MAPK Family Pathway (IKBs Degradation)**	0.095607	0.113289	0.087015	0.049535
**MAPK Family Pathway (Translation)**	0	0	0	1
**MAPK Signaling Main Pathway**	0.03125	0.036812	0.039413	0.049535
**MAPK Signaling Pathway (Cell Survival, Inflammation, Apoptosis, Osmoregulation)**	0.145247	0.25387	0.184091	0.049535
**MAPK Signaling Pathway (Gene Expression)**	0.046736	0.090164	0.063802	0.049535
**DDR pathway (MMR)**	-0.26616	-0.25979	-0.2396	0.049535
**Mismatch Repair Main Pathway**	-0.22647	-0.24101	-0.21887	0.049535
**Mitochondrial Apopotosis Main Pathway**	-0.03076	-0.04208	-0.03451	0.049535
**Mitochondrial Apopotosis Pathway (Apoptosis)**	-0.07991	-0.05812	-0.05687	0.049535

**Mitochondrial Apopotosis Pathway (Depolarization)**	0	0	0	1
**Mitochondrial Apopotosis Pathway (DNA fragmentation)**	-0.17178	-0.34204	-0.24617	0.049535
**Mitochondrial Apopotosis Pathway (Gene expression)**	0.480442	0.627712	0.473281	0.049535
**mTOR Main Pathway**	0.035225	0.034356	0.02806	0.049535
**mTOR Pathway (Actin organization)**	0.002343	-0.0029	-0.00086	0.512691
**mTOR Pathway (Akt signaling)**	0	0	0	1
**mTOR Pathway (Scanning)**	0.058136	0.048354	0.038148	0.049535
**mTOR Pathway (Translation on)**	0.023492	0.024056	0.006997	0.049535
**mTOR Pathway (VEGF pathway)**	0.100001	0.170811	0.157354	0.049535
**NGF (Negative) Main Pathway**	0.045333	0.087918	0.034155	0.049535
**NGF (Negative) Pathway (Apoptosis)**	0.062769	0.121733	0.047292	0.049535
**NGF (Positive) Main Pathway**	-0.02077	-0.04801	-0.03026	0.049535
**NHEJ mechanisms of DSBs repair effect**	-0.20586	-0.32666	-0.25265	0.049535
**Notch Main Pathway**	-0.08837	-0.11048	-0.09001	0.049535
**DDR Pathway (NER)**	-0.04049	-0.05262	-0.0463	0.049535
**p38 (Negative) Main Signaling Pathway**	0.017595	0.019715	0.022917	0.049535
**p38 (Positive) Main Signaling Pathway**	0.017559	0.019675	0.02287	0.049535
**p53 Signaling (Negative) Main Pathway**	0.061814	0.103394	0.083728	0.049535
**p53 Signaling (Negative) Pathway (p53 Degradation)**	-0.01511	-0.03391	-0.02231	0.049535
**PAK Main Pathway**	0.049111	0.045515	0.044179	0.049535
**PAK Pathway (Actin Cytoskeleton)**	0.012665	0.009113	0.016786	0.049535
**PAK Pathway (Myosin Activation)**	0.050618	0.054851	0.045101	0.049535
**PPAR Main Pathway**	0.040095	0.056441	0.054366	0.049535
**PTEN Main Pathway**	0.042037	0.084624	0.046073	0.049535
**RANK Signaling in Osteoclast Main Pathway**	0.087268	0.070001	0.081172	0.049535
**RANK Signaling in Osteoclast Pathway (IKBs Degradation)**	0.062144	0.073638	0.05656	0.049535
**RAS Main Pathway**	-0.01246	-0.02331	-0.00882	0.049535
**Telomere Main Pathway**	-0.06735	-0.10059	-0.09085	0.049535
**RNA Polymerase II Complex Pathway**	0.010907	-0.00357	0.002492	0.512691
**Cell Cycle Pathway (SCC during S-phase)**	0	0	0	1
**SMAD (Negative) Main Pathway**	0.152455	0.216095	0.15756	0.049535
**SMAD (Negative) Pathway (Degradation)**	0.010159	0.022798	0.015005	0.049535
**SMAD (Positive) Main Pathway**	0.152455	0.216095	0.15756	0.049535
**SMAD (Positive) Pathway (Degradation)**	0.010159	0.022798	0.015005	0.049535
**Cell Cycle Pathway (Metaphase-Anaphase)**	0.155642	0.149945	0.138099	0.049535
**Cell Cycle Pathway (Origin of S-phase)**	-0.07339	-0.08562	-0.07692	0.049535
**STAT3 Main Pathway**	0.027086	0.034411	0.03791	0.049535
**TGF beta Main Pathway**	0.005655	-0.00045	0.013125	0.512691
**TGF beta Pathway (Epithelial mesehchymal transition)**	0.001216	0.01139	0.055551	0.512691
**TGF beta Pathway (Post-transcriptional G1 arrest)**	0.009857	0.076379	0.037013	0.12663
**TGF beta Pathway (SnON degradation)**	0.249099	0.319436	0.230387	0.049535
**TGF beta Pathway (Tumorigenesis)**	0.355856	0.456337	0.329125	0.049535
**TGF beta Pathway (Tumor suppression)**	0.355856	0.456337	0.329125	0.049535
**TNF (Negative) Main Pathway**	-0.01829	0.056983	-0.02007	0.512691
**TNF (Negative) Pathway (Apoptosis)**	-0.0597	-0.01827	-0.0743	0.049535
**TNF (Positive) Main Pathway**	0.160042	0.262219	0.149374	0.049535
**TNF (Positive) Pathway (Gene expression, Cell survival)**	0.188985	0.287946	0.177962	0.049535
**TNF (Positive) Pathway (IKBs degradation)**	0.069049	0.08182	0.062844	0.049535
**TRAF (Negative) Main Pathway**	-0.01982	-0.00438	-0.03677	0.049535
**TRAF (Negative) Pathway (IKBs Degradation)**	0.106129	0.159228	0.111827	0.049535
**TRAF (Positive) Main Pathway**	0.012435	0.037854	0.032224	0.12663

**TRAF (Positive) Pathway (IKBs Degradation)**	0.106129	0.159228	0.111827	0.049535
**Transcription of mRNA Pathway**	0.023029	0.018459	0.017393	0.049535
**Cell Cycle Pathway (End of S-phase)**	-0.08643	-0.0717	-0.08309	0.049535
**Translation Regulation of EIF4F activity**	-0.1337	-0.10102	-0.10008	0.049535
**Ubiquitin Proteasome Main Pathway**	-0.02491	-0.02184	-0.02299	0.049535
**Ubiquitin Proteasome Pathway (Degraded Protein)**	-0.02287	-0.01862	-0.01656	0.049535
**VEGF Main Pathway**	0.011158	-0.00244	0.022635	0.512691
**VEGF Pathway (Actin Reorganization)**	0.145583	0.117731	0.146024	0.049535
**VEGF Pathway (Nitric Oxide Production)**	0	0	0	1
**Wnt Main Pathway**	0.02919	0.04233	0.038988	0.049535
**Wnt Pathway (Ctnn-b Degradation)**	-0.01684	-0.03778	-0.02487	0.049535
**IL-6 Pathway (IKBs degradation)**	0.103574	0.12273	0.094266	0.049535

Pathway	M_68_16h_1	M_68_16h_2	M_68_16h_3	p-value_Mean
**AHR Main Pathway**	0	0	0	1
**AHR Pathway (AHR Degradation)**	0	0	0	1
**AHR Pathway (Cath-D Expression)**	0	0	0	1
**AHR Pathway (C-MycExpression)**	0	0	0	1
**AHR Pathway (PS2 Gene Expression)**	0	0	0	1
**AKT Main Pathway**	0.027261	0.020475	0.024925	0.049535
**AKT Pathway (Aggregation & Neurodegeneration)**	0.075494	0.083752	0.088529	0.049535
**AKT Pathway (Apoptosis Inhibition)**	0.072534	0.080468	0.085057	0.049535
**AKT Pathway (Blocks Apoptosis)**	0	0	0	1
**AKT Pathway (Cardiovascular Homeostasis)**	0.082205	0.091197	0.096398	0.049535
**AKT Pathway (Caspase Cascade)**	0.038801	0.056302	0.046288	0.049535
**AKT Pathway (Cell Cycle)**	0.225955	0.165655	0.199176	0.049535
**AKT Pathway (Cell Cycle Progression)**	-0.04908	-0.05365	-0.04854	0.049535
**AKT Pathway (Cell Survival)**	0	0	0	1
**AKT Pathway (Death Genes)**	0.017364	0.029984	0.026556	0.049535
**AKT Pathway (Elevation of Glucose Import)**	0.01645	0.028406	0.025159	0.049535
**AKT Pathway (ERK Pathway)**	0	0	0	1
**AKT Pathway (Genetic Stability)**	0.016895	0.029174	0.025839	0.049535
**AKT Pathway (Glucose Uptake)**	0.084073	0.09327	0.098589	0.049535
**AKT Pathway (Glycogen Synthesis)**	0	0	0	1
**AKT Pathway (Insulin Stimulated Mitogenesis)**	0.082205	0.091197	0.096398	0.049535
**AKT Pathway (JNK Pathway)**	0.017364	0.029984	0.026556	0.049535
**AKT Pathway (Neuroprotection)**	0.082205	0.091197	0.096398	0.049535
**AKT Pathway (NF-kB Pathway)**	0.077067	0.085497	0.090373	0.049535
**AKT Pathway (p53 Degradation)**	0	0	0	1
**AKT Pathway (p73 Mediated Apoptosis)**	0	0	0	1
**AKT Pathway (Protein Synthesis)**	0	0	0	1
**AKT Pathway (Regeneration of Cyclic Nucleotide)**	-0.00177	0.006272	0.008674	0.512691
**AKT Pathway (Respiratory Burst)**	0.082205	0.091197	0.096398	0.049535
**AKT Pathway (Survival Genes)**	0	0	0	1
**AKT Pathway (Synaptic Signaling)**	0.0133	0.022967	0.020341	0.049535
**AKT Pathway (Translation)**	0	0	0	1
**Androgen Receptor Pathway**	0.021171	0.017441	0.014148	0.049535
**Androgen Receptor Pathway (Apoptosis)**	0.041674	0.071962	0.063736	0.049535
**Androgen Receptor Pathway (Degradation)**	-0.03004	-0.04684	-0.02532	0.049535

**Androgen Receptor Pathway (Cell Survival & Cell Growth)**	-0.0574	-0.06307	-0.04279	0.049535
**Androgen Receptor Pathway (Gonadotropin Regulation)**	-0.04088	-0.04067	-0.02556	0.049535
**Androgen Receptor Pathway (Histone Modification)**	-0.04088	-0.04067	-0.02556	0.049535
**Androgen Receptor Pathway (Prostate Differentiation & Development)**	-0.04088	-0.04067	-0.02556	0.049535
**Androgen Receptor Pathway (Sexual Differentiation & Sexual Maturation at Puberty)**	-0.04088	-0.04067	-0.02556	0.049535
**ATM Main Pathway**	0.014603	0.024598	0.025323	0.049535
**ATM Pathway (Apoptosis)**	0	0	0	1
**ATM Pathway (Apoptosis, Senescense)**	0	0	0	1
**ATM Pathway (Cell Cicle Checkpoint Control)**	0	0	0	1
**ATM Pathway (Cell Survival)**	0	0	0	1
**ATM Pathway (Checkpoint Activation)**	0	0	0	1
**ATM Pathway (DNA Repair)**	0	0	0	1
**ATM Pathway (G2_M Checkpoint Arrest)**	0.062396	0.105099	0.108197	0.049535
**ATM Pathway (G2 Mitosis Progression)**	0	0	0	1
**ATM Pathway (MDMX Ubiquitination, Degradation)**	0	0	0	1
**ATM Pathway (NF-kB Pathway)**	0	0	0	1
**ATM Pathway (Synaptic Vesicle Transport)**	0	0	0	1
**ATM Pathway (S-Phase Arrest)**	0	0	0	1
**ATM Pathway (S-Phase Progression)**	0	0	0	1
**DDR Pathway (BRCA1-induced responses)**	0.092252	0.160296	0.115965	0.049535
**BRCA1 Main Pathway**	-0.02033	-0.03881	-0.04374	0.049535
**cAMP Main Pathway**	0.007278	0.002828	0.004454	0.049535
**cAMP Pathway (Axonal Growth)**	-0.07975	-0.08535	-0.06909	0.049535
**cAMP Pathway (Cardiovascular Homeostasis)**	-0.1709	-0.18289	-0.14805	0.049535
**cAMP Pathway (Cell Growth)**	0	0	0	1
**cAMP Pathway (Cell Proliferation)**	-0.18405	-0.19696	-0.15943	0.049535
**cAMP Pathway (Cell Survival)**	-0.04273	-0.04572	-0.03701	0.049535
**cAMP Pathway (Cell Survival, Chemotaxis)**	0	0	0	1
**cAMP Pathway (Cytokine Production)**	0	0	0	1
**cAMP Pathway (Degradation of Cell Cycle Regulators)**	-0.07718	-0.0826	-0.06686	0.049535
**cAMP Pathway (Endothelial Cell Regulation)**	0.278873	0.257925	0.250245	0.049535
**cAMP Pathway (Glycogen Synthesis)**	-0.21751	-0.23277	-0.18842	0.049535
**cAMP Pathway (Glycolysis)**	-0.18886	-0.19399	-0.14174	0.049535
**cAMP Pathway (Metabolic Energy)**	0.041674	0.071962	0.063736	0.049535
**cAMP Pathway (Myocardial Contraction)**	-0.04659	-0.05595	-0.05383	0.049535
**cAMP Pathway (Oncogenesis)**	-0.1709	-0.18289	-0.14805	0.049535
**cAMP Pathway (Protein Retention)**	-0.14954	-0.16003	-0.12954	0.049535
**cAMP Pathway (Regulation of Cytoskeleton)**	-0.14954	-0.16003	-0.12954	0.049535
**Caspase Cascade Main**	-0.03502	-0.03593	-0.03086	0.049535
**Caspase Cascade (Activated Tissue Transglutaminase)**	-0.0497	-0.06765	-0.05305	0.049535
**Caspase Cascade (Apoptosis)**	-0.04195	-0.06161	-0.05543	0.049535
**Caspase Cascade (Cell Survival)**	0	0	0	1
**Caspase Cascade (ICAD Degradation)**	-0.04569	-0.06711	-0.06038	0.049535
**CD40 Main Pathway**	-0.01853	-0.01295	-0.00457	0.049535
**CD40 Pathway (Cell Survival)**	0	0	0	1
**CD40 Pathway (Gene Expression)**	0	0	0	1
**CD40 Pathway (IKBs Degradation)**	0	0	0	1
**Cellular Anti Apoptosis Main Pathway**	0.044434	0.039626	0.035723	0.049535
**Cellular Anti Apoptosis Pathway (Apoptosis)**	0.079136	0.107641	0.08539	0.049535
**Cellular Anti Apoptosis Pathway (Depolarization)**	0	0	0	1

**Chemokine Main Pathway**	-0.00714	-0.00796	-0.00233	0.049535
**Chemokine Pathway (Cell Activation)**	0	0	0	1
**Chemokine Pathway (Gene Expression, Apoptosis)**	0	0	0	1
**Chemokine Pathway (Internalization, Degradation, Recycling)**	0.001092	0.011329	0.014594	0.049535
**Chromatin Main Pathway**	0	0	0	1
**Chromatin Pathway (Octamer Sliding)**	0	0	0	1
**Chromatin Pathway (Octamer Transfer)**	0	0	0	1
**Circadian Main Pathway**	0.219417	0.158016	-0.05261	0.512691
**CREB Main Pathway**	0.022803	0.020152	0.017042	0.049535
**CREB Pathway (Gene Expression Pathway)**	0.050556	0.055435	0.055907	0.049535
**Cytokine Main Pathway**	-0.05123	-0.04104	-0.04014	0.049535
**DDR pathway Apoptosis**	0	0	0	1
**DDR Main pathway**	0	0	0	1
**DNA Repair Mechanisms Pathway**	0.020333	0.023368	0.020084	0.049535
**EGFR Main Pathway**	0.000471	0.004052	0.001452	0.049535
**ErbB Family Main Pathway**	0.024199	0.031154	0.040477	0.049535
**ErbB Family Pathway (Anti-Apoptosis)**	0	0	0	1
**ERK Signaling Main Pathway**	0.031071	0.030352	0.026191	0.049535
**Erythropoeitin Main Pathway**	-0.00696	-0.01057	-0.00356	0.049535
**Estrogen Main Pathway**	0.036898	0.031886	0.030095	0.049535
**Fas Signaling Pathway (Negative)**	-0.00279	0.005367	0.011673	0.512691
**Fas Signaling Pathway (Positive)**	0	0	0	1
**FLT3 Main Pathway**	0.000476	0.012189	0.009732	0.049535
**Glucocorticoid Receptor Main Pathway**	0.013387	0.024265	0.023478	0.049535
**Glucocorticoid Receptor Pathway (Cell cycle arrest)**	0.613366	0.733299	0.853326	0.049535
**Glucocorticoid Receptor Pathway (Cell cycle progression)**	0.296237	0.413954	0.344374	0.049535
**Glucocorticoid Receptor Pathway (Gene expression)**	0.01764	0.030229	0.02886	0.049535
**Glucocorticoid Receptor Pathway (Inflammatory cytokines)**	-0.00559	0.001312	-0.00058	0.827259
**Glucocorticoid Receptor Pathway (SMAD signaling)**	0	0	0	1
**GPCR Main Pathway**	0.026606	0.021442	0.020408	0.049535
**GPCR Pathway (Gene expression)**	-0.02363	-0.02597	-0.01762	0.049535
**Growth Hormone Main Pathway**	0.009098	0.02565	0.027619	0.049535
**Growth Hormone Pathway (Cell survival)**	0	0	0	1
**Growth Hormone Pathway (Gene expression)**	0.033787	0.064979	0.060064	0.049535
**Growth Hormone Pathway (Glucose uptake)**	0.007815	0.022033	0.023724	0.049535
**Growth Hormone Pathway (Protein synthesis)**	0	0	0	1
**GSK3 Main Pathway**	0.04643	0.041312	0.041725	0.049535
**GSK3 Pathway (Degradation)**	0.046062	0.044976	0.043659	0.049535
**GSK3 Pathway (Gene expression)**	0.03351	0.062417	0.071762	0.049535
**GSK3 Pathway (Translation)**	0	0	0	1
**G-protein Pathway (Ras family GTPases)**	0.054337	0.079647	0.042558	0.049535
**Hedgehog Main Pathway**	-0.04514	-0.1443	-0.07402	0.049535
**Hedgehog Pathway (Repression of Hh, BMP)**	0.179169	0.169793	0.154752	0.049535
**Hedgehog Pathway (Activation of BMP, Ptc, WNT)**	0	0	0	1
**HGF Main Pathway**	0.047325	0.065486	0.056166	0.049535
**HGF Pathway (Anoikis)**	0.090171	0.092669	0.076962	0.049535
**HGF Pathway (Cell adhesion, cell mirgation)**	0.044128	0.091163	0.080549	0.049535
**HGF Pathway (Cell cycle progression)**	0.129848	0.131766	0.104326	0.049535
**HGF Pathway (Cell polarity, cell motility)**	0	0	0	1
**HGF Pathway (Cell scattering)**	0.093983	0.116244	0.101129	0.049535
**HGF Pathway (Cell survival)**	0.093983	0.116244	0.101129	0.049535

**HGF Pathway (IP3 pathway)**	0.108081	0.133681	0.116298	0.049535
**HGF Pathway (PKC pathway)**	0	0	0	1
**HIF1-Alpha Main Pathway**	-0.02187	-0.02977	-0.02513	0.049535
**HIF1Alpha Pathway (Gene expression)**	0.085314	0.088471	0.053157	0.049535
**HIF1Alpha Pathway (HIF1alpha degradation)**	0	0	0	1
**HIF1Alpha Pathway (NOS pathway)**	0.098439	0.102082	0.061335	0.049535
**HIF1Alpha Pathway (p53 Hypoxia pathway)**	0	0	0	1
**HIF1Alpha Pathway (Pyruvate)**	0.085314	0.088471	0.053157	0.049535
**HIF1Alpha Pathway (VEGF pathway)**	0.02662	0.009905	-0.00993	0.512691
**Hypoxia pathway EMT 1**	0.90899	0.935941	0.773995	0.049535
**Hypoxia pathway EMT 2**	0.90899	0.935941	0.773995	0.049535
**Hypoxia pathway EMT 3**	0.90899	0.935941	0.773995	0.049535
**Hypoxia pathway EMT 4**	0.90899	0.935941	0.773995	0.049535
**IGF1R Main Pathway**	-0.00737	-0.00246	-0.00551	0.049535
**IGF1R Signaling Pathway (Cell survival)**	0	0	0	1
**IGF1R Signaling Pathway (Glucose uptake)**	0	0	0	1
**IGF1R Signaling Pathway (Glycogen synthesis)**	0	0	0	1
**IGF1R Signaling Pathway (IKB degradation)**	0	0	0	1
**IGF1R Signaling Pathway (Protein synthesis)**	0	0	0	1
**ILK Main Pathway**	0.094929	0.092468	0.074222	0.049535
**ILK Pathway (Apoptosis)**	0.157373	0.18198	0.139049	0.049535
**ILK Pathway (Cell adhesion, cell motility, opsonization)**	0.157555	0.181247	0.137357	0.049535
**ILK Pathway (Cell cycle proliferation)**	0.146101	0.170681	0.13035	0.049535
**ILK Pathway (Cell migration, retraction)**	0.155054	0.17837	0.135177	0.049535
**ILK Pathway (Cell motility)**	0.147317	0.160445	0.12799	0.049535
**ILK Pathway (Cytoskeletal reorganization)**	0.167781	0.184446	0.141182	0.049535
**ILK Pathway (G2-phase arrest)**	0.146101	0.170681	0.13035	0.049535
**ILK Pathway (Induced cell proliferation)**	0.107279	0.099773	0.082334	0.049535
**ILK Pathway (Regulation of intermediate filaments)**	0.156294	0.179797	0.136259	0.049535
**ILK Pathway (Regulation of junction assembly of desmosomes)**	0.156294	0.179797	0.136259	0.049535
**ILK Pathway (Wound healing)**	0.168755	0.190295	0.144129	0.049535
**IL-10 Main Pathway**	0.026642	0.023353	0.040201	0.049535
**IL-10 Pathway (Gene expression)**	0.050638	0.06335	0.059886	0.049535
**IL-10 Pathway (Stability determination)**	0.810215	1.013598	0.958183	0.049535
**IL-10 Pathway (Translational modulation)**	-0.07969	-0.07524	-0.02399	0.049535
**IL-2 Main Pathway**	-0.01141	-0.01101	-0.00609	0.049535
**IL-2 Pathway (Actin reorganization)**	0	0	0	1
**IL-2 Pathway (Apoptosis)**	0.039069	0.067465	0.059752	0.049535
**IL-2 Pathway (Apoptosis inhibition)**	0.0329	0.056812	0.050318	0.049535
**IL-2 Pathway (Protein synthesis)**	0	0	0	1
**IL-6 Main Pathway**	0.0316	0.034346	0.028582	0.049535
**Integrin SIgnaling Main Pathway**	0.072077	0.082544	0.06238	0.049535
**Integrin SIgnaling Pathway (Cell survival)**	-0.00455	0.013069	0.018409	0.512691
**Integrin SIgnaling Pathway (Cytoskeleton contraction integrin modulation cell invasion and migration)**	-0.02208	-0.03027	-0.02083	0.049535
**Integrin SIgnaling Pathway (Focal adhesion and stress fibers)**	0	0	0	1
**Integrin SIgnaling Pathway (Translocation to the nucleus)**	0	0	0	1
**Interactions Report**	0.818091	0.842347	0.696595	0.049535
**Interferon Main Pathway**	0.006763	0.014075	0.014053	0.049535
**Interferon Pathway (Gene expression)**	0	0	0	1
**Interferon Pathway (Transcription)**	0	0	0	1

**Interferon Pathway (Translation)**	0	0	0	1
**IP3 Main Pathway**	-0.00328	-0.00329	-0.00898	0.049535
**IP3 Pathway (Gene expression)**	0	0	0	1
**JAK mStat Main Pathway**	-0.01394	-0.01739	-0.00646	0.049535
**JAK mStat Pathway (Akt pathway)**	0.004527	-0.00019	0.000417	0.512691
**JAK mStat Pathway (JAK degradation)**	-0.03992	-0.03898	-0.03784	0.049535
**JNK Main Pathway**	0.018537	0.011788	0.008844	0.049535
**JNK Pathway (Apoptosis, Inflammation, Tumorigenesis, Cell Migration)**	0.038005	0.042919	0.024783	0.049535
**JNK Pathway (Insulin signaling)**	0.003864	0.006662	0.004707	0.512691
**MAPK Family Main Pathway**	0.021246	0.022949	0.015639	0.049535
**MAPK Family Pathway (Chromatin Remodeling)**	0	0	0	1
**MAPK Family Pathway (Cytoskeleton)**	0	0	0	1
**MAPK Family Pathway (Gene Expression)**	-0.04121	-0.04528	-0.03072	0.049535
**MAPK Family Pathway (IKBs Degradation)**	0	0	0	1
**MAPK Family Pathway (Translation)**	0	0	0	1
**MAPK Signaling Main Pathway**	0.046954	0.050053	0.039358	0.049535
**MAPK Signaling Pathway (Cell Survival, Inflammation, Apoptosis, Osmoregulation)**	0.055819	0.063037	0.0364	0.049535
**MAPK Signaling Pathway (Gene Expression)**	0.046009	0.052239	0.034835	0.049535
**DDR pathway (MMR)**	0	0	0	1
**Mismatch Repair Main Pathway**	0	0	0	1
**Mitochondrial Apopotosis Main Pathway**	-0.06359	-0.05931	-0.04925	0.049535
**Mitochondrial Apopotosis Pathway (Apoptosis)**	-0.05784	-0.06957	-0.05148	0.049535
**Mitochondrial Apopotosis Pathway (Depolarization)**	0	0	0	1
**Mitochondrial Apopotosis Pathway (DNA fragmentation)**	0	0	0	1
**Mitochondrial Apopotosis Pathway (Gene expression)**	0	0	0	1
**mTOR Main Pathway**	0.024678	0.016309	0.018182	0.049535
**mTOR Pathway (Actin organization)**	-0.03123	-0.03983	-0.02712	0.049535
**mTOR Pathway (Akt signaling)**	0	0	0	1
**mTOR Pathway (Scanning)**	0	0	0	1
**mTOR Pathway (Translation on)**	0	0	0	1
**mTOR Pathway (VEGF pathway)**	0	0	0	1
**NGF (Negative) Main Pathway**	0	0	0	1
**NGF (Negative) Pathway (Apoptosis)**	0	0	0	1
**NGF (Positive) Main Pathway**	0.036912	0.047195	0.037257	0.049535
**NHEJ mechanisms of DSBs repair effect**	0	0	0	1
**Notch Main Pathway**	-0.04792	-0.08212	-0.0784	0.049535
**DDR Pathway (NER)**	0	0	0	1
**p38 (Negative) Main Signaling Pathway**	0.016658	0.007807	0.01317	0.049535
**p38 (Positive) Main Signaling Pathway**	0.016625	0.007791	0.013143	0.049535
**p53 Signaling (Negative) Main Pathway**	0.028123	0.033866	0.02047	0.049535
**p53 Signaling (Negative) Pathway (p53 Degradation)**	-0.04606	-0.04498	-0.04366	0.049535
**PAK Main Pathway**	0.049057	0.043208	0.039318	0.049535
**PAK Pathway (Actin Cytoskeleton)**	0.054098	0.055296	0.044393	0.049535
**PAK Pathway (Myosin Activation)**	0.060102	0.055587	0.053932	0.049535
**PPAR Main Pathway**	0.017985	0.02127	0.023131	0.049535
**PTEN Main Pathway**	-0.02791	-0.03573	-0.03	0.049535
**RANK Signaling in Osteoclast Main Pathway**	0.061995	0.058967	0.051696	0.049535
**RANK Signaling in Osteoclast Pathway (IKBs Degradation)**	0	0	0	1
**RAS Main Pathway**	-0.0013	0.005781	0.007307	0.275234
**Telomere Main Pathway**	-0.03388	-0.06747	-0.03937	0.049535

**RNA Polymerase II Complex Pathway**	-0.03243	-0.04279	-0.03068	0.049535
**Cell Cycle Pathway (SCC during S-phase)**	0	0	0	1
**SMAD (Negative) Main Pathway**	0.132269	0.130497	0.109576	0.049535
**SMAD (Negative) Pathway (Degradation)**	0.030973	0.030243	0.029357	0.049535
**SMAD (Positive) Main Pathway**	0.132269	0.130497	0.109576	0.049535
**SMAD (Positive) Pathway (Degradation)**	0.030973	0.030243	0.029357	0.049535
**Cell Cycle Pathway (Metaphase-Anaphase)**	0.080622	0.075465	0.06424	0.049535
**Cell Cycle Pathway (Origin of S-phase)**	-0.0027	0.013142	0.022545	0.512691
**STAT3 Main Pathway**	0.027185	0.025415	0.028889	0.049535
**TGF beta Main Pathway**	0.019475	0.015002	0.015212	0.049535
**TGF beta Pathway (Epithelial mesehchymal transition)**	0.175988	0.175629	0.132742	0.049535
**TGF beta Pathway (Post-transcriptional G1 arrest)**	0.108747	0.090301	0.062643	0.049535
**TGF beta Pathway (SnON degradation)**	0	0	0	1
**TGF beta Pathway (Tumorigenesis)**	0	0	0	1
**TGF beta Pathway (Tumor suppression)**	0	0	0	1
**TNF (Negative) Main Pathway**	-0.00573	0.011017	0.023959	0.512691
**TNF (Negative) Pathway (Apoptosis)**	-0.00641	0.012313	0.026778	0.512691
**TNF (Positive) Main Pathway**	0	0	0	1
**TNF (Positive) Pathway (Gene expression, Cell survival)**	0	0	0	1
**TNF (Positive) Pathway (IKBs degradation)**	0	0	0	1
**TRAF (Negative) Main Pathway**	0	0	0	1
**TRAF (Negative) Pathway (IKBs Degradation)**	0	0	0	1
**TRAF (Positive) Main Pathway**	-0.0217	-0.02361	-0.01158	0.049535
**TRAF (Positive) Pathway (IKBs Degradation)**	0	0	0	1
**Transcription of mRNA Pathway**	-0.03784	-0.04992	-0.03579	0.049535
**Cell Cycle Pathway (End of S-phase)**	0.041547	0.037657	0.03481	0.049535
**Translation Regulation of EIF4F activity**	-0.0328	-0.03604	-0.02445	0.049535
**Ubiquitin Proteasome Main Pathway**	0	0	0	1
**Ubiquitin Proteasome Pathway (Degraded Protein)**	0	0	0	1
**VEGF Main Pathway**	0.066299	0.056145	0.051296	0.049535
**VEGF Pathway (Actin Reorganization)**	0.204945	0.206975	0.171885	0.049535
**VEGF Pathway (Nitric Oxide Production)**	0	0	0	1
**Wnt Main Pathway**	0.052537	0.050437	0.046679	0.049535
**Wnt Pathway (Ctnn-b Degradation)**	-0.05133	-0.05012	-0.04865	0.049535
**IL-6 Pathway (IKBs degradation)**	0	0	0	1

**Pathway**	**M_68_32h_1**	**M_68_32h_2**	**M_68_32h_3**	**p-value_Mea[illegible]**
**AHR Main Pathway**	0.044304	0.070361	0.06288	0.049535
**AHR Pathway (AHR Degradation)**	0	0	0	1
**AHR Pathway (Cath-D Expression)**	0	0	0	1
**AHR Pathway (C-MycExpression)**	0	0	0	1
**AHR Pathway (PS2 Gene Expression)**	0	0	0	1
**AKT Main Pathway**	0.068023	0.058516	0.046349	0.049535
**AKT Pathway (Aggregation & Neurodegeneration)**	0.048402	0.046428	0.035218	0.049535
**AKT Pathway (Apoptosis Inhibition)**	0.046504	0.044607	0.033837	0.049535

AKT Pathway (Blocks Apoptosis)	0.046466	0.046524	0.038414	0.049535
AKT Pathway (Cardiovascular Homeostasis)	0.052704	0.050555	0.038348	0.049535
AKT Pathway (Caspase Cascade)	0.0328	0.03284	0.027116	0.049535
AKT Pathway (Cell Cycle)	0.349805	0.325805	0.280421	0.049535
AKT Pathway (Cell Cycle Progression)	-0.03525	-0.0545	-0.04996	0.049535
AKT Pathway (Cell Survival)	0.185865	0.186096	0.153656	0.049535
AKT Pathway (Death Genes)	0.030978	0.031016	0.025609	0.049535
AKT Pathway (Elevation of Glucose Import)	0.029347	0.029384	0.024261	0.049535
AKT Pathway (ERK Pathway)	0.046466	0.046524	0.038414	0.049535
AKT Pathway (Genetic Stability)	-0.03869	-0.06175	-0.0626	0.049535
AKT Pathway (Glucose Uptake)	0.053902	0.051704	0.03922	0.049535
AKT Pathway (Glycogen Synthesis)	0.159313	0.159511	0.131705	0.049535
AKT Pathway (Insulin Stimulated Mitogenesis)	0.052704	0.050555	0.038348	0.049535
AKT Pathway (JNK Pathway)	0	0	0	1
AKT Pathway (Neuroprotection)	0.052704	0.050555	0.038348	0.049535
AKT Pathway (NF-kB Pathway)	0.04941	0.047395	0.035951	0.049535
AKT Pathway (p53 Degradation)	0.139399	0.139572	0.115242	0.049535
AKT Pathway (p73 Mediated Apoptosis)	0.031863	0.031902	0.026341	0.049535
AKT Pathway (Protein Synthesis)	0	0	0	1
AKT Pathway (Regeneration of Cyclic Nucleotide)	0.028595	0.02863	0.023639	0.049535
AKT Pathway (Respiratory Burst)	0.052704	0.050555	0.038348	0.049535
AKT Pathway (Survival Genes)	0.041303	0.041355	0.034146	0.049535
AKT Pathway (Synaptic Signaling)	0.023727	0.023757	0.019616	0.049535
AKT Pathway (Translation)	0	0	0	1
Androgen Receptor Pathway	0.079438	0.078274	0.051179	0.049535
Androgen Receptor Pathway (Apoptosis)	0.160485	0.166826	0.105153	0.049535
Androgen Receptor Pathway (Degradation)	0.034401	0.017718	0.007185	0.12663
Androgen Receptor Pathway (Cell Survival & Cell Growth)	0	0	0	1
Androgen Receptor Pathway (Gonadotropin Regulation)	0.027213	0.059472	0.035748	0.049535
Androgen Receptor Pathway (Histone Modification)	0.027213	0.059472	0.035748	0.049535
Androgen Receptor Pathway (Prostate Differentiation & Development)	0.027213	0.059472	0.035748	0.049535
Androgen Receptor Pathway (Sexual Differentiation & Sexual Maturation at Puberty)	0.027213	0.059472	0.035748	0.049535
ATM Main Pathway	0.108366	0.158694	0.121106	0.049535
ATM Pathway (Apoptosis)	0	0	0	1
ATM Pathway (Apoptosis, Senescense)	0.097647	0.182283	0.15187	0.049535
ATM Pathway (Cell Cicle Checkpoint Control)	0.130483	0.240559	0.210975	0.049535
ATM Pathway (Cell Survival)	0.113483	0.13238	0.070325	0.049535
ATM Pathway (Checkpoint Activation)	0.130483	0.240559	0.210975	0.049535
ATM Pathway (DNA Repair)	0.39345	0.633368	0.55218	0.049535
ATM Pathway (G2_M Checkpoint Arrest)	-0.54987	-0.74113	-0.66289	0.049535
ATM Pathway (G2 Mitosis Progression)	-1.55066	-2.17181	-1.96092	0.049535
ATM Pathway (MDMX Ubiquitination, Degradation)	-0.01331	-0.02182	-0.02873	0.049535
ATM Pathway (NF-kB Pathway)	0.130483	0.240559	0.210975	0.049535
ATM Pathway (Synaptic Vesicle Transport)	0.130483	0.240559	0.210975	0.049535
ATM Pathway (S-Phase Arrest)	0.832106	1.130864	1.025136	0.049535
ATM Pathway (S-Phase Progression)	-0.17854	-0.20008	-0.20586	0.049535
DDR Pathway (BRCA1-induced responses)	-0.04303	0.001146	5.71E-05	0.512691
BRCA1 Main Pathway	0.263717	0.318565	0.294741	0.049535
cAMP Main Pathway	0.084825	0.070865	0.053961	0.049535
cAMP Pathway (Axonal Growth)	-0.04437	-0.02311	-0.03264	0.049535

**cAMP Pathway (Cardiovascular Homeostasis)**	-0.31371	-0.24367	-0.18523	0.049535
**cAMP Pathway (Cell Growth)**	-0.51013	-0.45301	-0.26898	0.049535
**cAMP Pathway (Cell Proliferation)**	-0.1024	-0.05333	-0.07533	0.049535
**cAMP Pathway (Cell Survival)**	0.02657	0.036737	0.016018	0.049535
**cAMP Pathway (Cell Survival, Chemotaxis)**	-0.38259	-0.33976	-0.20174	0.049535
**cAMP Pathway (Cytokine Production)**	-0.06865	-0.08231	-0.11528	0.049535
**cAMP Pathway (Degradation of Cell Cycle Regulators)**	-0.04294	-0.02236	-0.03159	0.049535
**cAMP Pathway (Endothelial Cell Regulation)**	0.203895	0.170311	0.098425	0.049535
**cAMP Pathway (Glycogen Synthesis)**	-0.12102	-0.06303	-0.08903	0.049535
**cAMP Pathway (Glycolysis)**	0.088632	0.061197	0.013579	0.12663
**cAMP Pathway (Metabolic Energy)**	0.074346	0.074438	0.061462	0.049535
**cAMP Pathway (Myocardial Contraction)**	0.032085	0.042931	0.028381	0.049535
**cAMP Pathway (Oncogenesis)**	-0.09508	-0.04952	-0.06995	0.049535
**cAMP Pathway (Protein Retention)**	0.12457	0.171791	0.117245	0.049535
**cAMP Pathway (Regulation of Cytoskeleton)**	-0.0832	-0.04333	-0.06121	0.049535
**Caspase Cascade Main**	-0.08424	-0.07551	-0.05695	0.049535
**Caspase Cascade (Activated Tissue Transglutaminase)**	0.042844	0.056327	0.040117	0.049535
**Caspase Cascade (Apoptosis)**	-0.01407	-0.01648	-0.0195	0.049535
**Caspase Cascade (Cell Survival)**	-0.15045	-0.1564	-0.09858	0.049535
**Caspase Cascade (ICAD Degradation)**	0.035154	0.047173	0.035523	0.049535
**CD40 Main Pathway**	0.041351	0.03102	0.013252	0.049535
**CD40 Pathway (Cell Survival)**	0	0	0	1
**CD40 Pathway (Gene Expression)**	0.09515	0.071764	0.05853	0.049535
**CD40 Pathway (IKBs Degradation)**	0.067165	0.050657	0.041315	0.049535
**Cellular Anti Apoptosis Main Pathway**	0.095377	0.081195	0.066982	0.049535
**Cellular Anti Apoptosis Pathway (Apoptosis)**	0.017718	0.036025	0.030614	0.049535
**Cellular Anti Apoptosis Pathway (Depolarization)**	0	0	0	1
**Chemokine Main Pathway**	0.056917	0.061855	0.053113	0.049535
**Chemokine Pathway (Cell Activation)**	0.036201	0.026997	0	0.19043
**Chemokine Pathway (Gene Expression, Apoptosis)**	0.045645	0.034039	0	0.19043
**Chemokine Pathway (Internalization, Degradation, Recycling)**	0	0	0	1
**Chromatin Main Pathway**	0.156614	0.23993	0.208066	0.049535
**Chromatin Pathway (Octamer Sliding)**	0.180709	0.276843	0.240076	0.049535
**Chromatin Pathway (Octamer Transfer)**	0.156614	0.23993	0.208066	0.049535
**Circadian Main Pathway**	0	0	0	1
**CREB Main Pathway**	0.089458	0.080743	0.060982	0.049535
**CREB Pathway (Gene Expression Pathway)**	0	0	0	1
**Cytokine Main Pathway**	0	0	0	1
**DDR pathway Apoptosis**	0.956957	1.235703	1.11903	0.049535
**DDR Main pathway**	0.680211	0.970477	0.879195	0.049535
**DNA Repair Mechanisms Pathway**	0.230537	0.282172	0.264534	0.049535
**EGFR Main Pathway**	0.051502	0.041716	0.032197	0.049535
**ErbB Family Main Pathway**	0.031228	0.031927	0.024515	0.049535
**ErbB Family Pathway (Anti-Apoptosis)**	0	0	0	1
**ERK Signaling Main Pathway**	0.123194	0.11842	0.09125	0.049535
**Erythropoeitin Main Pathway**	0.018502	0.014324	0.007803	0.049535
**Estrogen Main Pathway**	0.103491	0.088829	0.066817	0.049535
**Fas Signaling Pathway (Negative)**	0.108783	0.134434	0.11121	0.049535
**Fas Signaling Pathway (Positive)**	0	0	0	1
**FLT3 Main Pathway**	0.067021	0.067222	0.039176	0.049535
**Glucocorticoid Receptor Main Pathway**	0.060963	0.076794	0.058104	0.049535

**Glucocorticoid Receptor Pathway (Cell cycle arrest)**	0.610874	0.895908	0.810402	0.049535
**Glucocorticoid Receptor Pathway (Cell cycle progression)**	0	0	0	1
**Glucocorticoid Receptor Pathway (Gene expression)**	0.052926	0.072764	0.061717	0.049535
**Glucocorticoid Receptor Pathway (Inflammatory cytokines)**	0.043616	0.053758	0.034862	0.049535
**Glucocorticoid Receptor Pathway (SMAD signaling)**	0.115254	0.092651	0.074199	0.049535
**GPCR Main Pathway**	0.0957	0.082866	0.061485	0.049535
**GPCR Pathway (Gene expression)**	0.03223	0.024178	0.010329	0.049535
**Growth Hormone Main Pathway**	0.000577	0.002698	-0.00806	0.512691
**Growth Hormone Pathway (Cell survival)**	0	0	0	1
**Growth Hormone Pathway (Gene expression)**	0.0009	0.004204	-0.01256	0.512691
**Growth Hormone Pathway (Glucose uptake)**	0.000496	0.002318	-0.00692	0.512691
**Growth Hormone Pathway (Protein synthesis)**	0	0	0	1
**GSK3 Main Pathway**	0.090052	0.078655	0.058726	0.049535
**GSK3 Pathway (Degradation)**	0	0	0	1
**GSK3 Pathway (Gene expression)**	-0.04678	-0.01737	-0.00179	0.275234
**GSK3 Pathway (Translation)**	0	0	0	1
**G-protein Pathway (Ras family GTPases)**	0.05576	0.055829	0.046097	0.049535
**Hedgehog Main Pathway**	0.248696	0.29425	0.241068	0.049535
**Hedgehog Pathway (Repression of Hh, BMP)**	0.549349	0.511273	0.442021	0.049535
**Hedgehog Pathway (Activation of BMP, Ptc, WNT)**	0	0	0	1
**HGF Main Pathway**	0.072948	0.066795	0.053112	0.049535
**HGF Pathway (Anoikis)**	0.177068	0.164957	0.137647	0.049535
**HGF Pathway (Cell adhesion, cell mirgation)**	0.032325	0.026951	0.015353	0.049535
**HGF Pathway (Cell cycle progression)**	0.197913	0.1918	0.175661	0.049535
**HGF Pathway (Cell polarity, cell motility)**	0	0	0	1
**HGF Pathway (Cell scattering)**	0.153515	0.140109	0.110803	0.049535
**HGF Pathway (Cell survival)**	0.153515	0.140109	0.110803	0.049535
**HGF Pathway (IP3 pathway)**	0.120782	0.105297	0.081326	0.049535
**HGF Pathway (PKC pathway)**	0	0	0	1
**HIF1-Alpha Main Pathway**	0	0	0	1
**HIF1Alpha Pathway (Gene expression)**	0	0	0	1
**HIF1Alpha Pathway (HIF1alpha degradation)**	0	0	0	1
**HIF1Alpha Pathway (NOS pathway)**	0	0	0	1
**HIF1Alpha Pathway (p53 Hypoxia pathway)**	0	0	0	1
**HIF1Alpha Pathway (Pyruvate)**	0	0	0	1
**HIF1Alpha Pathway (VEGF pathway)**	0	0	0	1
**Hypoxia pathway EMT 1**	0.779946	0.650166	0.617522	0.049535
**Hypoxia pathway EMT 2**	0.779946	0.650166	0.617522	0.049535
**Hypoxia pathway EMT 3**	0.779946	0.650166	0.617522	0.049535
**Hypoxia pathway EMT 4**	0.779946	0.650166	0.617522	0.049535
**IGF1R Main Pathway**	0.027653	0.030292	0.020624	0.049535
**IGF1R Signaling Pathway (Cell survival)**	0.010666	0.005973	0.003406	0.512691
**IGF1R Signaling Pathway (Glucose uptake)**	0.223038	0.223315	0.184387	0.049535
**IGF1R Signaling Pathway (Glycogen synthesis)**	0	0	0	1
**IGF1R Signaling Pathway (IKB degradation)**	0.150466	0.131849	0.108286	0.049535
**IGF1R Signaling Pathway (Protein synthesis)**	0	0	0	1
**ILK Main Pathway**	0.186112	0.175056	0.136778	0.049535
**ILK Pathway (Apoptosis)**	0.293043	0.278309	0.208281	0.049535
**ILK Pathway (Cell adhesion, cell motility, opsonization)**	0.314312	0.302423	0.226038	0.049535
**ILK Pathway (Cell cycle proliferation)**	0.282425	0.271742	0.203106	0.049535
**ILK Pathway (Cell migration, retraction)**	0.309323	0.297622	0.22245	0.049535

**ILK Pathway (Cell motility)**	0.274823	0.263703	0.19557	0.049535
**ILK Pathway (Cytoskeletal reorganization)**	0.306546	0.292639	0.219317	0.049535
**ILK Pathway (G2-phase arrest)**	0.282425	0.271742	0.203106	0.049535
**ILK Pathway (Induced cell proliferation)**	0.10247	0.149207	0.14004	0.049535
**ILK Pathway (Regulation of intermediate filaments)**	0.311797	0.300003	0.224229	0.049535
**ILK Pathway (Regulation of junction assembly of desmosomes)**	0.311797	0.300003	0.224229	0.049535
**ILK Pathway (Wound healing)**	0.326419	0.312925	0.233527	0.049535
**IL-10 Main Pathway**	0.137197	0.120609	0.086914	0.049535
**IL-10 Pathway (Gene expression)**	0.136977	0.102754	0.043897	0.049535
**IL-10 Pathway (Stability determination)**	0	0	0	1
**IL-10 Pathway (Translational modulation)**	0.104982	0.078291	0	0.19043
**IL-2 Main Pathway**	0.022896	0.022484	0.012082	0.049535
**IL-2 Pathway (Actin reorganization)**	0.133953	0.15138	0.096688	0.049535
**IL-2 Pathway (Apoptosis)**	0	0	0	1
**IL-2 Pathway (Apoptosis inhibition)**	0	0	0	1
**IL-2 Pathway (Protein synthesis)**	0	0	0	1
**IL-6 Main Pathway**	0.056685	0.052962	0.048649	0.049535
**Integrin SIgnaling Main Pathway**	0.182378	0.180013	0.130216	0.049535
**Integrin SIgnaling Pathway (Cell survival)**	0.012169	0.010146	0.00578	0.049535
**Integrin SIgnaling Pathway (Cytoskeleton contraction integrin modulation cell invasion and migration)**	0	0	0	1
**Integrin SIgnaling Pathway (Focal adhesion and stress fibers)**	0	0	0	1
**Integrin SIgnaling Pathway (Translocation to the nucleus)**	0.117059	0.174666	0.066859	0.049535
**Interactions Report**	0.701951	0.585149	0.55577	0.049535
**Interferon Main Pathway**	0.011411	0.00851	0	0.19043
**Interferon Pathway (Gene expression)**	0.052491	0.039145	0	0.19043
**Interferon Pathway (Transcription)**	0	0	0	1
**Interferon Pathway (Translation)**	0	0	0	1
**IP3 Main Pathway**	0.040438	0.041617	0.029966	0.049535
**IP3 Pathway (Gene expression)**	0	0	0	1
**JAK mStat Main Pathway**	0.140869	0.146567	0.135404	0.049535
**JAK mStat Pathway (Akt pathway)**	0.10224	0.087417	0.060864	0.049535
**JAK mStat Pathway (JAK degradation)**	0	0	0	1
**JNK Main Pathway**	0.095342	0.094603	0.071835	0.049535
**JNK Pathway (Apoptosis, Inflammation, Tumorigenesis, Cell Migration)**	0.091386	0.137704	0.09043	0.049535
**JNK Pathway (Insulin signaling)**	0	0	0	1
**MAPK Family Main Pathway**	0.034133	0.032587	0.023371	0.049535
**MAPK Family Pathway (Chromatin Remodeling)**	0.036201	0.026997	0	0.19043
**MAPK Family Pathway (Cytoskeleton)**	0.069988	0.052194	0	0.19043
**MAPK Family Pathway (Gene Expression)**	0.056196	0.042156	0.018009	0.049535
**MAPK Family Pathway (IKBs Degradation)**	0.087831	0.066243	0.054028	0.049535
**MAPK Family Pathway (Translation)**	0	0	0	1
**MAPK Signaling Main Pathway**	0.136838	0.131473	0.09669	0.049535
**MAPK Signaling Pathway (Cell Survival, Inflammation, Apoptosis, Osmoregulation)**	0.027238	0.076614	0.067388	0.049535
**MAPK Signaling Pathway (Gene Expression)**	0.043514	0.062786	0.050534	0.049535
**DDR pathway (MMR)**	0.606022	0.759544	0.678561	0.049535
**Mismatch Repair Main Pathway**	0.979397	1.184456	1.049507	0.049535
**Mitochondrial Apopotosis Main Pathway**	-0.08159	-0.07573	-0.05811	0.049535
**Mitochondrial Apopotosis Pathway (Apoptosis)**	0.047248	0.096067	0.081638	0.049535
**Mitochondrial Apopotosis Pathway (Depolarization)**	0	0	0	1
**Mitochondrial Apopotosis Pathway (DNA fragmentation)**	-0.21535	-0.23097	-0.10923	0.049535

**Mitochondrial Apopotosis Pathway (Gene expression)**	-0.23518	-0.38425	-0.32805	0.049535
**mTOR Main Pathway**	0.073795	0.063215	0.052786	0.049535
**mTOR Pathway (Actin organization)**	0.031878	0.026146	0.02307	0.049535
**mTOR Pathway (Akt signaling)**	0	0	0	1
**mTOR Pathway (Scanning)**	0	0	0	1
**mTOR Pathway (Translation on)**	0	0	0	1
**mTOR Pathway (VEGF pathway)**	0	0	0	1
**NGF (Negative) Main Pathway**	0	0	0	1
**NGF (Negative) Pathway (Apoptosis)**	0	0	0	1
**NGF (Positive) Main Pathway**	0.035969	0.031041	0.028957	0.049535
**NHEJ mechanisms of DSBs repair effect**	0.280197	0.37238	0.333434	0.049535
**Notch Main Pathway**	0.018938	0.033894	0.026336	0.049535
**DDR Pathway (NER)**	0.365804	0.430157	0.380717	0.049535
**p38 (Negative) Main Signaling Pathway**	0.075149	0.067862	0.054343	0.049535
**p38 (Positive) Main Signaling Pathway**	0.074997	0.067724	0.054233	0.049535
**p53 Signaling (Negative) Main Pathway**	-0.02016	-0.01456	-0.01977	0.049535
**p53 Signaling (Negative) Pathway (p53 Degradation)**	0.022119	0.020388	0.020581	0.049535
**PAK Main Pathway**	0.115735	0.10123	0.082661	0.049535
**PAK Pathway (Actin Cytoskeleton)**	0.116109	0.109472	0.085051	0.049535
**PAK Pathway (Myosin Activation)**	0.148438	0.144202	0.10489	0.049535
**PPAR Main Pathway**	0.080236	0.072654	0.04646	0.049535
**PTEN Main Pathway**	-0.03943	-0.03831	-0.03545	0.049535
**RANK Signaling in Osteoclast Main Pathway**	0.095899	0.079299	0.049764	0.049535
**RANK Signaling in Osteoclast Pathway (IKBs Degradation)**	0.05709	0.043058	0.035118	0.049535
**RAS Main Pathway**	0.09821	0.104929	0.080294	0.049535
**Telomere Main Pathway**	0.046229	0.033663	0.032004	0.049535
**RNA Polymerase II Complex Pathway**	0.02386	0.025863	0.01788	0.049535
**Cell Cycle Pathway (SCC during S-phase)**	-0.1126	-0.18589	-0.15937	0.049535
**SMAD (Negative) Main Pathway**	0.090561	0.080142	0.063333	0.049535
**SMAD (Negative) Pathway (Degradation)**	0	0	0	1
**SMAD (Positive) Main Pathway**	0.090561	0.080142	0.063333	0.049535
**SMAD (Positive) Pathway (Degradation)**	0	0	0	1
**Cell Cycle Pathway (Metaphase-Anaphase)**	0.478544	0.588901	0.491488	0.049535
**Cell Cycle Pathway (Origin of S-phase)**	1.221171	1.402324	1.319554	0.049535
**STAT3 Main Pathway**	0.070363	0.077386	0.063382	0.049535
**TGF beta Main Pathway**	0.071568	0.068764	0.053216	0.049535
**TGF beta Pathway (Epithelial mesehchymal transition)**	0.063693	0.051202	0.041005	0.049535
**TGF beta Pathway (Post-transcriptional G1 arrest)**	0.048407	0.038913	0.031163	0.049535
**TGF beta Pathway (SnON degradation)**	0	0	0	1
**TGF beta Pathway (Tumorigenesis)**	0	0	0	1
**TGF beta Pathway (Tumor suppression)**	0	0	0	1
**TNF (Negative) Main Pathway**	0.081647	0.130191	0.103911	0.049535
**TNF (Negative) Pathway (Apoptosis)**	0.091253	0.145508	0.116136	0.049535
**TNF (Positive) Main Pathway**	0.046914	0.033739	0.02218	0.049535
**TNF (Positive) Pathway (Gene expression, Cell survival)**	0.079693	0.057715	0.059639	0.049535
**TNF (Positive) Pathway (IKBs degradation)**	0.063434	0.047842	0.03902	0.049535
**TRAF (Negative) Main Pathway**	0	0	0	1
**TRAF (Negative) Pathway (IKBs Degradation)**	0.063434	0.047842	0.03902	0.049535
**TRAF (Positive) Main Pathway**	0.025637	0.018491	0.005889	0.049535
**TRAF (Positive) Pathway (IKBs Degradation)**	0.063434	0.047842	0.03902	0.049535
**Transcription of mRNA Pathway**	0.027837	0.030174	0.02086	0.049535

Borger et al
Supplemental Data

**Cell Cycle Pathway (End of S-phase)**	0.229924	0.237727	0.208453	0.049535
**Translation Regulation of EIF4F activity**	0.047456	0.042641	0.034715	0.049535
**Ubiquitin Proteasome Main Pathway**	0.046428	0.064835	0.05796	0.049535
**Ubiquitin Proteasome Pathway (Degraded Protein)**	0.093096	0.128342	0.114635	0.049535
**VEGF Main Pathway**	0.115981	0.100023	0.068459	0.049535
**VEGF Pathway (Actin Reorganization)**	0.228337	0.19692	0.134779	0.049535
**VEGF Pathway (Nitric Oxide Production)**	0	0	0	1
**Wnt Main Pathway**	0.070375	0.075174	0.061155	0.049535
**Wnt Pathway (Ctnn-b Degradation)**	0	0	0	1
**IL-6 Pathway (IKBs degradation)**	0.09515	0.071764	0.05853	0.049535

**Pathway**	**M_68_48h_1**	**M_68_48h_2**	**M_68_48h_3**	**p-value_Mean**
**AHR Main Pathway**	0.036728	0.046444	0.026825	0.049535
**AHR Pathway (AHR Degradation)**	0	0	0	1
**AHR Pathway (Cath-D Expression)**	0	0	0	1
**AHR Pathway (C-MycExpression)**	0	0	0	1
**AHR Pathway (PS2 Gene Expression)**	0	0	0	1
**AKT Main Pathway**	0.054606	0.04757	0.066547	0.049535
**AKT Pathway (Aggregation & Neurodegeneration)**	0.023604	0.024499	0.020661	0.049535
**AKT Pathway (Apoptosis Inhibition)**	0.018598	0.015434	0.014777	0.049535
**AKT Pathway (Blocks Apoptosis)**	0	0	0	1
**AKT Pathway (Cardiovascular Homeostasis)**	0.04449	0.040949	0.038959	0.049535
**AKT Pathway (Caspase Cascade)**	0.033325	0.030838	0.025781	0.049535
**AKT Pathway (Cell Cycle)**	0.362676	0.357815	0.471927	0.049535
**AKT Pathway (Cell Cycle Progression)**	-0.02659	-0.01515	-0.01234	0.049535
**AKT Pathway (Cell Survival)**	0	0	0	1
**AKT Pathway (Death Genes)**	0.031474	0.029125	0.024349	0.049535
**AKT Pathway (Elevation of Glucose Import)**	0.029818	0.027592	0.023067	0.049535
**AKT Pathway (ERK Pathway)**	0	0	0	1
**AKT Pathway (Genetic Stability)**	-0.03275	-0.04434	-0.04164	0.049535
**AKT Pathway (Glucose Uptake)**	0.045501	0.04188	0.039844	0.049535
**AKT Pathway (Glycogen Synthesis)**	0	0	0	1
**AKT Pathway (Insulin Stimulated Mitogenesis)**	0.04449	0.040949	0.038959	0.049535
**AKT Pathway (JNK Pathway)**	0.031474	0.029125	0.024349	0.049535
**AKT Pathway (Neuroprotection)**	0.04449	0.040949	0.038959	0.049535
**AKT Pathway (NF-kB Pathway)**	0.041709	0.03839	0.036524	0.049535
**AKT Pathway (p53 Degradation)**	0	0	0	1
**AKT Pathway (p73 Mediated Apoptosis)**	0.032373	0.029957	0.025045	0.049535
**AKT Pathway (Protein Synthesis)**	0	0	0	1
**AKT Pathway (Regeneration of Cyclic Nucleotide)**	0.029053	0.026884	0.022476	0.049535
**AKT Pathway (Respiratory Burst)**	0.04449	0.040949	0.038959	0.049535
**AKT Pathway (Survival Genes)**	0	0	0	1
**AKT Pathway (Synaptic Signaling)**	0.024108	0.022308	0.01865	0.049535
**AKT Pathway (Translation)**	0	0	0	1
**Androgen Receptor Pathway**	0.032771	0.020346	0.03375	0.049535
**Androgen Receptor Pathway (Apoptosis)**	0.133481	0.121463	0.152766	0.049535
**Androgen Receptor Pathway (Degradation)**	-0.0187	-0.0177	-0.01808	0.049535
**Androgen Receptor Pathway (Cell Survival & Cell Growth)**	0.061718	0.063472	0.042461	0.049535
**Androgen Receptor Pathway (Gonadotropin Regulation)**	0.062013	0.075312	0.043257	0.049535

**Androgen Receptor Pathway (Histone Modification)**	0.062013	0.075312	0.043257	0.049535
**Androgen Receptor Pathway (Prostate Differentiation & Development)**	0.062013	0.075312	0.043257	0.049535
**Androgen Receptor Pathway (Sexual Differentiation & Sexual Maturation at Puberty)**	0.062013	0.075312	0.043257	0.049535
**ATM Main Pathway**	0.018054	-0.00069	0.028422	0.512691
**ATM Pathway (Apoptosis)**	0	0	0	1
**ATM Pathway (Apoptosis, Senescense)**	-0.1357	-0.14452	-0.10423	0.049535
**ATM Pathway (Cell Cicle Checkpoint Control)**	0.141175	0.184025	0.117486	0.049535
**ATM Pathway (Cell Survival)**	0.047058	0.061342	0.039162	0.049535
**ATM Pathway (Checkpoint Activation)**	0.141175	0.184025	0.117486	0.049535
**ATM Pathway (DNA Repair)**	0.766937	0.85886	0.667837	0.049535
**ATM Pathway (G2_M Checkpoint Arrest)**	-1.1773	-1.36111	-0.98292	0.049535
**ATM Pathway (G2 Mitosis Progression)**	-2.07466	-2.31119	-1.80782	0.049535
**ATM Pathway (MDMX Ubiquitination, Degradation)**	-0.30401	-0.35745	-0.24256	0.049535
**ATM Pathway (NF-kB Pathway)**	0.141175	0.184025	0.117486	0.049535
**ATM Pathway (Synaptic Vesicle Transport)**	0.141175	0.184025	0.117486	0.049535
**ATM Pathway (S-Phase Arrest)**	0.810177	0.897245	0.700733	0.049535
**ATM Pathway (S-Phase Progression)**	-0.07577	-0.08268	-0.01444	0.12663
**DDR Pathway (BRCA1-induced responses)**	-0.15049	-0.15019	-0.21674	0.049535
**BRCA1 Main Pathway**	0.24454	0.29364	0.234965	0.049535
**cAMP Main Pathway**	0.021889	0.015306	0.035501	0.049535
**cAMP Pathway (Axonal Growth)**	-0.16667	-0.16741	-0.1298	0.049535
**cAMP Pathway (Cardiovascular Homeostasis)**	-0.25756	-0.21943	-0.24139	0.049535
**cAMP Pathway (Cell Growth)**	-0.3568	-0.2805	-0.32916	0.049535
**cAMP Pathway (Cell Proliferation)**	-0.1127	-0.10684	-0.10804	0.049535
**cAMP Pathway (Cell Survival)**	0.011465	0.012897	0.010617	0.049535
**cAMP Pathway (Cell Survival, Chemotaxis)**	-0.2676	-0.21038	-0.24687	0.049535
**cAMP Pathway (Cytokine Production)**	-0.15291	-0.12022	-0.14107	0.049535
**cAMP Pathway (Degradation of Cell Cycle Regulators)**	-0.10091	-0.10665	-0.0769	0.049535
**cAMP Pathway (Endothelial Cell Regulation)**	0.037887	0.023993	0.060627	0.049535
**cAMP Pathway (Glycogen Synthesis)**	-0.13319	-0.12627	-0.12768	0.049535
**cAMP Pathway (Glycolysis)**	0.023752	0.03368	0.056436	0.049535
**cAMP Pathway (Metabolic Energy)**	0.075538	0.0699	0.058437	0.049535
**cAMP Pathway (Myocardial Contraction)**	-0.0297	-0.03997	-0.01743	0.049535
**cAMP Pathway (Oncogenesis)**	-0.10465	-0.09921	-0.10032	0.049535
**cAMP Pathway (Protein Retention)**	0.116422	0.13794	0.226651	0.12663
**cAMP Pathway (Regulation of Cytoskeleton)**	-0.09157	-0.08681	-0.08778	0.049535
**Caspase Cascade Main**	-0.05417	-0.05201	-0.05542	0.049535
**Caspase Cascade (Activated Tissue Transglutaminase)**	0.037353	-0.00437	0.044459	0.275234
**Caspase Cascade (Apoptosis)**	-0.08292	-0.08373	-0.0757	0.049535
**Caspase Cascade (Cell Survival)**	-0.12017	-0.11431	-0.1509	0.049535
**Caspase Cascade (ICAD Degradation)**	0	0	0	1
**CD40 Main Pathway**	0.03894	0.040173	0.028174	0.049535
**CD40 Pathway (Cell Survival)**	0	0	0	1
**CD40 Pathway (Gene Expression)**	0.077564	0.090056	0.05139	0.049535
**CD40 Pathway (IKBs Degradation)**	-0.05475	-0.06357	-0.03628	0.049535
**Cellular Anti Apoptosis Main Pathway**	0.049591	0.038846	0.062527	0.049535
**Cellular Anti Apoptosis Pathway (Apoptosis)**	0.03568	0.032518	0.029831	0.049535
**Cellular Anti Apoptosis Pathway (Depolarization)**	0	0	0	1
**Chemokine Main Pathway**	0.027177	0.018956	0.036278	0.049535
**Chemokine Pathway (Cell Activation)**	-0.04853	-0.08778	-0.04163	0.049535

Borger et al
Supplemental Data

**Chemokine Pathway (Gene Expression, Apoptosis)**	-0.06119	-0.11068	-0.05249	0.049535
**Chemokine Pathway (Internalization, Degradation, Recycling)**	-0.03851	-0.0589	-0.0777	0.049535
**Chromatin Main Pathway**	0.154373	0.201673	0.190655	0.049535
**Chromatin Pathway (Octamer Sliding)**	0.178123	0.232699	0.219986	0.049535
**Chromatin Pathway (Octamer Transfer)**	0.154373	0.201673	0.190655	0.049535
**Circadian Main Pathway**	0.114524	0.147999	0.139278	0.049535
**CREB Main Pathway**	0.049473	0.035683	0.053392	0.049535
**CREB Pathway (Gene Expression Pathway)**	0.028536	0.020907	-0.00195	0.512691
**Cytokine Main Pathway**	0	0	0	1
**DDR pathway Apoptosis**	0.994612	1.140498	0.955828	0.049535
**DDR Main pathway**	0.780207	0.904517	0.750381	0.049535
**DNA Repair Mechanisms Pathway**	0.174312	0.200041	0.159235	0.049535
**EGFR Main Pathway**	0.020203	0.014925	0.034889	0.049535
**ErbB Family Main Pathway**	0.052045	0.047577	0.059065	0.049535
**ErbB Family Pathway (Anti-Apoptosis)**	0	0	0	1
**ERK Signaling Main Pathway**	0.094798	0.07889	0.110907	0.049535
**Erythropoeitin Main Pathway**	0.010734	0.001124	0.018103	0.049535
**Estrogen Main Pathway**	0.060228	0.048553	0.072529	0.049535
**Fas Signaling Pathway (Negative)**	0.094125	0.092163	0.083682	0.049535
**Fas Signaling Pathway (Positive)**	0	0	0	1
**FLT3 Main Pathway**	0.074905	0.06659	0.070066	0.049535
**Glucocorticoid Receptor Main Pathway**	0.034695	0.037712	0.042526	0.049535
**Glucocorticoid Receptor Pathway (Cell cycle arrest)**	0.534654	0.266731	0.336548	0.049535
**Glucocorticoid Receptor Pathway (Cell cycle progression)**	0	0	0	1
**Glucocorticoid Receptor Pathway (Gene expression)**	0.038614	0.048889	0.046009	0.049535
**Glucocorticoid Receptor Pathway (Inflammatory cytokines)**	0.026287	0.030481	0.039986	0.049535
**Glucocorticoid Receptor Pathway (SMAD signaling)**	0	0	0	1
**GPCR Main Pathway**	0.075195	0.060527	0.080114	0.049535
**GPCR Pathway (Gene expression)**	0.01091	0.013456	0.01207	0.049535
**Growth Hormone Main Pathway**	-0.00855	-0.03446	-0.00642	0.275234
**Growth Hormone Pathway (Cell survival)**	0.509998	0.427402	0.582719	0.049535
**Growth Hormone Pathway (Gene expression)**	-0.03379	-0.06139	-0.03842	0.049535
**Growth Hormone Pathway (Glucose uptake)**	-0.00734	-0.0296	-0.00551	0.275234
**Growth Hormone Pathway (Protein synthesis)**	0.764997	0.641103	0.874079	0.049535
**GSK3 Main Pathway**	0.081741	0.071212	0.09152	0.049535
**GSK3 Pathway (Degradation)**	0	0	0	1
**GSK3 Pathway (Gene expression)**	0.10626	0.098293	0.098142	0.049535
**GSK3 Pathway (Translation)**	0	0	0	1
**G-protein Pathway (Ras family GTPases)**	0.03582	0.057165	0.058385	0.049535
**Hedgehog Main Pathway**	0.286093	0.338785	0.307757	0.049535
**Hedgehog Pathway (Repression of Hh, BMP)**	0.281988	0.323329	0.258048	0.049535
**Hedgehog Pathway (Activation of BMP, Ptc, WNT)**	0	0	0	1
**HGF Main Pathway**	0.079802	0.075184	0.082961	0.049535
**HGF Pathway (Anoikis)**	0.153773	0.146072	0.152049	0.049535
**HGF Pathway (Cell adhesion, cell mirgation)**	0.108208	0.10023	0.110398	0.049535
**HGF Pathway (Cell cycle progression)**	0.194931	0.195803	0.232179	0.049535
**HGF Pathway (Cell polarity, cell motility)**	0	0	0	1
**HGF Pathway (Cell scattering)**	0.124296	0.11391	0.107472	0.049535
**HGF Pathway (Cell survival)**	0.124296	0.11391	0.107472	0.049535
**HGF Pathway (IP3 pathway)**	0.142941	0.130997	0.123593	0.049535
**HGF Pathway (PKC pathway)**	0	0	0	1

**HIF1-Alpha Main Pathway**	0.02545	0.032889	0.030951	0.049535
**HIF1Alpha Pathway (Gene expression)**	0.091619	0.1184	0.111422	0.049535
**HIF1Alpha Pathway (HIF1alpha degradation)**	0	0	0	1
**HIF1Alpha Pathway (NOS pathway)**	0.105715	0.136615	0.128564	0.049535
**HIF1Alpha Pathway (p53 Hypoxia pathway)**	0	0	0	1
**HIF1Alpha Pathway (Pyruvate)**	0.091619	0.1184	0.111422	0.049535
**HIF1Alpha Pathway (VEGF pathway)**	0.085893	0.111	0.104458	0.049535
**Hypoxia pathway EMT 1**	0.89267	0.802141	0.973324	0.049535
**Hypoxia pathway EMT 2**	0.89267	0.802141	0.973324	0.049535
**Hypoxia pathway EMT 3**	0.89267	0.802141	0.973324	0.049535
**Hypoxia pathway EMT 4**	0.89267	0.802141	0.973324	0.049535
**IGF1R Main Pathway**	0.031878	0.016748	0.037119	0.049535
**IGF1R Signaling Pathway (Cell survival)**	0.027014	0.027066	0.025629	0.049535
**IGF1R Signaling Pathway (Glucose uptake)**	0	0	0	1
**IGF1R Signaling Pathway (Glycogen synthesis)**	0	0	0	1
**IGF1R Signaling Pathway (IKB degradation)**	0.062051	0.072045	0.041112	0.049535
**IGF1R Signaling Pathway (Protein synthesis)**	0	0	0	1
**ILK Main Pathway**	0.142092	0.130203	0.172716	0.049535
**ILK Pathway (Apoptosis)**	0.243874	0.224321	0.280643	0.049535
**ILK Pathway (Cell adhesion, cell motility, opsonization)**	0.263328	0.245084	0.308702	0.049535
**ILK Pathway (Cell cycle proliferation)**	0.244825	0.227818	0.283736	0.049535
**ILK Pathway (Cell migration, retraction)**	0.259149	0.241194	0.303802	0.049535
**ILK Pathway (Cell motility)**	0.210926	0.198114	0.251635	0.049535
**ILK Pathway (Cytoskeletal reorganization)**	0.245786	0.230464	0.294722	0.049535
**ILK Pathway (G2-phase arrest)**	0.244825	0.227818	0.283736	0.049535
**ILK Pathway (Induced cell proliferation)**	0.14231	0.129934	0.161922	0.049535
**ILK Pathway (Regulation of intermediate filaments)**	0.267768	0.248166	0.31423	0.049535
**ILK Pathway (Regulation of junction assembly of desmosomes)**	0.261222	0.243123	0.306232	0.049535
**ILK Pathway (Wound healing)**	0.273847	0.254804	0.319065	0.049535
**IL-10 Main Pathway**	0.129986	0.131055	0.129441	0.049535
**IL-10 Pathway (Gene expression)**	0.058173	0.067542	0.038542	0.049535
**IL-10 Pathway (Stability determination)**	0	0	0	1
**IL-10 Pathway (Translational modulation)**	0	0	0	1
**IL-2 Main Pathway**	-0.0023	0.01259	0.015912	0.512691
**IL-2 Pathway (Actin reorganization)**	0.137727	0.112631	0.128634	0.049535
**IL-2 Pathway (Apoptosis)**	0.131479	0.19551	0.136187	0.049535
**IL-2 Pathway (Apoptosis inhibition)**	0.133638	0.205085	0.166523	0.049535
**IL-2 Pathway (Protein synthesis)**	0	0	0	1
**IL-6 Main Pathway**	0.053093	0.046232	0.05661	0.049535
**Integrin SIgnaling Main Pathway**	0.156633	0.141103	0.180467	0.049535
**Integrin SIgnaling Pathway (Cell survival)**	0.034958	0.028	0.028444	0.049535
**Integrin SIgnaling Pathway (Cytoskeleton contraction integrin modulation cell invasion and migration)**	-0.02621	-0.02363	-0.01812	0.049535
**Integrin SIgnaling Pathway (Focal adhesion and stress fibers)**	0.046719	0.046207	0.05975	0.049535
**Integrin SIgnaling Pathway (Translocation to the nucleus)**	0.188348	0.093023	0.098492	0.049535
**Interactions Report**	0.803403	0.721927	0.875991	0.049535
**Interferon Main Pathway**	0.013361	0.012	0.013524	0.049535
**Interferon Pathway (Gene expression)**	0	0	0	1
**Interferon Pathway (Transcription)**	0	0	0	1
**Interferon Pathway (Translation)**	0	0	0	1
**IP3 Main Pathway**	0.018806	0.009881	0.021121	0.049535

**IP3 Pathway (Gene expression)**	0.073646	0.075697	0.051863	0.049535
**JAK mStat Main Pathway**	0.11073	0.124833	0.105898	0.049535
**JAK mStat Pathway (Akt pathway)**	0.081147	0.070243	0.09044	0.049535
**JAK mStat Pathway (JAK degradation)**	-0.01977	-0.01908	-0.01538	0.049535
**JNK Main Pathway**	0.073204	0.066221	0.087196	0.049535
**JNK Pathway (Apoptosis, Inflammation, Tumorigenesis, Cell Migration)**	0.097647	0.092186	0.102668	0.049535
**JNK Pathway (Insulin signaling)**	0	0	0	1
**MAPK Family Main Pathway**	0.022231	0.014809	0.027637	0.049535
**MAPK Family Pathway (Chromatin Remodeling)**	-0.02196	-0.0636	-0.01928	0.049535
**MAPK Family Pathway (Cytoskeleton)**	-0.18713	-0.30007	-0.13055	0.049535
**MAPK Family Pathway (Gene Expression)**	-0.01222	-0.03756	-0.01514	0.049535
**MAPK Family Pathway (IKBs Degradation)**	0.071597	0.083128	0.047437	0.049535
**MAPK Family Pathway (Translation)**	0	0	0	1
**MAPK Signaling Main Pathway**	0.108187	0.093827	0.125028	0.049535
**MAPK Signaling Pathway (Cell Survival, Inflammation, Apoptosis, Osmoregulation)**	0.150707	0.129762	0.162951	0.049535
**MAPK Signaling Pathway (Gene Expression)**	0.065278	0.054399	0.069908	0.049535
**DDR pathway (MMR)**	0.624213	0.724759	0.58544	0.049535
**Mismatch Repair Main Pathway**	0.728948	0.87579	0.705174	0.049535
**Mitochondrial Apopotosis Main Pathway**	-0.09227	-0.08221	-0.10414	0.049535
**Mitochondrial Apopotosis Pathway (Apoptosis)**	0.095146	0.086715	0.079551	0.049535
**Mitochondrial Apopotosis Pathway (Depolarization)**	0	0	0	1
**Mitochondrial Apopotosis Pathway (DNA fragmentation)**	-0.14486	-0.12891	-0.23582	0.049535
**Mitochondrial Apopotosis Pathway (Gene expression)**	-0.30401	-0.35745	-0.24256	0.049535
**mTOR Main Pathway**	0.05139	0.03171	0.063379	0.049535
**mTOR Pathway (Actin organization)**	0.006471	-0.0036	0.033927	0.512691
**mTOR Pathway (Akt signaling)**	0	0	0	1
**mTOR Pathway (Scanning)**	0	0	0	1
**mTOR Pathway (Translation on)**	0	0	0	1
**mTOR Pathway (VEGF pathway)**	0	0	0	1
**NGF (Negative) Main Pathway**	0	0	0	1
**NGF (Negative) Pathway (Apoptosis)**	0	0	0	1
**NGF (Positive) Main Pathway**	0.060153	0.047934	0.054554	0.049535
**NHEJ mechanisms of DSBs repair effect**	0.290869	0.343563	0.276643	0.049535
**Notch Main Pathway**	0.027155	0.0419	0.028805	0.049535
**DDR Pathway (NER)**	0.227787	0.272262	0.231125	0.049535
**p38 (Negative) Main Signaling Pathway**	0.051272	0.040313	0.0613	0.049535
**p38 (Positive) Main Signaling Pathway**	0.051168	0.040231	0.061175	0.049535
**p53 Signaling (Negative) Main Pathway**	0.02272	0.001631	0.000723	0.827259
**p53 Signaling (Negative) Pathway (p53 Degradation)**	0.024456	0.014127	0.026535	0.049535
**PAK Main Pathway**	0.073179	0.064732	0.090114	0.049535
**PAK Pathway (Actin Cytoskeleton)**	0.061895	0.061529	0.080164	0.049535
**PAK Pathway (Myosin Activation)**	0.070233	0.0692	0.09716	0.049535
**PPAR Main Pathway**	0.065344	0.051704	0.07544	0.049535
**PTEN Main Pathway**	-0.04153	-0.02581	-0.03715	0.049535
**RANK Signaling in Osteoclast Main Pathway**	0.07319	0.078086	0.06996	0.049535
**RANK Signaling in Osteoclast Pathway (IKBs Degradation)**	0.046538	0.054033	0.030834	0.049535
**RAS Main Pathway**	0.109894	0.103871	0.112969	0.049535
**Telomere Main Pathway**	0.002405	0.012154	0.036094	0.049535
**RNA Polymerase II Complex Pathway**	0.011524	0.013727	0.013866	0.049535
**Cell Cycle Pathway (SCC during S-phase)**	-0.2675	-0.24173	-0.16512	0.049535

**SMAD (Negative) Main Pathway**	0.058948	0.05618	0.071333	0.049535
**SMAD (Negative) Pathway (Degradation)**	0.010506	0.012594	0.013321	0.049535
**SMAD (Positive) Main Pathway**	0.058948	0.05618	0.071333	0.049535
**SMAD (Positive) Pathway (Degradation)**	0.010506	0.012594	0.013321	0.049535
**Cell Cycle Pathway (Metaphase-Anaphase)**	0.566138	0.592349	0.48403	0.049535
**Cell Cycle Pathway (Origin of S-phase)**	1.006473	1.207531	1.13324	0.049535
**STAT3 Main Pathway**	0.05439	0.050247	0.055703	0.049535
**TGF beta Main Pathway**	0.046411	0.040776	0.061264	0.049535
**TGF beta Pathway (Epithelial mesehchymal transition)**	0.091707	0.093628	0.086799	0.049535
**TGF beta Pathway (Post-transcriptional G1 arrest)**	0.083562	0.070026	0.107422	0.049535
**TGF beta Pathway (SnON degradation)**	0	0	0	1
**TGF beta Pathway (Tumorigenesis)**	0	0	0	1
**TGF beta Pathway (Tumor suppression)**	0	0	0	1
**TNF (Negative) Main Pathway**	0.01584	0.017096	0.022154	0.049535
**TNF (Negative) Pathway (Apoptosis)**	0.017704	0.019107	0.02476	0.049535
**TNF (Positive) Main Pathway**	0.031224	0.022817	0.047216	0.049535
**TNF (Positive) Pathway (Gene expression, Cell survival)**	0.11005	0.097148	0.115465	0.049535
**TNF (Positive) Pathway (IKBs degradation)**	0.051709	0.060037	0.03426	0.049535
**TRAF (Negative) Main Pathway**	-0.08408	-0.07158	-0.05337	0.049535
**TRAF (Negative) Pathway (IKBs Degradation)**	0.051709	0.060037	0.03426	0.049535
**TRAF (Positive) Main Pathway**	-0.01621	-0.00823	-0.00985	0.049535
**TRAF (Positive) Pathway (IKBs Degradation)**	0.051709	0.060037	0.03426	0.049535
**Transcription of mRNA Pathway**	0.013444	0.016015	0.016177	0.049535
**Cell Cycle Pathway (End of S-phase)**	-0.01372	0.020322	-0.00634	0.827259
**Translation Regulation of EIF4F activity**	-0.00082	-0.00451	0.005037	0.512691
**Ubiquitin Proteasome Main Pathway**	0.113309	0.126927	0.107144	0.049535
**Ubiquitin Proteasome Pathway (Degraded Protein)**	0.200411	0.2141	0.160671	0.049535
**VEGF Main Pathway**	0.038914	0.015262	0.062675	0.049535
**VEGF Pathway (Actin Reorganization)**	0.132323	0.092549	0.167216	0.049535
**VEGF Pathway (Nitric Oxide Production)**	0	0	0	1
**Wnt Main Pathway**	0.065248	0.068961	0.072748	0.049535
**Wnt Pathway (Ctnn-b Degradation)**	0	0	0	1
**IL-6 Pathway (IKBs degradation)**	0.077564	0.090056	0.05139	0.049535

**Pathway**	**M_86_1h_1**	**M_86_1h_2**	**M_86_1h_3**	**p-value_Mean**
**AHR Main Pathway**	0	0	0	1
**AHR Pathway (AHR Degradation)**	0	0	0	1
**AHR Pathway (Cath-D Expression)**	0	0	0	1
**AHR Pathway (C-MycExpression)**	0	0	0	1
**AHR Pathway (PS2 Gene Expression)**	0	0	0	1
**AKT Main Pathway**	0.027896	0.02529	0.028096	0.049535
**AKT Pathway (Aggregation & Neurodegeneration)**	0.018409	0.016008	0.020155	0.049535
**AKT Pathway (Apoptosis Inhibition)**	0.017687	0.015381	0.019364	0.049535
**AKT Pathway (Blocks Apoptosis)**	0	0	0	1
**AKT Pathway (Cardiovascular Homeostasis)**	0.020046	0.017431	0.021946	0.049535
**AKT Pathway (Caspase Cascade)**	0	0	0	1
**AKT Pathway (Cell Cycle)**	0	0	0	1
**AKT Pathway (Cell Cycle Progression)**	0.040536	0.045611	0.030169	0.049535
**AKT Pathway (Cell Survival)**	0	0	0	1

**AKT Pathway (Death Genes)**	0	0	0	1
**AKT Pathway (Elevation of Glucose Import)**	0	0	0	1
**AKT Pathway (ERK Pathway)**	0	0	0	1
**AKT Pathway (Genetic Stability)**	0	0	0	1
**AKT Pathway (Glucose Uptake)**	0.020501	0.017828	0.022445	0.049535
**AKT Pathway (Glycogen Synthesis)**	0	0	0	1
**AKT Pathway (Insulin Stimulated Mitogenesis)**	0.020046	0.017431	0.021946	0.049535
**AKT Pathway (JNK Pathway)**	0	0	0	1
**AKT Pathway (Neuroprotection)**	0.020046	0.017431	0.021946	0.049535
**AKT Pathway (NF-kB Pathway)**	0.018793	0.016342	0.020575	0.049535
**AKT Pathway (p53 Degradation)**	0	0	0	1
**AKT Pathway (p73 Mediated Apoptosis)**	0	0	0	1
**AKT Pathway (Protein Synthesis)**	0	0	0	1
**AKT Pathway (Regeneration of Cyclic Nucleotide)**	0	0	0	1
**AKT Pathway (Respiratory Burst)**	0.020046	0.017431	0.021946	0.049535
**AKT Pathway (Survival Genes)**	0	0	0	1
**AKT Pathway (Synaptic Signaling)**	0	0	0	1
**AKT Pathway (Translation)**	0	0	0	1
**Androgen Receptor Pathway**	0.036135	0.031967	0.03486	0.049535
**Androgen Receptor Pathway (Apoptosis)**	0	0	0	1
**Androgen Receptor Pathway (Degradation)**	0.039705	0.039764	0.04586	0.049535
**Androgen Receptor Pathway (Cell Survival & Cell Growth)**	0	0	0	1
**Androgen Receptor Pathway (Gonadotropin Regulation)**	0.185333	0.177203	0.200038	0.049535
**Androgen Receptor Pathway (Histone Modification)**	0.185333	0.177203	0.200038	0.049535
**Androgen Receptor Pathway (Prostate Differentiation & Development)**	0.185333	0.177203	0.200038	0.049535
**Androgen Receptor Pathway (Sexual Differentiation & Sexual Maturation at Puberty)**	0.185333	0.177203	0.200038	0.049535
**ATM Main Pathway**	0.047114	0.040296	0.059754	0.049535
**ATM Pathway (Apoptosis)**	0	0	0	1
**ATM Pathway (Apoptosis, Senescense)**	0	0	0	1
**ATM Pathway (Cell Cicle Checkpoint Control)**	0	0	0	1
**ATM Pathway (Cell Survival)**	0.112989	0.107874	0.122581	0.049535
**ATM Pathway (Checkpoint Activation)**	0	0	0	1
**ATM Pathway (DNA Repair)**	0	0	0	1
**ATM Pathway (G2_M Checkpoint Arrest)**	0.047229	0.025072	0.088157	0.049535
**ATM Pathway (G2 Mitosis Progression)**	0	0	0	1
**ATM Pathway (MDMX Ubiquitination, Degradation)**	0	0	0	1
**ATM Pathway (NF-kB Pathway)**	0	0	0	1
**ATM Pathway (Synaptic Vesicle Transport)**	0	0	0	1
**ATM Pathway (S-Phase Arrest)**	0	0	0	1
**ATM Pathway (S-Phase Progression)**	0	0	0	1
**DDR Pathway (BRCA1-induced responses)**	-0.22286	-0.22795	-0.18961	0.049535
**BRCA1 Main Pathway**	0.00783	0.008172	0.000552	0.049535
**cAMP Main Pathway**	0.008144	0.007368	0.006893	0.049535
**cAMP Pathway (Axonal Growth)**	0	0	0	1
**cAMP Pathway (Cardiovascular Homeostasis)**	0	0	0	1
**cAMP Pathway (Cell Growth)**	0	0	0	1
**cAMP Pathway (Cell Proliferation)**	0	0	0	1
**cAMP Pathway (Cell Survival)**	0	0	0	1
**cAMP Pathway (Cell Survival, Chemotaxis)**	0	0	0	1
**cAMP Pathway (Cytokine Production)**	0	0	0	1

**cAMP Pathway (Degradation of Cell Cycle Regulators)**	0	0	0	1
**cAMP Pathway (Endothelial Cell Regulation)**	0	0	0	1
**cAMP Pathway (Glycogen Synthesis)**	0	0	0	1
**cAMP Pathway (Glycolysis)**	0	0	0	1
**cAMP Pathway (Metabolic Energy)**	0	0	0	1
**cAMP Pathway (Myocardial Contraction)**	0	0	0	1
**cAMP Pathway (Oncogenesis)**	0	0	0	1
**cAMP Pathway (Protein Retention)**	0	0	0	1
**cAMP Pathway (Regulation of Cytoskeleton)**	0	0	0	1
**Caspase Cascade Main**	-0.01582	-0.01379	-0.01409	0.049535
**Caspase Cascade (Activated Tissue Transglutaminase)**	0	0	0	1
**Caspase Cascade (Apoptosis)**	0	0	0	1
**Caspase Cascade (Cell Survival)**	0	0	0	1
**Caspase Cascade (ICAD Degradation)**	0	0	0	1
**CD40 Main Pathway**	0.019488	0.016097	0.016549	0.049535
**CD40 Pathway (Cell Survival)**	0	0	0	1
**CD40 Pathway (Gene Expression)**	0.086074	0.071096	0.073093	0.049535
**CD40 Pathway (IKBs Degradation)**	0.060758	0.050186	0.051595	0.049535
**Cellular Anti Apoptosis Main Pathway**	0.023533	0.020291	0.019139	0.049535
**Cellular Anti Apoptosis Pathway (Apoptosis)**	0	0	0	1
**Cellular Anti Apoptosis Pathway (Depolarization)**	0	0	0	1
**Chemokine Main Pathway**	0.060062	0.055273	0.063833	0.049535
**Chemokine Pathway (Cell Activation)**	0	0	0	1
**Chemokine Pathway (Gene Expression, Apoptosis)**	0	0	0	1
**Chemokine Pathway (Internalization, Degradation, Recycling)**	0	0	0	1
**Chromatin Main Pathway**	0	0	0	1
**Chromatin Pathway (Octamer Sliding)**	0	0	0	1
**Chromatin Pathway (Octamer Transfer)**	0	0	0	1
**Circadian Main Pathway**	-0.07233	-0.05406	-0.06871	0.049535
**CREB Main Pathway**	0.014639	0.012912	0.01318	0.049535
**CREB Pathway (Gene Expression Pathway)**	0	0	0	1
**Cytokine Main Pathway**	0.054902	0.058454	0.075092	0.049535
**DDR pathway Apoptosis**	0	0	0	1
**DDR Main pathway**	0	0	0	1
**DNA Repair Mechanisms Pathway**	-0.01572	-0.01534	-0.01615	0.049535
**EGFR Main Pathway**	0.0998	0.097553	0.099154	0.049535
**ErbB Family Main Pathway**	0.115499	0.102643	0.126909	0.049535
**ErbB Family Pathway (Anti-Apoptosis)**	0	0	0	1
**ERK Signaling Main Pathway**	0.028823	0.025788	0.029733	0.049535
**Erythropoeitin Main Pathway**	0.023755	0.021621	0.025563	0.049535
**Estrogen Main Pathway**	0.021635	0.021441	0.022244	0.049535
**Fas Signaling Pathway (Negative)**	0.043457	0.04149	0.047146	0.049535
**Fas Signaling Pathway (Positive)**	0	0	0	1
**FLT3 Main Pathway**	0	0	0	1
**Glucocorticoid Receptor Main Pathway**	0.042057	0.038185	0.048573	0.049535
**Glucocorticoid Receptor Pathway (Cell cycle arrest)**	-0.61614	-0.69328	-0.45858	0.049535
**Glucocorticoid Receptor Pathway (Cell cycle progression)**	0	0	0	1
**Glucocorticoid Receptor Pathway (Gene expression)**	0	0	0	1
**Glucocorticoid Receptor Pathway (Inflammatory cytokines)**	0.079481	0.076861	0.083026	0.049535
**Glucocorticoid Receptor Pathway (SMAD signaling)**	0	0	0	1
**GPCR Main Pathway**	0.025003	0.023388	0.0242	0.049535

**GPCR Pathway (Gene expression)**	0.08449	0.080784	0.091194	0.049535
**Growth Hormone Main Pathway**	-0.02875	-0.02597	-0.03041	0.049535
**Growth Hormone Pathway (Cell survival)**	0	0	0	1
**Growth Hormone Pathway (Gene expression)**	0	0	0	1
**Growth Hormone Pathway (Glucose uptake)**	-0.02469	-0.0223	-0.02612	0.049535
**Growth Hormone Pathway (Protein synthesis)**	0	0	0	1
**GSK3 Main Pathway**	0.004789	0.004284	0.002669	0.049535
**GSK3 Pathway (Degradation)**	0	0	0	1
**GSK3 Pathway (Gene expression)**	0	0	0	1
**GSK3 Pathway (Translation)**	0	0	0	1
**G-protein Pathway (Ras family GTPases)**	0	0	0	1
**Hedgehog Main Pathway**	0	0	0	1
**Hedgehog Pathway (Repression of Hh, BMP)**	0.032814	0.045253	0.059835	0.049535
**Hedgehog Pathway (Activation of BMP, Ptc, WNT)**	0	0	0	1
**HGF Main Pathway**	0.070478	0.065531	0.078337	0.049535
**HGF Pathway (Anoikis)**	0.204386	0.190041	0.227177	0.049535
**HGF Pathway (Cell adhesion, cell mirgation)**	0	0	0	1
**HGF Pathway (Cell cycle progression)**	0.681286	0.633469	0.757258	0.049535
**HGF Pathway (Cell polarity, cell motility)**	0	0	0	1
**HGF Pathway (Cell scattering)**	0	0	0	1
**HGF Pathway (Cell survival)**	0	0	0	1
**HGF Pathway (IP3 pathway)**	0	0	0	1
**HGF Pathway (PKC pathway)**	0	0	0	1
**HIF1-Alpha Main Pathway**	0.031386	0.029965	0.03405	0.049535
**HIF1Alpha Pathway (Gene expression)**	0.112989	0.107874	0.122581	0.049535
**HIF1Alpha Pathway (HIF1alpha degradation)**	0	0	0	1
**HIF1Alpha Pathway (NOS pathway)**	0.130372	0.12447	0.141439	0.049535
**HIF1Alpha Pathway (p53 Hypoxia pathway)**	0	0	0	1
**HIF1Alpha Pathway (Pyruvate)**	0.112989	0.107874	0.122581	0.049535
**HIF1Alpha Pathway (VEGF pathway)**	0.105928	0.101132	0.114919	0.049535
**Hypoxia pathway EMT 1**	0	0	0	1
**Hypoxia pathway EMT 2**	0	0	0	1
**Hypoxia pathway EMT 3**	0	0	0	1
**Hypoxia pathway EMT 4**	0	0	0	1
**IGF1R Main Pathway**	0.0148	0.019363	0.017166	0.049535
**IGF1R Signaling Pathway (Cell survival)**	0	0	0	1
**IGF1R Signaling Pathway (Glucose uptake)**	0	0	0	1
**IGF1R Signaling Pathway (Glycogen synthesis)**	0	0	0	1
**IGF1R Signaling Pathway (IKB degradation)**	0.068859	0.056877	0.058474	0.049535
**IGF1R Signaling Pathway (Protein synthesis)**	0	0	0	1
**ILK Main Pathway**	0.053293	0.046253	0.049728	0.049535
**ILK Pathway (Apoptosis)**	0.011231	-0.00113	0.00187	0.512691
**ILK Pathway (Cell adhesion, cell motility, opsonization)**	0	-0.00954	-0.00735	0.19043
**ILK Pathway (Cell cycle proliferation)**	0	-0.00857	-0.0066	0.19043
**ILK Pathway (Cell migration, retraction)**	0	-0.00939	-0.00723	0.19043
**ILK Pathway (Cell motility)**	0.005589	-0.00139	0.001528	0.512691
**ILK Pathway (Cytoskeletal reorganization)**	0.017377	0.007399	0.012077	0.049535
**ILK Pathway (G2-phase arrest)**	0	-0.00857	-0.0066	0.19043
**ILK Pathway (Induced cell proliferation)**	0.09211	0.092431	0.072412	0.049535
**ILK Pathway (Regulation of intermediate filaments)**	0	-0.00946	-0.00729	0.19043
**ILK Pathway (Regulation of junction assembly of desmosomes)**	0	-0.00946	-0.00729	0.19043

**ILK Pathway (Wound healing)**	0	-0.00946	-0.00729	0.19043
**IL-10 Main Pathway**	0.091219	0.094321	0.085816	0.049535
**IL-10 Pathway (Gene expression)**	0.064555	0.053322	0.05482	0.049535
**IL-10 Pathway (Stability determination)**	0	0	0	1
**IL-10 Pathway (Translational modulation)**	-0.1926	-0.17398	-0.20372	0.049535
**IL-2 Main Pathway**	0.083394	0.074773	0.080944	0.049535
**IL-2 Pathway (Actin reorganization)**	0.148154	0.133827	0.15671	0.049535
**IL-2 Pathway (Apoptosis)**	0	0	0	1
**IL-2 Pathway (Apoptosis inhibition)**	0	0	0	1
**IL-2 Pathway (Protein synthesis)**	0.175091	0.15816	0.185203	0.049535
**IL-6 Main Pathway**	0.046378	0.044348	0.047319	0.049535
**Integrin SIgnaling Main Pathway**	0.038617	0.032652	0.03509	0.049535
**Integrin SIgnaling Pathway (Cell survival)**	0	0	0	1
**Integrin SIgnaling Pathway (Cytoskeleton contraction integrin modulation cell invasion and migration)**	0.127363	0.125409	0.143893	0.049535
**Integrin SIgnaling Pathway (Focal adhesion and stress fibers)**	0	0	0	1
**Integrin SIgnaling Pathway (Translocation to the nucleus)**	0	0	0	1
**Interactions Report**	0	0	0	1
**Interferon Main Pathway**	0.021718	0.028413	0.025189	0.049535
**Interferon Pathway (Gene expression)**	0	0	0	1
**Interferon Pathway (Transcription)**	0	0	0	1
**Interferon Pathway (Translation)**	0	0	0	1
**IP3 Main Pathway**	0.005126	0.006198	0.007024	0.049535
**IP3 Pathway (Gene expression)**	0	0	0	1
**JAK mStat Main Pathway**	0.103477	0.10543	0.096919	0.049535
**JAK mStat Pathway (Akt pathway)**	0	0	0	1
**JAK mStat Pathway (JAK degradation)**	0	0	0	1
**JNK Main Pathway**	0.002468	0.000679	0.000322	0.275234
**JNK Pathway (Apoptosis, Inflammation, Tumorigenesis, Cell Migration)**	0.03606	0.034428	0.039121	0.049535
**JNK Pathway (Insulin signaling)**	-0.1851	-0.18727	-0.22286	0.049535
**MAPK Family Main Pathway**	0.02145	0.020386	0.021262	0.049535
**MAPK Family Pathway (Chromatin Remodeling)**	0	0	0	1
**MAPK Family Pathway (Cytoskeleton)**	0	0	0	1
**MAPK Family Pathway (Gene Expression)**	0.069942	0.063366	0.069637	0.049535
**MAPK Family Pathway (IKBs Degradation)**	0.079453	0.065627	0.06747	0.049535
**MAPK Family Pathway (Translation)**	0	0	0	1
**MAPK Signaling Main Pathway**	0.038943	0.033996	0.038151	0.049535
**MAPK Signaling Pathway (Cell Survival, Inflammation, Apoptosis, Osmoregulation)**	0.094477	0.080643	0.089866	0.049535
**MAPK Signaling Pathway (Gene Expression)**	0.056776	0.055905	0.064145	0.049535
**DDR pathway (MMR)**	0	0	0	1
**Mismatch Repair Main Pathway**	0	0	0	1
**Mitochondrial Apopotosis Main Pathway**	-0.00771	-0.00457	-0.00535	0.049535
**Mitochondrial Apopotosis Pathway (Apoptosis)**	0	0	0	1
**Mitochondrial Apopotosis Pathway (Depolarization)**	0	0	0	1
**Mitochondrial Apopotosis Pathway (DNA fragmentation)**	0	0	0	1
**Mitochondrial Apopotosis Pathway (Gene expression)**	0	0	0	1
**mTOR Main Pathway**	0.012937	0.011609	0.009875	0.049535
**mTOR Pathway (Actin organization)**	0.054938	0.049475	0.0545	0.049535
**mTOR Pathway (Akt signaling)**	0	0	0	1
**mTOR Pathway (Scanning)**	0	0	0	1
**mTOR Pathway (Translation on)**	0	0	0	1

**mTOR Pathway (VEGF pathway)**	0	0	0	1
**NGF (Negative) Main Pathway**	0	0	0	1
**NGF (Negative) Pathway (Apoptosis)**	0	0	0	1
**NGF (Positive) Main Pathway**	0.057203	0.054856	0.045874	0.049535
**NHEJ mechanisms of DSBs repair effect**	0	0	0	1
**Notch Main Pathway**	0	0	0	1
**DDR Pathway (NER)**	0	0	0	1
**p38 (Negative) Main Signaling Pathway**	0.035134	0.030717	0.037796	0.049535
**p38 (Positive) Main Signaling Pathway**	0.035062	0.030654	0.037719	0.049535
**p53 Signaling (Negative) Main Pathway**	0.099876	0.086335	0.110895	0.049535
**p53 Signaling (Negative) Pathway (p53 Degradation)**	0	0	0	1
**PAK Main Pathway**	0.028246	0.025086	0.030016	0.049535
**PAK Pathway (Actin Cytoskeleton)**	0.044444	0.039471	0.047228	0.049535
**PAK Pathway (Myosin Activation)**	0	0	0	1
**PPAR Main Pathway**	0.012366	0.009933	0.009734	0.049535
**PTEN Main Pathway**	0	0	0	1
**RANK Signaling in Osteoclast Main Pathway**	0.093019	0.087904	0.101338	0.049535
**RANK Signaling in Osteoclast Pathway (IKBs Degradation)**	0.051644	0.042658	0.043856	0.049535
**RAS Main Pathway**	0.014373	0.015293	0.014251	0.049535
**Telomere Main Pathway**	0.106403	0.106774	0.083648	0.049535
**RNA Polymerase II Complex Pathway**	0	0	0	1
**Cell Cycle Pathway (SCC during S-phase)**	0	0	0	1
**SMAD (Negative) Main Pathway**	0.150536	0.132403	0.150081	0.049535
**SMAD (Negative) Pathway (Degradation)**	0	0	0	1
**SMAD (Positive) Main Pathway**	0.150536	0.132403	0.150081	0.049535
**SMAD (Positive) Pathway (Degradation)**	0	0	0	1
**Cell Cycle Pathway (Metaphase-Anaphase)**	0	0	0	1
**Cell Cycle Pathway (Origin of S-phase)**	0	0	0	1
**STAT3 Main Pathway**	0.048446	0.042526	0.048591	0.049535
**TGF beta Main Pathway**	0.034237	0.032572	0.038419	0.049535
**TGF beta Pathway (Epithelial mesehchymal transition)**	0	0	0	1
**TGF beta Pathway (Post-transcriptional G1 arrest)**	0	0	0	1
**TGF beta Pathway (SnON degradation)**	0.471242	0.464015	0.532406	0.049535
**TGF beta Pathway (Tumorigenesis)**	0.673203	0.662879	0.760579	0.049535
**TGF beta Pathway (Tumor suppression)**	0.673203	0.662879	0.760579	0.049535
**TNF (Negative) Main Pathway**	0	0	0	1
**TNF (Negative) Pathway (Apoptosis)**	0	0	0	1
**TNF (Positive) Main Pathway**	0.157871	0.147362	0.167342	0.049535
**TNF (Positive) Pathway (Gene expression, Cell survival)**	0.292286	0.269129	0.302562	0.049535
**TNF (Positive) Pathway (IKBs degradation)**	0.057383	0.047398	0.048729	0.049535
**TRAF (Negative) Main Pathway**	0	0	0	1
**TRAF (Negative) Pathway (IKBs Degradation)**	0.057383	0.047398	0.048729	0.049535
**TRAF (Positive) Main Pathway**	0.082239	0.082845	0.098465	0.049535
**TRAF (Positive) Pathway (IKBs Degradation)**	0.057383	0.047398	0.048729	0.049535
**Transcription of mRNA Pathway**	0	0	0	1
**Cell Cycle Pathway (End of S-phase)**	0	0	0	1
**Translation Regulation of EIF4F activity**	0	0	0	1
**Ubiquitin Proteasome Main Pathway**	0	0	0	1
**Ubiquitin Proteasome Pathway (Degraded Protein)**	0	0	0	1
**VEGF Main Pathway**	0.016589	0.014662	0.018993	0.049535
**VEGF Pathway (Actin Reorganization)**	0.032659	0.028866	0.037392	0.049535

Borger et al
Supplemental Data

**VEGF Pathway (Nitric Oxide Production)**	0	0	0	1
**Wnt Main Pathway**	0.039935	0.036491	0.034757	0.049535
**Wnt Pathway (Ctnn-b Degradation)**	0	0	0	1
**IL-6 Pathway (IKBs degradation)**	0.086074	0.071096	0.073093	0.049535

**Pathway**	**M_86_4h_1**	**M_86_4h_2**	**M_86_4h_3**
**AHR Main Pathway**	0	0	0
**AHR Pathway (AHR Degradation)**	0	0	0
**AHR Pathway (Cath-D Expression)**	0	0	0
**AHR Pathway (C-MycExpression)**	0	0	0
**AHR Pathway (PS2 Gene Expression)**	0	0	0
**AKT Main Pathway**	0.024117	0.0211	0.014302
**AKT Pathway (Aggregation & Neurodegeneration)**	-0.0436	-0.03755	-0.01971
**AKT Pathway (Apoptosis Inhibition)**	-0.04189	-0.03608	-0.01893
**AKT Pathway (Blocks Apoptosis)**	0	0	0
**AKT Pathway (Cardiovascular Homeostasis)**	-0.04748	-0.04089	-0.02146
**AKT Pathway (Caspase Cascade)**	0	0	0
**AKT Pathway (Cell Cycle)**	0	0	0
**AKT Pathway (Cell Cycle Progression)**	0	0	0
**AKT Pathway (Cell Survival)**	0	0	0
**AKT Pathway (Death Genes)**	0	0	0
**AKT Pathway (Elevation of Glucose Import)**	0	0	0
**AKT Pathway (ERK Pathway)**	0	0	0
**AKT Pathway (Genetic Stability)**	0	0	0
**AKT Pathway (Glucose Uptake)**	-0.04856	-0.04182	-0.02195
**AKT Pathway (Glycogen Synthesis)**	0	0	0
**AKT Pathway (Insulin Stimulated Mitogenesis)**	-0.04748	-0.04089	-0.02146
**AKT Pathway (JNK Pathway)**	0	0	0
**AKT Pathway (Neuroprotection)**	-0.04748	-0.04089	-0.02146
**AKT Pathway (NF-kB Pathway)**	-0.04451	-0.03833	-0.02012
**AKT Pathway (p53 Degradation)**	0	0	0
**AKT Pathway (p73 Mediated Apoptosis)**	0	0	0
**AKT Pathway (Protein Synthesis)**	0	0	0
**AKT Pathway (Regeneration of Cyclic Nucleotide)**	0	0	0
**AKT Pathway (Respiratory Burst)**	-0.04748	-0.04089	-0.02146
**AKT Pathway (Survival Genes)**	0	0	0
**AKT Pathway (Synaptic Signaling)**	0	0	0
**AKT Pathway (Translation)**	0	0	0
**Androgen Receptor Pathway**	0.045189	0.037219	0.033572
**Androgen Receptor Pathway (Apoptosis)**	0	0	0
**Androgen Receptor Pathway (Degradation)**	0.063675	0.057234	0.046014
**Androgen Receptor Pathway (Cell Survival & Cell Growth)**	0	0	0
**Androgen Receptor Pathway (Gonadotropin Regulation)**	0.155931	0.129067	0.126325
**Androgen Receptor Pathway (Histone Modification)**	0.155931	0.129067	0.126325
**Androgen Receptor Pathway (Prostate Differentiation & Development)**	0.155931	0.129067	0.126325
**Androgen Receptor Pathway (Sexual Differentiation & Sexual Maturation at Puberty)**	0.155931	0.129067	0.126325
**ATM Main Pathway**	0.043056	0.044558	0.043182
**ATM Pathway (Apoptosis)**	0	0	0
**ATM Pathway (Apoptosis, Senescense)**	0	0	0

**ATM Pathway (Cell Cicle Checkpoint Control)**	0	0	0
**ATM Pathway (Cell Survival)**	0	0	0
**ATM Pathway (Checkpoint Activation)**	0	0	0
**ATM Pathway (DNA Repair)**	0	0	0
**ATM Pathway (G2_M Checkpoint Arrest)**	0.183967	0.190384	0.184503
**ATM Pathway (G2 Mitosis Progression)**	0	0	0
**ATM Pathway (MDMX Ubiquitination, Degradation)**	0	0	0
**ATM Pathway (NF-kB Pathway)**	0	0	0
**ATM Pathway (Synaptic Vesicle Transport)**	0	0	0
**ATM Pathway (S-Phase Arrest)**	0	0	0
**ATM Pathway (S-Phase Progression)**	0	0	0
**DDR Pathway (BRCA1-induced responses)**	-0.15967	-0.16556	-0.12285
**BRCA1 Main Pathway**	-0.02891	-0.02992	-0.02899
**cAMP Main Pathway**	0.02747	0.023533	0.016396
**cAMP Pathway (Axonal Growth)**	-0.05094	-0.05609	-0.04747
**cAMP Pathway (Cardiovascular Homeostasis)**	-0.10916	-0.12019	-0.10173
**cAMP Pathway (Cell Growth)**	0	0	0
**cAMP Pathway (Cell Proliferation)**	-0.11756	-0.12944	-0.10956
**cAMP Pathway (Cell Survival)**	-0.02729	-0.03005	-0.02543
**cAMP Pathway (Cell Survival, Chemotaxis)**	0	0	0
**cAMP Pathway (Cytokine Production)**	0	0	0
**cAMP Pathway (Degradation of Cell Cycle Regulators)**	-0.0493	-0.05428	-0.04594
**cAMP Pathway (Endothelial Cell Regulation)**	-0.0568	-0.05295	-0.02392
**cAMP Pathway (Glycogen Synthesis)**	-0.13894	-0.15297	-0.12948
**cAMP Pathway (Glycolysis)**	-0.06947	-0.07649	-0.06474
**cAMP Pathway (Metabolic Energy)**	0	0	0
**cAMP Pathway (Myocardial Contraction)**	-0.00921	-0.01014	-0.00858
**cAMP Pathway (Oncogenesis)**	-0.10916	-0.12019	-0.10173
**cAMP Pathway (Protein Retention)**	-0.09552	-0.10517	-0.08902
**cAMP Pathway (Regulation of Cytoskeleton)**	-0.09552	-0.10517	-0.08902
**Caspase Cascade Main**	-0.02573	-0.01956	-0.01732
**Caspase Cascade (Activated Tissue Transglutaminase)**	0.023901	0.025823	0.019067
**Caspase Cascade (Apoptosis)**	0	0	0
**Caspase Cascade (Cell Survival)**	-0.10135	-0.08756	-0.06727
**Caspase Cascade (ICAD Degradation)**	0	0	0
**CD40 Main Pathway**	0.016502	0.010306	0.01201
**CD40 Pathway (Cell Survival)**	0	0	0
**CD40 Pathway (Gene Expression)**	0	0	0
**CD40 Pathway (IKBs Degradation)**	0.051449	0.03213	0.037444
**Cellular Anti Apoptosis Main Pathway**	0.052076	0.047117	0.033541
**Cellular Anti Apoptosis Pathway (Apoptosis)**	0.063907	0.068083	0.058041
**Cellular Anti Apoptosis Pathway (Depolarization)**	0	0	0
**Chemokine Main Pathway**	0.035365	0.025268	0.03126
**Chemokine Pathway (Cell Activation)**	0.034016	0.033145	0.01958
**Chemokine Pathway (Gene Expression, Apoptosis)**	0.04289	0.041791	0.024688
**Chemokine Pathway (Internalization, Degradation, Recycling)**	-0.11069	-0.10952	-0.06296
**Chromatin Main Pathway**	0	0	0
**Chromatin Pathway (Octamer Sliding)**	0	0	0
**Chromatin Pathway (Octamer Transfer)**	0	0	0
**Circadian Main Pathway**	-0.22928	-0.24828	-0.18258
**CREB Main Pathway**	0.026416	0.02162	0.018341

**CREB Pathway (Gene Expression Pathway)**	-0.04825	-0.04774	-0.02744
**Cytokine Main Pathway**	0.020186	0.027076	0.00076
**DDR pathway Apoptosis**	0	0	0
**DDR Main pathway**	0	0	0
**DNA Repair Mechanisms Pathway**	-0.01545	-0.01599	-0.01549
**EGFR Main Pathway**	0.085905	0.086062	0.065925
**ErbB Family Main Pathway**	0.098451	0.086394	0.07251
**ErbB Family Pathway (Anti-Apoptosis)**	0	0	0
**ERK Signaling Main Pathway**	0.056186	0.052776	0.039475
**Erythropoeitin Main Pathway**	-0.01897	-0.02756	-0.0075
**Estrogen Main Pathway**	0.030291	0.025923	0.024327
**Fas Signaling Pathway (Negative)**	0	0	0
**Fas Signaling Pathway (Positive)**	0.051449	0.03213	0.037444
**FLT3 Main Pathway**	0	0	0
**Glucocorticoid Receptor Main Pathway**	0.02024	0.019041	0.01156
**Glucocorticoid Receptor Pathway (Cell cycle arrest)**	0	0	0
**Glucocorticoid Receptor Pathway (Cell cycle progression)**	0.757473	0.633218	0.418275
**Glucocorticoid Receptor Pathway (Gene expression)**	0	0	0
**Glucocorticoid Receptor Pathway (Inflammatory cytokines)**	0.024784	0.024678	0.014402
**Glucocorticoid Receptor Pathway (SMAD signaling)**	0	0	0
**GPCR Main Pathway**	0.034355	0.029022	0.026709
**GPCR Pathway (Gene expression)**	0.106475	0.092441	0.074734
**Growth Hormone Main Pathway**	0.013735	-0.00534	0.002455
**Growth Hormone Pathway (Cell survival)**	0	0	0
**Growth Hormone Pathway (Gene expression)**	0.122883	0.103959	0.084418
**Growth Hormone Pathway (Glucose uptake)**	0.011798	-0.00459	0.002109
**Growth Hormone Pathway (Protein synthesis)**	0	0	0
**GSK3 Main Pathway**	0.018316	0.016851	0.011087
**GSK3 Pathway (Degradation)**	0	0	0
**GSK3 Pathway (Gene expression)**	0	0	0
**GSK3 Pathway (Translation)**	0	0	0
**G-protein Pathway (Ras family GTPases)**	0.196896	0.191677	0.151912
**Hedgehog Main Pathway**	-0.09423	-0.09325	-0.09726
**Hedgehog Pathway (Repression of Hh, BMP)**	0.135741	0.122394	0.091267
**Hedgehog Pathway (Activation of BMP, Ptc, WNT)**	0	0	0
**HGF Main Pathway**	0.09172	0.087376	0.067874
**HGF Pathway (Anoikis)**	0.16762	0.152614	0.136021
**HGF Pathway (Cell adhesion, cell mirgation)**	0.09222	0.094478	0.057013
**HGF Pathway (Cell cycle progression)**	0.558733	0.508713	0.453404
**HGF Pathway (Cell polarity, cell motility)**	0	0	0
**HGF Pathway (Cell scattering)**	0	0	0
**HGF Pathway (Cell survival)**	0	0	0
**HGF Pathway (IP3 pathway)**	0	0	0
**HGF Pathway (PKC pathway)**	0	0	0
**HIF1-Alpha Main Pathway**	0.017391	0.019901	0.009056
**HIF1Alpha Pathway (Gene expression)**	0.062607	0.071642	0.032601
**HIF1Alpha Pathway (HIF1alpha degradation)**	0.078259	0.089553	0.040752
**HIF1Alpha Pathway (NOS pathway)**	0.072239	0.082664	0.037617
**HIF1Alpha Pathway (p53 Hypoxia pathway)**	0.067079	0.07676	0.03493
**HIF1Alpha Pathway (Pyruvate)**	0.062607	0.071642	0.032601
**HIF1Alpha Pathway (VEGF pathway)**	0.058694	0.067165	0.030564

**Hypoxia pathway EMT 1**	0.231619	0.27409	0.182832
**Hypoxia pathway EMT 2**	0.231619	0.27409	0.182832
**Hypoxia pathway EMT 3**	0.231619	0.27409	0.182832
**Hypoxia pathway EMT 4**	0.231619	0.27409	0.182832
**IGF1R Main Pathway**	0.003746	0.006557	0.0083
**IGF1R Signaling Pathway (Cell survival)**	0	0	0
**IGF1R Signaling Pathway (Glucose uptake)**	0	0	0
**IGF1R Signaling Pathway (Glycogen synthesis)**	0	0	0
**IGF1R Signaling Pathway (IKB degradation)**	0	0	0
**IGF1R Signaling Pathway (Protein synthesis)**	0	0	0
**ILK Main Pathway**	0.107741	0.104742	0.081771
**ILK Pathway (Apoptosis)**	0.122901	0.127541	0.103698
**ILK Pathway (Cell adhesion, cell motility, opsonization)**	0.125862	0.129915	0.105295
**ILK Pathway (Cell cycle proliferation)**	0.113093	0.116735	0.094613
**ILK Pathway (Cell migration, retraction)**	0.123864	0.127853	0.103623
**ILK Pathway (Cell motility)**	0.111889	0.109386	0.087996
**ILK Pathway (Cytoskeletal reorganization)**	0.151909	0.151104	0.123295
**ILK Pathway (G2-phase arrest)**	0.113093	0.116735	0.094613
**ILK Pathway (Induced cell proliferation)**	0.229441	0.238619	0.167519
**ILK Pathway (Regulation of intermediate filaments)**	0.148455	0.145663	0.116131
**ILK Pathway (Regulation of junction assembly of desmosomes)**	0.124855	0.128876	0.104452
**ILK Pathway (Wound healing)**	0.124855	0.128876	0.104452
**IL-10 Main Pathway**	0.183347	0.180544	0.123506
**IL-10 Pathway (Gene expression)**	0.148535	0.15219	0.111218
**IL-10 Pathway (Stability determination)**	2.376553	2.435033	1.779481
**IL-10 Pathway (Translational modulation)**	0.211932	0.198636	0.184246
**IL-2 Main Pathway**	0.06565	0.055603	0.041244
**IL-2 Pathway (Actin reorganization)**	0.33567	0.371395	0.266575
**IL-2 Pathway (Apoptosis)**	0	0	0
**IL-2 Pathway (Apoptosis inhibition)**	0	0	0
**IL-2 Pathway (Protein synthesis)**	0.396701	0.438921	0.315043
**IL-6 Main Pathway**	0.065093	0.058318	0.036382
**Integrin SIgnaling Main Pathway**	0.109225	0.106039	0.08684
**Integrin SIgnaling Pathway (Cell survival)**	-0.01424	-0.00746	-0.01191
**Integrin SIgnaling Pathway (Cytoskeleton contraction integrin modulation cell invasion and migration)**	0.10229	0.081293	0.074973
**Integrin SIgnaling Pathway (Focal adhesion and stress fibers)**	0.012623	0.034765	-0.00179
**Integrin SIgnaling Pathway (Translocation to the nucleus)**	0	0	0
**Interactions Report**	0.302369	0.354145	0.213451
**Interferon Main Pathway**	0.00679	0.007875	0.01617
**Interferon Pathway (Gene expression)**	0	0	0
**Interferon Pathway (Transcription)**	0	0	0
**Interferon Pathway (Translation)**	0	0	0
**IP3 Main Pathway**	-0.01143	-0.01284	-0.00332
**IP3 Pathway (Gene expression)**	0	0	0
**JAK mStat Main Pathway**	0.074233	0.084048	0.067714
**JAK mStat Pathway (Akt pathway)**	-0.04522	-0.04659	-0.03254
**JAK mStat Pathway (JAK degradation)**	0	0	0
**JNK Main Pathway**	0.015707	0.013377	0.013132
**JNK Pathway (Apoptosis, Inflammation, Tumorigenesis, Cell Migration)**	0.104679	0.108962	0.084168
**JNK Pathway (Insulin signaling)**	-0.22924	-0.23859	-0.14005

**MAPK Family Main Pathway**	0.029247	0.027083	0.020488
**MAPK Family Pathway (Chromatin Remodeling)**	0.034016	0.033145	0.01958
**MAPK Family Pathway (Cytoskeleton)**	0.211779	0.221788	0.154623
**MAPK Family Pathway (Gene Expression)**	0.025294	0.024646	0.01456
**MAPK Family Pathway (IKBs Degradation)**	0.067279	0.042016	0.048965
**MAPK Family Pathway (Translation)**	0.382823	0.28289	0.204144
**MAPK Signaling Main Pathway**	0.087445	0.083015	0.064506
**MAPK Signaling Pathway (Cell Survival, Inflammation, Apoptosis, Osmoregulation)**	0.284746	0.275141	0.209317
**MAPK Signaling Pathway (Gene Expression)**	0.110553	0.111552	0.089557
**DDR pathway (MMR)**	-0.12073	-0.10513	-0.10084
**Mismatch Repair Main Pathway**	-0.07474	-0.06508	-0.06243
**Mitochondrial Apopotosis Main Pathway**	-0.05018	-0.0391	-0.02721
**Mitochondrial Apopotosis Pathway (Apoptosis)**	0	0	0
**Mitochondrial Apopotosis Pathway (Depolarization)**	0	0	0
**Mitochondrial Apopotosis Pathway (DNA fragmentation)**	-0.27028	-0.2335	-0.17938
**Mitochondrial Apopotosis Pathway (Gene expression)**	0	0	0
**mTOR Main Pathway**	0.05079	0.049236	0.033764
**mTOR Pathway (Actin organization)**	0.051586	0.047295	0.0433
**mTOR Pathway (Akt signaling)**	0	0	0
**mTOR Pathway (Scanning)**	0	0	0
**mTOR Pathway (Translation on)**	0	0	0
**mTOR Pathway (VEGF pathway)**	0.187822	0.214927	0.097804
**NGF (Negative) Main Pathway**	0	0	0
**NGF (Negative) Pathway (Apoptosis)**	0	0	0
**NGF (Positive) Main Pathway**	0.081429	0.07376	0.046901
**NHEJ mechanisms of DSBs repair effect**	0	0	0
**Notch Main Pathway**	-0.04923	-0.02989	-0.03784
**DDR Pathway (NER)**	0	0	0
**p38 (Negative) Main Signaling Pathway**	0.055762	0.048977	0.035163
**p38 (Positive) Main Signaling Pathway**	0.055649	0.048878	0.035092
**p53 Signaling (Negative) Main Pathway**	0.116383	0.113399	0.100632
**p53 Signaling (Negative) Pathway (p53 Degradation)**	0	0	0
**PAK Main Pathway**	0.052832	0.04783	0.030153
**PAK Pathway (Actin Cytoskeleton)**	0.029967	0.026913	0.015856
**PAK Pathway (Myosin Activation)**	0.050972	0.042942	0.0241
**PPAR Main Pathway**	0.074211	0.069649	0.045747
**PTEN Main Pathway**	0	0	0
**RANK Signaling in Osteoclast Main Pathway**	0.107012	0.090569	0.082585
**RANK Signaling in Osteoclast Pathway (IKBs Degradation)**	0.043732	0.02731	0.031827
**RAS Main Pathway**	0.012755	0.011392	0.011054
**Telomere Main Pathway**	0.095392	0.099053	0.057103
**RNA Polymerase II Complex Pathway**	0	0	0
**Cell Cycle Pathway (SCC during S-phase)**	0	0	0
**SMAD (Negative) Main Pathway**	0.180487	0.167123	0.129087
**SMAD (Negative) Pathway (Degradation)**	0	0	0
**SMAD (Positive) Main Pathway**	0.180487	0.167123	0.129087
**SMAD (Positive) Pathway (Degradation)**	0	0	0
**Cell Cycle Pathway (Metaphase-Anaphase)**	0	0	0
**Cell Cycle Pathway (Origin of S-phase)**	0	0	0
**STAT3 Main Pathway**	0.032294	0.026505	0.012099
**TGF beta Main Pathway**	0.032516	0.033963	0.019217

**TGF beta Pathway (Epithelial mesehchymal transition)**	0.205885	0.208848	0.14812
**TGF beta Pathway (Post-transcriptional G1 arrest)**	0.156472	0.158724	0.112571
**TGF beta Pathway (SnON degradation)**	0.315727	0.288661	0.279322
**TGF beta Pathway (Tumorigenesis)**	0.451039	0.412373	0.399031
**TGF beta Pathway (Tumor suppression)**	0.451039	0.412373	0.399031
**TNF (Negative) Main Pathway**	0.057304	0.065602	0.037686
**TNF (Negative) Pathway (Apoptosis)**	0	0	0
**TNF (Positive) Main Pathway**	0.210186	0.190281	0.15216
**TNF (Positive) Pathway (Gene expression, Cell survival)**	0.274129	0.237204	0.205733
**TNF (Positive) Pathway (IKBs degradation)**	0.048591	0.030345	0.035364
**TRAF (Negative) Main Pathway**	0	0	0
**TRAF (Negative) Pathway (IKBs Degradation)**	0.060488	0.069247	0.039779
**TRAF (Positive) Main Pathway**	0.100395	0.092166	0.075122
**TRAF (Positive) Pathway (IKBs Degradation)**	0.060488	0.069247	0.039779
**Transcription of mRNA Pathway**	0	0	0
**Cell Cycle Pathway (End of S-phase)**	0	0	0
**Translation Regulation of EIF4F activity**	0	0	0
**Ubiquitin Proteasome Main Pathway**	0.007071	0.006115	0.007322
**Ubiquitin Proteasome Pathway (Degraded Protein)**	0.019151	0.016562	0.01983
**VEGF Main Pathway**	0.075575	0.070814	0.052043
**VEGF Pathway (Actin Reorganization)**	0.148789	0.139415	0.10246
**VEGF Pathway (Nitric Oxide Production)**	0	0	0
**Wnt Main Pathway**	0.05914	0.058717	0.045303
**Wnt Pathway (Ctnn-b Degradation)**	0	0	0
**IL-6 Pathway (IKBs degradation)**	0	0	0

**Pathway**	**M_86_8h_1**	**M_86_8h_2**	**M_86_8h_3**	**p-value_Mean**
**AHR Main Pathway**	-0.08414	-0.08758	-0.0169	0.049535
**AHR Pathway (AHR Degradation)**	-0.17334	-0.19519	-0.1205	0.049535
**AHR Pathway (Cath-D Expression)**	-0.05316	-0.0675	0	0.19043
**AHR Pathway (C-MycExpression)**	-0.05004	-0.06353	0	0.19043
**AHR Pathway (PS2 Gene Expression)**	-0.15144	-0.15765	-0.03043	0.049535
**AKT Main Pathway**	0.013183	0.046792	0.029352	0.049535
**AKT Pathway (Aggregation & Neurodegeneration)**	-0.03339	0.008106	-0.05243	0.512691
**AKT Pathway (Apoptosis Inhibition)**	-0.03208	0.007788	-0.05037	0.512691
**AKT Pathway (Blocks Apoptosis)**	0.035658	0.057673	0.040319	0.049535
**AKT Pathway (Cardiovascular Homeostasis)**	-0.03636	0.008827	-0.05709	0.512691
**AKT Pathway (Caspase Cascade)**	-0.03228	0.001684	-0.03815	0.275234
**AKT Pathway (Cell Cycle)**	0	0	0	1
**AKT Pathway (Cell Cycle Progression)**	-0.12354	-0.09628	-0.14499	0.049535
**AKT Pathway (Cell Survival)**	-0.48254	-0.33015	-0.32349	0.049535
**AKT Pathway (Death Genes)**	-0.05645	-0.01755	-0.06144	0.049535
**AKT Pathway (Elevation of Glucose Import)**	-0.05348	-0.01662	-0.05821	0.049535
**AKT Pathway (ERK Pathway)**	-0.02464	-0.00964	-0.02527	0.049535
**AKT Pathway (Genetic Stability)**	-0.08785	-0.05282	-0.085	0.049535

**AKT Pathway (Glucose Uptake)**	-0.03719	0.009027	-0.05839	0.512691
**AKT Pathway (Glycogen Synthesis)**	-0.1185	-0.08621	-0.09364	0.049535
**AKT Pathway (Insulin Stimulated Mitogenesis)**	-0.03636	0.008827	-0.05709	0.512691
**AKT Pathway (JNK Pathway)**	-0.05645	-0.01755	-0.06144	0.049535
**AKT Pathway (Neuroprotection)**	-0.03636	0.008827	-0.05709	0.512691
**AKT Pathway (NF-kB Pathway)**	-0.03409	0.008275	-0.05352	0.512691
**AKT Pathway (p53 Degradation)**	0	0	0	1
**AKT Pathway (p73 Mediated Apoptosis)**	-0.05806	-0.01805	-0.0632	0.049535
**AKT Pathway (Protein Synthesis)**	0	0	0	1
**AKT Pathway (Regeneration of Cyclic Nucleotide)**	-0.1101	-0.06528	-0.10045	0.049535
**AKT Pathway (Respiratory Burst)**	-0.03636	0.008827	-0.05709	0.512691
**AKT Pathway (Survival Genes)**	-0.0219	-0.00857	-0.02246	0.049535
**AKT Pathway (Synaptic Signaling)**	-0.0102	0.009061	-0.02156	0.512691
**AKT Pathway (Translation)**	0	0	0	1
**Androgen Receptor Pathway**	0.045031	0.069187	0.063658	0.049535
**Androgen Receptor Pathway (Apoptosis)**	-0.09605	-0.0267	-0.10703	0.049535
**Androgen Receptor Pathway (Degradation)**	-0.01009	0.014012	0.027302	0.512691
**Androgen Receptor Pathway (Cell Survival & Cell Growth)**	-0.01464	-0.01373	-0.0078	0.049535
**Androgen Receptor Pathway (Gonadotropin Regulation)**	0.160787	0.122426	0.214704	0.049535
**Androgen Receptor Pathway (Histone Modification)**	0.160787	0.122426	0.214704	0.049535
**Androgen Receptor Pathway (Prostate Differentiation & Development)**	0.160787	0.122426	0.214704	0.049535
**Androgen Receptor Pathway (Sexual Differentiation & Sexual Maturation at Puberty)**	0.160787	0.122426	0.214704	0.049535
**ATM Main Pathway**	0.180829	0.161742	0.189068	0.049535
**ATM Pathway (Apoptosis)**	-0.3079	-0.26497	-0.35542	0.049535
**ATM Pathway (Apoptosis, Senescense)**	0.079299	-0.09852	-0.05919	0.512691
**ATM Pathway (Cell Cicle Checkpoint Control)**	-0.35122	-0.27695	-0.39621	0.049535
**ATM Pathway (Cell Survival)**	-0.03108	-0.03832	-0.01511	0.275234
**ATM Pathway (Checkpoint Activation)**	-0.35122	-0.27695	-0.39621	0.049535
**ATM Pathway (DNA Repair)**	-0.26205	-0.14258	-0.32267	0.049535
**ATM Pathway (G2_M Checkpoint Arrest)**	0.809624	0.674804	0.844752	0.049535
**ATM Pathway (G2 Mitosis Progression)**	0.557977	0.198405	0.406487	0.049535
**ATM Pathway (MDMX Ubiquitination, Degradation)**	0.261641	-0.00026	0.111937	0.512691
**ATM Pathway (NF-kB Pathway)**	-0.35122	-0.27695	-0.39621	0.049535
**ATM Pathway (Synaptic Vesicle Transport)**	-0.35122	-0.27695	-0.39621	0.049535
**ATM Pathway (S-Phase Arrest)**	-0.35122	-0.27695	-0.39621	0.049535
**ATM Pathway (S-Phase Progression)**	-0.11407	-0.14306	-0.221	0.049535
**DDR Pathway (BRCA1-induced responses)**	0.017991	0.097605	0.044216	0.049535
**BRCA1 Main Pathway**	-0.24159	-0.21168	-0.25984	0.049535
**cAMP Main Pathway**	0.028785	0.043351	0.038407	0.049535
**cAMP Pathway (Axonal Growth)**	-0.07413	-0.1062	-0.08176	0.049535
**cAMP Pathway (Cardiovascular Homeostasis)**	-0.15884	-0.22758	-0.1752	0.049535
**cAMP Pathway (Cell Growth)**	0	0	0	1
**cAMP Pathway (Cell Proliferation)**	-0.17106	-0.24508	-0.18868	0.049535
**cAMP Pathway (Cell Survival)**	-0.0964	-0.09545	-0.08691	0.049535
**cAMP Pathway (Cell Survival, Chemotaxis)**	0	0	0	1
**cAMP Pathway (Cytokine Production)**	0	0	0	1
**cAMP Pathway (Degradation of Cell Cycle Regulators)**	-0.07173	-0.10278	-0.07912	0.049535
**cAMP Pathway (Endothelial Cell Regulation)**	0.283086	0.342552	0.28436	0.049535
**cAMP Pathway (Glycogen Synthesis)**	-0.20216	-0.28965	-0.22299	0.049535
**cAMP Pathway (Glycolysis)**	-0.16332	-0.2076	-0.16769	0.049535

**cAMP Pathway (Metabolic Energy)**	-0.09605	-0.0267	-0.10703	0.049535
**cAMP Pathway (Myocardial Contraction)**	-0.07195	-0.05858	-0.06893	0.049535
**cAMP Pathway (Oncogenesis)**	-0.15884	-0.22758	-0.1752	0.049535
**cAMP Pathway (Protein Retention)**	0.08974	0.049681	0.08063	0.275234
**cAMP Pathway (Regulation of Cytoskeleton)**	-0.13899	-0.19913	-0.1533	0.049535
**Caspase Cascade Main**	-0.04271	-0.05533	-0.04146	0.049535
**Caspase Cascade (Activated Tissue Transglutaminase)**	-0.0052	-0.0003	0.007137	0.512691
**Caspase Cascade (Apoptosis)**	-0.00691	0.003892	-0.00419	0.512691
**Caspase Cascade (Cell Survival)**	-0.14145	-0.11332	-0.16159	0.049535
**Caspase Cascade (ICAD Degradation)**	-0.03337	-0.02461	-0.03267	0.049535
**CD40 Main Pathway**	-0.05352	-0.03174	-0.06558	0.049535
**CD40 Pathway (Cell Survival)**	0	0	0	1
**CD40 Pathway (Gene Expression)**	0.040789	0.026418	0.012553	0.049535
**CD40 Pathway (IKBs Degradation)**	0.028792	0.018648	0.008861	0.049535
**Cellular Anti Apoptosis Main Pathway**	0.056795	0.073763	0.06358	0.049535
**Cellular Anti Apoptosis Pathway (Apoptosis)**	0.130172	0.078577	0.123438	0.049535
**Cellular Anti Apoptosis Pathway (Depolarization)**	0	0	0	1
**Chemokine Main Pathway**	0.022471	0.02276	0.034229	0.049535
**Chemokine Pathway (Cell Activation)**	0.096683	0.077419	0.120836	0.049535
**Chemokine Pathway (Gene Expression, Apoptosis)**	0.054581	0.048613	0.066024	0.049535
**Chemokine Pathway (Internalization, Degradation, Recycling)**	-0.13405	-0.15801	-0.13011	0.049535
**Chromatin Main Pathway**	-0.03304	-0.02026	-0.02488	0.049535
**Chromatin Pathway (Octamer Sliding)**	-0.03812	-0.02338	-0.02871	0.049535
**Chromatin Pathway (Octamer Transfer)**	-0.03304	-0.02026	-0.02488	0.049535
**Circadian Main Pathway**	-0.20616	-0.10279	-0.17949	0.049535
**CREB Main Pathway**	0.033258	0.055116	0.036187	0.049535
**CREB Pathway (Gene Expression Pathway)**	-0.05843	-0.06888	-0.05672	0.049535
**Cytokine Main Pathway**	0.092406	0.108287	0.132131	0.049535
**DDR pathway Apoptosis**	-0.37414	-0.21506	-0.38036	0.049535
**DDR Main pathway**	-0.74823	-0.42454	-0.69824	0.049535
**DNA Repair Mechanisms Pathway**	-0.05048	-0.0528	-0.05563	0.049535
**EGFR Main Pathway**	0.006442	0.010011	0.024908	0.275234
**ErbB Family Main Pathway**	0.112328	0.123333	0.137386	0.049535
**ErbB Family Pathway (Anti-Apoptosis)**	0	0	0	1
**ERK Signaling Main Pathway**	0.038167	0.062466	0.052338	0.049535
**Erythropoeitin Main Pathway**	-0.00224	-0.01557	0.00777	0.827259
**Estrogen Main Pathway**	0.062006	0.069593	0.06927	0.049535
**Fas Signaling Pathway (Negative)**	0.030197	0.008527	0.053989	0.12663
**Fas Signaling Pathway (Positive)**	0	0	0	1
**FLT3 Main Pathway**	-0.04798	-0.02158	-0.0438	0.049535
**Glucocorticoid Receptor Main Pathway**	0.090038	0.089999	0.121325	0.049535
**Glucocorticoid Receptor Pathway (Cell cycle arrest)**	1.340698	0.93413	1.600081	0.049535
**Glucocorticoid Receptor Pathway (Cell cycle progression)**	0.149054	0.113761	0.518191	0.049535
**Glucocorticoid Receptor Pathway (Gene expression)**	0.081101	0.059961	0.072647	0.049535
**Glucocorticoid Receptor Pathway (Inflammatory cytokines)**	0.078939	0.094345	0.129468	0.049535
**Glucocorticoid Receptor Pathway (SMAD signaling)**	0.176008	0.252994	0.106479	0.049535
**GPCR Main Pathway**	0.05237	0.070663	0.056953	0.049535
**GPCR Pathway (Gene expression)**	0.125216	0.084649	0.13914	0.049535
**Growth Hormone Main Pathway**	0.01734	0.018475	0.026888	0.049535
**Growth Hormone Pathway (Cell survival)**	0	0	0	1
**Growth Hormone Pathway (Gene expression)**	0.080855	0.086787	0.106565	0.049535

**Growth Hormone Pathway (Glucose uptake)**	0.014894	0.015869	0.023096	0.049535
**Growth Hormone Pathway (Protein synthesis)**	0	0	0	1
**GSK3 Main Pathway**	0.024268	0.050807	0.02443	0.049535
**GSK3 Pathway (Degradation)**	0.031526	0.032367	0.037078	0.049535
**GSK3 Pathway (Gene expression)**	0.044212	0.053583	0.044309	0.049535
**GSK3 Pathway (Translation)**	0.054361	0.030213	0.043732	0.049535
**G-protein Pathway (Ras family GTPases)**	0	0	0	1
**Hedgehog Main Pathway**	-0.2179	-0.18263	-0.18831	0.049535
**Hedgehog Pathway (Repression of Hh, BMP)**	0.449774	0.416245	0.366414	0.049535
**Hedgehog Pathway (Activation of BMP, Ptc, WNT)**	0	0	0	1
**HGF Main Pathway**	0.050282	0.064892	0.089614	0.049535
**HGF Pathway (Anoikis)**	0.08366	0.078413	0.132223	0.049535
**HGF Pathway (Cell adhesion, cell mirgation)**	0.093349	0.154263	0.124611	0.049535
**HGF Pathway (Cell cycle progression)**	0.609356	0.461843	0.838736	0.049535
**HGF Pathway (Cell polarity, cell motility)**	0	0	0	1
**HGF Pathway (Cell scattering)**	-0.17325	-0.12131	-0.18029	0.049535
**HGF Pathway (Cell survival)**	-0.17325	-0.12131	-0.18029	0.049535
**HGF Pathway (IP3 pathway)**	-0.19924	-0.13951	-0.20734	0.049535
**HGF Pathway (PKC pathway)**	0	0	0	1
**HIF1-Alpha Main Pathway**	-0.08634	-0.02714	-0.03043	0.049535
**HIF1Alpha Pathway (Gene expression)**	0.139869	0.125456	0.197164	0.049535
**HIF1Alpha Pathway (HIF1alpha degradation)**	0.067347	0.089324	0.100255	0.049535
**HIF1Alpha Pathway (NOS pathway)**	0.161388	0.144757	0.227497	0.049535
**HIF1Alpha Pathway (p53 Hypoxia pathway)**	0.057726	0.076564	0.085932	0.049535
**HIF1Alpha Pathway (Pyruvate)**	0.224645	0.270052	0.24716	0.049535
**HIF1Alpha Pathway (VEGF pathway)**	0.131127	0.117615	0.184842	0.049535
**Hypoxia pathway EMT 1**	0.779536	0.96196	0.872569	0.049535
**Hypoxia pathway EMT 2**	0.779536	0.96196	0.872569	0.049535
**Hypoxia pathway EMT 3**	0.779536	0.96196	0.872569	0.049535
**Hypoxia pathway EMT 4**	0.779536	0.96196	0.872569	0.049535
**IGF1R Main Pathway**	-0.02564	-0.01541	-0.01842	0.049535
**IGF1R Signaling Pathway (Cell survival)**	0.056943	0.039791	0.061636	0.049535
**IGF1R Signaling Pathway (Glucose uptake)**	0	0	0	1
**IGF1R Signaling Pathway (Glycogen synthesis)**	0.207381	0.150865	0.163862	0.049535
**IGF1R Signaling Pathway (IKB degradation)**	0.226049	0.118426	0.214	0.049535
**IGF1R Signaling Pathway (Protein synthesis)**	0.234072	0.183615	0.154723	0.049535
**ILK Main Pathway**	0.106573	0.144427	0.12539	0.049535
**ILK Pathway (Apoptosis)**	0.070725	0.120693	0.090518	0.049535
**ILK Pathway (Cell adhesion, cell motility, opsonization)**	0.06273	0.111977	0.083962	0.049535
**ILK Pathway (Cell cycle proliferation)**	0.045925	0.097715	0.06381	0.049535
**ILK Pathway (Cell migration, retraction)**	0.055294	0.106355	0.075959	0.049535
**ILK Pathway (Cell motility)**	0.097299	0.143862	0.105284	0.049535
**ILK Pathway (Cytoskeletal reorganization)**	0.147408	0.201531	0.164655	0.049535
**ILK Pathway (G2-phase arrest)**	0.045925	0.097715	0.06381	0.049535
**ILK Pathway (Induced cell proliferation)**	0.063658	0.11788	0.103846	0.049535
**ILK Pathway (Regulation of intermediate filaments)**	0.087768	0.133471	0.103907	0.049535
**ILK Pathway (Regulation of junction assembly of desmosomes)**	0.062228	0.111082	0.083291	0.049535
**ILK Pathway (Wound healing)**	0.072551	0.123013	0.091753	0.049535
**IL-10 Main Pathway**	0.115188	0.119186	0.149787	0.049535
**IL-10 Pathway (Gene expression)**	0.178893	0.160445	0.195871	0.049535
**IL-10 Pathway (Stability determination)**	2.474941	2.57804	2.652107	0.049535

**IL-10 Pathway (Translational modulation)**	0.192497	0.20687	0.23378	0.049535
**IL-2 Main Pathway**	0.055033	0.054159	0.078474	0.049535
**IL-2 Pathway (Actin reorganization)**	-0.23409	-0.17381	-0.17158	0.049535
**IL-2 Pathway (Apoptosis)**	-0.0481	0.060382	0.010631	0.827259
**IL-2 Pathway (Apoptosis inhibition)**	-0.05363	0.088436	0.029062	0.512691
**IL-2 Pathway (Protein synthesis)**	-0.27666	-0.20541	-0.20277	0.049535
**IL-6 Main Pathway**	0.084696	0.091561	0.090675	0.049535
**Integrin SIgnaling Main Pathway**	0.056259	0.090581	0.073109	0.049535
**Integrin SIgnaling Pathway (Cell survival)**	-0.10511	-0.05286	-0.08859	0.049535
**Integrin SIgnaling Pathway (Cytoskeleton contraction integrin modulation cell invasion and migration)**	0.054567	0.042015	0.088494	0.049535
**Integrin SIgnaling Pathway (Focal adhesion and stress fibers)**	-0.13931	-0.13175	-0.13543	0.049535
**Integrin SIgnaling Pathway (Translocation to the nucleus)**	0.375556	0.367713	0.410091	0.049535
**Interactions Report**	0.782399	0.972953	0.905617	0.049535
**Interferon Main Pathway**	-0.02631	-0.01757	-0.02184	0.049535
**Interferon Pathway (Gene expression)**	-0.05569	-0.04398	-0.04324	0.049535
**Interferon Pathway (Transcription)**	0	0	0	1
**Interferon Pathway (Translation)**	0.227036	0.215387	0.126676	0.049535
**IP3 Main Pathway**	-0.03223	-0.01181	-0.02672	0.049535
**IP3 Pathway (Gene expression)**	0.03209	0.034256	0.036421	0.049535
**JAK mStat Main Pathway**	0.024432	0.013239	0.022453	0.12663
**JAK mStat Pathway (Akt pathway)**	-0.09984	-0.06061	-0.09681	0.049535
**JAK mStat Pathway (JAK degradation)**	-0.02732	-0.02805	-0.03213	0.049535
**JNK Main Pathway**	0.015084	0.049531	0.030544	0.049535
**JNK Pathway (Apoptosis, Inflammation, Tumorigenesis, Cell Migration)**	0.096517	0.119968	0.129748	0.049535
**JNK Pathway (Insulin signaling)**	-0.41716	-0.36377	-0.42094	0.049535
**MAPK Family Main Pathway**	0.04129	0.062049	0.064613	0.049535
**MAPK Family Pathway (Chromatin Remodeling)**	0.094157	0.080437	0.114516	0.049535
**MAPK Family Pathway (Cytoskeleton)**	0.172696	0.147182	0.215003	0.049535
**MAPK Family Pathway (Gene Expression)**	0.075308	0.031432	0.092238	0.049535
**MAPK Family Pathway (IKBs Degradation)**	0.260826	0.136646	0.246923	0.049535
**MAPK Family Pathway (Translation)**	0	0	0	1
**MAPK Signaling Main Pathway**	0.063097	0.088731	0.080798	0.049535
**MAPK Signaling Pathway (Cell Survival, Inflammation, Apoptosis, Osmoregulation)**	0.241793	0.276547	0.304633	0.049535
**MAPK Signaling Pathway (Gene Expression)**	0.094873	0.100049	0.129592	0.049535
**DDR pathway (MMR)**	-0.22779	-0.15958	-0.22601	0.049535
**Mismatch Repair Main Pathway**	-0.20019	-0.14658	-0.20471	0.049535
**Mitochondrial Apopotosis Main Pathway**	-0.06772	-0.0909	-0.06584	0.049535
**Mitochondrial Apopotosis Pathway (Apoptosis)**	0.179404	0.150505	0.216033	0.049535
**Mitochondrial Apopotosis Pathway (Depolarization)**	0	0	0	1
**Mitochondrial Apopotosis Pathway (DNA fragmentation)**	-0.37719	-0.3022	-0.43092	0.049535
**Mitochondrial Apopotosis Pathway (Gene expression)**	0.631122	0.317708	0.538441	0.049535
**mTOR Main Pathway**	0.075653	0.087187	0.072923	0.049535
**mTOR Pathway (Actin organization)**	-0.00922	0.017175	-0.00096	0.512691
**mTOR Pathway (Akt signaling)**	0	0	0	1
**mTOR Pathway (Scanning)**	0.134055	0.111978	0.085044	0.049535
**mTOR Pathway (Translation on)**	0.063354	0.039977	0.037505	0.049535
**mTOR Pathway (VEGF pathway)**	0.161633	0.214378	0.240611	0.049535
**NGF (Negative) Main Pathway**	0.132001	0.034662	0.113354	0.049535
**NGF (Negative) Pathway (Apoptosis)**	0.18277	0.047993	0.156952	0.049535
**NGF (Positive) Main Pathway**	0.001175	-0.01962	-0.02804	0.275234

**NHEJ mechanisms of DSBs repair effect**	-0.33444	-0.23468	-0.32633	0.049535
**Notch Main Pathway**	-0.12594	-0.09367	-0.1277	0.049535
**DDR Pathway (NER)**	-0.03883	-0.03136	-0.04253	0.049535
**p38 (Negative) Main Signaling Pathway**	0.033539	0.056873	0.049941	0.049535
**p38 (Positive) Main Signaling Pathway**	0.033471	0.056758	0.04984	0.049535
**p53 Signaling (Negative) Main Pathway**	0.145089	0.146864	0.195515	0.049535
**p53 Signaling (Negative) Pathway (p53 Degradation)**	-0.03153	-0.03237	-0.03708	0.049535
**PAK Main Pathway**	0.095815	0.116389	0.101465	0.049535
**PAK Pathway (Actin Cytoskeleton)**	0.036041	0.087419	0.066666	0.049535
**PAK Pathway (Myosin Activation)**	0.06101	0.073826	0.061284	0.049535
**PPAR Main Pathway**	0.087572	0.117996	0.108546	0.049535
**PTEN Main Pathway**	0.082057	0.064895	0.093979	0.049535
**RANK Signaling in Osteoclast Main Pathway**	0.15937	0.167022	0.155451	0.049535
**RANK Signaling in Osteoclast Pathway (IKBs Degradation)**	0.169537	0.08882	0.1605	0.049535
**RAS Main Pathway**	-0.03501	-0.01228	-0.02147	0.049535
**Telomere Main Pathway**	-0.09369	-0.09577	-0.11635	0.049535
**RNA Polymerase II Complex Pathway**	0.006981	0.003504	-0.00746	0.512691
**Cell Cycle Pathway (SCC during S-phase)**	0	0	0	1
**SMAD (Negative) Main Pathway**	0.243757	0.247588	0.255446	0.049535
**SMAD (Negative) Pathway (Degradation)**	0.042269	0.029222	0.050089	0.049535
**SMAD (Positive) Main Pathway**	0.243757	0.247588	0.255446	0.049535
**SMAD (Positive) Pathway (Degradation)**	0.042269	0.029222	0.050089	0.049535
**Cell Cycle Pathway (Metaphase-Anaphase)**	0.163385	0.178416	0.159228	0.049535
**Cell Cycle Pathway (Origin of S-phase)**	-0.11153	-0.10491	-0.1011	0.049535
**STAT3 Main Pathway**	0.040093	0.071864	0.072365	0.049535
**TGF beta Main Pathway**	0.043595	0.07223	0.064726	0.049535
**TGF beta Pathway (Epithelial mesehchymal transition)**	0.181214	0.259453	0.156587	0.049535
**TGF beta Pathway (Post-transcriptional G1 arrest)**	0.190064	0.190146	0.176001	0.049535
**TGF beta Pathway (SnON degradation)**	0.42667	0.314055	0.54749	0.049535
**TGF beta Pathway (Tumorigenesis)**	0.609529	0.44865	0.782129	0.049535
**TGF beta Pathway (Tumor suppression)**	0.609529	0.44865	0.782129	0.049535
**TNF (Negative) Main Pathway**	0.103739	0.062985	0.145959	0.049535
**TNF (Negative) Pathway (Apoptosis)**	0.061952	0.013873	0.067804	0.049535
**TNF (Positive) Main Pathway**	0.262542	0.194654	0.316919	0.049535
**TNF (Positive) Pathway (Gene expression, Cell survival)**	0.379275	0.270525	0.44051	0.049535
**TNF (Positive) Pathway (IKBs degradation)**	0.188375	0.098689	0.178333	0.049535
**TRAF (Negative) Main Pathway**	0.015551	-0.05119	0.014581	0.512691
**TRAF (Negative) Pathway (IKBs Degradation)**	0.239367	0.152071	0.268363	0.049535
**TRAF (Positive) Main Pathway**	0.063845	0.048284	0.0892	0.049535
**TRAF (Positive) Pathway (IKBs Degradation)**	0.239367	0.152071	0.268363	0.049535
**Transcription of mRNA Pathway**	0.022396	0.017653	0.016719	0.049535
**Cell Cycle Pathway (End of S-phase)**	-0.06027	-0.06233	-0.04944	0.049535
**Translation Regulation of EIF4F activity**	-0.0962	-0.09364	-0.08197	0.049535
**Ubiquitin Proteasome Main Pathway**	-0.02813	-0.0251	-0.01459	0.049535
**Ubiquitin Proteasome Pathway (Degraded Protein)**	-0.03039	-0.03128	-0.00195	0.049535
**VEGF Main Pathway**	0.022625	0.057237	0.015176	0.049535
**VEGF Pathway (Actin Reorganization)**	0.168876	0.248556	0.152006	0.049535
**VEGF Pathway (Nitric Oxide Production)**	0	0	0	1
**Wnt Main Pathway**	0.033954	0.048929	0.03941	0.049535
**Wnt Pathway (Ctnn-b Degradation)**	-0.03513	-0.03607	-0.04132	0.049535
**IL-6 Pathway (IKBs degradation)**	0.282562	0.148033	0.2675	0.049535

Borger et al
Supplemental Data

Pathway	M_86_16h_1	M_86_16h_2	M_86_16h_3	p-value_Mean
**AHR Main Pathway**	0	0	0	1
**AHR Pathway (AHR Degradation)**	0	0	0	1
**AHR Pathway (Cath-D Expression)**	0	0	0	1
**AHR Pathway (C-MycExpression)**	0	0	0	1
**AHR Pathway (PS2 Gene Expression)**	0	0	0	1
**AKT Main Pathway**	0.040622	0.022564	0.039138	0.049535
**AKT Pathway (Aggregation & Neurodegeneration)**	0.07901	0.077393	0.096023	0.049535
**AKT Pathway (Apoptosis Inhibition)**	0.075911	0.074358	0.092257	0.049535
**AKT Pathway (Blocks Apoptosis)**	0	0	0	1
**AKT Pathway (Cardiovascular Homeostasis)**	0.086033	0.084273	0.104558	0.049535
**AKT Pathway (Caspase Cascade)**	0.040163	0.049035	0.058188	0.049535
**AKT Pathway (Cell Cycle)**	0.166657	0.103037	0.114533	0.049535
**AKT Pathway (Cell Cycle Progression)**	-0.06681	-0.06486	-0.04931	0.049535
**AKT Pathway (Cell Survival)**	0	0	0	1
**AKT Pathway (Death Genes)**	0.01666	0.024548	0.037489	0.049535
**AKT Pathway (Elevation of Glucose Import)**	0.015783	0.023256	0.035516	0.049535
**AKT Pathway (ERK Pathway)**	0	0	0	1
**AKT Pathway (Genetic Stability)**	0.016209	0.023885	0.036476	0.049535
**AKT Pathway (Glucose Uptake)**	0.087988	0.086188	0.106934	0.049535
**AKT Pathway (Glycogen Synthesis)**	0	0	0	1
**AKT Pathway (Insulin Stimulated Mitogenesis)**	0.086033	0.084273	0.104558	0.049535
**AKT Pathway (JNK Pathway)**	0.01666	0.024548	0.037489	0.049535
**AKT Pathway (Neuroprotection)**	0.086033	0.084273	0.104558	0.049535
**AKT Pathway (NF-kB Pathway)**	0.080656	0.079006	0.098023	0.049535
**AKT Pathway (p53 Degradation)**	0	0	0	1
**AKT Pathway (p73 Mediated Apoptosis)**	0	0	0	1
**AKT Pathway (Protein Synthesis)**	0	0	0	1
**AKT Pathway (Regeneration of Cyclic Nucleotide)**	-0.00426	0.002571	0.018483	0.512691
**AKT Pathway (Respiratory Burst)**	0.086033	0.084273	0.104558	0.049535
**AKT Pathway (Survival Genes)**	0	0	0	1
**AKT Pathway (Synaptic Signaling)**	0.012761	0.018803	0.028715	0.049535
**AKT Pathway (Translation)**	0	0	0	1
**Androgen Receptor Pathway**	0.006292	0.003225	0.025462	0.049535
**Androgen Receptor Pathway (Apoptosis)**	0.039983	0.058915	0.089975	0.049535
**Androgen Receptor Pathway (Degradation)**	-0.05404	-0.06835	-0.03417	0.049535
**Androgen Receptor Pathway (Cell Survival & Cell Growth)**	-0.0059	-0.08147	-0.03343	0.12663
**Androgen Receptor Pathway (Gonadotropin Regulation)**	-0.03066	-0.06729	-0.06318	0.049535
**Androgen Receptor Pathway (Histone Modification)**	-0.03066	-0.06729	-0.06318	0.049535
**Androgen Receptor Pathway (Prostate Differentiation & Development)**	-0.03066	-0.06729	-0.06318	0.049535
**Androgen Receptor Pathway (Sexual Differentiation & Sexual Maturation at Puberty)**	-0.03066	-0.06729	-0.06318	0.049535
**ATM Main Pathway**	0.089436	0.074042	0.072212	0.049535
**ATM Pathway (Apoptosis)**	0	0	0	1
**ATM Pathway (Apoptosis, Senescense)**	0	0	0	1
**ATM Pathway (Cell Cicle Checkpoint Control)**	0	0	0	1
**ATM Pathway (Cell Survival)**	0.105612	0.081326	0.101959	0.049535
**ATM Pathway (Checkpoint Activation)**	0	0	0	1

**ATM Pathway (DNA Repair)**	0	0	0	1
**ATM Pathway (G2_M Checkpoint Arrest)**	0.238121	0.205464	0.169506	0.049535
**ATM Pathway (G2 Mitosis Progression)**	0	0	0	1
**ATM Pathway (MDMX Ubiquitination, Degradation)**	0	0	0	1
**ATM Pathway (NF-kB Pathway)**	0	0	0	1
**ATM Pathway (Synaptic Vesicle Transport)**	0	0	0	1
**ATM Pathway (S-Phase Arrest)**	0	0	0	1
**ATM Pathway (S-Phase Progression)**	0	0	0	1
**DDR Pathway (BRCA1-induced responses)**	0.078804	0.171514	0.141939	0.049535
**BRCA1 Main Pathway**	-0.05963	-0.07286	-0.06117	0.049535
**cAMP Main Pathway**	0.007687	0.005024	0.01512	0.12663
**cAMP Pathway (Axonal Growth)**	-0.05118	-0.07303	-0.06731	0.049535
**cAMP Pathway (Cardiovascular Homeostasis)**	-0.10967	-0.1565	-0.14423	0.049535
**cAMP Pathway (Cell Growth)**	0	0	0	1
**cAMP Pathway (Cell Proliferation)**	-0.1181	-0.16854	-0.15532	0.049535
**cAMP Pathway (Cell Survival)**	0.022031	0.008514	0.013984	0.275234
**cAMP Pathway (Cell Survival, Chemotaxis)**	0	0	0	1
**cAMP Pathway (Cytokine Production)**	0.226311	0.174269	0.218484	0.049535
**cAMP Pathway (Degradation of Cell Cycle Regulators)**	-0.04953	-0.07068	-0.06514	0.049535
**cAMP Pathway (Endothelial Cell Regulation)**	0.328027	0.352236	0.328286	0.049535
**cAMP Pathway (Glycogen Synthesis)**	-0.13958	-0.19918	-0.18357	0.049535
**cAMP Pathway (Glycolysis)**	-0.14325	-0.19026	-0.17632	0.049535
**cAMP Pathway (Metabolic Energy)**	0.039983	0.058915	0.089975	0.049535
**cAMP Pathway (Myocardial Contraction)**	-0.03267	-0.03037	-0.02482	0.049535
**cAMP Pathway (Oncogenesis)**	-0.10967	-0.1565	-0.14423	0.049535
**cAMP Pathway (Protein Retention)**	-0.09596	-0.13693	-0.1262	0.049535
**cAMP Pathway (Regulation of Cytoskeleton)**	-0.09596	-0.13693	-0.1262	0.049535
**Caspase Cascade Main**	-0.01228	-0.00606	-0.02755	0.049535
**Caspase Cascade (Activated Tissue Transglutaminase)**	0.010974	0.001669	0.006787	0.512691
**Caspase Cascade (Apoptosis)**	-0.02511	-0.02569	-0.02062	0.049535
**Caspase Cascade (Cell Survival)**	0	0	0	1
**Caspase Cascade (ICAD Degradation)**	-0.02735	-0.02798	-0.02246	0.049535
**CD40 Main Pathway**	0.008036	-0.0248	0.008748	0.512691
**CD40 Pathway (Cell Survival)**	0	0	0	1
**CD40 Pathway (Gene Expression)**	0	0	0	1
**CD40 Pathway (IKBs Degradation)**	0	0	0	1
**Cellular Anti Apoptosis Main Pathway**	0.040902	0.020485	0.044266	0.049535
**Cellular Anti Apoptosis Pathway (Apoptosis)**	0.119613	0.087404	0.078597	0.049535
**Cellular Anti Apoptosis Pathway (Depolarization)**	0	0	0	1
**Chemokine Main Pathway**	0.023605	0.001006	0.017467	0.12663
**Chemokine Pathway (Cell Activation)**	0.054627	0.042065	0.052737	0.049535
**Chemokine Pathway (Gene Expression, Apoptosis)**	0.068877	0.053038	0.066495	0.049535
**Chemokine Pathway (Internalization, Degradation, Recycling)**	0.001816	-0.02092	0.037159	0.512691
**Chromatin Main Pathway**	0	0	0	1
**Chromatin Pathway (Octamer Sliding)**	0	0	0	1
**Chromatin Pathway (Octamer Transfer)**	0	0	0	1
**Circadian Main Pathway**	0.102311	0.112881	0.124641	0.049535
**CREB Main Pathway**	0.019788	0.014197	0.039527	0.049535
**CREB Pathway (Gene Expression Pathway)**	0.073689	0.062698	0.088345	0.049535
**Cytokine Main Pathway**	-0.04517	-0.058	-0.03285	0.049535
**DDR pathway Apoptosis**	0	0	0	1

**DDR Main pathway**	0	0	0	1
**DNA Repair Mechanisms Pathway**	-0.00107	0.010361	0.010752	0.12663
**EGFR Main Pathway**	-0.00955	-0.01166	-0.00432	0.049535
**ErbB Family Main Pathway**	0.105321	0.067931	0.094712	0.049535
**ErbB Family Pathway (Anti-Apoptosis)**	0	0	0	1
**ERK Signaling Main Pathway**	0.043805	0.034087	0.049716	0.049535
**Erythropoeitin Main Pathway**	-0.02343	-0.0269	-0.01205	0.049535
**Estrogen Main Pathway**	0.033409	0.031557	0.049068	0.049535
**Fas Signaling Pathway (Negative)**	-0.01964	-0.02009	-0.01612	0.049535
**Fas Signaling Pathway (Positive)**	0	0	0	1
**FLT3 Main Pathway**	0.033974	-0.03037	0.020268	0.512691
**Glucocorticoid Receptor Main Pathway**	0.035477	0.038705	0.047446	0.049535
**Glucocorticoid Receptor Pathway (Cell cycle arrest)**	0.617242	0.360404	0.519783	0.049535
**Glucocorticoid Receptor Pathway (Cell cycle progression)**	0.530535	0.460576	0.473887	0.049535
**Glucocorticoid Receptor Pathway (Gene expression)**	0.032976	0.048552	0.042949	0.049535
**Glucocorticoid Receptor Pathway (Inflammatory cytokines)**	0.020811	0.022654	0.039245	0.049535
**Glucocorticoid Receptor Pathway (SMAD signaling)**	0	0	0	1
**GPCR Main Pathway**	0.039096	0.019919	0.041535	0.049535
**GPCR Pathway (Gene expression)**	0.020869	-0.01561	0.008727	0.827259
**Growth Hormone Main Pathway**	0.020873	0.021779	0.046941	0.049535
**Growth Hormone Pathway (Cell survival)**	0	0	0	1
**Growth Hormone Pathway (Gene expression)**	0.073041	0.075099	0.110037	0.049535
**Growth Hormone Pathway (Glucose uptake)**	0.01793	0.018707	0.040321	0.049535
**Growth Hormone Pathway (Protein synthesis)**	0	0	0	1
**GSK3 Main Pathway**	0.044731	0.043171	0.056708	0.049535
**GSK3 Pathway (Degradation)**	0.044507	0.058939	0.059318	0.049535
**GSK3 Pathway (Gene expression)**	0.088776	0.115861	0.10705	0.049535
**GSK3 Pathway (Translation)**	0	0	0	1
**G-protein Pathway (Ras family GTPases)**	0.062562	0.042391	0.047969	0.049535
**Hedgehog Main Pathway**	-0.18074	-0.20246	-0.16841	0.049535
**Hedgehog Pathway (Repression of Hh, BMP)**	0.295242	0.274566	0.231255	0.049535
**Hedgehog Pathway (Activation of BMP, Ptc, WNT)**	0	0	0	1
**HGF Main Pathway**	0.053555	0.076911	0.076151	0.049535
**HGF Pathway (Anoikis)**	0.103672	0.116248	0.104765	0.049535
**HGF Pathway (Cell adhesion, cell mirgation)**	0.048411	0.10012	0.108818	0.049535
**HGF Pathway (Cell cycle progression)**	0.196932	0.245833	0.205158	0.049535
**HGF Pathway (Cell polarity, cell motility)**	0	0	0	1
**HGF Pathway (Cell scattering)**	0.08424	0.093855	0.11505	0.049535
**HGF Pathway (Cell survival)**	0.08424	0.093855	0.11505	0.049535
**HGF Pathway (IP3 pathway)**	0.096875	0.107933	0.132308	0.049535
**HGF Pathway (PKC pathway)**	0	0	0	1
**HIF1-Alpha Main Pathway**	-0.02059	-0.0655	-0.03649	0.049535
**HIF1Alpha Pathway (Gene expression)**	0.081849	0.090305	0.099713	0.049535
**HIF1Alpha Pathway (HIF1alpha degradation)**	0	0	0	1
**HIF1Alpha Pathway (NOS pathway)**	0.094441	0.104198	0.115053	0.049535
**HIF1Alpha Pathway (p53 Hypoxia pathway)**	0	0	0	1
**HIF1Alpha Pathway (Pyruvate)**	0.081849	0.090305	0.099713	0.049535
**HIF1Alpha Pathway (VEGF pathway)**	0.040378	-0.00744	0.027802	0.512691
**Hypoxia pathway EMT 1**	0.970476	0.955626	1.015518	0.049535
**Hypoxia pathway EMT 2**	0.970476	0.955626	1.015518	0.049535
**Hypoxia pathway EMT 3**	0.970476	0.955626	1.015518	0.049535

**Hypoxia pathway EMT 4**	0.970476	0.955626	1.015518	0.049535
**IGF1R Main Pathway**	0.027193	0.035007	0.038386	0.049535
**IGF1R Signaling Pathway (Cell survival)**	0	0	0	1
**IGF1R Signaling Pathway (Glucose uptake)**	0	0	0	1
**IGF1R Signaling Pathway (Glycogen synthesis)**	0	0	0	1
**IGF1R Signaling Pathway (IKB degradation)**	0	0	0	1
**IGF1R Signaling Pathway (Protein synthesis)**	0	0	0	1
**ILK Main Pathway**	0.105898	0.091368	0.104873	0.049535
**ILK Pathway (Apoptosis)**	0.194154	0.185308	0.203222	0.049535
**ILK Pathway (Cell adhesion, cell motility, opsonization)**	0.197466	0.186582	0.204751	0.049535
**ILK Pathway (Cell cycle proliferation)**	0.181779	0.174057	0.193759	0.049535
**ILK Pathway (Cell migration, retraction)**	0.194332	0.18362	0.201501	0.049535
**ILK Pathway (Cell motility)**	0.1737	0.170718	0.183708	0.049535
**ILK Pathway (Cytoskeletal reorganization)**	0.1981	0.189891	0.205534	0.049535
**ILK Pathway (G2-phase arrest)**	0.181779	0.174057	0.193759	0.049535
**ILK Pathway (Induced cell proliferation)**	0.127897	0.069619	0.100491	0.049535
**ILK Pathway (Regulation of intermediate filaments)**	0.195886	0.185089	0.203113	0.049535
**ILK Pathway (Regulation of junction assembly of desmosomes)**	0.195886	0.185089	0.203113	0.049535
**ILK Pathway (Wound healing)**	0.208613	0.194371	0.212366	0.049535
**IL-10 Main Pathway**	0.038361	-0.03859	0.003888	0.827259
**IL-10 Pathway (Gene expression)**	0.260245	0.191861	0.226769	0.049535
**IL-10 Pathway (Stability determination)**	2.579745	1.849887	2.098921	0.049535
**IL-10 Pathway (Translational modulation)**	0.240588	-0.03484	0.121292	0.512691
**IL-2 Main Pathway**	-0.00823	-0.01928	-0.00702	0.049535
**IL-2 Pathway (Actin reorganization)**	0	0	0	1
**IL-2 Pathway (Apoptosis)**	0.037484	0.055233	0.084351	0.049535
**IL-2 Pathway (Apoptosis inhibition)**	0.031566	0.046512	0.071033	0.049535
**IL-2 Pathway (Protein synthesis)**	0	0	0	1
**IL-6 Main Pathway**	0.0583	0.011092	0.032355	0.049535
**Integrin SIgnaling Main Pathway**	0.089848	0.079099	0.09784	0.049535
**Integrin SIgnaling Pathway (Cell survival)**	-0.01658	-0.01162	0.014136	0.512691
**Integrin SIgnaling Pathway (Cytoskeleton contraction integrin modulation cell invasion and migration)**	-0.04864	-0.04479	-0.03283	0.049535
**Integrin SIgnaling Pathway (Focal adhesion and stress fibers)**	0	0	0	1
**Integrin SIgnaling Pathway (Translocation to the nucleus)**	0.159292	0.113687	0.147941	0.049535
**Interactions Report**	0.873429	0.860064	0.913966	0.049535
**Interferon Main Pathway**	0.035094	0.014611	0.028029	0.049535
**Interferon Pathway (Gene expression)**	0.079209	0.060994	0.076469	0.049535
**Interferon Pathway (Transcription)**	0	0	0	1
**Interferon Pathway (Translation)**	0	0	0	1
**IP3 Main Pathway**	0.001686	0.006551	0.02337	0.12663
**IP3 Pathway (Gene expression)**	0	0	0	1
**JAK mStat Main Pathway**	-0.01408	-0.03715	-0.02412	0.049535
**JAK mStat Pathway (Akt pathway)**	0.017054	0.013816	0.022938	0.049535
**JAK mStat Pathway (JAK degradation)**	-0.03857	-0.05108	-0.05141	0.049535
**JNK Main Pathway**	0.026678	0.015854	0.034642	0.049535
**JNK Pathway (Apoptosis, Inflammation, Tumorigenesis, Cell Migration)**	0.089578	0.052092	0.080799	0.049535
**JNK Pathway (Insulin signaling)**	-0.06783	-0.00073	-0.03283	0.12663
**MAPK Family Main Pathway**	0.038164	0.018884	0.03699	0.049535
**MAPK Family Pathway (Chromatin Remodeling)**	0.054627	0.042065	0.052737	0.049535
**MAPK Family Pathway (Cytoskeleton)**	0.105612	0.081326	0.101959	0.049535

**MAPK Family Pathway (Gene Expression)**	-0.00446	-0.05637	-0.02272	0.049535
**MAPK Family Pathway (IKBs Degradation)**	0	0	0	1
**MAPK Family Pathway (Translation)**	0	0	0	1
**MAPK Signaling Main Pathway**	0.060147	0.049605	0.07038	0.049535
**MAPK Signaling Pathway (Cell Survival, Inflammation, Apoptosis, Osmoregulation)**	0.092228	0.047123	0.076568	0.049535
**MAPK Signaling Pathway (Gene Expression)**	0.072834	0.031393	0.056388	0.049535
**DDR pathway (MMR)**	0	0	0	1
**Mismatch Repair Main Pathway**	0	0	0	1
**Mitochondrial Apopotosis Main Pathway**	-0.04947	-0.06614	-0.07108	0.049535
**Mitochondrial Apopotosis Pathway (Apoptosis)**	-0.06382	-0.06529	-0.0524	0.049535
**Mitochondrial Apopotosis Pathway (Depolarization)**	0	0	0	1
**Mitochondrial Apopotosis Pathway (DNA fragmentation)**	0	0	0	1
**Mitochondrial Apopotosis Pathway (Gene expression)**	0	0	0	1
**mTOR Main Pathway**	0.024505	0.00607	0.024953	0.049535
**mTOR Pathway (Actin organization)**	-0.06453	-0.06556	-0.05876	0.049535
**mTOR Pathway (Akt signaling)**	0	0	0	1
**mTOR Pathway (Scanning)**	0	0	0	1
**mTOR Pathway (Translation on)**	0	0	0	1
**mTOR Pathway (VEGF pathway)**	0	0	0	1
**NGF (Negative) Main Pathway**	0	0	0	1
**NGF (Negative) Pathway (Apoptosis)**	0	0	0	1
**NGF (Positive) Main Pathway**	0.051301	0.02752	0.049717	0.049535
**NHEJ mechanisms of DSBs repair effect**	0	0	0	1
**Notch Main Pathway**	-0.08958	-0.1319	-0.11668	0.049535
**DDR Pathway (NER)**	0	0	0	1
**p38 (Negative) Main Signaling Pathway**	0.033812	0.007969	0.029217	0.049535
**p38 (Positive) Main Signaling Pathway**	0.033743	0.007953	0.029158	0.049535
**p53 Signaling (Negative) Main Pathway**	0.069182	0.054447	0.059183	0.049535
**p53 Signaling (Negative) Pathway (p53 Degradation)**	-0.04451	-0.05894	-0.05932	0.049535
**PAK Main Pathway**	0.059386	0.0391	0.055402	0.049535
**PAK Pathway (Actin Cytoskeleton)**	0.061143	0.051945	0.062997	0.049535
**PAK Pathway (Myosin Activation)**	0.070696	0.075913	0.070751	0.049535
**PPAR Main Pathway**	0.032177	0.024819	0.047386	0.049535
**PTEN Main Pathway**	-0.03002	-0.00325	-0.03066	0.049535
**RANK Signaling in Osteoclast Main Pathway**	0.075068	0.076781	0.087535	0.049535
**RANK Signaling in Osteoclast Pathway (IKBs Degradation)**	0	0	0	1
**RAS Main Pathway**	0.008002	0.013863	0.025914	0.049535
**Telomere Main Pathway**	-0.04043	-0.09137	-0.05778	0.049535
**RNA Polymerase II Complex Pathway**	-0.03922	-0.05305	-0.04809	0.049535
**Cell Cycle Pathway (SCC during S-phase)**	0	0	0	1
**SMAD (Negative) Main Pathway**	0.12905	0.144013	0.139306	0.049535
**SMAD (Negative) Pathway (Degradation)**	0.029927	0.039631	0.039886	0.049535
**SMAD (Positive) Main Pathway**	0.12905	0.144013	0.139306	0.049535
**SMAD (Positive) Pathway (Degradation)**	0.029927	0.039631	0.039886	0.049535
**Cell Cycle Pathway (Metaphase-Anaphase)**	0.096991	0.103638	0.090483	0.049535
**Cell Cycle Pathway (Origin of S-phase)**	-0.00316	0.037238	0.007268	0.512691
**STAT3 Main Pathway**	0.057792	0.028634	0.044222	0.049535
**TGF beta Main Pathway**	0.026385	0.009942	0.035585	0.049535
**TGF beta Pathway (Epithelial mesehchymal transition)**	0.101418	0.12055	0.153032	0.049535
**TGF beta Pathway (Post-transcriptional G1 arrest)**	0.053088	0.056269	0.062319	0.049535

**TGF beta Pathway (SnON degradation)**	0	0	0	1
**TGF beta Pathway (Tumorigenesis)**	0	0	0	1
**TGF beta Pathway (Tumor suppression)**	0	0	0	1
**TNF (Negative) Main Pathway**	-0.0403	-0.04124	-0.03309	0.049535
**TNF (Negative) Pathway (Apoptosis)**	-0.04505	-0.04609	-0.03699	0.049535
**TNF (Positive) Main Pathway**	0.03233	0.024896	0.031212	0.049535
**TNF (Positive) Pathway (Gene expression, Cell survival)**	0	0	0	1
**TNF (Positive) Pathway (IKBs degradation)**	0	0	0	1
**TRAF (Negative) Main Pathway**	0	0	0	1
**TRAF (Negative) Pathway (IKBs Degradation)**	0	0	0	1
**TRAF (Positive) Main Pathway**	0.00293	-0.02573	-0.0053	0.512691
**TRAF (Positive) Pathway (IKBs Degradation)**	0	0	0	1
**Transcription of mRNA Pathway**	-0.04576	-0.06189	-0.0561	0.049535
**Cell Cycle Pathway (End of S-phase)**	0.03065	0.045689	0.029901	0.049535
**Translation Regulation of EIF4F activity**	-0.03588	-0.06976	-0.04929	0.049535
**Ubiquitin Proteasome Main Pathway**	0	0	0	1
**Ubiquitin Proteasome Pathway (Degraded Protein)**	0	0	0	1
**VEGF Main Pathway**	0.083169	0.022447	0.061863	0.049535
**VEGF Pathway (Actin Reorganization)**	0.267935	0.209459	0.269378	0.049535
**VEGF Pathway (Nitric Oxide Production)**	0	0	0	1
**Wnt Main Pathway**	0.047603	0.035112	0.049138	0.049535
**Wnt Pathway (Ctnn-b Degradation)**	-0.04959	-0.06567	-0.0661	0.049535
**IL-6 Pathway (IKBs degradation)**	0	0	0	1

**Pathway**	**M_86_32h_1**	**M_86_32h_2**	**M_86_32h_3**	**p-value_Mean**
**AHR Main Pathway**	0	0	0	1
**AHR Pathway (AHR Degradation)**	0	0	0	1
**AHR Pathway (Cath-D Expression)**	0	0	0	1
**AHR Pathway (C-MycExpression)**	0	0	0	1
**AHR Pathway (PS2 Gene Expression)**	0	0	0	1
**AKT Main Pathway**	0.060424	0.071181	0.061002	0.049535
**AKT Pathway (Aggregation & Neurodegeneration)**	0.027091	0.030477	0.032081	0.049535
**AKT Pathway (Apoptosis Inhibition)**	0.026029	0.029282	0.030823	0.049535
**AKT Pathway (Blocks Apoptosis)**	0	0	0	1
**AKT Pathway (Cardiovascular Homeostasis)**	0.029499	0.033186	0.034932	0.049535
**AKT Pathway (Caspase Cascade)**	0	0	0	1
**AKT Pathway (Cell Cycle)**	0	0	0	1
**AKT Pathway (Cell Cycle Progression)**	-0.12165	-0.09055	-0.11137	0.049535
**AKT Pathway (Cell Survival)**	0	0	0	1
**AKT Pathway (Death Genes)**	0	0	0	1
**AKT Pathway (Elevation of Glucose Import)**	0	0	0	1
**AKT Pathway (ERK Pathway)**	0	0	0	1
**AKT Pathway (Genetic Stability)**	0	0	0	1
**AKT Pathway (Glucose Uptake)**	0.03017	0.033941	0.035726	0.049535
**AKT Pathway (Glycogen Synthesis)**	0	0	0	1
**AKT Pathway (Insulin Stimulated Mitogenesis)**	0.029499	0.033186	0.034932	0.049535
**AKT Pathway (JNK Pathway)**	0	0	0	1
**AKT Pathway (Neuroprotection)**	0.029499	0.033186	0.034932	0.049535
**AKT Pathway (NF-kB Pathway)**	0.027655	0.031112	0.032749	0.049535

**AKT Pathway (p53 Degradation)**	0	0	0	1
**AKT Pathway (p73 Mediated Apoptosis)**	0	0	0	1
**AKT Pathway (Protein Synthesis)**	0	0	0	1
**AKT Pathway (Regeneration of Cyclic Nucleotide)**	0	0	0	1
**AKT Pathway (Respiratory Burst)**	0.029499	0.033186	0.034932	0.049535
**AKT Pathway (Survival Genes)**	0	0	0	1
**AKT Pathway (Synaptic Signaling)**	0	0	0	1
**AKT Pathway (Translation)**	0	0	0	1
**Androgen Receptor Pathway**	0.08491	0.09619	0.090212	0.049535
**Androgen Receptor Pathway (Apoptosis)**	0.046287	0.104754	0.08255	0.049535
**Androgen Receptor Pathway (Degradation)**	0.045988	0.069977	0.054133	0.049535
**Androgen Receptor Pathway (Cell Survival & Cell Growth)**	0.059659	0.057729	0.048361	0.049535
**Androgen Receptor Pathway (Gonadotropin Regulation)**	-0.03578	-0.01244	-0.02965	0.049535
**Androgen Receptor Pathway (Histone Modification)**	-0.03578	-0.01244	-0.02965	0.049535
**Androgen Receptor Pathway (Prostate Differentiation & Development)**	-0.03578	-0.01244	-0.02965	0.049535
**Androgen Receptor Pathway (Sexual Differentiation & Sexual Maturation at Puberty)**	-0.03578	-0.01244	-0.02965	0.049535
**ATM Main Pathway**	0.120154	0.100467	0.092802	0.049535
**ATM Pathway (Apoptosis)**	0	0	0	1
**ATM Pathway (Apoptosis, Senescense)**	0.102388	0.195777	0.114126	0.049535
**ATM Pathway (Cell Cicle Checkpoint Control)**	0.122866	0.234933	0.136952	0.049535
**ATM Pathway (Cell Survival)**	0.122831	0.133878	0.104691	0.049535
**ATM Pathway (Checkpoint Activation)**	0.122866	0.234933	0.136952	0.049535
**ATM Pathway (DNA Repair)**	0.061433	0.117466	0.068476	0.049535
**ATM Pathway (G2_M Checkpoint Arrest)**	0.345887	0.246709	0.253758	0.049535
**ATM Pathway (G2 Mitosis Progression)**	0	0	0	1
**ATM Pathway (MDMX Ubiquitination, Degradation)**	0	0	0	1
**ATM Pathway (NF-kB Pathway)**	0.122866	0.234933	0.136952	0.049535
**ATM Pathway (Synaptic Vesicle Transport)**	0.122866	0.234933	0.136952	0.049535
**ATM Pathway (S-Phase Arrest)**	0.122866	0.234933	0.136952	0.049535
**ATM Pathway (S-Phase Progression)**	0	0	0	1
**DDR Pathway (BRCA1-induced responses)**	0.152817	0.091193	0.148008	0.049535
**BRCA1 Main Pathway**	-0.04919	-0.02	-0.02652	0.049535
**cAMP Main Pathway**	0.082057	0.085698	0.084975	0.049535
**cAMP Pathway (Axonal Growth)**	-0.10942	-0.04915	-0.0668	0.049535
**cAMP Pathway (Cardiovascular Homeostasis)**	-0.23448	-0.10531	-0.14315	0.049535
**cAMP Pathway (Cell Growth)**	0	0	0	1
**cAMP Pathway (Cell Proliferation)**	-0.25251	-0.11341	-0.15416	0.049535
**cAMP Pathway (Cell Survival)**	-0.0248	0.031946	0.010576	0.512691
**cAMP Pathway (Cell Survival, Chemotaxis)**	0	0	0	1
**cAMP Pathway (Cytokine Production)**	0.175447	0.119072	0.126515	0.049535
**cAMP Pathway (Degradation of Cell Cycle Regulators)**	-0.10589	-0.04756	-0.06465	0.049535
**cAMP Pathway (Endothelial Cell Regulation)**	0.157386	0.190101	0.231713	0.049535
**cAMP Pathway (Glycogen Synthesis)**	-0.29842	-0.13403	-0.18219	0.049535
**cAMP Pathway (Glycolysis)**	-0.08291	0.03422	-0.05398	0.512691
**cAMP Pathway (Metabolic Energy)**	0	0	0	1
**cAMP Pathway (Myocardial Contraction)**	0.009528	0.016436	0.007179	0.12663
**cAMP Pathway (Oncogenesis)**	-0.23448	-0.10531	-0.14315	0.049535
**cAMP Pathway (Protein Retention)**	-0.00042	0.175815	0.090615	0.12663
**cAMP Pathway (Regulation of Cytoskeleton)**	-0.20517	-0.09215	-0.12526	0.049535
**Caspase Cascade Main**	-0.08774	-0.09824	-0.09366	0.049535

**Caspase Cascade (Activated Tissue Transglutaminase)**	0.009822	0.010252	0.003712	0.12663
**Caspase Cascade (Apoptosis)**	0	0	0	1
**Caspase Cascade (Cell Survival)**	-0.04339	-0.09821	-0.07739	0.049535
**Caspase Cascade (ICAD Degradation)**	0	0	0	1
**CD40 Main Pathway**	0.049554	0.028879	0.03687	0.049535
**CD40 Pathway (Cell Survival)**	0	0	0	1
**CD40 Pathway (Gene Expression)**	0.11652	0.058092	0.089044	0.049535
**CD40 Pathway (IKBs Degradation)**	0.08225	0.041006	0.062854	0.049535
**Cellular Anti Apoptosis Main Pathway**	0.092676	0.103181	0.098285	0.049535
**Cellular Anti Apoptosis Pathway (Apoptosis)**	0.041012	0.027319	0.016389	0.049535
**Cellular Anti Apoptosis Pathway (Depolarization)**	0	0	0	1
**Chemokine Main Pathway**	0.057938	0.043987	0.051538	0.049535
**Chemokine Pathway (Cell Activation)**	0.042349	0.028741	0.030538	0.049535
**Chemokine Pathway (Gene Expression, Apoptosis)**	0.053397	0.036239	0.038505	0.049535
**Chemokine Pathway (Internalization, Degradation, Recycling)**	0	0	0	1
**Chromatin Main Pathway**	0	0	0	1
**Chromatin Pathway (Octamer Sliding)**	0	0	0	1
**Chromatin Pathway (Octamer Transfer)**	0	0	0	1
**Circadian Main Pathway**	0	0	0	1
**CREB Main Pathway**	0.092702	0.103743	0.097046	0.049535
**CREB Pathway (Gene Expression Pathway)**	0.042832	0.041446	0.03472	0.049535
**Cytokine Main Pathway**	0	0	0	1
**DDR pathway Apoptosis**	0	0	0	1
**DDR Main pathway**	0	0	0	1
**DNA Repair Mechanisms Pathway**	0.001557	0.008155	0.009464	0.049535
**EGFR Main Pathway**	0.054547	0.040641	0.052893	0.049535
**ErbB Family Main Pathway**	0.03893	0.044505	0.044717	0.049535
**ErbB Family Pathway (Anti-Apoptosis)**	0	0	0	1
**ERK Signaling Main Pathway**	0.121017	0.128844	0.122809	0.049535
**Erythropoeitin Main Pathway**	-0.00737	-0.00124	-0.00686	0.275234
**Estrogen Main Pathway**	0.094038	0.109419	0.094906	0.049535
**Fas Signaling Pathway (Negative)**	0.050066	0.056246	0.038913	0.049535
**Fas Signaling Pathway (Positive)**	0	0	0	1
**FLT3 Main Pathway**	0.056749	0.063925	0.064677	0.049535
**Glucocorticoid Receptor Main Pathway**	0.071938	0.072846	0.067562	0.049535
**Glucocorticoid Receptor Pathway (Cell cycle arrest)**	1.081263	0.927807	0.98317	0.049535
**Glucocorticoid Receptor Pathway (Cell cycle progression)**	0	0	0	1
**Glucocorticoid Receptor Pathway (Gene expression)**	0.032084	0.031087	0.027553	0.049535
**Glucocorticoid Receptor Pathway (Inflammatory cytokines)**	0.072744	0.070875	0.071041	0.049535
**Glucocorticoid Receptor Pathway (SMAD signaling)**	0.098788	0.127857	0.07354	0.049535
**GPCR Main Pathway**	0.095239	0.10642	0.095506	0.049535
**GPCR Pathway (Gene expression)**	0.063189	0.046279	0.04865	0.049535
**Growth Hormone Main Pathway**	-0.00976	-0.00839	-0.01434	0.049535
**Growth Hormone Pathway (Cell survival)**	0	0	0	1
**Growth Hormone Pathway (Gene expression)**	-0.01521	-0.01307	-0.02234	0.049535
**Growth Hormone Pathway (Glucose uptake)**	-0.00838	-0.00721	-0.01232	0.049535
**Growth Hormone Pathway (Protein synthesis)**	0	0	0	1
**GSK3 Main Pathway**	0.083098	0.111157	0.088229	0.049535
**GSK3 Pathway (Degradation)**	0	0	0	1
**GSK3 Pathway (Gene expression)**	0.044417	-0.01452	0.020726	0.512691
**GSK3 Pathway (Translation)**	0	0	0	1

**G-protein Pathway (Ras family GTPases)**	0	0	0	1
**Hedgehog Main Pathway**	-0.19376	-0.07686	-0.15323	0.049535
**Hedgehog Pathway (Repression of Hh, BMP)**	0.387358	0.478421	0.540323	0.049535
**Hedgehog Pathway (Activation of BMP, Ptc, WNT)**	0	0	0	1
**HGF Main Pathway**	0.082662	0.076748	0.056066	0.049535
**HGF Pathway (Anoikis)**	0.19946	0.185189	0.127918	0.049535
**HGF Pathway (Cell adhesion, cell mirgation)**	0.037744	0.035044	0.032507	0.049535
**HGF Pathway (Cell cycle progression)**	0.33418	0.279023	0.209415	0.049535
**HGF Pathway (Cell polarity, cell motility)**	0	0	0	1
**HGF Pathway (Cell scattering)**	0.1294	0.132368	0.084904	0.049535
**HGF Pathway (Cell survival)**	0.1294	0.132368	0.084904	0.049535
**HGF Pathway (IP3 pathway)**	0.14881	0.152223	0.09764	0.049535
**HGF Pathway (PKC pathway)**	0	0	0	1
**HIF1-Alpha Main Pathway**	0	0	0	1
**HIF1Alpha Pathway (Gene expression)**	0	0	0	1
**HIF1Alpha Pathway (HIF1alpha degradation)**	0	0	0	1
**HIF1Alpha Pathway (NOS pathway)**	0	0	0	1
**HIF1Alpha Pathway (p53 Hypoxia pathway)**	0	0	0	1
**HIF1Alpha Pathway (Pyruvate)**	0	0	0	1
**HIF1Alpha Pathway (VEGF pathway)**	0	0	0	1
**Hypoxia pathway EMT 1**	1.509285	1.336039	1.719183	0.049535
**Hypoxia pathway EMT 2**	1.509285	1.336039	1.719183	0.049535
**Hypoxia pathway EMT 3**	1.509285	1.336039	1.719183	0.049535
**Hypoxia pathway EMT 4**	1.509285	1.336039	1.719183	0.049535
**IGF1R Main Pathway**	0.009621	0.009586	0.011457	0.049535
**IGF1R Signaling Pathway (Cell survival)**	-0.03673	-0.0264	-0.03259	0.049535
**IGF1R Signaling Pathway (Glucose uptake)**	0	0	0	1
**IGF1R Signaling Pathway (Glycogen synthesis)**	0	0	0	1
**IGF1R Signaling Pathway (IKB degradation)**	0.093216	0.046473	0.071235	0.049535
**IGF1R Signaling Pathway (Protein synthesis)**	0.112141	0	0.126904	0.19043
**ILK Main Pathway**	0.154363	0.18115	0.178657	0.049535
**ILK Pathway (Apoptosis)**	0.248492	0.266625	0.280078	0.049535
**ILK Pathway (Cell adhesion, cell motility, opsonization)**	0.272465	0.294643	0.305122	0.049535
**ILK Pathway (Cell cycle proliferation)**	0.244824	0.264752	0.274167	0.049535
**ILK Pathway (Cell migration, retraction)**	0.268141	0.289966	0.300278	0.049535
**ILK Pathway (Cell motility)**	0.229961	0.247452	0.269804	0.049535
**ILK Pathway (Cytoskeletal reorganization)**	0.283726	0.298371	0.316153	0.049535
**ILK Pathway (G2-phase arrest)**	0.244824	0.264752	0.274167	0.049535
**ILK Pathway (Induced cell proliferation)**	-0.01925	-0.02662	-0.01606	0.049535
**ILK Pathway (Regulation of intermediate filaments)**	0.270286	0.292286	0.302681	0.049535
**ILK Pathway (Regulation of junction assembly of desmosomes)**	0.270286	0.292286	0.302681	0.049535
**ILK Pathway (Wound healing)**	0.282744	0.308054	0.319175	0.049535
**IL-10 Main Pathway**	0.05902	0.034396	0.043913	0.049535
**IL-10 Pathway (Gene expression)**	0.164148	0.095663	0.122133	0.049535
**IL-10 Pathway (Stability determination)**	0	0	0	1
**IL-10 Pathway (Translational modulation)**	0.122813	0.08335	0.08856	0.049535
**IL-2 Main Pathway**	0.001408	0.018905	0.015148	0.12663
**IL-2 Pathway (Actin reorganization)**	0.055123	0.226592	0.239233	0.049535
**IL-2 Pathway (Apoptosis)**	0	0	0	1
**IL-2 Pathway (Apoptosis inhibition)**	0	0	0	1
**IL-2 Pathway (Protein synthesis)**	0	0	0	1

**IL-6 Main Pathway**	0.116788	0.096952	0.110098	0.049535
**Integrin SIgnaling Main Pathway**	0.168492	0.177336	0.182812	0.049535
**Integrin SIgnaling Pathway (Cell survival)**	0.01421	0.013193	0.012238	0.049535
**Integrin SIgnaling Pathway (Cytoskeleton contraction integrin modulation cell invasion and migration)**	0	0	0	1
**Integrin SIgnaling Pathway (Focal adhesion and stress fibers)**	0	0	0	1
**Integrin SIgnaling Pathway (Translocation to the nucleus)**	0.275278	0.298325	0.245147	0.049535
**Interactions Report**	1.358357	1.202435	1.547265	0.049535
**Interferon Main Pathway**	0.025538	0.00906	0.02342	0.049535
**Interferon Pathway (Gene expression)**	0.061407	0.041675	0.04428	0.049535
**Interferon Pathway (Transcription)**	0	0	0	1
**Interferon Pathway (Translation)**	0.186901	0	0.211507	0.19043
**IP3 Main Pathway**	0.026184	0.031556	0.026505	0.049535
**IP3 Pathway (Gene expression)**	0	0	0	1
**JAK mStat Main Pathway**	0.041421	0.03046	0.039761	0.049535
**JAK mStat Pathway (Akt pathway)**	0.024713	0.057966	0.014309	0.049535
**JAK mStat Pathway (JAK degradation)**	0	0	0	1
**JNK Main Pathway**	0.084376	0.102984	0.087283	0.049535
**JNK Pathway (Apoptosis, Inflammation, Tumorigenesis, Cell Migration)**	0.066154	0.071333	0.073173	0.049535
**JNK Pathway (Insulin signaling)**	0	0	0	1
**MAPK Family Main Pathway**	0.044061	0.04307	0.042304	0.049535
**MAPK Family Pathway (Chromatin Remodeling)**	0.042349	0.028741	0.030538	0.049535
**MAPK Family Pathway (Cytoskeleton)**	0.081875	0.055567	0.05904	0.049535
**MAPK Family Pathway (Gene Expression)**	0.067343	0.039246	0.050106	0.049535
**MAPK Family Pathway (IKBs Degradation)**	0.107557	0.053623	0.082194	0.049535
**MAPK Family Pathway (Translation)**	0	0	0	1
**MAPK Signaling Main Pathway**	0.113417	0.135182	0.123783	0.049535
**MAPK Signaling Pathway (Cell Survival, Inflammation, Apoptosis, Osmoregulation)**	-0.02015	-0.02787	-0.01681	0.049535
**MAPK Signaling Pathway (Gene Expression)**	0.023928	0.031585	0.035839	0.049535
**DDR pathway (MMR)**	0	0	0	1
**Mismatch Repair Main Pathway**	0	0	0	1
**Mitochondrial Apopotosis Main Pathway**	-0.09588	-0.12088	-0.12038	0.049535
**Mitochondrial Apopotosis Pathway (Apoptosis)**	0.109365	0.07285	0.043703	0.049535
**Mitochondrial Apopotosis Pathway (Depolarization)**	0	0	0	1
**Mitochondrial Apopotosis Pathway (DNA fragmentation)**	-0.11572	-0.26189	-0.20638	0.049535
**Mitochondrial Apopotosis Pathway (Gene expression)**	0	0	0	1
**mTOR Main Pathway**	0.095974	0.104567	0.106931	0.049535
**mTOR Pathway (Actin organization)**	0.047897	0.049332	0.04629	0.049535
**mTOR Pathway (Akt signaling)**	0	0	0	1
**mTOR Pathway (Scanning)**	0.0623	0	0.070502	0.19043
**mTOR Pathway (Translation on)**	0.03115	0	0.035251	0.19043
**mTOR Pathway (VEGF pathway)**	0	0	0	1
**NGF (Negative) Main Pathway**	0	0	0	1
**NGF (Negative) Pathway (Apoptosis)**	0	0	0	1
**NGF (Positive) Main Pathway**	0.046316	0.036655	0.046427	0.049535
**NHEJ mechanisms of DSBs repair effect**	0.036137	0.069098	0.04028	0.049535
**Notch Main Pathway**	-0.03695	-0.04311	-0.03275	0.049535
**DDR Pathway (NER)**	0	0	0	1
**p38 (Negative) Main Signaling Pathway**	0.076694	0.084565	0.076289	0.049535
**p38 (Positive) Main Signaling Pathway**	0.076538	0.084393	0.076134	0.049535
**p53 Signaling (Negative) Main Pathway**	0.148255	0.076654	0.106024	0.049535

**p53 Signaling (Negative) Pathway (p53 Degradation)**	0.02038	0.022439	0.020923	0.049535
**PAK Main Pathway**	0.122325	0.12987	0.13986	0.049535
**PAK Pathway (Actin Cytoskeleton)**	0.108031	0.108341	0.116224	0.049535
**PAK Pathway (Myosin Activation)**	0.083898	0.098266	0.123438	0.049535
**PPAR Main Pathway**	0.092556	0.100331	0.090991	0.049535
**PTEN Main Pathway**	-0.02979	-0.02988	-0.03499	0.049535
**RANK Signaling in Osteoclast Main Pathway**	0.124038	0.083654	0.122852	0.049535
**RANK Signaling in Osteoclast Pathway (IKBs Degradation)**	0.069912	0.034855	0.053426	0.049535
**RAS Main Pathway**	0.018408	0.037666	0.016147	0.049535
**Telomere Main Pathway**	-0.02118	-0.04051	-0.02361	0.049535
**RNA Polymerase II Complex Pathway**	0.026897	0.022147	0.022554	0.049535
**Cell Cycle Pathway (SCC during S-phase)**	0	0	0	1
**SMAD (Negative) Main Pathway**	0.117697	0.120776	0.137763	0.049535
**SMAD (Negative) Pathway (Degradation)**	0	0	0	1
**SMAD (Positive) Main Pathway**	0.117697	0.120776	0.137763	0.049535
**SMAD (Positive) Pathway (Degradation)**	0	0	0	1
**Cell Cycle Pathway (Metaphase-Anaphase)**	0.102339	0.129445	0.132727	0.049535
**Cell Cycle Pathway (Origin of S-phase)**	0.021278	0.079555	0.04297	0.049535
**STAT3 Main Pathway**	0.107979	0.088025	0.092858	0.049535
**TGF beta Main Pathway**	0.086893	0.100158	0.095158	0.049535
**TGF beta Pathway (Epithelial mesehchymal transition)**	0.054593	0.070658	0.04064	0.049535
**TGF beta Pathway (Post-transcriptional G1 arrest)**	0.041491	0.0537	0.030887	0.049535
**TGF beta Pathway (SnON degradation)**	0	0	0	1
**TGF beta Pathway (Tumorigenesis)**	0	0	0	1
**TGF beta Pathway (Tumor suppression)**	0	0	0	1
**TNF (Negative) Main Pathway**	0.069072	0.046011	0.027602	0.049535
**TNF (Negative) Pathway (Apoptosis)**	0.077198	0.051424	0.030849	0.049535
**TNF (Positive) Main Pathway**	0.035356	0.049911	0.038263	0.049535
**TNF (Positive) Pathway (Gene expression, Cell survival)**	0.063418	0.076974	0.068593	0.049535
**TNF (Positive) Pathway (IKBs degradation)**	0.07768	0.038728	0.059362	0.049535
**TRAF (Negative) Main Pathway**	0	0	0	1
**TRAF (Negative) Pathway (IKBs Degradation)**	0.07768	0.038728	0.059362	0.049535
**TRAF (Positive) Main Pathway**	0.041321	0.017141	0.030511	0.049535
**TRAF (Positive) Pathway (IKBs Degradation)**	0.07768	0.038728	0.059362	0.049535
**Transcription of mRNA Pathway**	0.03138	0.025838	0.026313	0.049535
**Cell Cycle Pathway (End of S-phase)**	0	0	0	1
**Translation Regulation of EIF4F activity**	0.021169	0.027398	0.015758	0.049535
**Ubiquitin Proteasome Main Pathway**	0.001187	0.007084	0.005111	0.049535
**Ubiquitin Proteasome Pathway (Degraded Protein)**	-0.01328	-0.01936	-0.01728	0.049535
**VEGF Main Pathway**	0.15227	0.116694	0.16149	0.049535
**VEGF Pathway (Actin Reorganization)**	0.299781	0.229741	0.317933	0.049535
**VEGF Pathway (Nitric Oxide Production)**	0	0	0	1
**Wnt Main Pathway**	0.04592	0.052567	0.057212	0.049535
**Wnt Pathway (Ctnn-b Degradation)**	0	0	0	1
**IL-6 Pathway (IKBs degradation)**	0.11652	0.058092	0.089044	0.049535

**Pathway**	**M_86_48h_1**	**M_86_48h_2**	**M_86_48h_3**	**p-value_Mean**
**AHR Main Pathway**	0.036218	0.035253	0.030011	0.049535
**AHR Pathway (AHR Degradation)**	0	0	0	1

**AHR Pathway (Cath-D Expression)**	0	0	0	1
**AHR Pathway (C-MycExpression)**	0	0	0	1
**AHR Pathway (PS2 Gene Expression)**	0	0	0	1
**AKT Main Pathway**	0.04742	0.062944	0.057645	0.049535
**AKT Pathway (Aggregation & Neurodegeneration)**	0.008996	0.026391	0.026068	0.049535
**AKT Pathway (Apoptosis Inhibition)**	0.006714	0.021303	0.021508	0.049535
**AKT Pathway (Blocks Apoptosis)**	0	0	0	1
**AKT Pathway (Cardiovascular Homeostasis)**	0.029605	0.049007	0.045835	0.049535
**AKT Pathway (Caspase Cascade)**	0.018503	0.037952	0.03176	0.049535
**AKT Pathway (Cell Cycle)**	0.508975	0.48619	0.527627	0.049535
**AKT Pathway (Cell Cycle Progression)**	-0.0206	-0.01351	-0.01272	0.049535
**AKT Pathway (Cell Survival)**	0	0	0	1
**AKT Pathway (Death Genes)**	0.017475	0.035843	0.029996	0.049535
**AKT Pathway (Elevation of Glucose Import)**	0.016555	0.033957	0.028417	0.049535
**AKT Pathway (ERK Pathway)**	0	0	0	1
**AKT Pathway (Genetic Stability)**	-0.05517	-0.03546	-0.04262	0.049535
**AKT Pathway (Glucose Uptake)**	0.030278	0.050121	0.046877	0.049535
**AKT Pathway (Glycogen Synthesis)**	0	0	0	1
**AKT Pathway (Insulin Stimulated Mitogenesis)**	0.029605	0.049007	0.045835	0.049535
**AKT Pathway (JNK Pathway)**	0.017475	0.035843	0.029996	0.049535
**AKT Pathway (Neuroprotection)**	0.029605	0.049007	0.045835	0.049535
**AKT Pathway (NF-kB Pathway)**	0.027755	0.045944	0.042971	0.049535
**AKT Pathway (p53 Degradation)**	0	0	0	1
**AKT Pathway (p73 Mediated Apoptosis)**	0.017974	0.036868	0.030853	0.049535
**AKT Pathway (Protein Synthesis)**	0	0	0	1
**AKT Pathway (Regeneration of Cyclic Nucleotide)**	0.016131	0.033086	0.027688	0.049535
**AKT Pathway (Respiratory Burst)**	0.029605	0.049007	0.045835	0.049535
**AKT Pathway (Survival Genes)**	0	0	0	1
**AKT Pathway (Synaptic Signaling)**	0.013385	0.027455	0.022976	0.049535
**AKT Pathway (Translation)**	0	0	0	1
**Androgen Receptor Pathway**	0.01098	0.022616	0.022983	0.049535
**Androgen Receptor Pathway (Apoptosis)**	0.126175	0.17175	0.187624	0.049535
**Androgen Receptor Pathway (Degradation)**	-0.027	-0.01769	-0.02586	0.049535
**Androgen Receptor Pathway (Cell Survival & Cell Growth)**	0.042917	0.064109	0.055582	0.049535
**Androgen Receptor Pathway (Gonadotropin Regulation)**	0.031545	0.030704	0.026139	0.049535
**Androgen Receptor Pathway (Histone Modification)**	0.031545	0.030704	0.026139	0.049535
**Androgen Receptor Pathway (Prostate Differentiation & Development)**	0.031545	0.030704	0.026139	0.049535
**Androgen Receptor Pathway (Sexual Differentiation & Sexual Maturation at Puberty)**	0.031545	0.030704	0.026139	0.049535
**ATM Main Pathway**	0.024857	0.025512	0.031773	0.512691
**ATM Pathway (Apoptosis)**	0	0	0	1
**ATM Pathway (Apoptosis, Senescense)**	-0.08273	-0.11114	-0.07168	0.049535
**ATM Pathway (Cell Cicle Checkpoint Control)**	0.155888	0.160741	0.168673	0.049535
**ATM Pathway (Cell Survival)**	0.051963	0.05358	0.056224	0.049535
**ATM Pathway (Checkpoint Activation)**	0.155888	0.160741	0.168673	0.049535
**ATM Pathway (DNA Repair)**	0.726209	0.755694	0.702463	0.049535
**ATM Pathway (G2_M Checkpoint Arrest)**	-1.04843	-1.14942	-1.00181	0.049535
**ATM Pathway (G2 Mitosis Progression)**	-1.92915	-2.11304	-1.94095	0.049535
**ATM Pathway (MDMX Ubiquitination, Degradation)**	-0.25517	-0.29411	-0.25469	0.049535
**ATM Pathway (NF-kB Pathway)**	0.155888	0.160741	0.168673	0.049535
**ATM Pathway (Synaptic Vesicle Transport)**	0.155888	0.160741	0.168673	0.049535

**ATM Pathway (S-Phase Arrest)**	0.7608	0.811532	0.726234	0.049535
**ATM Pathway (S-Phase Progression)**	-0.0484	-0.02253	-0.04959	0.12663
**DDR Pathway (BRCA1-induced responses)**	-0.14176	-0.1591	-0.17686	0.049535
**BRCA1 Main Pathway**	0.247051	0.240449	0.206269	0.049535
**cAMP Main Pathway**	0.017076	0.022329	0.022773	0.049535
**cAMP Pathway (Axonal Growth)**	-0.14496	-0.16525	-0.15261	0.049535
**cAMP Pathway (Cardiovascular Homeostasis)**	-0.248	-0.28444	-0.28439	0.049535
**cAMP Pathway (Cell Growth)**	-0.3154	-0.41144	-0.36931	0.049535
**cAMP Pathway (Cell Proliferation)**	-0.12151	-0.11642	-0.13581	0.049535
**cAMP Pathway (Cell Survival)**	0.007142	0.012933	0.00296	0.049535
**cAMP Pathway (Cell Survival, Chemotaxis)**	-0.23655	-0.30858	-0.27698	0.049535
**cAMP Pathway (Cytokine Production)**	-0.13517	-0.17633	-0.15827	0.049535
**cAMP Pathway (Degradation of Cell Cycle Regulators)**	-0.07872	-0.10373	-0.10638	0.049535
**cAMP Pathway (Endothelial Cell Regulation)**	0.060787	0.038899	0.032366	0.049535
**cAMP Pathway (Glycogen Synthesis)**	-0.14361	-0.13759	-0.16051	0.049535
**cAMP Pathway (Glycolysis)**	0.055736	0.024437	0.003095	0.049535
**cAMP Pathway (Metabolic Energy)**	0.04194	0.086024	0.07199	0.049535
**cAMP Pathway (Myocardial Contraction)**	-0.01377	-0.01374	-0.00819	0.12663
**cAMP Pathway (Oncogenesis)**	-0.11283	-0.10811	-0.12611	0.049535
**cAMP Pathway (Protein Retention)**	0.190722	0.200014	0.160206	0.049535
**cAMP Pathway (Regulation of Cytoskeleton)**	-0.09873	-0.09459	-0.11035	0.049535
**Caspase Cascade Main**	-0.04331	-0.05211	-0.04805	0.049535
**Caspase Cascade (Activated Tissue Transglutaminase)**	-0.00601	0.008712	0.02018	0.512691
**Caspase Cascade (Apoptosis)**	-0.08705	-0.08255	-0.08114	0.049535
**Caspase Cascade (Cell Survival)**	-0.14083	-0.1503	-0.16876	0.049535
**Caspase Cascade (ICAD Degradation)**	0	0	0	1
**CD40 Main Pathway**	0.01187	0.024346	0.020375	0.049535
**CD40 Pathway (Cell Survival)**	0	0	0	1
**CD40 Pathway (Gene Expression)**	0	0	0	1
**CD40 Pathway (IKBs Degradation)**	0	0	0	1
**Cellular Anti Apoptosis Main Pathway**	0.030287	0.039853	0.043121	0.049535
**Cellular Anti Apoptosis Pathway (Apoptosis)**	0.033009	0.037386	0.027432	0.049535
**Cellular Anti Apoptosis Pathway (Depolarization)**	0	0	0	1
**Chemokine Main Pathway**	0.018858	0.023375	0.025285	0.049535
**Chemokine Pathway (Cell Activation)**	-0.05117	-0.06219	-0.04622	0.049535
**Chemokine Pathway (Gene Expression, Apoptosis)**	-0.06452	-0.07841	-0.05828	0.049535
**Chemokine Pathway (Internalization, Degradation, Recycling)**	-0.07222	-0.08454	-0.09249	0.049535
**Chromatin Main Pathway**	0.182511	0.188254	0.185987	0.049535
**Chromatin Pathway (Octamer Sliding)**	0.210589	0.217217	0.214601	0.049535
**Chromatin Pathway (Octamer Transfer)**	0.182511	0.188254	0.185987	0.049535
**Circadian Main Pathway**	0.090621	0.175452	0.16099	0.049535
**CREB Main Pathway**	0.028818	0.037635	0.039814	0.049535
**CREB Pathway (Gene Expression Pathway)**	0.00402	0.007302	-0.00426	0.512691
**Cytokine Main Pathway**	0	0	0	1
**DDR pathway Apoptosis**	0.995677	1.004169	0.898957	0.049535
**DDR Main pathway**	0.797762	0.818253	0.761004	0.049535
**DNA Repair Mechanisms Pathway**	0.179596	0.173798	0.144169	0.049535
**EGFR Main Pathway**	0.025787	0.017003	0.021288	0.049535
**ErbB Family Main Pathway**	0.052843	0.051843	0.056228	0.049535
**ErbB Family Pathway (Anti-Apoptosis)**	0	0	0	1
**ERK Signaling Main Pathway**	0.075713	0.098048	0.096999	0.049535

**Erythropoeitin Main Pathway**	-2.70E-05	-0.00275	0.001202	0.512691
**Estrogen Main Pathway**	0.050552	0.061875	0.060783	0.049535
**Fas Signaling Pathway (Negative)**	0.095166	0.095234	0.085963	0.049535
**Fas Signaling Pathway (Positive)**	0	0	0	1
**FLT3 Main Pathway**	0.070035	0.090717	0.093141	0.049535
**Glucocorticoid Receptor Main Pathway**	0.030834	0.034079	0.033044	0.049535
**Glucocorticoid Receptor Pathway (Cell cycle arrest)**	0.319655	0.185837	0.200564	0.049535
**Glucocorticoid Receptor Pathway (Cell cycle progression)**	0	0	0	1
**Glucocorticoid Receptor Pathway (Gene expression)**	0.044298	0.049765	0.048667	0.049535
**Glucocorticoid Receptor Pathway (Inflammatory cytokines)**	0.019717	0.024821	0.023343	0.049535
**Glucocorticoid Receptor Pathway (SMAD signaling)**	0	0	0	1
**GPCR Main Pathway**	0.057158	0.073381	0.067269	0.049535
**GPCR Pathway (Gene expression)**	-0.00019	0.003041	0.00573	0.512691
**Growth Hormone Main Pathway**	-0.04366	-0.05015	-0.04462	0.049535
**Growth Hormone Pathway (Cell survival)**	0	0	0	1
**Growth Hormone Pathway (Gene expression)**	-0.05239	-0.04553	-0.03943	0.049535
**Growth Hormone Pathway (Glucose uptake)**	-0.0375	-0.04307	-0.03833	0.049535
**Growth Hormone Pathway (Protein synthesis)**	0	0	0	1
**GSK3 Main Pathway**	0.076661	0.089384	0.084036	0.049535
**GSK3 Pathway (Degradation)**	0	0	0	1
**GSK3 Pathway (Gene expression)**	0.106198	0.116503	0.115345	0.049535
**GSK3 Pathway (Translation)**	0	0	0	1
**G-protein Pathway (Ras family GTPases)**	0.067535	0.059194	0.077925	0.049535
**Hedgehog Main Pathway**	0.298325	0.309043	0.316638	0.049535
**Hedgehog Pathway (Repression of Hh, BMP)**	0.357592	0.450399	0.393275	0.049535
**Hedgehog Pathway (Activation of BMP, Ptc, WNT)**	0	0	0	1
**HGF Main Pathway**	0.082817	0.106006	0.100715	0.049535
**HGF Pathway (Anoikis)**	0.141425	0.190953	0.187154	0.049535
**HGF Pathway (Cell adhesion, cell mirgation)**	0.112232	0.149509	0.132109	0.049535
**HGF Pathway (Cell cycle progression)**	0.227464	0.269254	0.290264	0.049535
**HGF Pathway (Cell polarity, cell motility)**	0	0	0	1
**HGF Pathway (Cell scattering)**	0.09546	0.14371	0.130532	0.049535
**HGF Pathway (Cell survival)**	0.09546	0.14371	0.130532	0.049535
**HGF Pathway (IP3 pathway)**	0.109779	0.165266	0.150112	0.049535
**HGF Pathway (PKC pathway)**	0	0	0	1
**HIF1-Alpha Main Pathway**	0.020138	0.038989	0.035776	0.049535
**HIF1Alpha Pathway (Gene expression)**	0.072497	0.140361	0.128792	0.049535
**HIF1Alpha Pathway (HIF1alpha degradation)**	0	0	0	1
**HIF1Alpha Pathway (NOS pathway)**	0.083651	0.161956	0.148606	0.049535
**HIF1Alpha Pathway (p53 Hypoxia pathway)**	0	0	0	1
**HIF1Alpha Pathway (Pyruvate)**	0.072497	0.140361	0.128792	0.049535
**HIF1Alpha Pathway (VEGF pathway)**	0.067966	0.131589	0.120743	0.049535
**Hypoxia pathway EMT 1**	0.642914	0.869094	0.956599	0.049535
**Hypoxia pathway EMT 2**	0.642914	0.869094	0.956599	0.049535
**Hypoxia pathway EMT 3**	0.642914	0.869094	0.956599	0.049535
**Hypoxia pathway EMT 4**	0.642914	0.869094	0.956599	0.049535
**IGF1R Main Pathway**	0.009992	0.007812	0.009489	0.12663
**IGF1R Signaling Pathway (Cell survival)**	0.02538	0.028689	0.024761	0.049535
**IGF1R Signaling Pathway (Glucose uptake)**	0	0	0	1
**IGF1R Signaling Pathway (Glycogen synthesis)**	0	0	0	1
**IGF1R Signaling Pathway (IKB degradation)**	0	0	0	1

**IGF1R Signaling Pathway (Protein synthesis)**	0	0	0	1
**ILK Main Pathway**	0.134683	0.156314	0.154108	0.049535
**ILK Pathway (Apoptosis)**	0.21401	0.269464	0.267004	0.049535
**ILK Pathway (Cell adhesion, cell motility, opsonization)**	0.233082	0.290202	0.287427	0.049535
**ILK Pathway (Cell cycle proliferation)**	0.213995	0.270112	0.266093	0.049535
**ILK Pathway (Cell migration, retraction)**	0.229383	0.285595	0.282865	0.049535
**ILK Pathway (Cell motility)**	0.193289	0.235497	0.233983	0.049535
**ILK Pathway (Cytoskeletal reorganization)**	0.225332	0.275381	0.274023	0.049535
**ILK Pathway (G2-phase arrest)**	0.213995	0.270112	0.266093	0.049535
**ILK Pathway (Induced cell proliferation)**	0.163497	0.159168	0.153488	0.049535
**ILK Pathway (Regulation of intermediate filaments)**	0.239546	0.295089	0.292239	0.049535
**ILK Pathway (Regulation of junction assembly of desmosomes)**	0.231218	0.28788	0.285128	0.049535
**ILK Pathway (Wound healing)**	0.244569	0.301665	0.298022	0.049535
**IL-10 Main Pathway**	0.119695	0.131549	0.127666	0.049535
**IL-10 Pathway (Gene expression)**	0	0	0	1
**IL-10 Pathway (Stability determination)**	0	0	0	1
**IL-10 Pathway (Translational modulation)**	0	0	0	1
**IL-2 Main Pathway**	-0.00231	-0.00651	-0.01824	0.049535
**IL-2 Pathway (Actin reorganization)**	0.104181	0.051574	0.063748	0.049535
**IL-2 Pathway (Apoptosis)**	0.115928	0.186336	0.167941	0.049535
**IL-2 Pathway (Apoptosis inhibition)**	0.136592	0.211819	0.175541	0.049535
**IL-2 Pathway (Protein synthesis)**	0	0	0	1
**IL-6 Main Pathway**	0.037428	0.054936	0.051055	0.049535
**Integrin SIgnaling Main Pathway**	0.13696	0.173864	0.170168	0.049535
**Integrin SIgnaling Pathway (Cell survival)**	0.026275	0.044069	0.033062	0.049535
**Integrin SIgnaling Pathway (Cytoskeleton contraction integrin modulation cell invasion and migration)**	-0.02052	-0.0241	-0.02499	0.049535
**Integrin SIgnaling Pathway (Focal adhesion and stress fibers)**	0.051689	0.072179	0.079226	0.049535
**Integrin SIgnaling Pathway (Translocation to the nucleus)**	0.142895	0.244288	0.215129	0.049535
**Interactions Report**	0.578623	0.782185	0.860939	0.049535
**Interferon Main Pathway**	0.008951	0.01452	0.015733	0.049535
**Interferon Pathway (Gene expression)**	0	0	0	1
**Interferon Pathway (Transcription)**	0	0	0	1
**Interferon Pathway (Translation)**	0	0	0	1
**IP3 Main Pathway**	0.005077	0.006979	0.007318	0.12663
**IP3 Pathway (Gene expression)**	0.05769	0.07175	0.0586	0.049535
**JAK mStat Main Pathway**	0.102324	0.098236	0.088069	0.049535
**JAK mStat Pathway (Akt pathway)**	0.070377	0.088324	0.073467	0.049535
**JAK mStat Pathway (JAK degradation)**	-0.01752	-0.01946	-0.01775	0.049535
**JNK Main Pathway**	0.062224	0.076241	0.070894	0.049535
**JNK Pathway (Apoptosis, Inflammation, Tumorigenesis, Cell Migration)**	0.104868	0.107983	0.10856	0.049535
**JNK Pathway (Insulin signaling)**	0	0	0	1
**MAPK Family Main Pathway**	0.009919	0.018957	0.016386	0.049535
**MAPK Family Pathway (Chromatin Remodeling)**	-0.02981	-0.03145	-0.01618	0.049535
**MAPK Family Pathway (Cytoskeleton)**	-0.21509	-0.25315	-0.22666	0.049535
**MAPK Family Pathway (Gene Expression)**	-0.03805	-0.04624	-0.03437	0.049535
**MAPK Family Pathway (IKBs Degradation)**	0	0	0	1
**MAPK Family Pathway (Translation)**	0	0	0	1
**MAPK Signaling Main Pathway**	0.091843	0.112623	0.10969	0.049535
**MAPK Signaling Pathway (Cell Survival, Inflammation, Apoptosis, Osmoregulation)**	0.163641	0.169069	0.157371	0.049535
**MAPK Signaling Pathway (Gene Expression)**	0.063538	0.066093	0.067598	0.049535

**DDR pathway (MMR)**	0.682608	0.669981	0.574641	0.049535
**Mismatch Repair Main Pathway**	0.827316	0.756464	0.635162	0.049535
**Mitochondrial Apopotosis Main Pathway**	-0.07026	-0.09064	-0.08929	0.049535
**Mitochondrial Apopotosis Pathway (Apoptosis)**	0.088025	0.099697	0.073153	0.049535
**Mitochondrial Apopotosis Pathway (Depolarization)**	0	0	0	1
**Mitochondrial Apopotosis Pathway (DNA fragmentation)**	-0.21059	-0.21432	-0.28908	0.049535
**Mitochondrial Apopotosis Pathway (Gene expression)**	-0.25517	-0.29411	-0.25469	0.049535
**mTOR Main Pathway**	0.038914	0.04882	0.049056	0.049535
**mTOR Pathway (Actin organization)**	0.022779	-0.00069	0.003529	0.512691
**mTOR Pathway (Akt signaling)**	0	0	0	1
**mTOR Pathway (Scanning)**	0	0	0	1
**mTOR Pathway (Translation on)**	0	0	0	1
**mTOR Pathway (VEGF pathway)**	0	0	0	1
**NGF (Negative) Main Pathway**	0	0	0	1
**NGF (Negative) Pathway (Apoptosis)**	0	0	0	1
**NGF (Positive) Main Pathway**	0.034782	0.039594	0.040136	0.049535
**NHEJ mechanisms of DSBs repair effect**	0.307716	0.305916	0.277011	0.049535
**Notch Main Pathway**	0.04782	0.040677	0.04391	0.049535
**DDR Pathway (NER)**	0.270945	0.241722	0.206078	0.049535
**p38 (Negative) Main Signaling Pathway**	0.038729	0.048362	0.044614	0.049535
**p38 (Positive) Main Signaling Pathway**	0.038651	0.048264	0.044523	0.049535
**p53 Signaling (Negative) Main Pathway**	-0.02161	0.011315	0.017708	0.512691
**p53 Signaling (Negative) Pathway (p53 Degradation)**	0.028589	0.031072	0.02791	0.049535
**PAK Main Pathway**	0.060591	0.075751	0.076415	0.049535
**PAK Pathway (Actin Cytoskeleton)**	0.055441	0.06859	0.064769	0.049535
**PAK Pathway (Myosin Activation)**	0.082634	0.088723	0.092795	0.049535
**PPAR Main Pathway**	0.0566	0.072871	0.066157	0.049535
**PTEN Main Pathway**	-0.0266	-0.0272	-0.02618	0.049535
**RANK Signaling in Osteoclast Main Pathway**	0.061532	0.077901	0.074681	0.049535
**RANK Signaling in Osteoclast Pathway (IKBs Degradation)**	0	0	0	1
**RAS Main Pathway**	0.101786	0.116192	0.107033	0.049535
**Telomere Main Pathway**	0.027822	0.011676	0.020336	0.049535
**RNA Polymerase II Complex Pathway**	0.017399	0.014814	0.016368	0.049535
**Cell Cycle Pathway (SCC during S-phase)**	-0.11923	-0.24614	-0.21441	0.049535
**SMAD (Negative) Main Pathway**	0.066723	0.076881	0.075778	0.049535
**SMAD (Negative) Pathway (Degradation)**	0.014275	0.017558	0.015113	0.049535
**SMAD (Positive) Main Pathway**	0.066723	0.076881	0.075778	0.049535
**SMAD (Positive) Pathway (Degradation)**	0.014275	0.017558	0.015113	0.049535
**Cell Cycle Pathway (Metaphase-Anaphase)**	0.519492	0.599195	0.554786	0.049535
**Cell Cycle Pathway (Origin of S-phase)**	1.263029	1.136616	1.088144	0.049535
**STAT3 Main Pathway**	0.046231	0.063103	0.057581	0.049535
**TGF beta Main Pathway**	0.037198	0.04914	0.048553	0.049535
**TGF beta Pathway (Epithelial mesehchymal transition)**	0.076687	0.121514	0.10297	0.049535
**TGF beta Pathway (Post-transcriptional G1 arrest)**	0.033118	0.040736	0.035063	0.049535
**TGF beta Pathway (SnON degradation)**	0	0	0	1
**TGF beta Pathway (Tumorigenesis)**	0	0	0	1
**TGF beta Pathway (Tumor suppression)**	0	0	0	1
**TNF (Negative) Main Pathway**	0.026489	0.019047	0.00049	0.049535
**TNF (Negative) Pathway (Apoptosis)**	0.029605	0.021288	0.000547	0.049535
**TNF (Positive) Main Pathway**	0.046462	0.046354	0.046508	0.049535
**TNF (Positive) Pathway (Gene expression, Cell survival)**	0.094322	0.103527	0.104914	0.049535

**TNF (Positive) Pathway (IKBs degradation)**	0	0	0	1
**TRAF (Negative) Main Pathway**	-0.0553	-0.08345	-0.08685	0.049535
**TRAF (Negative) Pathway (IKBs Degradation)**	0	0	0	1
**TRAF (Positive) Main Pathway**	-0.00643	-0.01799	-0.01405	0.049535
**TRAF (Positive) Pathway (IKBs Degradation)**	0	0	0	1
**Transcription of mRNA Pathway**	0.020299	0.017283	0.019096	0.049535
**Cell Cycle Pathway (End of S-phase)**	0.035394	-0.00141	-0.0088	0.827259
**Translation Regulation of EIF4F activity**	0.003177	-0.00783	-0.00851	0.512691
**Ubiquitin Proteasome Main Pathway**	0.107772	0.117022	0.105482	0.049535
**Ubiquitin Proteasome Pathway (Degraded Protein)**	0.174239	0.19316	0.167847	0.049535
**VEGF Main Pathway**	0.031011	0.01878	0.022261	0.049535
**VEGF Pathway (Actin Reorganization)**	0.101722	0.121117	0.118001	0.049535
**VEGF Pathway (Nitric Oxide Production)**	0	0	0	1
**Wnt Main Pathway**	0.076756	0.076768	0.072887	0.049535
**Wnt Pathway (Ctnn-b Degradation)**	0	0	0	1
**IL-6 Pathway (IKBs degradation)**	0	0	0	1

Supplemental Data S-3

Heatmap 8h post 68% (red bars) and 86% (green bars) (1st part)

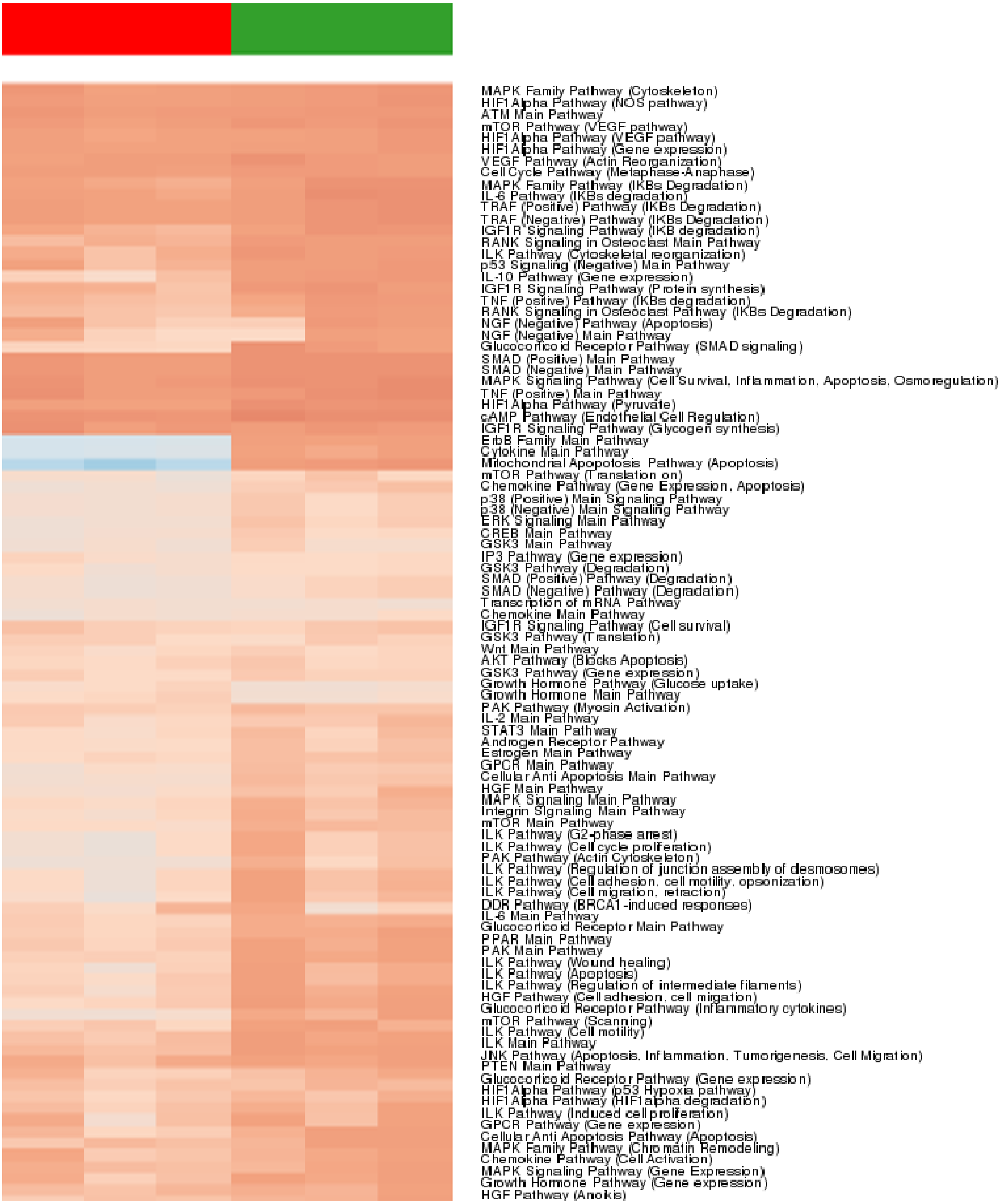

Borger et al
Supplemental Data

<u>Heatmap 8h post 68% (red bars) and 86% (green bars) (2nd part)</u>

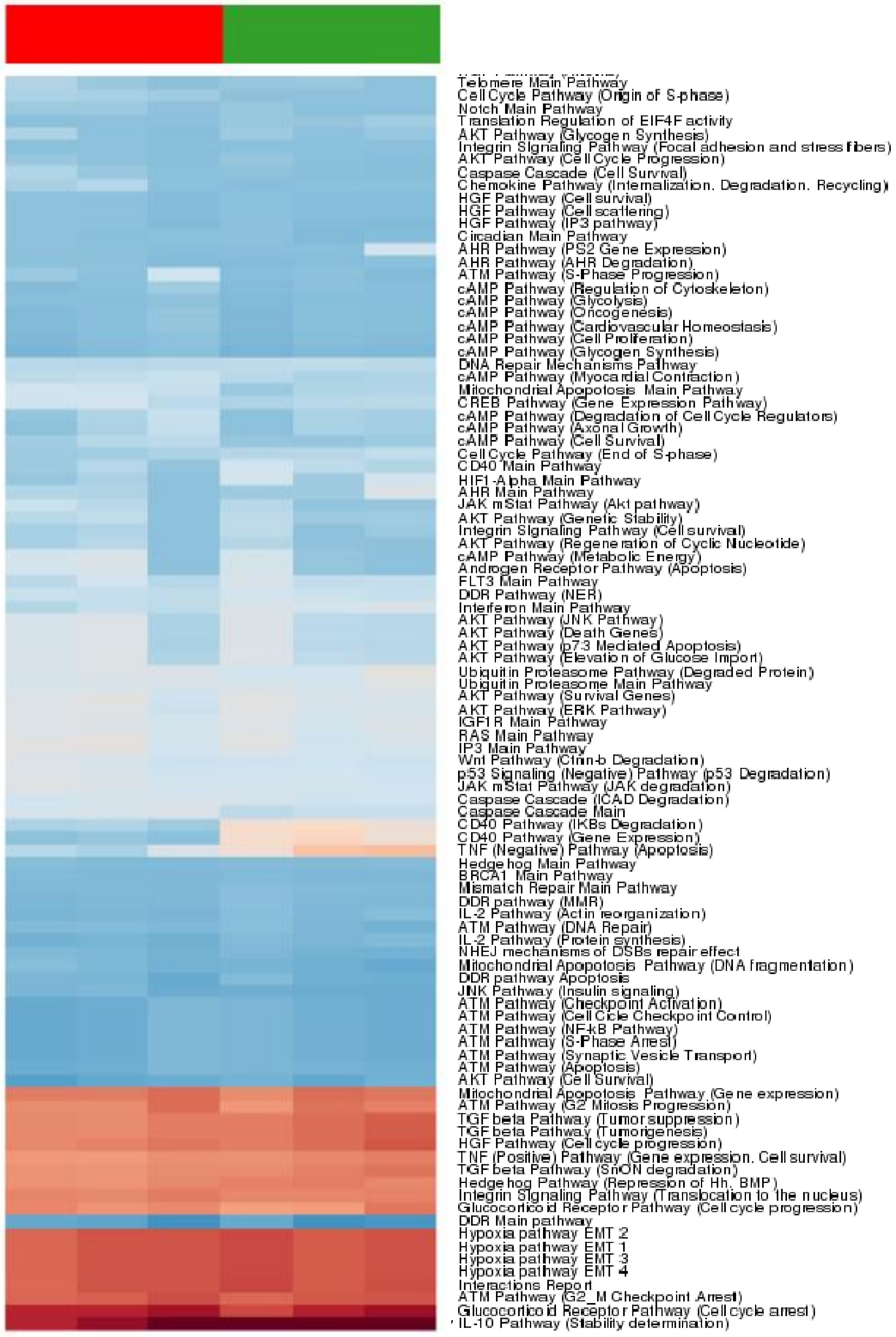

Heatmap 16h post 68% (red bars) and 86% (green bars) (1st part)

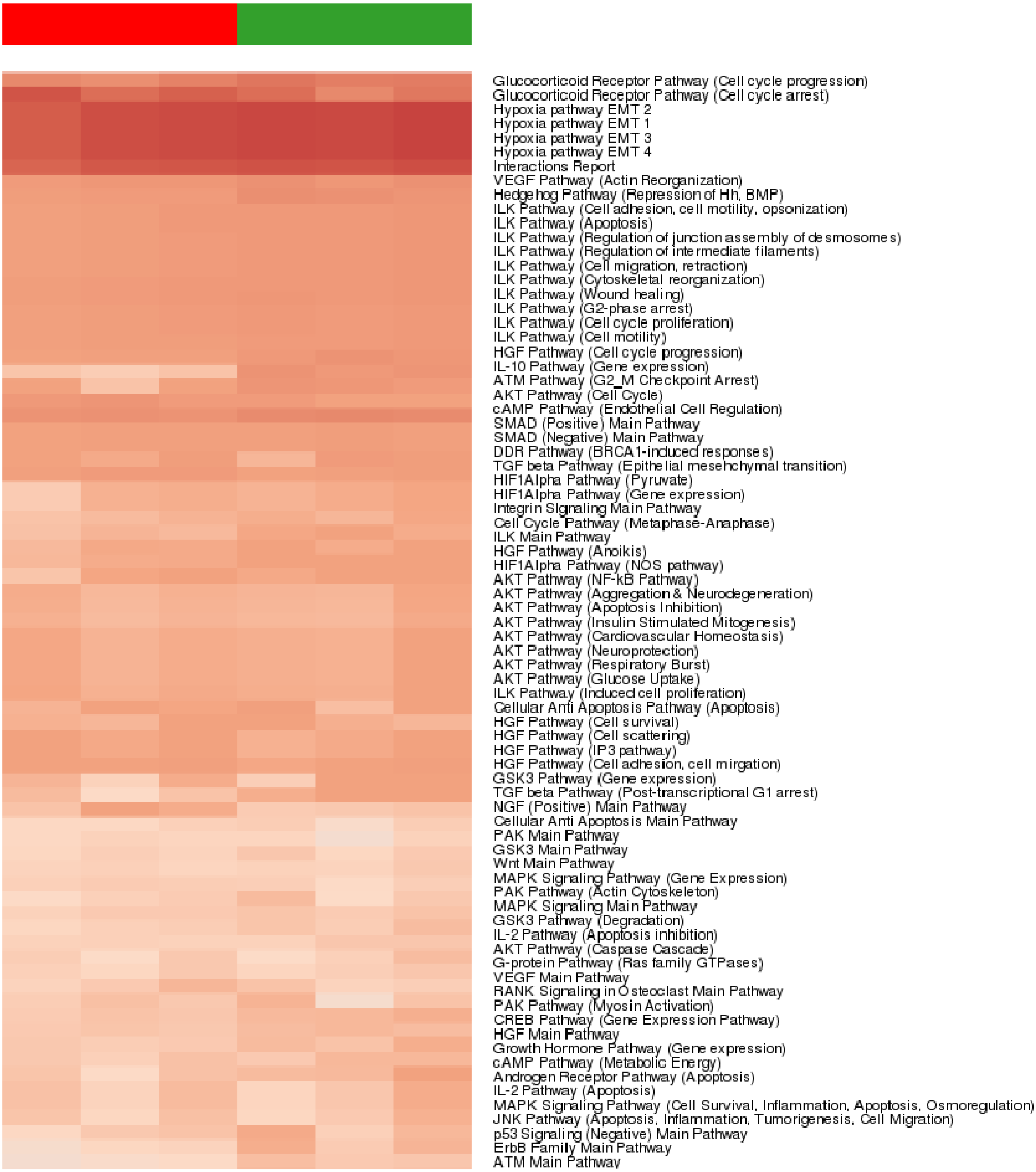

Heatmap 16h post 68% (red bars) and 86% (green bars) (2nd part)

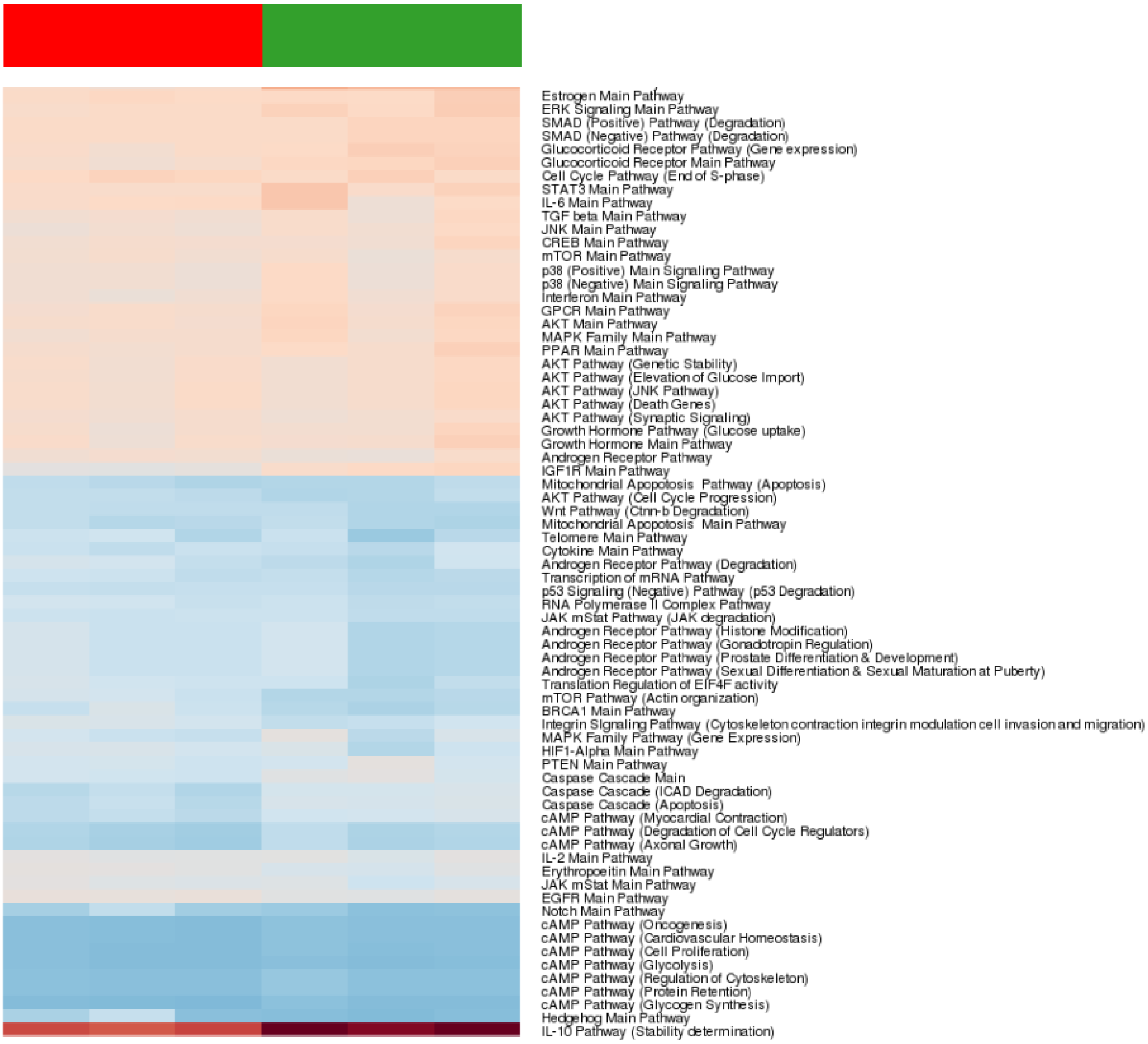
Estrogen Main Pathway
ERK Signaling Main Pathway
SMAD (Positive) Pathway (Degradation)
SMAD (Negative) Pathway (Degradation)
Glucocorticoid Receptor Pathway (Gene expression)
Glucocorticoid Receptor Main Pathway
Cell Cycle Pathway (End of S-phase)
STAT3 Main Pathway
IL-6 Main Pathway
TGF beta Main Pathway
JNK Main Pathway
CREB Main Pathway
mTOR Main Pathway
p38 (Positive) Main Signaling Pathway
p38 (Negative) Main Signaling Pathway
Interferon Main Pathway
GPCR Main Pathway
AKT Main Pathway
MAPK Family Main Pathway
PPAR Main Pathway
AKT Pathway (Genetic Stability)
AKT Pathway (Elevation of Glucose Import)
AKT Pathway (JNK Pathway)
AKT Pathway (Death Genes)
AKT Pathway (Synaptic Signaling)
Growth Hormone Pathway (Glucose uptake)
Growth Hormone Main Pathway
Androgen Receptor Pathway
IGF1R Main Pathway
Mitochondrial Apoptosis Pathway (Apoptosis)
AKT Pathway (Cell Cycle Progression)
Wnt Pathway (Ctnn-b Degradation)
Mitochondrial Apoptosis Main Pathway
Telomere Main Pathway
Cytokine Main Pathway
Androgen Receptor Pathway (Degradation)
Transcription of mRNA Pathway
p53 Signaling (Negative) Pathway (p53 Degradation)
RNA Polymerase II Complex Pathway
JAK mStat Pathway (JAK degradation)
Androgen Receptor Pathway (Histone Modification)
Androgen Receptor Pathway (Gonadotropin Regulation)
Androgen Receptor Pathway (Prostate Differentiation & Development)
Androgen Receptor Pathway (Sexual Differentiation & Sexual Maturation at Puberty)
Translation Regulation of EIF4F activity
mTOR Pathway (Actin organization)
BRCA1 Main Pathway
Integrin Signaling Pathway (Cytoskeleton contraction integrin modulation cell invasion and migration)
MAPK Family Pathway (Gene Expression)
HIF1-Alpha Main Pathway
PTEN Main Pathway
Caspase Cascade Main
Caspase Cascade (ICAD Degradation)
Caspase Cascade (Apoptosis)
cAMP Pathway (Myocardial Contraction)
cAMP Pathway (Degradation of Cell Cycle Regulators)
cAMP Pathway (Axonal Growth)
IL-2 Main Pathway
Erythropoeitin Main Pathway
JAK mStat Main Pathway
EGFR Main Pathway
Notch Main Pathway
cAMP Pathway (Oncogenesis)
cAMP Pathway (Cardiovascular Homeostasis)
cAMP Pathway (Cell Proliferation)
cAMP Pathway (Glycolysis)
cAMP Pathway (Regulation of Cytoskeleton)
cAMP Pathway (Protein Retention)
cAMP Pathway (Glycogen Synthesis)
Hedgehog Main Pathway
IL-10 Pathway (Stability determination)

Heatmap 32h post 68% (red bars) and 86% (green bars) (1st part)

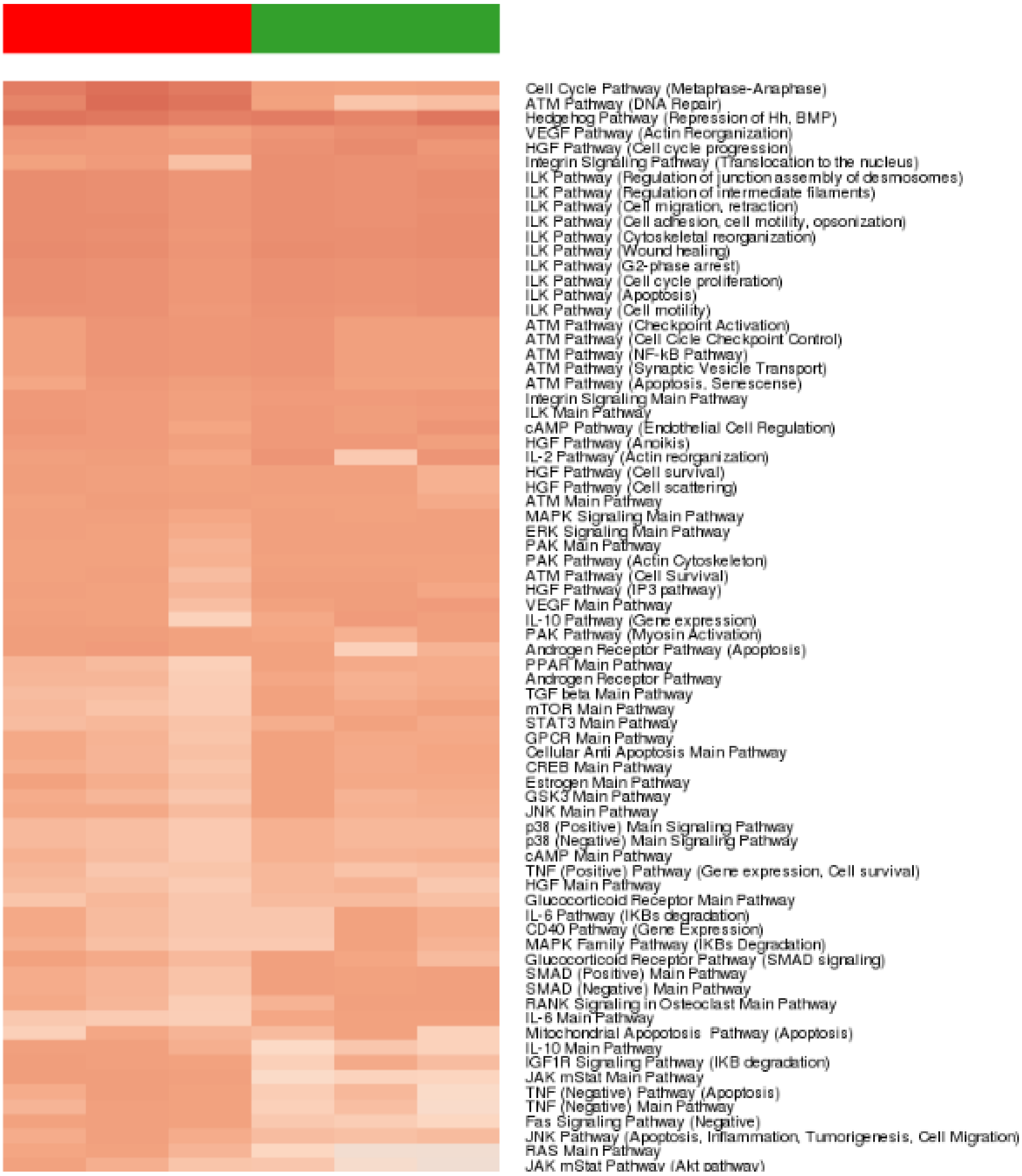

Heatmap 32h post 68% (red bars) and 86% (green bars) (2nd part)

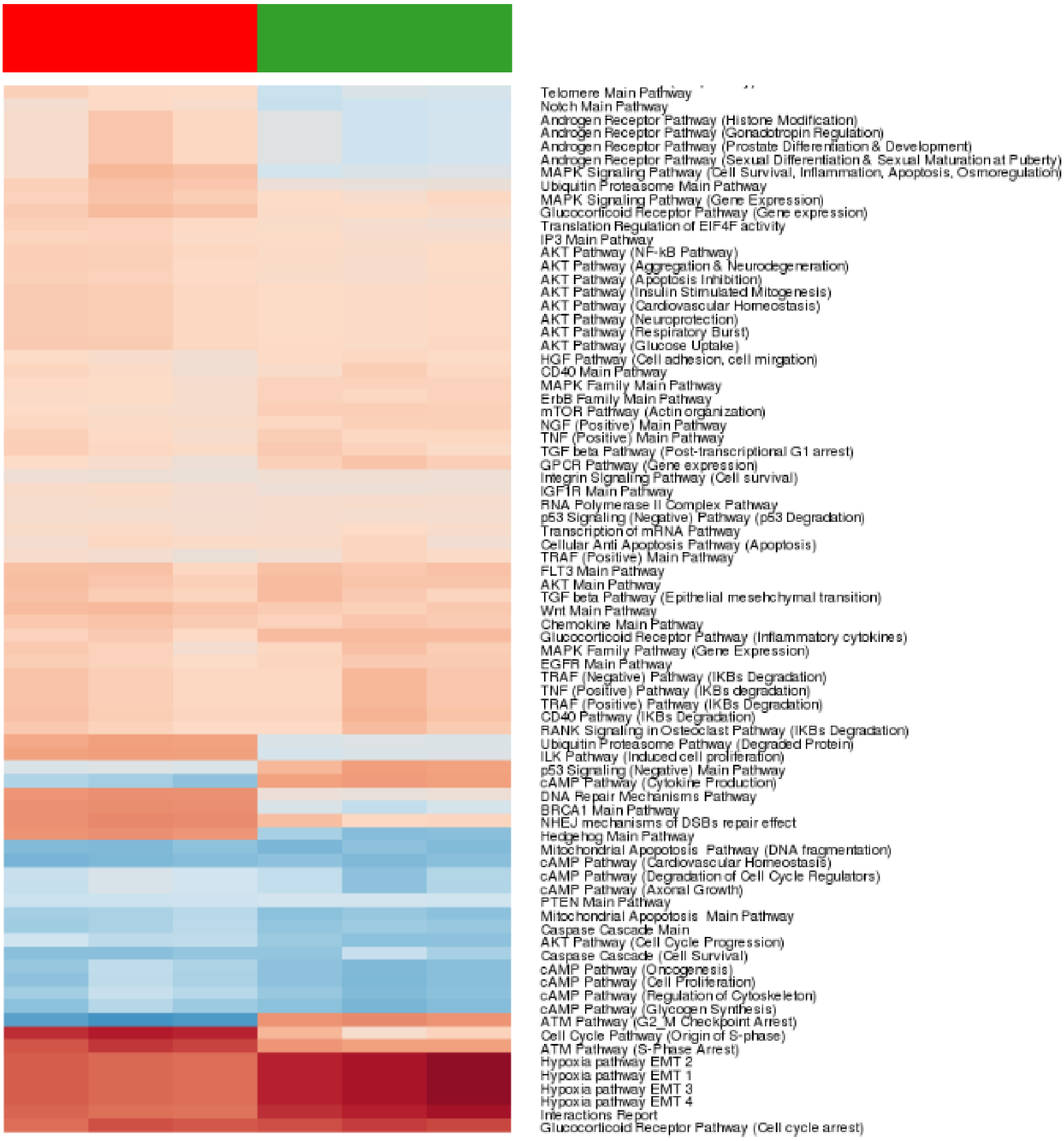

Heatmap 48h post 68% (red bars) and 86% (green bars) (1st part)

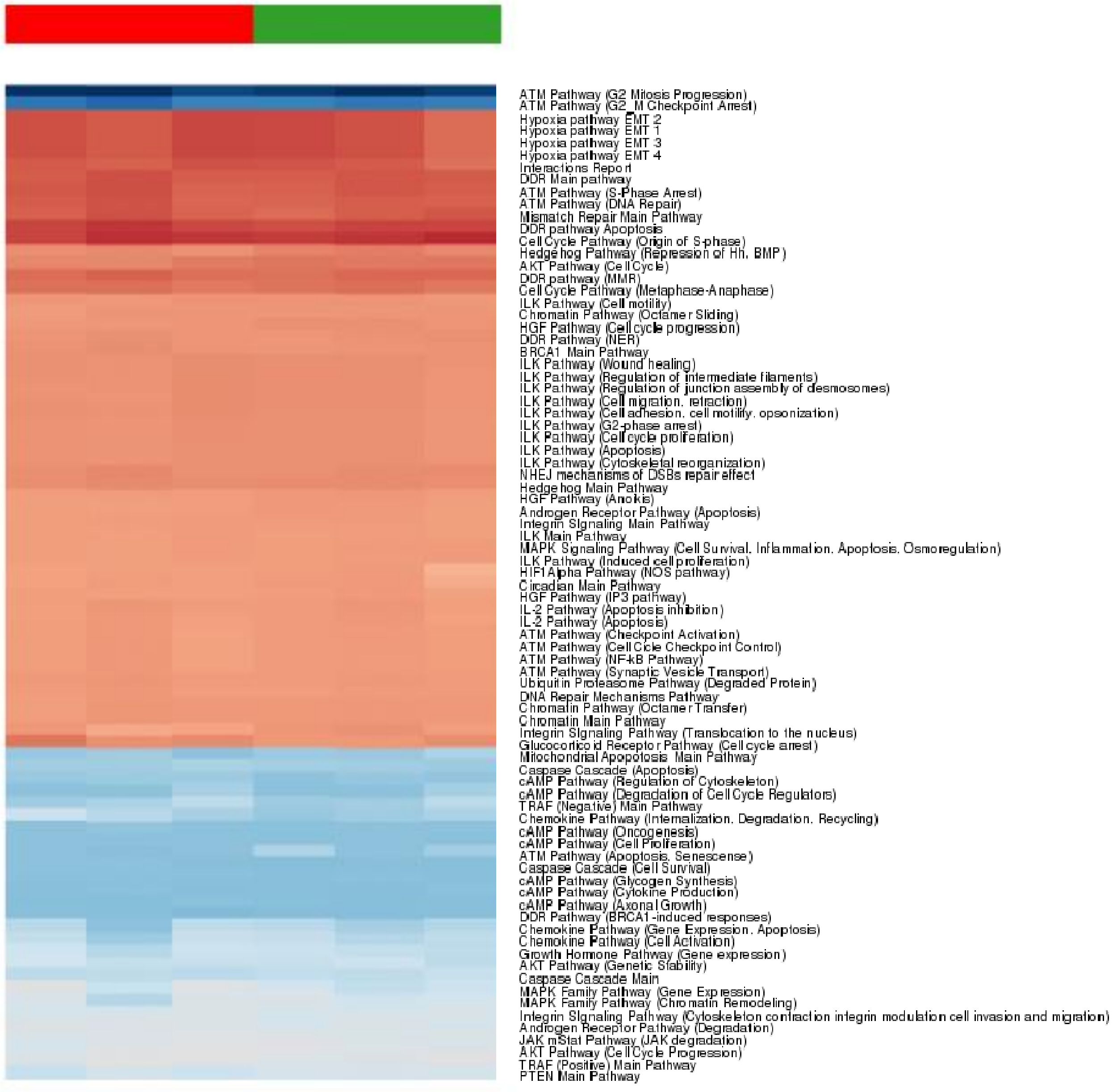

Borger et al
Supplemental Data

<u>Heatmap 48h post 68% (red bars) and 86% (green bars) (2nd part)</u>

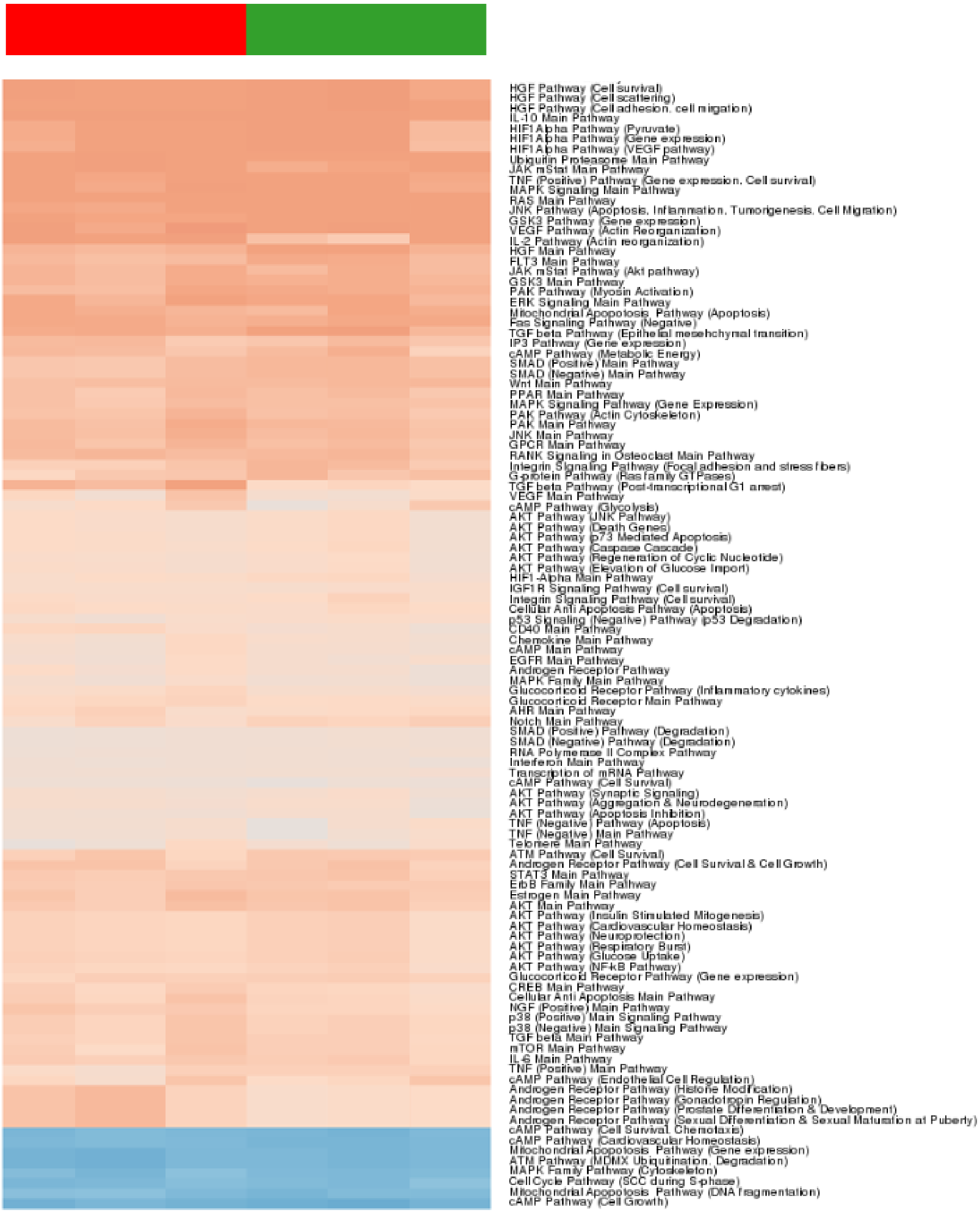

Supplemental Data S-4

Signaling Pathways (total = 269)

First split PWs in up- and downregulated groups

Filter: all experiments same sign (n=3); p<0.05

A: Unique and shared Signaling Pathways after 68% and 86% hepatectomy (t=1h post OP)

1) T=1h; activated signaling pathways (p<0.05)

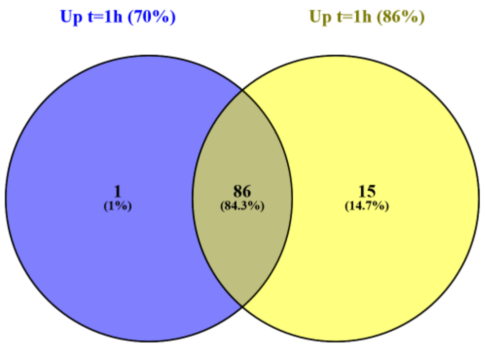

1 Unique activated Signaling Pathway after 68% Hx (1h post OP):

Circadian Main Pathway

15 Unique activated Signaling Pathways after 86% Hx (1h post OP):

ATM Main Pathway

ATM Pathway (Cell Survival)

ATM Pathway (G2_M Checkpoint Arrest)

Fas Signaling Pathway (Negative)

HIF1-Alpha Main Pathway

HIF1Alpha Pathway (Gene expression)

HIF1Alpha Pathway (NOS pathway)

HIF1Alpha Pathway (Pyruvate)

HIF1Alpha Pathway (VEGF pathway)

ILK Pathway (Cytoskeletal reorganization)

ILK Pathway (Induced cell proliferation)

JNK Pathway (Apoptosis, Inflammation, Tumorigenesis, Cell Migration)

MAPK Signaling Pathway (Cell Survival, Inflammation, Apoptosis, Osmoregulation)

VEGF Main Pathway

VEGF Pathway (Actin Reorganization)

86 common (shared) activated Signaling PW (1h post OP):

AKT Main Pathway

AKT Pathway (Aggregation & Neurodegeneration)

AKT Pathway (Apoptosis Inhibition)

AKT Pathway (Cardiovascular Homeostasis)

AKT Pathway (Cell Cycle Progression)

AKT Pathway (Glucose Uptake)

AKT Pathway (Insulin Stimulated Mitogenesis)

AKT Pathway (Neuroprotection)

AKT Pathway (NF-kB Pathway)

AKT Pathway (Respiratory Burst)

Androgen Receptor Pathway

Androgen Receptor Pathway (Degradation)

Androgen Receptor Pathway (Gonadotropin Regulation)

Androgen Receptor Pathway (Histone Modification)

Androgen Receptor Pathway (Prostate Differentiation & Development)

Androgen Receptor Pathway (Sexual Differentiation & Sexual Maturation at Puberty)

BRCA1 Main Pathway

cAMP Main Pathway

CD40 Main Pathway

CD40 Pathway (Gene Expression)

CD40 Pathway (IKBs Degradation)

Cellular Anti Apoptosis Main Pathway

Chemokine Main Pathway

CREB Main Pathway

Cytokine Main Pathway

EGFR Main Pathway

ErbB Family Main Pathway

ERK Signaling Main Pathway

Erythropoeitin Main Pathway

Estrogen Main Pathway

Glucocorticoid Receptor Main Pathway

Glucocorticoid Receptor Pathway (Inflammatory cytokines)

GPCR Main Pathway

GPCR Pathway (Gene expression)

GSK3 Main Pathway

Hedgehog Pathway (Repression of Hh, BMP)

HGF Main Pathway

HGF Pathway (Anoikis)

HGF Pathway (Cell cycle progression)

IGF1R Main Pathway

IGF1R Signaling Pathway (IKB degradation)

ILK Main Pathway

IL-10 Main Pathway

IL-10 Pathway (Gene expression)

IL-2 Main Pathway

IL-2 Pathway (Actin reorganization)

IL-2 Pathway (Protein synthesis)

IL-6 Main Pathway

Integrin SIgnaling Main Pathway

Integrin SIgnaling Pathway (Cytoskeleton contraction integrin modulation cell invasion and migration)

Interferon Main Pathway

IP3 Main Pathway

JAK mStat Main Pathway

MAPK Family Main Pathway

MAPK Family Pathway (Gene Expression)

MAPK Family Pathway (IKBs Degradation)

MAPK Signaling Main Pathway

MAPK Signaling Pathway (Gene Expression)

mTOR Main Pathway

mTOR Pathway (Actin organization)

NGF (Positive) Main Pathway

p38 (Negative) Main Signaling Pathway

p38 (Positive) Main Signaling Pathway

p53 Signaling (Negative) Main Pathway

PAK Main Pathway

PAK Pathway (Actin Cytoskeleton)

PPAR Main Pathway

RANK Signaling in Osteoclast Main Pathway

RANK Signaling in Osteoclast Pathway (IKBs Degradation)

RAS Main Pathway

Telomere Main Pathway

SMAD (Negative) Main Pathway

SMAD (Positive) Main Pathway

STAT3 Main Pathway

TGF beta Main Pathway

TGF beta Pathway (SnON degradation)

TGF beta Pathway (Tumorigenesis)

TGF beta Pathway (Tumor suppression)

TNF (Positive) Main Pathway

TNF (Positive) Pathway (Gene expression, Cell survival)

TNF (Positive) Pathway (IKBs degradation)

TRAF (Negative) Pathway (IKBs Degradation)

TRAF (Positive) Main Pathway

TRAF (Positive) Pathway (IKBs Degradation)

Wnt Main Pathway

IL-6 Pathway (IKBs degradation)

2) T=1h; silenced signaling pathways ($p<0.05$)

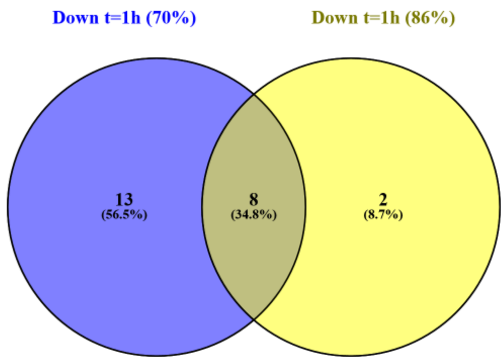

<u>13 Unique silenced Signaling PW after 68% Hx (1h post OP):</u>

ATM Main Pathway

ATM Pathway (G2_M Checkpoint Arrest)

GSK3 Main Pathway

ILK Pathway (Apoptosis)

ILK Pathway (Cell adhesion, cell motility, opsonization)

ILK Pathway (Cell cycle proliferation)

ILK Pathway (Cell migration, retraction)

ILK Pathway (Cell motility)

ILK Pathway (G2-phase arrest)

ILK Pathway (Regulation of intermediate filaments)

ILK Pathway (Regulation of junction assembly of desmosomes)

ILK Pathway (Wound healing)

JNK Main Pathway

2 Unique silenced Signaling PW after 86% Hx (1h post OP):

Circadian Main Pathway

DNA Repair Mechanisms Pathway

8 common (shared) silenced Signaling PW (1h post OP):

DDR Pathway (BRCA1-induced responses)

Caspase Cascade Main

Glucocorticoid Receptor Pathway (Cell cycle arrest)

Growth Hormone Main Pathway

Growth Hormone Pathway (Glucose uptake)

IL-10 Pathway (Translational modulation)

JNK Pathway (Insulin signaling)

Mitochondrial Apoptosis Main Pathway

B: Unique and shared Signaling Pathways after 68% and 86% hepatectomy (t=8h post OP)

1) T=8h; activated signaling pathways (p<0.05)

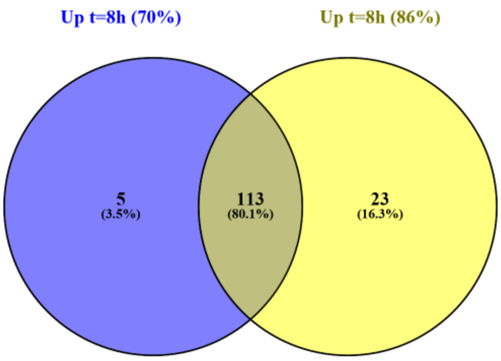

5 Unique activated Signaling Pathway after 68% Hx (8h post OP):

Caspase Cascade Main

JNK Pathway (Insulin signaling)

Mitochondrial Apoptosis Main Pathway

ATM Pathway (MDMX Ubiquitination, Degradation)

G-protein Pathway (Ras family GTPases)

23 Unique activated Signaling Pathways after 86% Hx (8h post OP):

AKT Main Pathway

Androgen Receptor Pathway (Gonadotropin Regulation)

Androgen Receptor Pathway (Histone Modification)

Androgen Receptor Pathway (Prostate Differentiation & Development)

Androgen Receptor Pathway (Sexual Differentiation & Sexual Maturation at Puberty)

cAMP Main Pathway

CD40 Pathway (Gene Expression)

CD40 Pathway (IKBs Degradation)

Cytokine Main Pathway

ErbB Family Main Pathway

IL-10 Main Pathway

Integrin SIgnaling Pathway (Cytoskeleton contraction, integrin modulation, cell invasion and migration)

Interferon Pathway (Translation)

MAPK Family Main Pathway

MAPK Family Pathway (Gene Expression)

Mitochondrial Apoptosis Pathway (Apoptosis)

TGF beta Main Pathway

TGF beta Pathway (Epithelial mesehchymal transition)

TGF beta Pathway (Post-transcriptional G1 arrest)

TNF (Negative) Main Pathway

TNF (Negative) Pathway (Apoptosis)

TRAF (Positive) Main Pathway

VEGF Main Pathway

113 common (shared) activated Signaling PW (8h post OP):

ATM Main Pathway

ATM Pathway (G2_M Checkpoint Arrest)

DDR Pathway (BRCA1-induced responses)

Glucocorticoid Receptor Pathway (Cell cycle arrest)

Growth Hormone Main Pathway

Growth Hormone Pathway (Glucose uptake)

GSK3 Main Pathway

ILK Pathway (Apoptosis)

ILK Pathway (Cell adhesion, cell motility, opsonization)

ILK Pathway (Cell cycle proliferation)

ILK Pathway (Cell migration, retraction)

ILK Pathway (Cell motility)

ILK Pathway (G2-phase arrest)

ILK Pathway (Regulation of intermediate filaments)

ILK Pathway (Regulation of junction assembly of desmosomes)

ILK Pathway (Wound healing)

IL-10 Pathway (Translational modulation)

JNK Main Pathway

AKT Pathway (Blocks Apoptosis)

Androgen Receptor Pathway

ATM Pathway (G2 Mitosis Progression)

cAMP Pathway (Endothelial Cell Regulation)

Cellular Anti Apoptosis Main Pathway

Cellular Anti Apoptosis Pathway (Apoptosis)

Chemokine Main Pathway

Chemokine Pathway (Cell Activation)

Chemokine Pathway (Gene Expression, Apoptosis)

CREB Main Pathway

ERK Signaling Main Pathway

Estrogen Main Pathway

Glucocorticoid Receptor Main Pathway

Glucocorticoid Receptor Pathway (Cell cycle progression)

Glucocorticoid Receptor Pathway (Gene expression)

Glucocorticoid Receptor Pathway (Inflammatory cytokines)

Glucocorticoid Receptor Pathway (SMAD signaling)

GPCR Main Pathway

GPCR Pathway (Gene expression)

Growth Hormone Pathway (Gene expression)

GSK3 Pathway (Degradation)

GSK3 Pathway (Gene expression)

GSK3 Pathway (Translation)

Hedgehog Pathway (Repression of Hh, BMP)

HGF Main Pathway

HGF Pathway (Anoikis)

HGF Pathway (Cell adhesion, cell mirgation)

HGF Pathway (Cell cycle progression)

HIF1Alpha Pathway (Gene expression)

HIF1Alpha Pathway (HIF1alpha degradation)

HIF1Alpha Pathway (NOS pathway)

HIF1Alpha Pathway (p53 Hypoxia pathway)

HIF1Alpha Pathway (Pyruvate)

HIF1Alpha Pathway (VEGF pathway)

Hypoxia pathway EMT 1

Hypoxia pathway EMT 2

Hypoxia pathway EMT 3

Hypoxia pathway EMT 4

IGF1R Signaling Pathway (Cell survival)

IGF1R Signaling Pathway (Glycogen synthesis)

IGF1R Signaling Pathway (IKB degradation)

IGF1R Signaling Pathway (Protein synthesis)

ILK Main Pathway

ILK Pathway (Cytoskeletal reorganization)

ILK Pathway (Induced cell proliferation)

IL-10 Pathway (Gene expression)

IL-10 Pathway (Stability determination)

IL-2 Main Pathway

IL-6 Main Pathway

Integrin SIgnaling Main Pathway

Integrin SIgnaling Pathway (Translocation to the nucleus)

Interactions Report

IP3 Pathway (Gene expression)

JNK Pathway (Apoptosis, Inflammation, Tumorigenesis, Cell Migration)

MAPK Family Pathway (Chromatin Remodeling)

MAPK Family Pathway (Cytoskeleton)

MAPK Family Pathway (IKBs Degradation)

MAPK Signaling Main Pathway

MAPK Signaling Pathway (Cell Survival, Inflammation, Apoptosis, Osmoregulation)

MAPK Signaling Pathway (Gene Expression)

Mitochondrial Apopotosis Pathway (Gene expression)

mTOR Main Pathway

mTOR Pathway (Scanning)

mTOR Pathway (Translation on)

mTOR Pathway (VEGF pathway)

NGF (Negative) Main Pathway

NGF (Negative) Pathway (Apoptosis)

p38 (Negative) Main Signaling Pathway

p38 (Positive) Main Signaling Pathway

p53 Signaling (Negative) Main Pathway

PAK Main Pathway

PAK Pathway (Actin Cytoskeleton)

PAK Pathway (Myosin Activation)

PPAR Main Pathway

PTEN Main Pathway

RANK Signaling in Osteoclast Main Pathway

RANK Signaling in Osteoclast Pathway (IKBs Degradation)

SMAD (Negative) Main Pathway

SMAD (Negative) Pathway (Degradation)

SMAD (Positive) Main Pathway

SMAD (Positive) Pathway (Degradation)

Cell Cycle Pathway (Metaphase-Anaphase)

STAT3 Main Pathway

TGF beta Pathway (SnON degradation)

TGF beta Pathway (Tumorigenesis)

TGF beta Pathway (Tumor suppression)

TNF (Positive) Main Pathway

TNF (Positive) Pathway (Gene expression, Cell survival)

TNF (Positive) Pathway (IKBs degradation)

TRAF (Negative) Pathway (IKBs Degradation)

TRAF (Positive) Pathway (IKBs Degradation)

Transcription of mRNA Pathway

VEGF Pathway (Actin Reorganization)

Wnt Main Pathway

IL-6 Pathway (IKBs degradation)

2) T=8h; silenced signaling pathways (p<0.05)

Borger et al
Supplemental Data

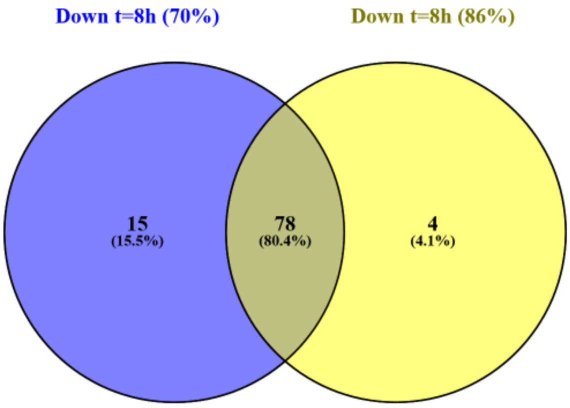

15 Unique silenced Signaling PW after 68% Hx (8h post OP):

AHR Pathway (Cath-D Expression)

AHR Pathway (C-MycExpression)

Caspase Cascade (Activated Tissue Transglutaminase)

CD40 Pathway (Gene Expression)

CD40 Pathway (IKBs Degradation)

Cytokine Main Pathway

ErbB Family Main Pathway

Erythropoeitin Main Pathway

IL-2 Pathway (Apoptosis inhibition)

JAK mStat Main Pathway

MAPK Family Main Pathway

Mitochondrial Apopotosis Pathway (Apoptosis)

NGF (Positive) Main Pathway

TNF (Negative) Pathway (Apoptosis)

TRAF (Negative) Main Pathway

4 Unique silenced Signaling PW after 86% Hx (8h post OP):

Androgen Receptor Pathway (Cell Survival & Cell Growth)

Chromatin Main Pathway

Chromatin Pathway (Octamer Sliding)

Chromatin Pathway (Octamer Transfer)

78 common (shared) silenced Signaling PW (8h post OP):

AHR Main Pathway

AHR Pathway (AHR Degradation)

AHR Pathway (PS2 Gene Expression)

AKT Pathway (Cell Cycle Progression)

AKT Pathway (Cell Survival)

AKT Pathway (Death Genes)

AKT Pathway (Elevation of Glucose Import)

AKT Pathway (ERK Pathway)

AKT Pathway (Genetic Stability)

AKT Pathway (Glycogen Synthesis)

AKT Pathway (JNK Pathway)

AKT Pathway (p73 Mediated Apoptosis)

AKT Pathway (Regeneration of Cyclic Nucleotide)

AKT Pathway (Survival Genes)

Androgen Receptor Pathway (Apoptosis)

ATM Pathway (Apoptosis)

ATM Pathway (Cell Cicle Checkpoint Control)

ATM Pathway (Checkpoint Activation)

ATM Pathway (DNA Repair)

ATM Pathway (NF-kB Pathway)

ATM Pathway (Synaptic Vesicle Transport)

ATM Pathway (S-Phase Arrest)

ATM Pathway (S-Phase Progression)

BRCA1 Main Pathway

cAMP Pathway (Axonal Growth)

cAMP Pathway (Cardiovascular Homeostasis)

cAMP Pathway (Cell Proliferation)

cAMP Pathway (Cell Survival)

cAMP Pathway (Degradation of Cell Cycle Regulators)

cAMP Pathway (Glycogen Synthesis)

cAMP Pathway (Glycolysis)

cAMP Pathway (Metabolic Energy)

cAMP Pathway (Myocardial Contraction)

cAMP Pathway (Oncogenesis)

cAMP Pathway (Regulation of Cytoskeleton)

Caspase Cascade Main

Caspase Cascade (Cell Survival)

Caspase Cascade (ICAD Degradation)

CD40 Main Pathway

Chemokine Pathway (Internalization, Degradation, Recycling)

Circadian Main Pathway

CREB Pathway (Gene Expression Pathway)

DDR pathway Apoptosis

DDR Main pathway

DNA Repair Mechanisms Pathway

FLT3 Main Pathway

Hedgehog Main Pathway

HGF Pathway (Cell scattering)

HGF Pathway (Cell survival)

HGF Pathway (IP3 pathway)

HIF1-Alpha Main Pathway

IGF1R Main Pathway

IL-2 Pathway (Actin reorganization)

IL-2 Pathway (Protein synthesis)

Integrin SIgnaling Pathway (Cell survival)

Integrin SIgnaling Pathway (Focal adhesion and stress fibers)

Interferon Main Pathway

Interferon Pathway (Gene expression)

IP3 Main Pathway

JAK mStat Pathway (Akt pathway)

JAK mStat Pathway (JAK degradation)

JNK Pathway (Insulin signaling)

DDR pathway (MMR)

Mismatch Repair Main Pathway

Mitochondrial Apopotosis Main Pathway

Mitochondrial Apopotosis Pathway (DNA fragmentation)

NHEJ mechanisms of DSBs repair effect

Notch Main Pathway

DDR Pathway (NER)

p53 Signaling (Negative) Pathway (p53 Degradation)

RAS Main Pathway

Telomere Main Pathway

Cell Cycle Pathway (Origin of S-phase)

Cell Cycle Pathway (End of S-phase)

Translation Regulation of EIF4F activity

Ubiquitin Proteasome Main Pathway

Ubiquitin Proteasome Pathway (Degraded Protein)

Wnt Pathway (Ctnn-b Degradation)

C: Unique and shared Signaling Pathways after 68% and 86% hepatectomy (t=16h post OP)

1) T=16h; activated signaling pathways (p<0.05)

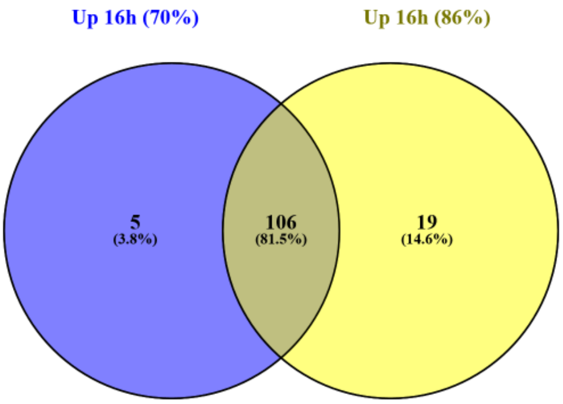

5 Unique activated Signaling PW after 68% Hx (16h post OP):

Chemokine Pathway (Internalization, Degradation, Recycling)

DNA Repair Mechanisms Pathway

EGFR Main Pathway

FLT3 Main Pathway

IL-10 Main Pathway

19 Unique activated Signaling PW after 86% Hx (16h post OP):

ATM Main Pathway

ATM Pathway (Cell Survival)

cAMP Pathway (Cytokine Production)

Caspase Cascade (Activated Tissue Transglutaminase)

Chemokine Main Pathway

Chemokine Pathway (Cell Activation)

Chemokine Pathway (Gene Expression, Apoptosis)

Circadian Main Pathway

Glucocorticoid Receptor Pathway (Inflammatory cytokines)

IGF1R Main Pathway

IL-10 Pathway (Translational modulation)

Integrin SIgnaling Pathway (Translocation to the nucleus)

Interferon Pathway (Gene expression)

IP3 Main Pathway

JAK mStat Pathway (Akt pathway)

MAPK Family Pathway (Chromatin Remodeling)

MAPK Family Pathway (Cytoskeleton)

RAS Main Pathway

TNF (Positive) Main Pathway

106 common (shared) activated Signaling PW (16h post OP):

AKT Main Pathway

AKT Pathway (Aggregation & Neurodegeneration)

AKT Pathway (Apoptosis Inhibition)

AKT Pathway (Cardiovascular Homeostasis)

AKT Pathway (Caspase Cascade)

AKT Pathway (Cell Cycle)

AKT Pathway (Death Genes)

AKT Pathway (Elevation of Glucose Import)

AKT Pathway (Genetic Stability)

AKT Pathway (Glucose Uptake)

AKT Pathway (Insulin Stimulated Mitogenesis)

AKT Pathway (JNK Pathway)

AKT Pathway (Neuroprotection)

AKT Pathway (NF-kB Pathway)

AKT Pathway (Respiratory Burst)

AKT Pathway (Synaptic Signaling)

Androgen Receptor Pathway

Androgen Receptor Pathway (Apoptosis)

ATM Pathway (G2_M Checkpoint Arrest)

DDR Pathway (BRCA1-induced responses)

cAMP Main Pathway

cAMP Pathway (Endothelial Cell Regulation)

cAMP Pathway (Metabolic Energy)

Cellular Anti Apoptosis Main Pathway

Cellular Anti Apoptosis Pathway (Apoptosis)

CREB Main Pathway

CREB Pathway (Gene Expression Pathway)

ErbB Family Main Pathway

ERK Signaling Main Pathway

Estrogen Main Pathway

Glucocorticoid Receptor Main Pathway

Glucocorticoid Receptor Pathway (Cell cycle arrest)

Glucocorticoid Receptor Pathway (Cell cycle progression)

Glucocorticoid Receptor Pathway (Gene expression)

GPCR Main Pathway

Growth Hormone Main Pathway

Growth Hormone Pathway (Gene expression)

Growth Hormone Pathway (Glucose uptake)

GSK3 Main Pathway

GSK3 Pathway (Degradation)

GSK3 Pathway (Gene expression)

G-protein Pathway (Ras family GTPases)

Hedgehog Pathway (Repression of Hh, BMP)

HGF Main Pathway

HGF Pathway (Anoikis)

HGF Pathway (Cell adhesion, cell mirgation)

HGF Pathway (Cell cycle progression)

HGF Pathway (Cell scattering)

HGF Pathway (Cell survival)

HGF Pathway (IP3 pathway)

HIF1Alpha Pathway (Gene expression)

HIF1Alpha Pathway (NOS pathway)

HIF1Alpha Pathway (Pyruvate)

Hypoxia pathway EMT 1

Hypoxia pathway EMT 2

Hypoxia pathway EMT 3

Hypoxia pathway EMT 4

ILK Main Pathway

ILK Pathway (Apoptosis)

ILK Pathway (Cell adhesion, cell motility, opsonization)

ILK Pathway (Cell cycle proliferation)

ILK Pathway (Cell migration, retraction)

ILK Pathway (Cell motility)

ILK Pathway (Cytoskeletal reorganization)

ILK Pathway (G2-phase arrest)

ILK Pathway (Induced cell proliferation)

ILK Pathway (Regulation of intermediate filaments)

ILK Pathway (Regulation of junction assembly of desmosomes)

ILK Pathway (Wound healing)

IL-10 Pathway (Gene expression)

IL-10 Pathway (Stability determination)

IL-2 Pathway (Apoptosis)

IL-2 Pathway (Apoptosis inhibition)

IL-6 Main Pathway

Integrin SIgnaling Main Pathway

Interactions Report

Interferon Main Pathway

JNK Main Pathway

JNK Pathway (Apoptosis, Inflammation, Tumorigenesis, Cell Migration)

MAPK Family Main Pathway

MAPK Signaling Main Pathway

MAPK Signaling Pathway (Cell Survival, Inflammation, Apoptosis, Osmoregulation)

MAPK Signaling Pathway (Gene Expression)

mTOR Main Pathway

NGF (Positive) Main Pathway

p38 (Negative) Main Signaling Pathway

p38 (Positive) Main Signaling Pathway

p53 Signaling (Negative) Main Pathway

PAK Main Pathway

PAK Pathway (Actin Cytoskeleton)

PAK Pathway (Myosin Activation)

PPAR Main Pathway

RANK Signaling in Osteoclast Main Pathway

SMAD (Negative) Main Pathway

SMAD (Negative) Pathway (Degradation)

SMAD (Positive) Main Pathway

SMAD (Positive) Pathway (Degradation)

Cell Cycle Pathway (Metaphase-Anaphase)

STAT3 Main Pathway

TGF beta Main Pathway

TGF beta Pathway (Epithelial mesehchymal transition)

TGF beta Pathway (Post-transcriptional G1 arrest)

Cell Cycle Pathway (End of S-phase)

VEGF Main Pathway

VEGF Pathway (Actin Reorganization)

Wnt Main Pathway

2) T=16h; silenced signaling pathways (p<0.05)

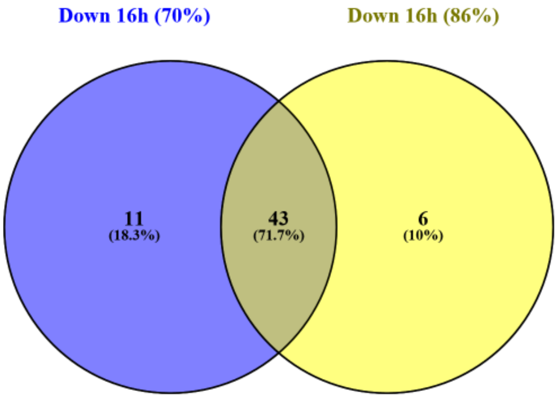

11 Unique silenced Signaling PW after 68% Hx (16h post OP):

cAMP Pathway (Cell Survival)

Caspase Cascade (Activated Tissue Transglutaminase)

CD40 Main Pathway

Cellular Anti Apoptosis Main Pathway

Cellular Anti Apoptosis Pathway (Apoptosis)

Chemokine Main Pathway

GPCR Pathway (Gene expression)

IGF1R Main Pathway

IL-10 Pathway (Translational modulation)

IP3 Main Pathway

TRAF (Positive) Main Pathway

6 Unique silenced Signaling PW after 86% Hx (16h post OP):

DNA Repair Mechanisms Pathway

EGFR Main Pathway

Fas Signaling Pathway (Negative)

Integrin SIgnaling Pathway (Cell survival)

JNK Pathway (Insulin signaling)

Cell Cycle Pathway (Origin of S-phase)

43 Common downregulated Signaling PW 16h post OP):

AKT Pathway (Cell Cycle Progression)

Androgen Receptor Pathway (Degradation)

Androgen Receptor Pathway (Cell Survival & Cell Growth)

Androgen Receptor Pathway (Gonadotropin Regulation)

Androgen Receptor Pathway (Histone Modification)

Androgen Receptor Pathway (Prostate Differentiation & Development)

Androgen Receptor Pathway (Sexual Differentiation & Sexual Maturation at Puberty)

BRCA1 Main Pathway

cAMP Pathway (Axonal Growth)

cAMP Pathway (Cardiovascular Homeostasis)

cAMP Pathway (Cell Proliferation)

cAMP Pathway (Degradation of Cell Cycle Regulators)

cAMP Pathway (Glycogen Synthesis)

cAMP Pathway (Glycolysis)

cAMP Pathway (Myocardial Contraction)

cAMP Pathway (Oncogenesis)

cAMP Pathway (Protein Retention)

cAMP Pathway (Regulation of Cytoskeleton)

Caspase Cascade Main

Caspase Cascade (Apoptosis)

Caspase Cascade (ICAD Degradation)

Cytokine Main Pathway

Erythropoeitin Main Pathway

Hedgehog Main Pathway

HIF1-Alpha Main Pathway

IL-2 Main Pathway

Integrin SIgnaling Pathway (Cytoskeleton contraction integrin modulation cell invasion and migration)

JAK mStat Main Pathway

JAK mStat Pathway (JAK degradation)

MAPK Family Pathway (Gene Expression)

Mitochondrial Apopotosis Main Pathway

Mitochondrial Apopotosis Pathway (Apoptosis)

mTOR Pathway (Actin organization)

Notch Main Pathway

p53 Signaling (Negative) Pathway (p53 Degradation)

PTEN Main Pathway

Telomere Main Pathway

RNA Polymerase II Complex Pathway

TNF (Negative) Main Pathway

TNF (Negative) Pathway (Apoptosis)

Transcription of mRNA Pathway

Translation Regulation of EIF4F activity

Wnt Pathway (Ctnn-b Degradation)

Borger et al
Supplemental Data

D: Unique and shared Signaling Pathways after 68% and 86% hepatectomy (t=32h post OP)

1) T=32h; activated signaling pathways (p<0.05)

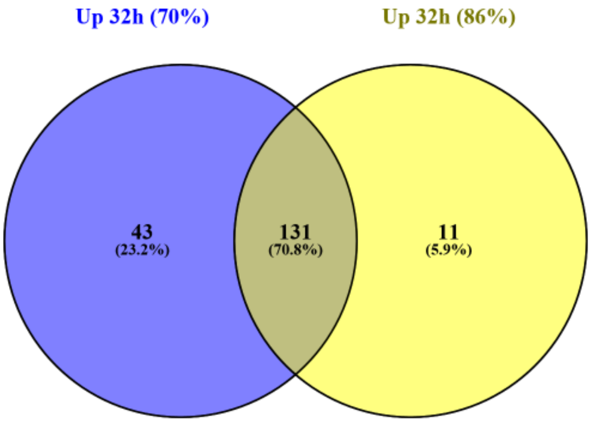

43 Unique activated Signaling PW after 68% Hx (32h post OP):

AHR Main Pathway

AKT Pathway (Blocks Apoptosis)

AKT Pathway (Caspase Cascade)

AKT Pathway (Cell Cycle)

AKT Pathway (Cell Survival)

AKT Pathway (Death Genes)

AKT Pathway (Elevation of Glucose Import)

AKT Pathway (ERK Pathway)

AKT Pathway (Glycogen Synthesis)

AKT Pathway (p53 Degradation)

AKT Pathway (p73 Mediated Apoptosis)

AKT Pathway (Regeneration of Cyclic Nucleotide)

AKT Pathway (Survival Genes)

AKT Pathway (Synaptic Signaling)

Androgen Receptor Pathway (Gonadotropin Regulation)

Androgen Receptor Pathway (Histone Modification)

Androgen Receptor Pathway (Prostate Differentiation & Development)

Androgen Receptor Pathway (Sexual Differentiation & Sexual Maturation at Puberty)

BRCA1 Main Pathway

cAMP Pathway (Cell Survival)

cAMP Pathway (Metabolic Energy)

cAMP Pathway (Protein Retention)

Caspase Cascade (ICAD Degradation)

Chromatin Main Pathway

Chromatin Pathway (Octamer Sliding)

Chromatin Pathway (Octamer Transfer)

DDR pathway Apoptosis

DDR Main pathway

Erythropoeitin Main Pathway

G-protein Pathway (Ras family GTPases)

Hedgehog Main Pathway

IGF1R Signaling Pathway (Cell survival)

IGF1R Signaling Pathway (Glucose uptake)

ILK Pathway (Induced cell proliferation)

IL-2 Main Pathway

MAPK Signaling Pathway (Cell Survival, Inflammation, Apoptosis, Osmoregulation)

DDR pathway (MMR)

Mismatch Repair Main Pathway

Notch Main Pathway

DDR Pathway (NER)

Telomere Main Pathway

Cell Cycle Pathway (End of S-phase)

Ubiquitin Proteasome Pathway (Degraded Protein)

11 Unique activated Signaling PW after 86% Hx (32h post OP):

Androgen Receptor Pathway (Cell Survival & Cell Growth)

ATM Pathway (G2_M Checkpoint Arrest)

DDR Pathway (BRCA1-induced responses)

cAMP Pathway (Cytokine Production)

CREB Pathway (Gene Expression Pathway)

IL-10 Pathway (Translational modulation)

Interferon Main Pathway

Interferon Pathway (Gene expression)

MAPK Family Pathway (Chromatin Remodeling)

MAPK Family Pathway (Cytoskeleton)

p53 Signaling (Negative) Main Pathway

131 Common activated Signaling PW (32h post OP):

AKT Main Pathway

AKT Pathway (Aggregation & Neurodegeneration)

AKT Pathway (Apoptosis Inhibition)

AKT Pathway (Cardiovascular Homeostasis)

AKT Pathway (Glucose Uptake)

AKT Pathway (Insulin Stimulated Mitogenesis)

AKT Pathway (Neuroprotection)

AKT Pathway (NF-kB Pathway)

AKT Pathway (Respiratory Burst)

Androgen Receptor Pathway

Androgen Receptor Pathway (Apoptosis)

Androgen Receptor Pathway (Degradation)

ATM Main Pathway

ATM Pathway (Apoptosis, Senescense)

ATM Pathway (Cell Cicle Checkpoint Control)

ATM Pathway (Cell Survival)

ATM Pathway (Checkpoint Activation)

ATM Pathway (DNA Repair)

ATM Pathway (NF-kB Pathway)

ATM Pathway (Synaptic Vesicle Transport)

ATM Pathway (S-Phase Arrest)

cAMP Main Pathway

cAMP Pathway (Endothelial Cell Regulation)

cAMP Pathway (Myocardial Contraction)

Caspase Cascade (Activated Tissue Transglutaminase)

CD40 Main Pathway

CD40 Pathway (Gene Expression)

CD40 Pathway (IKBs Degradation)

Cellular Anti Apoptosis Main Pathway

Cellular Anti Apoptosis Pathway (Apoptosis)

Chemokine Main Pathway

Chemokine Pathway (Cell Activation)

Chemokine Pathway (Gene Expression, Apoptosis)

CREB Main Pathway

DNA Repair Mechanisms Pathway

EGFR Main Pathway

ErbB Family Main Pathway

ERK Signaling Main Pathway

Estrogen Main Pathway

Fas Signaling Pathway (Negative)

FLT3 Main Pathway

Glucocorticoid Receptor Main Pathway

Glucocorticoid Receptor Pathway (Cell cycle arrest)

Glucocorticoid Receptor Pathway (Gene expression)

Glucocorticoid Receptor Pathway (Inflammatory cytokines)

Glucocorticoid Receptor Pathway (SMAD signaling)

GPCR Main Pathway

GPCR Pathway (Gene expression)

GSK3 Main Pathway

Hedgehog Pathway (Repression of Hh, BMP)

HGF Main Pathway

HGF Pathway (Anoikis)

HGF Pathway (Cell adhesion, cell mirgation)

HGF Pathway (Cell cycle progression)

HGF Pathway (Cell scattering)

HGF Pathway (Cell survival)

HGF Pathway (IP3 pathway)

Hypoxia pathway EMT 1

Hypoxia pathway EMT 2

Hypoxia pathway EMT 3

Hypoxia pathway EMT 4

IGF1R Main Pathway

IGF1R Signaling Pathway (IKB degradation)

ILK Main Pathway

ILK Pathway (Apoptosis)

ILK Pathway (Cell adhesion, cell motility, opsonization)

ILK Pathway (Cell cycle proliferation)

ILK Pathway (Cell migration, retraction)

ILK Pathway (Cell motility)

ILK Pathway (Cytoskeletal reorganization)

ILK Pathway (G2-phase arrest)

ILK Pathway (Regulation of intermediate filaments)

ILK Pathway (Regulation of junction assembly of desmosomes)

ILK Pathway (Wound healing)

IL-10 Main Pathway

IL-10 Pathway (Gene expression)

IL-2 Pathway (Actin reorganization)

IL-6 Main Pathway

Integrin SIgnaling Main Pathway

Integrin SIgnaling Pathway (Cell survival)

Integrin SIgnaling Pathway (Translocation to the nucleus)

Interactions Report

IP3 Main Pathway

JAK mStat Main Pathway

JAK mStat Pathway (Akt pathway)

JNK Main Pathway

JNK Pathway (Apoptosis, Inflammation, Tumorigenesis, Cell Migration)

MAPK Family Main Pathway

MAPK Family Pathway (Gene Expression)

MAPK Family Pathway (IKBs Degradation)

MAPK Signaling Main Pathway

MAPK Signaling Pathway (Gene Expression)

Mitochondrial Apopotosis Pathway (Apoptosis)

mTOR Main Pathway

mTOR Pathway (Actin organization)

NGF (Positive) Main Pathway

NHEJ mechanisms of DSBs repair effect

p38 (Negative) Main Signaling Pathway

p38 (Positive) Main Signaling Pathway

p53 Signaling (Negative) Pathway (p53 Degradation)

PAK Main Pathway

PAK Pathway (Actin Cytoskeleton)

PAK Pathway (Myosin Activation)

PPAR Main Pathway

RANK Signaling in Osteoclast Main Pathway

RANK Signaling in Osteoclast Pathway (IKBs Degradation)

RAS Main Pathway

RNA Polymerase II Complex Pathway

SMAD (Negative) Main Pathway

SMAD (Positive) Main Pathway

Cell Cycle Pathway (Metaphase-Anaphase)

Cell Cycle Pathway (Origin of S-phase)

STAT3 Main Pathway

TGF beta Main Pathway

TGF beta Pathway (Epithelial mesehchymal transition)

TGF beta Pathway (Post-transcriptional G1 arrest)

TNF (Negative) Main Pathway

TNF (Negative) Pathway (Apoptosis)

TNF (Positive) Main Pathway

TNF (Positive) Pathway (Gene expression, Cell survival)

TNF (Positive) Pathway (IKBs degradation)

TRAF (Negative) Pathway (IKBs Degradation)

TRAF (Positive) Main Pathway

TRAF (Positive) Pathway (IKBs Degradation)

Transcription of mRNA Pathway

Translation Regulation of EIF4F activity

Ubiquitin Proteasome Main Pathway

VEGF Main Pathway

VEGF Pathway (Actin Reorganization)

Wnt Main Pathway

IL-6 Pathway (IKBs degradation)

2) T=32h; silenced signaling pathways (p<0.05)

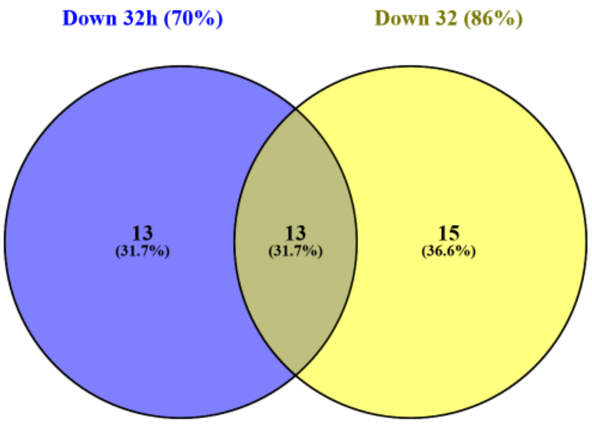

13 Unique silenced Signaling PW after 68% Hx (32h post OP):

AKT Pathway (Genetic Stability)

ATM Pathway (G2_M Checkpoint Arrest)

ATM Pathway (G2 Mitosis Progression)

ATM Pathway (MDMX Ubiquitination, Degradation)

ATM Pathway (S-Phase Progression)

cAMP Pathway (Cell Growth)

cAMP Pathway (Cell Survival, Chemotaxis)

cAMP Pathway (Cytokine Production)

Caspase Cascade (Apoptosis)

GSK3 Pathway (Gene expression)

Mitochondrial Apopotosis Pathway (Gene expression)

p53 Signaling (Negative) Main Pathway

Cell Cycle Pathway (SCC during S-phase)

15 Unique silenced Signaling PW after 86% Hx (32h post OP):

Androgen Receptor Pathway (Gonadotropin Regulation)

Androgen Receptor Pathway (Histone Modification)

Androgen Receptor Pathway (Prostate Differentiation & Development)

Androgen Receptor Pathway (Sexual Differentiation & Sexual Maturation at Puberty)

BRCA1 Main Pathway

Growth Hormone Main Pathway

Growth Hormone Pathway (Gene expression)

Growth Hormone Pathway (Glucose uptake)

Hedgehog Main Pathway

IGF1R Signaling Pathway (Cell survival)

ILK Pathway (Induced cell proliferation)

MAPK Signaling Pathway (Cell Survival, Inflammation, Apoptosis, Osmoregulation)

Notch Main Pathway

Telomere Main Pathway

Ubiquitin Proteasome Pathway (Degraded Protein)

13 Common silenced Signaling PW (32h post OP):

AKT Pathway (Cell Cycle Progression)

cAMP Pathway (Axonal Growth)

cAMP Pathway (Cardiovascular Homeostasis)

cAMP Pathway (Cell Proliferation)

cAMP Pathway (Degradation of Cell Cycle Regulators)

cAMP Pathway (Glycogen Synthesis)

cAMP Pathway (Oncogenesis)

cAMP Pathway (Regulation of Cytoskeleton)

Caspase Cascade Main

Caspase Cascade (Cell Survival)

Mitochondrial Apoptosis Main Pathway

Mitochondrial Apoptosis Pathway (DNA fragmentation)

PTEN Main Pathway

E: Unique and shared Signaling Pathways after 68% and 86% hepatectomy (t=48h post OP)

1) T=48h; activated signaling pathways (p<0.05)

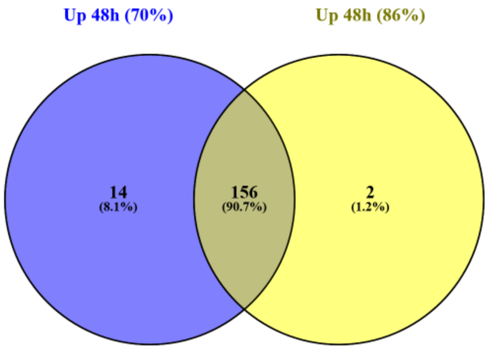

14 Unique activated Signaling PW after 68% Hx (48h post OP):

CD40 Pathway (Gene Expression)

Erythropoeitin Main Pathway

GPCR Pathway (Gene expression)

Growth Hormone Pathway (Cell survival)

Growth Hormone Pathway (Protein synthesis)

IGF1R Main Pathway

IGF1R Signaling Pathway (IKB degradation)

IL-10 Pathway (Gene expression)

MAPK Family Pathway (IKBs Degradation)

RANK Signaling in Osteoclast Pathway (IKBs Degradation)

TNF (Positive) Pathway (IKBs degradation)

TRAF (Negative) Pathway (IKBs Degradation)

TRAF (Positive) Pathway (IKBs Degradation)

IL-6 Pathway (IKBs degradation)

2 Unique activated Signaling PW after 86% Hx (48h post OP):

ATM Main Pathway

CREB Pathway (Gene Expression Pathway)

156 Common activated Signaling PW (48h post OP):

AHR Main Pathway

AKT Main Pathway

AKT Pathway (Aggregation & Neurodegeneration)

AKT Pathway (Apoptosis Inhibition)

AKT Pathway (Cardiovascular Homeostasis)

AKT Pathway (Caspase Cascade)

AKT Pathway (Cell Cycle)

AKT Pathway (Death Genes)

AKT Pathway (Elevation of Glucose Import)

AKT Pathway (Glucose Uptake)

AKT Pathway (Insulin Stimulated Mitogenesis)

AKT Pathway (JNK Pathway)

AKT Pathway (Neuroprotection)

AKT Pathway (NF-kB Pathway)

AKT Pathway (p73 Mediated Apoptosis)

AKT Pathway (Regeneration of Cyclic Nucleotide)

AKT Pathway (Respiratory Burst)

AKT Pathway (Synaptic Signaling)

Androgen Receptor Pathway

Androgen Receptor Pathway (Apoptosis)

Androgen Receptor Pathway (Cell Survival & Cell Growth)

Androgen Receptor Pathway (Gonadotropin Regulation)

Androgen Receptor Pathway (Histone Modification)

Androgen Receptor Pathway (Prostate Differentiation & Development)

Androgen Receptor Pathway (Sexual Differentiation & Sexual Maturation at Puberty)

ATM Pathway (Cell Cicle Checkpoint Control)

ATM Pathway (Cell Survival)

ATM Pathway (Checkpoint Activation)

ATM Pathway (DNA Repair)

ATM Pathway (NF-kB Pathway)

ATM Pathway (Synaptic Vesicle Transport)

ATM Pathway (S-Phase Arrest)

BRCA1 Main Pathway

cAMP Main Pathway

cAMP Pathway (Cell Survival)

cAMP Pathway (Endothelial Cell Regulation)

cAMP Pathway (Glycolysis)

cAMP Pathway (Metabolic Energy)

cAMP Pathway (Protein Retention)

CD40 Main Pathway

Cellular Anti Apoptosis Main Pathway

Cellular Anti Apoptosis Pathway (Apoptosis)

Chemokine Main Pathway

Chromatin Main Pathway

Chromatin Pathway (Octamer Sliding)

Chromatin Pathway (Octamer Transfer)

Circadian Main Pathway

CREB Main Pathway

DDR pathway Apoptosis

DDR Main pathway

DNA Repair Mechanisms Pathway

EGFR Main Pathway

ErbB Family Main Pathway

ERK Signaling Main Pathway

Estrogen Main Pathway

Fas Signaling Pathway (Negative)

FLT3 Main Pathway

Glucocorticoid Receptor Main Pathway

Glucocorticoid Receptor Pathway (Cell cycle arrest)

Glucocorticoid Receptor Pathway (Gene expression)

Glucocorticoid Receptor Pathway (Inflammatory cytokines)

GPCR Main Pathway

GSK3 Main Pathway

GSK3 Pathway (Gene expression)

G-protein Pathway (Ras family GTPases)

Hedgehog Main Pathway

Hedgehog Pathway (Repression of Hh, BMP)

HGF Main Pathway

HGF Pathway (Anoikis)

HGF Pathway (Cell adhesion, cell mirgation)

HGF Pathway (Cell cycle progression)

HGF Pathway (Cell scattering)

HGF Pathway (Cell survival)

HGF Pathway (IP3 pathway)

HIF1-Alpha Main Pathway

HIF1Alpha Pathway (Gene expression)

HIF1Alpha Pathway (NOS pathway)

HIF1Alpha Pathway (Pyruvate)

HIF1Alpha Pathway (VEGF pathway)

Hypoxia pathway EMT 1

Hypoxia pathway EMT 2

Hypoxia pathway EMT 3

Hypoxia pathway EMT 4

IGF1R Signaling Pathway (Cell survival)

ILK Main Pathway

ILK Pathway (Apoptosis)

ILK Pathway (Cell adhesion, cell motility, opsonization)

ILK Pathway (Cell cycle proliferation)

ILK Pathway (Cell migration, retraction)

ILK Pathway (Cell motility)

ILK Pathway (Cytoskeletal reorganization)

ILK Pathway (G2-phase arrest)

ILK Pathway (Induced cell proliferation)

ILK Pathway (Regulation of intermediate filaments)

ILK Pathway (Regulation of junction assembly of desmosomes)

ILK Pathway (Wound healing)

IL-10 Main Pathway

IL-2 Pathway (Actin reorganization)

IL-2 Pathway (Apoptosis)

IL-2 Pathway (Apoptosis inhibition)

IL-6 Main Pathway

Integrin SIgnaling Main Pathway

Integrin SIgnaling Pathway (Cell survival)

Integrin SIgnaling Pathway (Focal adhesion and stress fibers)

Integrin SIgnaling Pathway (Translocation to the nucleus)

Interactions Report

Interferon Main Pathway

IP3 Main Pathway

IP3 Pathway (Gene expression)

JAK mStat Main Pathway

JAK mStat Pathway (Akt pathway)

JNK Main Pathway

JNK Pathway (Apoptosis, Inflammation, Tumorigenesis, Cell Migration)

MAPK Family Main Pathway

MAPK Signaling Main Pathway

MAPK Signaling Pathway (Cell Survival, Inflammation, Apoptosis, Osmoregulation)

MAPK Signaling Pathway (Gene Expression)

DDR pathway (MMR)

Mismatch Repair Main Pathway

Mitochondrial Apopotosis Pathway (Apoptosis)

mTOR Main Pathway

NGF (Positive) Main Pathway

NHEJ mechanisms of DSBs repair effect

Notch Main Pathway

DDR Pathway (NER)

p38 (Negative) Main Signaling Pathway

p38 (Positive) Main Signaling Pathway

p53 Signaling (Negative) Pathway (p53 Degradation)

PAK Main Pathway

PAK Pathway (Actin Cytoskeleton)

PAK Pathway (Myosin Activation)

PPAR Main Pathway

RANK Signaling in Osteoclast Main Pathway

RAS Main Pathway

Telomere Main Pathway

RNA Polymerase II Complex Pathway

SMAD (Negative) Main Pathway

SMAD (Negative) Pathway (Degradation)

SMAD (Positive) Main Pathway

SMAD (Positive) Pathway (Degradation)

Cell Cycle Pathway (Metaphase-Anaphase)

Cell Cycle Pathway (Origin of S-phase)

STAT3 Main Pathway

TGF beta Main Pathway

TGF beta Pathway (Epithelial mesehchymal transition)

TGF beta Pathway (Post-transcriptional G1 arrest)

TNF (Negative) Main Pathway

TNF (Negative) Pathway (Apoptosis)

TNF (Positive) Main Pathway

TNF (Positive) Pathway (Gene expression, Cell survival)

Transcription of mRNA Pathway

Ubiquitin Proteasome Main Pathway

Ubiquitin Proteasome Pathway (Degraded Protein)

VEGF Main Pathway

VEGF Pathway (Actin Reorganization)

Wnt Main Pathway

2) T=48h; silenced signaling pathways ($p<0.05$)

Borger et al
Supplemental Data

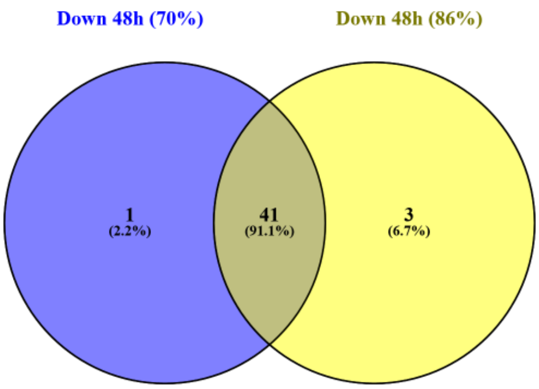

1 Unique silenced Signaling PW after 68% Hx (48h post OP):

CD40 Pathway (IKBs Degradation)

3 Unique silenced Signaling PW after 86% Hx (48h post OP):

cAMP Pathway (Cell Survival)

GPCR Pathway (Gene expression)

IL-2 Main Pathway

41 common downregulated Signaling PW (48h post OP):

AKT Pathway (Cell Cycle Progression)

AKT Pathway (Genetic Stability)

Androgen Receptor Pathway (Degradation)

ATM Pathway (Apoptosis, Senescense)

ATM Pathway (G2_M Checkpoint Arrest)

ATM Pathway (G2 Mitosis Progression)

ATM Pathway (MDMX Ubiquitination, Degradation)

ATM Pathway (S-Phase Progression)

DDR Pathway (BRCA1-induced responses)

cAMP Pathway (Axonal Growth)

cAMP Pathway (Cardiovascular Homeostasis)

cAMP Pathway (Cell Growth)

cAMP Pathway (Cell Proliferation)

cAMP Pathway (Cell Survival, Chemotaxis)

cAMP Pathway (Cytokine Production)

cAMP Pathway (Degradation of Cell Cycle Regulators)

cAMP Pathway (Glycogen Synthesis)

cAMP Pathway (Myocardial Contraction)

cAMP Pathway (Oncogenesis)

cAMP Pathway (Regulation of Cytoskeleton)

Caspase Cascade Main

Caspase Cascade (Apoptosis)

Caspase Cascade (Cell Survival)

Chemokine Pathway (Cell Activation)

Chemokine Pathway (Gene Expression, Apoptosis)

Chemokine Pathway (Internalization, Degradation, Recycling)

Growth Hormone Main Pathway

Growth Hormone Pathway (Gene expression)

Growth Hormone Pathway (Glucose uptake)

Integrin SIgnaling Pathway (Cytoskeleton contraction integrin modulation cell invasion and migration)

JAK mStat Pathway (JAK degradation)

MAPK Family Pathway (Chromatin Remodeling)

MAPK Family Pathway (Cytoskeleton)

MAPK Family Pathway (Gene Expression)

Mitochondrial Apopotosis Main Pathway

Mitochondrial Apopotosis Pathway (DNA fragmentation)

Mitochondrial Apopotosis Pathway (Gene expression)

PTEN Main Pathway

Cell Cycle Pathway (SCC during S-phase)

TRAF (Negative) Main Pathway

TRAF (Positive) Main Pathway

Borger et al
Supplemental Data

F: Signaling Pathways (total = 271) that are common between time points after 68% or 86% OP (T=8h, 16h, 32h & 48n)

1) Common up / down regulated pathways ($p<0.05$) <u>after 68% Hx</u> (left)

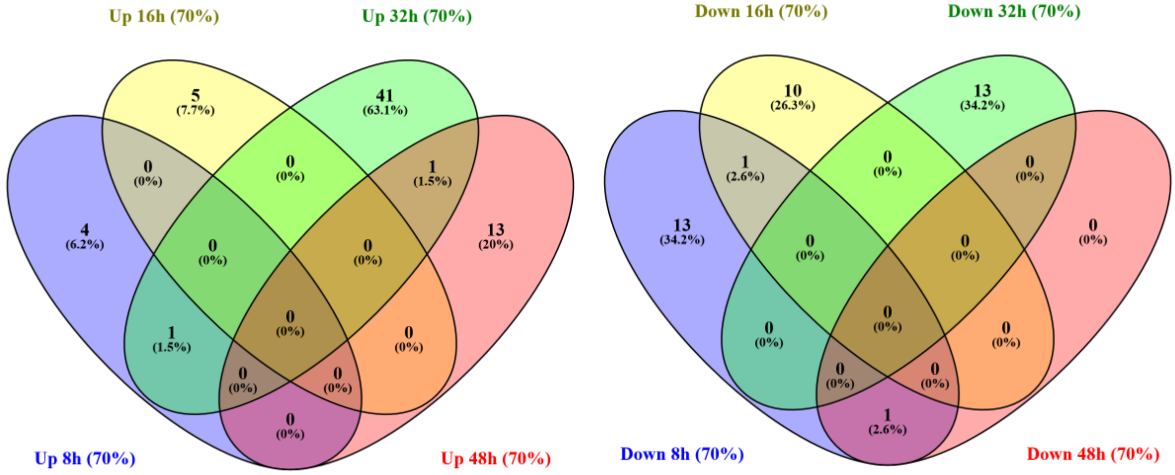

<u>1 Common upregulated Signaling PW between 8h and 32h (68% Hx)</u>

G-protein Pathway (Ras family GTPases)

<u>1 Common upregulated Signaling PW between 32h and 48h (68% Hx)</u>

Erythropoeitin Main Pathway

<u>1 Common downregulated Signaling PW between 8h and 16h (68% Hx)</u>

Caspase Cascade (Activated Tissue Transglutaminase)

2) Common up / down regulated pathways (p<0.05) after 86% Hx (left)

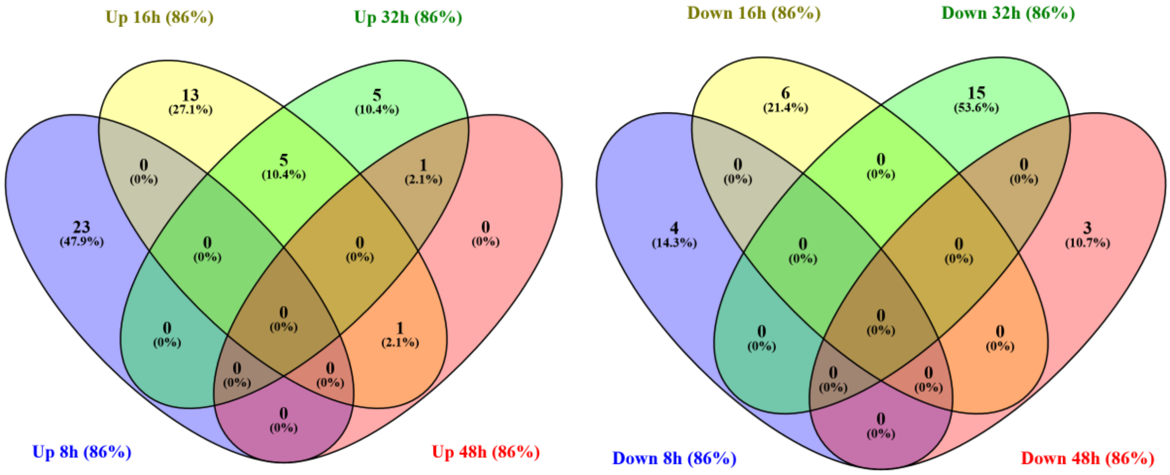

5 Common upregulated Signaling PW between 16h and 32h (86% Hx)

cAMP Pathway (Cytokine Production)

IL-10 Pathway (Translational modulation)

Interferon Pathway (Gene expression)

MAPK Family Pathway (Chromatin Remodeling)

MAPK Family Pathway (Cytoskeleton)

1 Common upregulated Signaling PW between 32h and 48h (86% Hx)

CREB Pathway (Gene Expression Pathway)

1 Common upregulated Signaling PW between 16h and 48h (86% Hx)

ATM Main Pathway

0 Common downregulated Signaling PW (86% Hx)

Activated ISPs

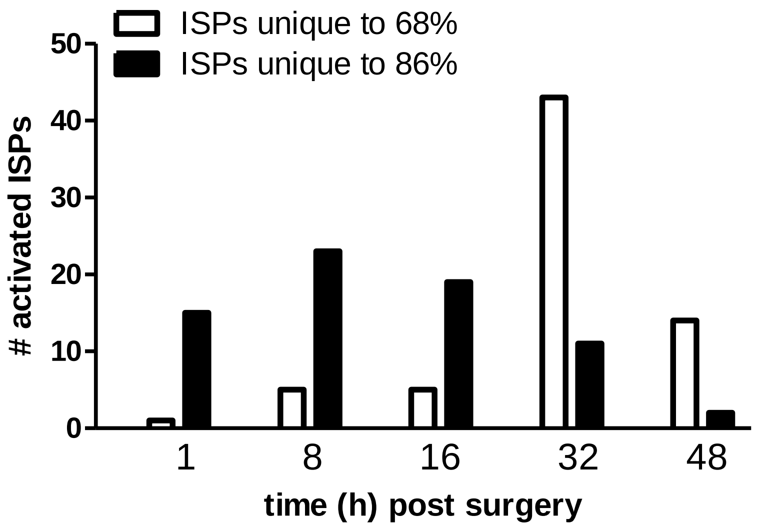

Silenced ISPs

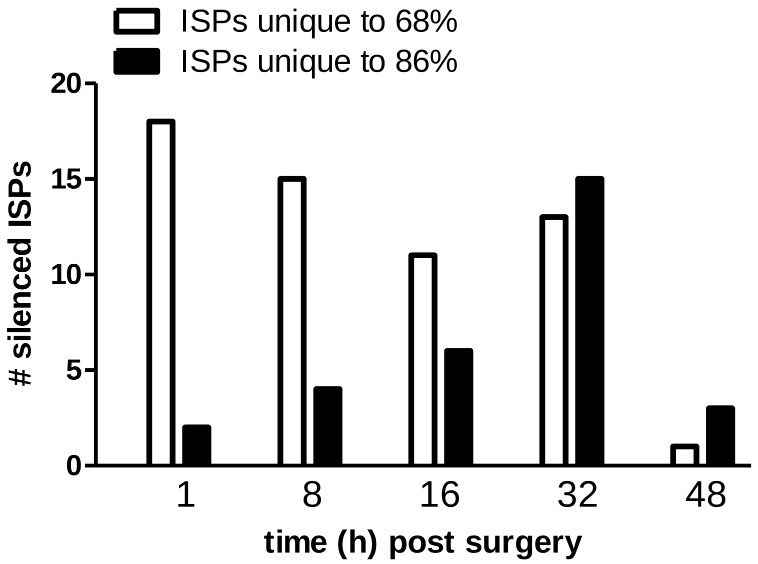

G: Inverse regulated signaling PW after 68% versus 86% hepatectomy (compare and find up- and downregulated between the two surgical procedures at all time point)

1) Inverse regulated signaling PWs ($p<0.05$) at t=1h

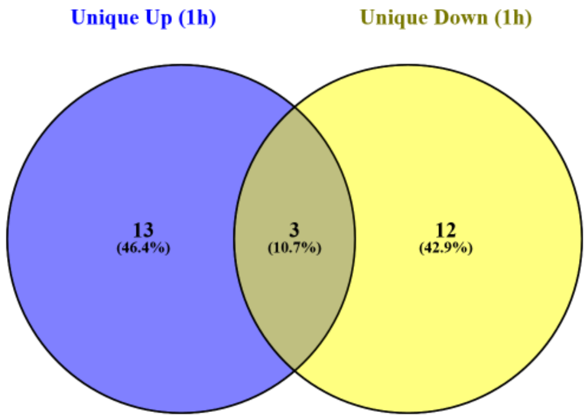

3 inverse regulated pathways:

Signaling pathway	PAS (68%) (1h post OP)	PAS (86%) (1h post OP)
ATM Main Pathway	-0.026931755	0.049054599
ATM Pathway (G2_M Checkpoint Arrest)	-0.115072046	0.053485971
Circadian Main Pathway	0.119250412	-0.06503196

2) Inverse regulated signaling PWs ($p<0.05$) at t=8h

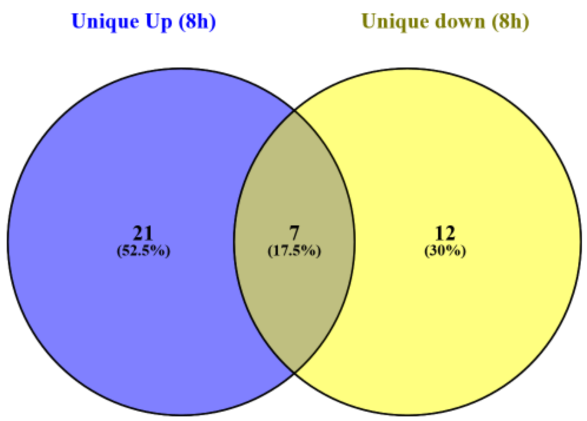

7 inverse regulated pathways:

Signaling pathway	PAS (68%) (8h post OP)	PAS (86%) (8h post OP)
CD40 Pathway (Gene Expression)	-0.106856601	0.026586434
CD40 Pathway (IKBs Degradation)	-0.075428189	0.018766894
Cytokine Main Pathway	-0.022302642	0.110941123
ErbB Family Main Pathway	-0.022836959	0.124348982
MAPK Family Main Pathway	-0.00622822	0.055983961
Mitochondrial Apoptosis Pathway (Apoptosis)	-0.064965944	0.181980954
TNF (Negative) Pathway (Apoptosis)	-0.050757976	0.047876501

3) Inverse regulated signaling PWs ($p<0.05$) at t=16h

Borger et al
Supplemental Data

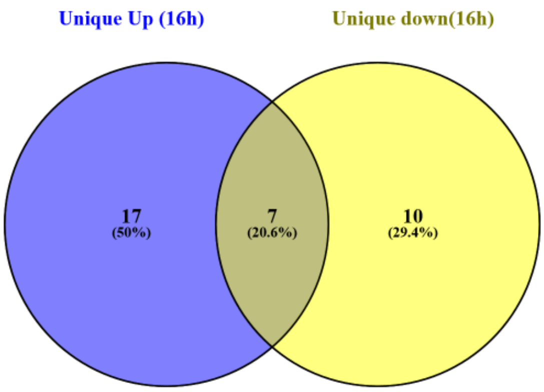

7 inverse regulated pathways:

Signaling pathway	PAS (68%) (16h post OP)	PAS (86%) (16h post OP)
DNA Repair Mechanisms Pathway	0.021261636	-0.006681124
EGFR Main Pathway	0.00199192	-0.008507834
Caspase Cascade (Activated Tissue Transglutaminase)	-0.056797429	0.006476885
Chemokine Main Pathway	-0.005810922	0.014025869
IGF1R Main Pathway	-0.005116309	0.033528677
IL-10 Pathway (Translational modulation)	-0.059638556	0.109013662
IP3 Main Pathway	-0.005184552	0.010535276

4) Inverse regulated signaling PWs ($p<0.05$) at t=32h

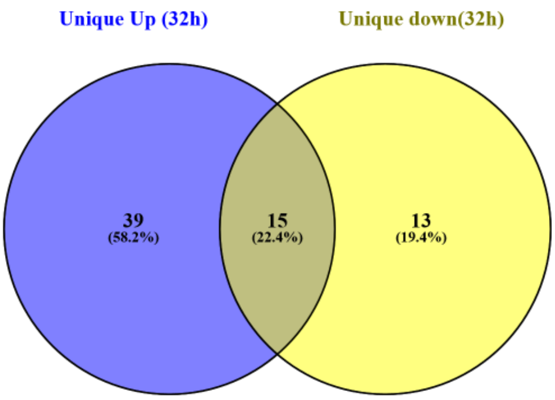

15 inverse regulated pathways:

Signaling Pathway	PAS (68%) (32h post OP)	PAS (86%) (32h post OP)
Androgen Receptor Pathway (Gonadotropin Regulation)	0.040811073	-0.025954953
Androgen Receptor Pathway (Histone Modification)	0.040811073	-0.025954953
Androgen Receptor Pathway (Prostate Differentiation & Development)	0.040811073	-0.025954953
Androgen Receptor Pathway (Sexual Differentiation & Sexual Maturation at Puberty)	0.040811073	-0.025954953
BRCA1 Main Pathway	0.292340978	-0.031905172
Hedgehog Main Pathway	0.261337874	-0.141280757
IGF1R Signaling Pathway (Cell survival)	0.006681752	-0.031908288

ILK Pathway (Induced cell proliferation)	0.130572193	-0.020641491
MAPK Signaling Pathway (Cell Survival, Inflammation, Apoptosis, Osmoregulation)	0.056879984	-0.021609061
Notch Main Pathway	0.02638906	-0.037605957
Telomere Main Pathway	0.037298818	-0.028433917
Ubiquitin Proteasome Pathway (Degraded Protein)	0.112024452	-0.016639519
ATM Pathway (G2_M Checkpoint Arrest)	-0.651298809	0.282117965
cAMP Pathway (Cytokine Production)	-0.088744286	0.140344644
p53 Signaling (Negative) Main Pathway	-0.018160145	0.110310831

5) Inverse regulated signaling PWs (p<0.05) at t=48h

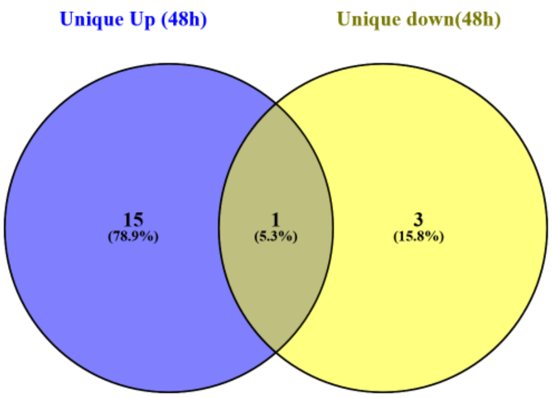

1 inverse regulated pathways:

Signaling Pathway	PAS (68%) (48h post OP)	PAS (86%) (48h post OP)
GPCR Pathway (Gene expression)	0.012145096	-0.002860084

Supplemental Data S-5

List of up and down regulated lncRNAs (70=normal hepatectomy; 86=extended hepatectomy)

		1 h		4h		8h		16h		32h		48h	
	%Hx	70	86	70	86	70	86	70	86	70	86	70	86
	1010001B22Rik						-2.067						-0.982
	1110019D14Rik				-0.548				-0.427				
	1110020A21Rik***						-0.943				-0.505		-0.858
	1700022N22Rik***						-0.825				-1.108		-0.84
	1810019N24Rik					-4.938		-2.978					
	2310001H17Rik				-0.567				-1.018				
	4831440E17Rik***		-1.501						-0.831				-0.395
	4930544I03Rik								-1.04		-0.297		
	4930556M19Rik								-0.868				-0.646
up	4931413I07Rik								2.049		2.135		
NB	4933404O12Rik***				-0.937				-0.886		-0.54		
	9330175E14Rik***		-1.055		-0.669				-0.38				
up	9430037G07Rik					1.481		2.201					
up	9930014A18Rik										0.247		0.466
	A330023F24Rik		-0.712						-0.581				
	A330044P14Rik								-0.721		-0.734		
	A430046D13Rik								-0.626		-0.591		
up	A730036I17Rik								1.688		1.948		
	AI838599								-0.777		-0.736		
	C430002E04Rik						-0.976				-0.238		
	E230001N04Rik		-1.204								-0.145		
	Fendrr						-0.999				-0.908		
	Gm10614								-1.552				-1.141
	Gm10660								-0.557				-0.954
	Gm11033		-0.167						-0.521				
	Gm12480										-0.417		-0.876
	Gm12798								-1.351		-1.687		
	Gm13203										-0.934		-1.083
	Gm14149								-0.363		-0.298		
	Gm14319								0.446		1.408		
up	Gm14827						1.188		0.934				
up	Gm14936***						4.727				3.769		4.421
	Gm15408			-1.629		-1.635							
	Gm15675				-0.393				-0.364				
	Gm15889						-0.959		-0.918				
up	Gm15892				2.864		3.526						
up	Gm16938								0.305		0.453		
	Gm17249		-0.599						-0.105				
	Gm17259						-0.877						-0.629
up	Gm17276						2.984		5.037				
	Gm17634								-1.256		-0.208		
	Gm17690				-1.111		-1.083						
	Gm20546								-0.907		-1.026		
	Gm20649***				-0.016				-0.402		-0.955		
	Gm26578						4.441				1.664		
	Gm26659								-0.970		-0.613		
up	Gm26707								0.544		0.338		
	Gm26797								-0.701		-0.913		
	Gm26876								-0.792		-1.13		
up	Gm26902						2.566				1.377		
	Gm4211						-1.294		-0.79				
	Gm4221						-0.894				-0.79		
up	Gm5544						2.055				0.869		
up	Gm5665									1.866		1.404	
	Gm6297		-0.91						-0.948				
	Gm6313								-0.81		-0.346		
	Gm9725						-2.437				-1.012		

Borger et al
Supplemental Data

Candidates lncRNA expression (log2(FC)(=treated/sham))

Inclusion Criteria: ≥2 lncRNA/procedure; same sign per procedure
Exclusion Criteria: lncRNAs were exlcuded when present in both procedures (“non-specific lincRNAs”)

Observations:

1) Using the above mentioned criteria, 57 lincRNAs are significantly up/down regulated

Most abundant lncRNAs in 68% Hx are:*1810019N24Rik; 9430037G07Rik; Gm15408; Gm5665*

Most abundant lncRNAs in 86% Hx are: *1110020A21Rik; 1680022N22Rik; 4831440E17Rik; 4933404O12Rik; 9330175E14Rik; Gm14936; Gm20649.*

2) LincRNAs are predominantly down-regulated compared to sham-procedure.

3) LincRNAs are predominantly involved in 86% Hepatectomy at later time points.

4) *4933404O12Rik* has been reported as one of five lincRNAs relevant in liver regeneration (Huang et al, PLOS July 2015). We only find this one, not the other four.

5) Several lincRNA were identified as pre-miRNAs (see next page)

Borger et al
Supplemental Data

<u>List of miRNA targets pre and post hepatectomy:</u>

sample	lincRNA	miRNA	mirtarbase	sequence
70_1h	-	ENSMUSG00000076036	mmu-miR-692	AUCUCUUUGAGCGCCUCACUC
70_1h	-	ENSMUSG00000098898	mmu-miR-3470a	UCACUUUGUAGACCAGGCUGG
70_1h	-	ENSMUSG00000098898	mmu-miR-3470b	UCACUCUGUAGACCAGGCUGG
86_1h	-	ENSMUSG00000076138	mmu-miR-703	AAAACCUUCAGAAGGAAAGAA
70_4h	-	ENSMUSG00000098973	no	
86_4h	-	ENSMUSG00000076036	mmu-miR-692	AUCUCUUUGAGCGCCUCACUC
86_4h	ENSMUSG00000097277	ENSMUSG00000104618	mmu-miR-1839-5p	AAGGUAGAUAGAACAGGUCUUG
86_4h	ENSMUSG00000097277	ENSMUSG00000104618	mmu-miR-1839-3p	AGACCUACUUAUCUACCAACAGC
70_8h	-	ENSMUSG00000098457	no	
86_8h	-	ENSMUSG00000080666	mmu-miR-466i-3p	AUACACACACACAUACACACUA
86_8h	-	ENSMUSG00000080666	mmu-miR-466i-5p	UGUGUGUGUGUGUGUGUGUG
86_8h	ENSMUSG00000086782	ENSMUSG00000065451	mmu-miR-101a-3p	UACAGUACUGUGAUAACUGAA
86_8h	-	ENSMUSG00000088354	mmu-miR-3470a	UCACUUUGUAGACCAGGCUGG
86_8h	-	ENSMUSG00000088354	mmu-miR-3470b	UCACUCUGUAGACCAGGCUGG
86_8h	-	ENSMUSG00000093119	no	
86_8h	-	ENSMUSG00000093133	no	
86_8h	-	ENSMUSG00000095278	mmu-miR-3470b	UCACUCUGUAGACCAGGCUGG
86_8h	-	ENSMUSG00000095278	mmu-miR-3470a	UCACUUUGUAGACCAGGCUGG
86_8h	ENSMUSG00000089726	ENSMUSG00000076256	mmu-miR-19b-3p	UGUGCAAAUCCAUGCAAAACUGA
86_8h	ENSMUSG00000089726	ENSMUSG00000065403	mmu-miR-18a-5p	UAAGGUGCAUCUAGUGCAGAUAG
86_8h	ENSMUSG00000089726	ENSMUSG00000076062	mmu-miR-92a-3p	UAUUGCACUUGUCCCGGCCUG
86_8h	ENSMUSG00000089726	ENSMUSG00000065508	mmu-miR-17-5p	CAAAGUGCUUACAGUGCAGGUAG
86_8h	ENSMUSG00000089726	ENSMUSG00000065416	mmu-miR-19a-3p	UGUGCAAAUCUAUGCAAAACUGA
86_8h	ENSMUSG00000089726	ENSMUSG00000065442	mmu-miR-20a-5p	UAAAGUGCUUAUAGUGCAGGUAG
86_16h	-	ENSMUSG00000093119	no	
86_16h	-	ENSMUSG00000093133	no	
86_16h	-	ENSMUSG00000096425	mmu-miR-3470b	UCACUCUGUAGACCAGGCUGG
86_16h	-	ENSMUSG00000096425	mmu-miR-3470a	UCACUUUGUAGACCAGGCUGG
86_16h	ENSMUSG00000097017	ENSMUSG00000096667	mmu-miR-1194	GAAUGAGUAACUGCUAGAUCCU
86_16h	ENSMUSG00000097391	ENSMUSG00000065497	mmu-miR-410-3p	AAUAUAACACAGAUGGCCUGU
86_16h	ENSMUSG00000097391	ENSMUSG00000065426	mmu-miR-134-5p	UGUGACUGGUUGACCAGAGGGG
86_16h	ENSMUSG00000097391	ENSMUSG00000070128	mmu-miR-485-5p	AGAGGCUGGCCGUGAUGAAUUC
86_16h	ENSMUSG00000097391	ENSMUSG00000065438	mmu-miR-377-3p	AUCACACAAAGGCAACUUUUGU
86_16h	ENSMUSG00000097501	ENSMUSG00000099245	no	
86_16h	ENSMUSG00000097961	ENSMUSG00000084559	mmu-miR-1906	UGCAGCAGCCUGAGGCAGGGCU
86_16h	ENSMUSG00000097961	ENSMUSG00000084535	mmu-miR-1906	UGCAGCAGCCUGAGGCAGGGCU
86_16h	ENSMUSG00000097961	ENSMUSG00000104193	mmu-miR-1906	UGCAGCAGCCUGAGGCAGGGCU
86_16h	-	ENSMUSG00000098343	no	
86_16h	-	ENSMUSG00000098638	mmu-miR-3470a	UCACUUUGUAGACCAGGCUGG
86_16h	-	ENSMUSG00000098638	mmu-miR-3470b	UCACUCUGUAGACCAGGCUGG
86_16h	-	ENSMUSG00000098898	mmu-miR-3470b	UCACUCUGUAGACCAGGCUGG
86_16h	-	ENSMUSG00000098898	mmu-miR-3470a	UCACUUUGUAGACCAGGCUGG
86_16h	-	ENSMUSG00000098973	no	
70_32h	-	ENSMUSG00000076138	mmu-miR-703	AAAACCUUCAGAAGGAAAGAA
86_32h	ENSMUSG00000021268	ENSMUSG00000084559	mmu-miR-1906	UGCAGCAGCCUGAGGCAGGGCU
86_32h	ENSMUSG00000021268	ENSMUSG00000084535	mmu-miR-1906	UGCAGCAGCCUGAGGCAGGGCU
86_32h	ENSMUSG00000021268	ENSMUSG00000104193	mmu-miR-1906	UGCAGCAGCCUGAGGCAGGGCU
86_32h	ENSMUSG00000021268	ENSMUSG00000076451	mmu-miR-770-5p	AGCACCACGUGUCUGGGCCACG
86_32h	ENSMUSG00000021268	ENSMUSG00000076451	mmu-miR-770-3p	CGUGGGCCUGACGUGGAGCUGG
86_32h	-	ENSMUSG00000076128	mmu-miR-686	AUUGCUUCCCAGACGGUGAAGA
86_32h	-	ENSMUSG00000080666	mmu-miR-466i-5p	UGUGUGUGUGUGUGUGUGUG
86_32h	-	ENSMUSG00000080666	mmu-miR-466i-3p	AUACACACACACAUACACACUA
86_32h	-	ENSMUSG00000093133	no	
86_32h	-	ENSMUSG00000096667	mmu-miR-1194	GAAUGAGUAACUGCUAGAUCCU
86_32h	ENSMUSG00000097017	ENSMUSG00000096667	mmu-miR-1194	GAAUGAGUAACUGCUAGAUCCU
86_32h	ENSMUSG00000097961	ENSMUSG00000065497	mmu-miR-410-3p	AAUAUAACACAGAUGGCCUGU
86_32h	ENSMUSG00000097961	ENSMUSG00000065426	mmu-miR-134-5p	UGUGACUGGUUGACCAGAGGGG
86_32h	ENSMUSG00000097961	ENSMUSG00000070128	mmu-miR-485-5p	AGAGGCUGGCCGUGAUGAAUUC
86_32h	ENSMUSG00000097961	ENSMUSG00000065438	mmu-miR-377-3p	AUCACACAAAGGCAACUUUUGU
86_32h	ENSMUSG00000097431	ENSMUSG00000094702	mmu-miR-3471	UGAGAUCCAACUGUAAGGCAUU
86_32h	ENSMUSG00000097431	ENSMUSG00000093302	mmu-miR-3471	UGAGAUCCAACUGUAAGGCAUU
86_32h	ENSMUSG00000097431	ENSMUSG00000092840	mmu-miR-3471	UGAGAUCCAACUGUAAGGCAUU
86_32h	-	ENSMUSG00000098898	mmu-miR-3470b	UCACUCUGUAGACCAGGCUGG
86_32h	-	ENSMUSG00000098898	mmu-miR-3470a	UCACUUUGUAGACCAGGCUGG
86_32h	-	ENSMUSG00000098973	no	
70_48h	ENSMUSG00000086782	ENSMUSG00000065451	mmu-miR-101a-3p	UACAGUACUGUGAUAACUGAA
86_48h	-	ENSMUSG00000076036	mmu-miR-692	AUCUCUUUGAGCGCCUCACUC
86_48h	-	ENSMUSG00000080666	mmu-miR-466i-3p	AUACACACACACAUACACACUA
86_48h	-	ENSMUSG00000080666	mmu-miR-466i-5p	UGUGUGUGUGUGUGUGUGUG

miRNA targets using mirtarbase:

1. ENSMUSG00000076036

mmu-miR-692

AUCUCUUUGAGCGCCUCACUC

Targets: 2510002D24Rik, 4931406C07Rik, 5730409E04Rik, A830018L16Rik, AI987944, Adam19, Adamts17, Alx4, Anln, Arhgef2, Arid3a, Ash1l, Asic1, BC021785, Bdp1, Bnc2, Cabp5, Calcrl, Car10, Ccnd1, Cd300lf, Cd53, Cdadc1, Cdc27, Clec4a2, Clic4, Crispld1, Cry2, D3Bwg0562e, Dhh, Diap2, Dip2b, Dkk2, Dmrta2, Dsg1a, Dsg2, Elavl3, Elmsan1, Elovl6, Ermap, F9, Fam134b, Fam49a, Fmnl2, Fnip1, Foxp1, Frmd3, Frrs1, Fut4, Gabpb2, Galnt13, Gbp10, Gda, Gdi1, Gm20172, Gm4944, Gm4951, Gpr158, Gpr64, Grpr, Hhex, Higd1a, Hist1h2be, Il21,Lhx5, Lhx9, Lsm11, Lzts3, Magt1, Maoa, Mcmdc2, Mex3c, Mmp14, Mpv17, Ms4a6c, Myo18b, Myo1c, Myrip, Nat14, Nav2, Nhlrc2, Nptx1, Ntm, Pappa, Phactr1, Pirt, Plekha2, Plxnb1, Pou4f1, Ppp6r1, Prkag3, Prkcb, Prlr, Rab11fip3, Rad23a, Rad54l2, Rassf3, Rgs7bp, Satb1, Scfd2, Sco1, Scyl3, Sema3e, Sgms1, Sik3, Slc35a1, Slc7a10, Slc7a13, Slco2a1, Spock2, Steap2, Stx17, Styx, Tbx22, Terf1, Tmem8b, Tnrc6c, Usp22, Zfp689, Zfp71-rs1, Zfp839, Zfp937, Zfp953, Zkscan1

2. ENSMUSG00000098898

mmu-miR-3470a

UCACUUUGUAGACCAGGCUGG

Targets: 2010109I03Rik, A630033H20Rik, Abhd15, Acmsd, Acp2, Ap5m1, Arid4b, BC030476, Btaf1, Calm1, Camsap1, Cand2, Clmn, Crtc1, Csf3r, Cx3cr1, Denr, Dut, Elmod3, Epg5, Erich1, Fam134b, Fam86, Fbxl3, Fcer2a, Fktn, Ftsj1, Gad2, Gla, Gprc5c, Ikbip, Il5ra, Invs, Itih5, Jakmip2, Kctd14, Ky, Lgr5, Limd2, Lrrc14b, Lrrc8e, Lsm11, March1, Med16, Mettl2, Mfap1a, Mob4, Mobp, Mrpl19, Myo1e, Nek7, Npy1r, Nwd1, Pax2, Pof1b, Prex2, Prickle2, Prss43, Ptpn7, Rangap1, Raph1, Rhag, Rint1, Samd4, Scd2, Slc17a8, Slc35g1, Slc7a2, Slx1b, Smyd4, Snx30, Srgn, Tmed5, Tmem170b, Tmem178b, Tmem50a, Tmppe, Trim39, Ttbk2, Ung, Wbp1l, Wdr59, Zfp397, Zfp459, Zfp617, Zfp959, Zfyve27

3. ENSMUSG00000098898

mmu-miR-3470b

UCACUCUGUAGACCAGGCUGG

Targets: 2010012O05Rik, 2510002D24Rik, 4930579G24Rik, 5730455P16Rik, A130010J15Rik, AY074887, Abcd4, Adam28, Afg3l1, Aida, Aoc3, Ap5z1, Arhgef9, Asxl2, Bckdk, Camk4, Car5a, Ccdc78, Cd300a, Cd300lf, Cd8b1, Chek2, Chrnb4, Ciapin1, Clmn, Cog7, Crtc3, Ctsa, Dbt, Dcp2, Ddx55, Denr, Dkc1, Dlgap5, Dnal1, Dtx3l, Eci1, Elf1, F10, Fbxo22, Fsd1l, Fxn, Gabpb2, Gga2, Gimap1, Glrx2, Gm4951, Gnal, Gng2, Hccs, Il11, Inip, Iws1, Kcna4, Krr1, Lhx9, Lmbrd1, Lrrc3, Lypla1, Madd, Mau2, Mettl2, Mob3c, Mpv17, Mtrr, N6amt1, Nav1, Nbl1, Ncl, Ncoa7, Nfatc2ip, Nol10, Opa3, Pik3c2a, Pla2g2d, Pla2g7, Polr3f, Prdm2, Psmb11, Psmd9, Rab27a, Rad51c, Rapgef4, Rnf168, Rp2h, Rpl23, Rpl7l1, Rprd1a, Samd8, Sap18, Sass6, Serpine1, Sfn, Sgpp2, Slc10a1, Slc23a1, Slc2a10, Slc6a2, Smurf2, Sppl2a, Svopl, Taf1, Taf1a, Tcea1, Timm17b, Tmem245, Tns4, Tprkb, Tspyl5, Ttc9c, Uvssa, Veph1, Vps72, Vsx1, Was, Xcr1, Ybx1, Zfp111, Zfp553, Zfp78, Zfp882, Zfp941, Zmym2, Zyg11b

<u>4. ENSMUSG00000076138</u>

mmu-miR-703

AAAACCUUCAGAAGGAAAGAA

Targets: 4933427D06Rik, Arhgap11a, Asxl2, Braf, Cep97, Cnnm3, Cnot4, Commd8, Epas1, Faah, Fam96a, Foxj3, Git2, Gpr3, Il1r1, Ilkap, Kif23, Larp4b, Mars2, Mau2, Mmab, Mnat1, Mxra7, Narf, Pcnp, Pctp, Pde8b, Pdxk, Phex, Pitpnm2, Prex2, Prr18, Prtg, Ptcd1, Rem1, Ret, Ric3, Rnf165, Rpp14, Rras2, Rsf1, Sept8, Slc12a2, Slc25a51, Slc30a7, Tcf7l2, Tctn3, Tlx2, Tmem170b, Ttc17, Ttc32, Usp29, Usp43, Vps26a, Xpnpep3, Zbed4, Zbtb14, Zfp106, Zfp11, Zfp53, Zfp84

<u>5. ENSMUSG00000098973</u>

no

<u>6. ENSMUSG00000097277</u>

no

<u>7. ENSMUSG00000104618</u>

mmu-miR-1839-5p

AAGGUAGAUAGAACAGGUCUUG

Targets: 2410012M07Rik, Akap8, Azi2, Ccdc157, Ecm2, Eif2s1, Fam211b, Fli1, Glt28d2, Il7, Mfsd4, Neu4, Ric8b, Slc11a2, Slc25a42, Tnfrsf26, Ttll1

<u>8. ENSMUSG00000104618</u>

mmu-miR-1839-3p

AGACCUACUUAUCUACCAACAGC

Targets: Grb14, Hist1h1d, Myo9a, Pvrl4, Reep3

<u>9. ENSMUSG00000098457</u>

no

10. ENSMUSG00000080666

mmu-miR-466i-3p

AUACACACACACAUACACACUA

Targets: 1700028K03Rik, 2900026A02Rik, 4933427D06Rik, 9930021J03Rik,A430078G23Rik, A630001G21Rik, AK010878, Aak1, Abca6, Acot11, Acvr1b, Adamts9, Adcyap1r1, Adm, Adrbk2, Akap10, Akap5, Alkbh1, Alkbh5, Ankle1, Anln, Aplnr, Apol8, Appl1, Arf3, Arhgap12, Arhgap32, Arl14epl, Arl4c, Arl5b, Arl8a, Armc2, Atg10, Atxn1, Atxn7, Azi2, B3gnt2, B4galt6, BC030336, Bbc3, Bcl2l11, Bcl2l15, Bpifc, Bsn, Btnl9, C2cd4c, Cadm3, Ccdc121, Ccdc144b, Ccdc169, Ccpg1, Cdc14a, Cdc37, Cdk12, Cebpa, Cecr6, Cep170b, Cep76, Chek1, Chic1, Chrna1, Clec7a, Cnr1, Col24a1, Col8a1, Cops7b, Crim1, Cry2, Csnk1e, Csrnp3, Cst6, Ctif, Ctsl, Cxcl5, Cyb5r1, Cytip, D230025D16Rik, D630023F18Rik, Dcaf17, Dcun1d1, Ddit4l, Ddx55, Decr1, Dennd4a, Desi2, Dhx37, Diap2, Dnajb1, Dnajb4, Dnajb6, Dpm1, Dus3l, Dusp18, Dusp9, Dzank1, Dzip3, E2f1, Efnb2, Ehhadh, Eif2s1, Eif4e2, Epha4, Erc1, Ermp1, Ets1, Evi2b, Eya1, Fam101b, Fam107a, Fam160b2, Fam168b, Fam169b, Fam212b, Fam214b, Fam227a, Fam46a, Fam53c, Fam78b, Fat3, Fbxl14, Fbxl17, Fech, Fgf5, Fhod1, Fign, Fndc1, Fndc3a, Foxj3, Foxn3, Frat2, Fus, Gabpb2, Gcnt4, Gfod1, Gm13476, Gm1587, Gm5148, Gmeb1, Gnb4, Gnl3l, Gpcpd1, Gpd1l, Gpkow, Gpr20, Grb2, Grik3, Grin2b, Gtf2h2, Gxylt1, Haus2, Hecw1, Hif1an, Hif3a, Hist1h1d, Hivep3, Hk2, Hook3, Hrk, I830012O16Rik, Ids, Ifitm7, Igf1, Ikzf2, Il15ra, Il17ra, Il18r1, Ints6, Ints8, Irgq, Itpripl2, Jag2, Jhdm1d, Kcnc1, Kctd11, Klf8, Klhl13, Klra2, Larp1, Ldb3, Ldlrad3, Lhfpl4, Lin28b, Lmx1a, Lnp, Lonrf1, Loxl3, Lrp12, Luc7l2, Lzts3, Map3k7, Mast3, Mast4, Mavs, Mcam, Mdga1, Mef2a, Mfsd5, Mill1, Mkx, Mmp12, Mms22l, Mrap2, Mrps24, Ms4a4b, Ms4a4c, Msr1, Msx2, Mtfr2, Mtx3, Mylk, Nacc2, Nbeal1, Ndrg2, Nfatc2, Nkx1-2, Npy1r, Nrf1, Nrg3, Nrp2, Nufip2, Nup133, Oas1e, Oas3, Ociad2, Oscp1, Otog, Pak6, Pappa, Pard6b, Pbld2, Pcdh15, Pcgf2, Pck1, Pde4dip, Pfas, Pgbd1, Pgm2l1, Phactr2, Phtf2, Pias1, Piga, Pik3r1, Plag1, Plau, Plcl1, Plxna4, Pofut1, Ppp1r14c, Prdx1, Prkcd, Prps2, Prr5, Prrg3, Prrx1, Prss22, Pter, Ptp4a2, Ptprk, Rab3c, Rabgap1, Rad51d, Rad54l2, Rap2b, Rbbp5, Rdh1, Rgl1, Rgs20, Rnasel, Rnf152, Rnf157, Rock2, Rpp14, Rraga, Rtf1, Rufy2, Rwdd4a, Scg2, Scrt2, Sept11, Sept3, Serpinb8, Set, Sgms1, Sin3a, Six4, Skint7, Slc10a2, Slc11a2, Slc12a2, Slc16a10, Slc25a46, Slc25a51, Slc41a3, Slc6a17, Slc6a8, Slc7a11, Slfn5, Slx1b, Smarca4, Snhg11, Sort1, Sox7, Spag7, Spata13, Spta1, Srgap2, Srp72, Srxn1, Ss18l1, Ssr1, St6galnac1, Ston2, Strbp, Stx1b, Stxbp6, Synj2bp, Synpo2l, Syt12, Syt2, Sytl4, Tagap, Tagap1, Tas1r3, Tbc1d13, Tbc1d22b, Tbl1xr1, Tbx20, Tcf4, Tcte1, Tdrd1, Thsd4, Tll1, Tmbim1, Tmem178b, Tmem201, Tmem252, Tmem26, Tmprss13, Tnfrsf11a, Tns1, Tns3, Tpm3, Tpte, Tril, Trim12c, Trim44, Tspan31, Tyw1, Ubac1, Ubap2l, Ube2v2, Ubn2, Ugt2b35, Uhrf1bp1l, Unc5a, Unc5c, Usf2, Usp27x, Vegfa, Vps4b, Vps72, Wfdc12, Wfs1, Whsc1l1, Xiap, Xk, Zbtb10, Zbtb34, Zbtb39, Zbtb42, Zbtb7b, Zc3h12d, Zdhhc5, Zfhx3, Zfp128, Zfp169, Zfp354a, Zfp408, Zfp462, Zfp516, Zfp827, Zfp839, Zfp871, Zfp874b, Zfp882, Zfp951, Zfp955a, Zfp963, Zscan4c, Zscan4d, Zxdc

11. ENSMUSG00000080666

mmu-miR-466i-5p

UGUGUGUGUGUGUGUGUGUG

Targets: 1700019G17Rik, 4921509C19Rik, Abcc9, Acot2, Acsm2, Acss1, Adamts14, Adamts17, Adarb2, Ado, Adra1b, Adrbk2, Agtrap, Akap10, Akap2, Akap7, Ank2, Ap1ar, Apba1, Aplnr, Appl1, Aptx, Asah2, Asap3, Asb13, Ascl4, Asxl2, Atp2b3, Atxn1, B4galt6, BC053749, Barhl2, Baz2b, Bcl2, Bcl2l11, Bend3, Bnc2, C030039L03Rik, C5ar2, Cacna2d2, Calcoco1, Calcr, Camk1d, Cap2, Car10, Casc4, Cask, Casp8, Ccdc85a, Ccna2, Ccpg1, Ccrl1, Cd1d1, Cd274, Cd28, Cd2ap, Cd33, Cd4, Cdh12, Cdh20, Cdk13, Cdk7, Cds2, Ceacam1, Ceacam18, Cer1, Chrnb4, Chrnd, Chst2, Clec7a, Clvs1, Cml2, Cnih3, Cnksr2, Cntn3, Commd7, Cox15, Cradd, Creg2, Csf2ra, D430042O09Rik, D630003M21Rik, D630045J12Rik, Ddx6, Dgkg, Dhdh, Dhfr, Dmd, Dmrta1, Dnase1l3, Dnlz, Draxin, Drp2, Dzank1, E2f8, Ebf3, Ece1, Efcab14,

Egfl6, Ehd3, Ehhadh, Elfn1, Elovl6, Entpd1, Eogt, Epas1, Epha4, Epha7, Etv3, Evi2b, Fam107a, Fam169b, Fam198a, Fam213a, Fblim1, Fbrs, Fbxl17, Fech, Flnb, Flrt1, Foxk1, Frk, Frmd5, Fsd1l, Gabpb2, Gatc, Gcnt4, Gdpgp1, Gfod1, Gfpt1, Gfra2, Gm14137, Gm14326, Gm4841, Gm5615, Gna13, Gnal, Gnb4, Gpr123, Gramd1c, Grasp, Greb1, Gria3, Grid1, Grik3, Gtf2h2, Has2, Havcr2, Heatr2, Hif1an, Hoxc8, Hpca, Hspb7, Htt, Iars, Iffo2, Ifi44, Igf1r, Igf2, Iglon5, Igsf11, Il18r1, Il8, Ildr2, Insig2, Insr, Iqgap2, Itga11, Itga9, Jarid2, Kank2, Kcnc1, Kcnip3, Kcnj1, Kcnj16, Kif1a, Klhl13, Klhl23, Krt222, L1cam, LOC100048884, Lbp, Lcp2, Lhx6, Lifr, Lin7a, Lnp, Lpp, Lrrc32, Lrrc61, Lrrn4cl, Lrtm2, Ltf, Ly6g6c, Ly96, Maf, Mafb, Magee2, Man1c1, Man2a2, Map3k7, Mapk11, Mapkbp1, Mavs, Mbnl3, Mcidas, Mdm2, Mfap3l, Micall1, Mlec, Mocs1, Mon1b, Mrgpre, Ms4a5, Mtmr12, Muc13, Mylk4, Nab1, Nab2, Napb, Nat8l, Nbeal1, Ncam1, Ncan, Nedd4l, Neu1, Neurod2, Nfat5, Nfatc1, Ngfr, Nid1, Nkap, Nkx2-9, Nmnat2, Npr3, Nqo2, Nr4a2, Nrbp2, Nsun3, Nwd1, Nxpe3, Oacyl, Onecut2, Opcml, Oprm1, Oxsm, Oxtr, P4ha3, Pacsin2, Palm2, Pappa2, Pcdh10, Pcdh17, Pclo, Pdcd4, Pdxk, Pgm5, Phactr3, Pik3r5, Pirt, Plcb1, Pld5, Podn, Pogk, Ppp1r16b, Ppp1r1c, Prdm12, Prdm8, Prex2, Prkcd, Prkci, Prrc2b, Prss42, Psd2, Psd3, Pstpip2, Ptgdr, Ptprb, Ptpre, Pura, Rab11fip1, Rab3c, Rab6b, Rab9b, Rabgap1, Rara, Rasa2, Rasal2, Rassf2, Reps2, Rfx3, Rgs9bp, Rhobtb1, Rhou, Rnasel, Rorb, Rpusd2, Rsph4a, Runx1, Runx1t1, Runx3, Saa4, Samd7, Scd3, Scn2a1, Sema5a, Sema5b, Sema6a, Sfrp1, Sh2d2a, Shisa6, Shroom3, Sike1, Sim1, Six4, Ski, Slc17a5, Slc1a2, Slc22a8, Slc24a2, Slc25a12, Slc25a21, Slc30a10, Slc31a2, Slc39a14, Slc4a4, Slc5a8, Slc6a17, Slc6a6, Slc7a1, Slc8a1, Slc8a3, Slmo1, Smarca2, Smo, Smpd4, Snai2, Snap23, Snap25, Snx12, Sox1, Sp9, Srgap3, Ssr1, St18, St8sia1, Steap2, Stk10, Stxbp4, Stxbp5l, Supt7l, Suv420h1, Syn3, Synj2bp, Syt15, Tacr1, Tacr2, Tbc1d30, Tbx15, Tbx22, Terf2ip, Tet2, Tfap2b, Tgfb2, Thsd4, Tiprl, Tmco1, Tmem132b, Tmem151b, Tmem236, Tmem245, Tmem26, Tmem47, Tmod2, Tnfrsf13c, Tns4, Trim65, Trp53i11, Trpc7, Trpm3, Tspan18, Tyw3, Ubtf, Uhrf1bp1l, Unc13b, Unc5d, Unc93a, Uncx, Urb2, Vdr, Vprbp, Vps33b, Vps37a, Vsnl1, Wars2, Wdr46, Wrn, Xk, Xpr1, Xrcc3, Zeb2, Zfand2a, Zfhx3, Zfp169, Zfp248, Zfp329, Zfp39, Zfp449, Zfp46, Zfp488, Zfp641, Zfp68, Zfp691, Zfp74, Zfp92, Zfp931, Znrf3, Zscan29

12. ENSMUSG00000086782

no

13. ENSMUSG00000065451

mmu-miR-101a-3p

UACAGUACUGUGAUAACUGAA

Targets: 4930539E08Rik, Akt1, Atxn1, B3gnt5, Bend4, Bsn, COX2, Car10, Cdc42ep3, Cebpa, Chml, Dnajb4, Dusp1, Gse1, Hdgfrp3, Hif1a, Icos, Lrig2, Map7d1, Mapre1, Msi2, Ptgs2, Ptp4a3, Rcor3, Wars2, Zfp329

14. ENSMUSG00000088354

mmu-miR-3470a

UCACUUUGUAGACCAGGCUGG

Targets: 2010109I03Rik, A630033H20Rik, Abhd15, Acmsd, Acp2, Ap5m1, Arid4b, BC030476, Btaf1, Calm1, Camsap1, Cand2, Clmn, Crtc1, Csf3r, Cx3cr1, Denr, Dut, Elmod3, Epg5, Erich1, Fam134b,

Fam86, Fbxl3, Fcer2a, Fktn, Ftsj1, Gad2, Gla, Gprc5c, Ikbip, Il5ra, Invs, Itih5, Jakmip2, Kctd14, Ky, Lgr5, Limd2, Lrrc14b, Lrrc8e, Lsm11, March1, Med16, Mettl2, Mfap1a, Mob4, Mobp, Mrpl19, Myo1e, Nek7, Npy1r, Nwd1, Pax2, Pof1b, Prex2, Prickle2, Prss43, Ptpn7, Rangap1, Raph1, Rhag, Rint1, Samd4, Scd2, Slc17a8, Slc35g1, Slc7a2, Slx1b, Smyd4, Snx30, Srgn, Tmed5, Tmem170b, Tmem178b, Tmem50a, Tmppe, Trim39, Ttbk2, Ung, Wbp1l, Wdr59, Zfp397, Zfp459, Zfp617, Zfp959, Zfyve27

15. ENSMUSG00000088354

mmu-miR-3470b

UCACUCUGUAGACCAGGCUGG

Targets: 2010012O05Rik, 2510002D24Rik, 4930579G24Rik, 5730455P16Rik, A130010J15Rik, AY074887, Abcd4, Adam28, Afg3l1, Aida, Aoc3, Ap5z1, Arhgef9, Asxl2, Bckdk, Camk4, Car5a, Ccdc78, Cd300a, Cd300lf, Cd8b1, Chek2, Chrnb4, Ciapin1, Clmn, Cog7, Crtc3, Ctsa, Dbt, Dcp2, Ddx55, Denr, Dkc1, Dlgap5, Dnal1, Dtx3l, Eci1, Elf1, F10, Fbxo22, Fsd1l, Fxn, Gabpb2, Gga2, Gimap1, Glrx2, Gm4951, Gnal, Gng2, Hccs, Il11, Inip, Iws1, Kcna4, Krr1, Lhx9, Lmbrd1, Lrrc3, Lypla1, Madd, Mau2, Mettl2, Mob3c, Mpv17, Mtrr, N6amt1, Nav1, Nbl1, Ncl, Ncoa7, Nfatc2ip, Nol10, Opa3, Pik3c2a, Pla2g2d, Pla2g7, Polr3f, Prdm2, Psmb11, Psmd9, Rab27a, Rad51c, Rapgef4, Rnf168, Rp2h, Rpl23, Rpl7l1, Rprd1a, Samd8, Sap18, Sass6, Serpine1, Sfn, Sgpp2, Slc10a1, Slc23a1, Slc2a10, Slc6a2, Smurf2, Sppl2a, Svopl, Taf1, Taf1a, Tcea1, Timm17b, Tmem245, Tns4, Tprkb, Tspyl5, Ttc9c, Uvssa, Veph1, Vps72, Vsx1, Was, Xcr1, Ybx1, Zfp111, Zfp553, Zfp78, Zfp882, Zfp941, Zmym2, Zyg11b

16. ENSMUSG00000093119

no

17. ENSMUSG00000093133

no

18. ENSMUSG00000095278

mmu-miR-3470a

UCACUUUGUAGACCAGGCUGG

Targets:
2010109I03Rik, A630033H20Rik, Abhd15, Acmsd, Acp2, Ap5m1, Arid4b, BC030476, Btaf1, Calm1, Camsap1, Cand2, Clmn, Crtc1, Csf3r, Cx3cr1, Denr, Dut, Elmod3, Epg5, Erich1, Fam134b, Fam86, Fbxl3, Fcer2a, Fktn, Ftsj1, Gad2, Gla, Gprc5c, Ikbip, Il5ra, Invs, Itih5, Jakmip2, Kctd14, Ky, Lgr5, Limd2, Lrrc14b, Lrrc8e, Lsm11, March1, Med16, Mettl2, Mfap1a, Mob4, Mobp, Mrpl19, Myo1e, Nek7, Npy1r, Nwd1, Pax2, Pof1b, Prex2, Prickle2, Prss43, Ptpn7, Rangap1, Raph1, Rhag, Rint1, Samd4, Scd2, Slc17a8, Slc35g1, Slc7a2, Slx1b, Smyd4, Snx30, Srgn, Tmed5, Tmem170b, Tmem178b, Tmem50a, Tmppe, Trim39, Ttbk2, Ung, Wbp1l, Wdr59, Zfp397, Zfp459, Zfp617, Zfp959, Zfyve27

19. ENSMUSG00000095278

mmu-miR-3470b

UCACUCUGUAGACCAGGCUGG

Targets: 2010012O05Rik, 2510002D24Rik, 4930579G24Rik, 5730455P16Rik, A130010J15Rik, AY074887, Abcd4, Adam28, Afg3l1, Aida, Aoc3, Ap5z1, Arhgef9, Asxl2, Bckdk, Camk4, Car5a, Ccdc78, Cd300a, Cd300lf, Cd8b1, Chek2, Crnb4, Ciapin1, Clmn, Cog7, Crtc3, Ctsa, Dbt, Dcp2, Ddx55, Denr, Dkc1, Dlgap5, Dnal1, Dtx3l, Eci1, Elf1, F10, Fbxo22, Fsd1l, Fxn, Gabpb2, Gga2, Gimap1, Glrx2, Gm4951, Gnal, Gng2, Hccs, Il11, Inip, Iws1, Kcna4, Krr1, Lhx9, Lmbrd1, Lrrc3, Lypla1, Madd, Mau2, Mettl2, Mob3c, Mpv17, Mtrr, N6amt1, Nav1, Nbl1, Ncl, Ncoa7, Nfatc2ip, Nol10, Opa3, Pik3c2a, Pla2g2d, Pla2g7, Polr3f, Prdm2, Psmb11, Psmd9, Rab27a, Rad51c, Rapgef4, Rnf168, Rp2h, Rpl23, Rpl7l1, Rprd1a, Samd8, Sap18, Sass6, Serpine1, Sfn, Sgpp2, Slc10a1, Slc23a1, Slc2a10, Slc6a2, Smurf2, Sppl2a, Svopl, Taf1, Taf1a, Tcea1, Timm17b, Tmem245, Tns4, Tprkb, Tspyl5, Ttc9c, Uvssa, Veph1, Vps72, Vsx1, Was, Xcr1, Ybx1, Zfp111, Zfp553, Zfp78, Zfp882, Zfp941, Zmym2, Zyg11b

20. ENSMUSG00000089726

no

21. ENSMUSG00000076256

mmu-miR-19b-3p

UGUGCAAAUCCAUGCAAAACUGA

Targets: 1500009C09Rik, 1700019G17Rik, 2510039O18Rik, 4931406P16Rik, 5730455P16Rik, Abcg1, Abhd10, Abi2, Abl2, Acadm, Actn1, Acvr1b, Adam11, Adam23, Adcy1, Adcyap1r1, Add3, Aff4, Agap1, Agfg1, Aicda, Akap2, Akap7, Akap9, Aldh3a2, Amz2, Ankib1, Ankra2, Ankrd26, Ap3s2, Arap2, Arel1, Arf3, Arhgap21, Arid4b, Arl6ip1, Arpc3, Asxl3, Atf2, Atp10a, Atp2a2, Atp2b2, Atp6ap2, Atp6v1b2, Atp8a1, Atraid, Avl9, B3galnt1, B4galt6, Btbd7, C1galt1, Cadm2, Cadm3, Camk2n1, Camkk2, Camsap2, Caprin1, Cav1, Cblb, Cbln2, Ccser2, Cds1, Celf1, Cep120, Chrnd, Chst1, Clock, Cltc, Cml2, Cmtm6, Cnih, Cnksr2, Cnot1, Cnot6, Cnr1, Cox5b, Csmd1, Daam1, Dcbld2, Dennd5a, Diras2, Dock4, Dpp6, Dpysl2, Drd1a, Dsel, Efnb2, Eif4g2, Enc1, Enpp4, Enpp5, Entpd7, Eogt, Ercc6, Etnk1, Ezh1, Fam104a, Fam126a, Fam173b, Fam179b, Fam20b, Fam83d, Fat3, Fbxo8, Fem1b, Fgfr1op2, Fkbp1b, Fndc3b, Foxo3, Ftsjd2, Furin, Fzd6, Gadd45a, Gja3, Gmeb2, Gmpr, Gnai1, Gng12, Golt1b, Gpcpd1, Gpr155, Gramd1b, Grb10, Grin3a, Gtdc2, Gtf2i, Hecw2, Heg1, Hif1an, Hipk3, Hlf, Hmgcll1, Hpca, Hprt, Hs2st1, Igfbp3, Ilf3, Inhbb, Itga6, Jtb, Kcnab3, Kcnj10, Kdm2a, Kif21a, Kif21b, Klf7, Ldhb, Ldlr, Ldlrad4, Lemd3, Lhfpl2, Lman1, Lnpep, Lonrf1, Lrp11, Maml1, Map3k1, Map3k12, Mfsd7c, Mid2, Mink1, Msantd4, Mtmr12, Myh9, Mylip, N4bp1, Naa50, Nap1l3, Nbea, Ncald, Ndfip2, Neurod1, Nfia, Nfya, Nhsl1, Nipal3, Npnt, Npy2r, Nrarp, Nsg2, Nudt10, Oprl1, Pak3, Pank1, Papss2, Paqr8, Parg, Pde7b, Pea15a, Phf3, Phtf2, Pianp, Pigl, Pik3r3, Plagl2, Ppap2b, Ppp2r2a, Ppp2r5e, Pros1, Prrc2c, Prrg3, Prune2, Psme4, Pten, Rab11b, Rab21, Ralbp1, Ralgps1, Rapgefl1, Rasgef1a, Rasgrp1, Rassf8, Reps2, Ric3, Rin2, Rnf11, Rnf111, Rnf128, Rnf167, Rnf216, Rnf38, Rnf44, Robo1, Rph3a, Sall3, Scamp5, Sema5a, Sfrs18, Sgcb, Sh3d19, Sh3rf3, Shank1, Sik1, Skil, Slc17a7, Slc24a2, Slc24a3, Slc35b4, Slc35f1, Slc35f3, Slc36a1, Slc6a8, Slc7a5, Slc9a2, Slitrk5, Smarca2, Smoc2, Snap25, Snip1, Sntb2, Snx27, Snx30, Socs6, Soga1, Soga3, Sp1, Srebf2, Srrm2, Sstr4, St8sia3, Stam, Stard13, Stat1, Stim2, Stk35, Suv420h1, Syt1, Syt11, Tbc1d12, Tbc1d23, Tef, Tet2, Tgif1, Timp2, Tm4sf1, Tm9sf3, Tmem179, Tmem245, Tmem25, Tmem30a, Tmem57, Tmod2, Tnip1, Tnks, Tnpo1, Tnrc6b, Trappc10, Trib2, Tspan2, Tspyl2, Ttc3, Ttc39b, Ttyh3, Tub, Txnip, Ubap2l, Ube3b, Ubl3, Ubr4, Ubxn4, Usp33, Vcpip1, Whsc1l1, Wnk1, Wnt1, Xxylt1, Ywhab, Zcchc2, Zeb2, Zfp120, Zfp275, Zfp41, Zfp521, Zfp597, Zfp800, Zfpm2, Zhx3, Zkscan1, Zmym2, Znfx1

22. ENSMUSG00000065403

mmu-miR-18a-5p

UAAGGUGCAUCUAGUGCAGAUAG

Targets: Abce1, Hsf2, Kif1a, Lrrc58, Map3k1, Pten, Rab11fip1, Runx1, Smad2

23. ENSMUSG00000076062

mmu-miR-92a-3p

UAUUGCACUUGUCCCGGCCUG

Targets: Btg2, Cd69, Chm, Cpeb1, Fam136a, Gm14420, Gm5148, Hipk3, Il5ra, Map2k4, Mylip, Ncam2, Rhbdl3, Shox2, Stk10, Tagap, Tagap1, Tbx3, Trp63, Uba1y, Zfp300, Zfpm2, Zpbp

24. ENSMUSG00000065508

mmu-miR-17-5p

CAAAGUGCUUACAGUGCAGGUAG

Targets: 1600012H06Rik, 1700052N19Rik, 2510009E07Rik, 2810055G20Rik, 4930506M07Rik, 4931406C07Rik, 6030458C11Rik, A330021E22Rik, Abca1, Acap2, Acvr1b, Adam10, Adam17, Adam19, Adcyap1r1, Aff4, Ago4, Aifm1, Ajuba, Akt3, Aldh6a1, Alkbh5, Amot, Ankhd1, Ankrd17, Ankrd29, Ankrd9, Anp32a, Ap3d1, Aplp2, App, Appl2, Arap2, Arhgef9, Aspscr1, Atf2, Atxn3, B3galt2, B4galnt1, Bcl2l11, Bmp2k, Bmp4, Brms1l, Brwd1, Btg2, C1qa, C2cd4c, Cacna2d1, Cadm2, Cald1, Camk2n1, Camta1, Capn2, Casc4, Cav1, Cbx2, Ccdc88a, Ccser2, Cd164, Cd28, Cdc42se2, Cdc7, Cdh2, Cds1, Celf2, Celsr2, Cend1, Chl1, Cnot6l, Col4a2, Cops2, Cpe, Cpeb3, Cpeb4 ,Crebrf, Cxcl12, Cyld, Cyth1, D15Ertd621e, D4Wsu53e, D630045J12Rik, Dcbld2, Derl2, Dgkd, Dhcr24, Dhx36, Dio2, Dip2a, Dmd, Dnm1l, Dnmt3a, Dpysl2, Dpysl5, Draxin, Dync1li2, Eef2k, Elovl6, Eml1, En2, Entpd7, Ep300, Epb4.1l5, Epha7, Eps15, Erbb4, Etnk1, Extl2, Extl3, Ezh1, F3, Fam117b, Fam120c, Fam134a, Fam134c, Fam227a, Fam49b, Fam63b, Fat2, Fbxo21, Fbxo9, Fcho2, Fchsd2, Fgf10, Fgfr1op2, Ficd, Flnb, Fn1, Foxp1, Frmpd4, Ftsjd2, Gabbr2, Gabra1, Gabrb3, Gas7, Gng4, Gpatch8, Gpm6b, Gpr63, Gramd1a, Grb10, Gtf2h2, Gxylt1, Hbp1, Hdac8, Heg1, Hid1, Hsd17b10, Id1, Ift88, Igfbp7, Igsf3, Il10rb, Ildr2, Insm1, Islr2, Itgb8, Itm2c, Kbtbd8, Kdelr2, Kdm2a, Kif21a, Kif5a, Kif5c, Klf10, Klf9, Klhl2, Klhl20, Klhl42, Kpnb1, Kras, Larp1, Lcorl, Limch1, Lmbrd1, Lnp, Lpp, Lrch1, Lrp11, Lrrc3, Lrrc55, Lrrn3, Lsamp, Luc7l3, M6pr, Macf1, Map1a, Map2, Map3k5, Map4, Map4k2, Map7d2, Mapk14, Mapre3, March6, March8, March9, Mcl1, Mcm7, Mctp2, Med17, Mef2c, Megf9, Mga, Mgll, Mlxip, Morf4l1, Mrc1, Msantd4, Msl1, Mycbp, Mylip, Myo10, N4bp2l2, Napb, Nbea, Ncam1, Necab1, Nedd4l, Nefh, Neurl1b, Neurod1, Nf1, Nfe2l2, Nfia, Nipa2, Npat, Nptx1, Nr1d2, Nrip1, Nsg2, Nt5dc3, Nudcd3, Nudt18, Ogfod1, Oprl1, Osr1, Otud4, Pabpc5, Pank1, Papolg, Pappa, Pbrm1, Pcdh10, Pcdhac1, Pcf11, Pdp2, Pfn2, Pgrmc2, Phlpp1, Pik3r4, Pim3, Plagl2, Plxna2, Polq, Polr3k, Ppap2b, Ppig, Ppp1r21, Ppp1r3b, Ppp2r2c, Ppp3r1, Ppp6c, Prune2, Pten, Ptp4a2, Ptpn11, Ptprg, Ptprj, Pum1, Pvr, Qser1, Rab11fip4, Rab12, Rab30, Rab33b, Rabgap1l, Ralgds, Ranbp2, Rap1gds1, Rapgefl1, Rasa1, Rasal1, Rasgef1a, Rassf4, Rb1, Rbl2, Rcan3, Rdx, Reep1, Rev3l, Rgma, Rnf103, Rnf213, Rnf220, Robo2, Rogdi, Rora, Rsf1, Rsrc2, Rundc3b, Ryr2, Sall3, Scara5, Scn1a, Scn2a1, Scn3b, Scn8a, Scp2, Sdccag3, Sema3c, Sepp1, Serinc1, Serpinb9, Sez6l,

Sh3bgrl, Sh3d19, Shh, Shox2, Ski, Skil, Slc10a7, Slc17a7, Slc1a4, Slc24a2, Slc35f3, Slc36a1, Slc44a5, Slc7a14, Slc7a2, Smim20, Smoc2, Sobp, Socs6, Sox8, Spag9, Spast, Sqstm1, Srcin1, St6galnac5, St8sia3, Stat3, Stxbp5, Syt1, Syt11, Taok1, Taok2, Tbc1d12, Tbc1d8b, Tbx3, Tceal1, Tceb3, Tenm4, Tex2, Tfe3, Tgfa, Timp1, Timp2, Tmcc1, Tmed8, Tmem230, Tmem64, Tmod2, Tmx4, Tnfrsf21, Tnks, Tnrc6b, Tomm34, Tor1aip2, Trim2, Trip12, Trp53inp1, Tspan9, Ttc14, Ttc9, Ubr3, Ubtf, Ubxn2a, Ugcg, Usp3, Vegfa, Wdr37, Wdr82, Wfs1, Whsc1l1, Wnk1, Xpc, Xrn1, Yipf6, Ythdf3, Yy1, Zbtb41, Zdhhc16, Zeb2, Zfand4, Zfhx3, Zfp217, Zfp317, Zfp367, Zfp597, Zfp62, Zfp652, Zfp 704, Zfp84, Zfpm2, Zhx3, Zic2, Zmat3, Znfx1

25. ENSMUSG00000065416

mmu-miR-19a-3p

UGUGCAAAUCUAUGCAAAACUGA

Targets: 1700019G17Rik, Acadm, Akap2, Atp10a, Atxn1, Chrnd, Cml2, Fam104a, Fam83d, Fgfr1op2, Gja3, Ilf3, Kdm2a, Map3k12, Nhsl1, Pten, Rnf11, Slc35f1, Snap25, Snx27, Tet2, Timp2, Tmem25, Tnf, Ttc39b, Ubap2l, Zfpm2

26. ENSMUSG00000065442

mmu-miR-20a-5p

UAAAGUGCUUAUAGUGCAGGUAG

Targets: Ago4, App, Bmp4, Cd28, Ep300, Fam227a, Fgf10, Fgfr1op2, Hbp1, Id1, Il10rb, Kdm2a, Larp1, Mapk14, Mctp2, Mef2c, Morf4l1, Nedd4l, Neurl1b, Osr1, Pcdh10, Pdp2, Ppp1r3b, Pten, Ptp4a2, Shox2, Sqstm1, St8sia3, Stat3, Tbx3, Timp2, Trp53inp1, ULK1, Ubxn2a, Vegfa, Zbtb41, Zbtb7a, Zfpm2

27. ENSMUSG00000096425

mmu-miR-3470a

UCACUUUGUAGACCAGGCUGG

Targets: 2010109I03Rik, A630033H20Rik, Abhd15, Acmsd, Acp2, Ap5m1, Arid4b, BC030476, Btaf1, Calm1, Camsap1, Cand2, Clmn, Crtc1, Csf3r, Cx3cr1, Denr, Dut, Elmod3, Epg5, Erich1, Fam134b, Fam86, Fbxl3, Fcer2a, Fktn, Ftsj1, Gad2, Gla, Gprc5c, Ikbip, Il5ra, Invs, Itih5, Jakmip2, Kctd14, Ky, Lgr5, Limd2, Lrrc14b, Lrrc8e, Lsm11, March1, Med16, Mettl2, Mfap1a, Mob4, Mobp, Mrpl19, Myo1e, Nek7, Npy1r, Nwd1, Pax2, Pof1b, Prex2, Prickle2, Prss43, Ptpn7, Rangap1, Raph1, Rhag, Rint1, Samd4, Scd2, Slc17a8, Slc35g1, Slc7a2, Slx1b, Smyd4, Snx30, Srgn, Tmed5, Tmem170b, Tmem178b, Tmem50a, Tmppe, Trim39, Ttbk2, Ung, Wbp1l, Wdr59, Zfp397, Zfp459, Zfp617, Zfp959, Zfyve27

28. ENSMUSG00000096425

mmu-miR-3470b

UCACUCUGUAGACCAGGCUGG

Targets: 2010012O05Rik, 2510002D24Rik, 4930579G24Rik, 5730455P16Rik, A130010J15Rik, AY074887, Abcd4, Adam28, Afg3l1, Aida, Aoc3, Ap5z1, Arhgef9, Asxl2, Bckdk, Camk4, Car5a, Ccdc78, Cd300a, Cd300lf, Cd8b1, Chek2, Chrnb4, Ciapin1, Clmn, Cog7, Crtc3, Ctsa, Dbt, Dcp2, Ddx55, Denr, Dkc1, Dlgap5, Dnal1, Dtx3l, Eci1, Elf1, F10, Fbxo22, Fsd1l, Fxn, Gabpb2, Gga2, Gimap1, Glrx2, Gm4951, Gnal, Gng2, Hccs, Il11, Inip, Iws1, Kcna4, Krr1, Lhx9, Lmbrd1, Lrrc3, Lypla1, Madd, Mau2, Mettl2, Mob3c, Mpv17, Mtrr, N6amt1, Nav1, Nbl1, Ncl, Ncoa7, Nfatc2ip, Nol10, Opa3, Pik3c2a, Pla2g2d, Pla2g7, Polr3f, Prdm2, Psmb11, Psmd9, Rab27a, Rad51c, Rapgef4, Rnf168, Rp2h, Rpl23, Rpl7l1, Rprd1a, Samd8, Sap18, Sass6, Serpine1, Sfn, Sgpp2, Slc10a1, Slc23a1, Slc2a10, Slc6a2, Smurf2, Sppl2a, Svopl, Taf1, Taf1a, Tcea1, Timm17b, Tmem245, Tns4, Tprkb, Tspyl5, Ttc9c, Uvssa, Veph1, Vps72, Vsx1, Was, Xcr1, Ybx1, Zfp111, Zfp553, Zfp78, Zfp882, Zfp941, Zmym2, Zyg11b

29. ENSMUSG00000097017

no

30. ENSMUSG00000096667

mmu-miR-1194

GAAUGAGUAACUGCUAGAUCCU

Targets: C3ar1, Car5b, Cd300a, Commd7, Dlc1, Efcab4a, Elf1, Fam154b, Gata5, Ggps1, Gtf2h2, H2-T24, Hs6st2, Itsn1, Jazf1, Lpar6, March5, Mzt1, Neu3, Nip7, Nrcam, Onecut3, Osbpl8, Pctp, Ppp1r16b, Rbak, Slc35d2, Spg21, Syne2, T2, Thoc6, Tmem151a, Tmem88b, Tnfsf10, Tox4, Unc5d, Usp29, Xpo7, Ybey

31. ENSMUSG00000097391

no

32. ENSMUSG00000065497

mmu-miR-410-3p

AAUAUAACACAGAUGGCCUGU

Targets: 1200011I18Rik, 4921524J17Rik, Acbd5, Akna, Anln, Arhgap11a, Arl15, Atp2b2, Cacnb2, Calm1, Casc4, Cbx3, Ccdc90b, Cd69, Ceacam1, Cops7b, Crebzf, Crp, Ctxn3, Cxxc5, Dimt1, Dlx3, Eif2s1, Elavl4, Ep300, Erc1, Etv3, Fam151b, Fam46a, Fbxl20, Fgf16, Folh1, Fsd1l, Fzd1, Fzd5, Gm8369, Gpc6, Grhl3, Has2, Hexim1, Hiat1, Hist1h1d, Hopx, Hoxa11, Id2, Igfbp3, Ikzf5, Ints6, Ism1, Klf12, L3mbtl4, Larp1, Lin54, Lrig2, Lrrc71, Lrrtm2, Lzts3, Map1a, Mettl20, Mex3b, Mgea5, Mllt3, Mpped2, Nadsyn1, Nck2, Nckap5l, Ndnf, Nf2, Nfib, Nln, Nol7, Ntrk3, Nupl2, Ocrl, Olr1, Osbp, Pcdh20, Pcdhb17, Pcsk5, Pfkl, Phf17, Pkib, Plau, Plaur, Plxnc1, Pm20d2, Pou4f2, Ppp4r2, Ppp6r1, Prrx1, Pten, Ranbp10, Rasal2, Rassf5, Rbms3, Rccd1, Reep5, Ret, Rnf166, Rnf6, Rragb, Rufy2, Runx1t1, Senp3, Shh, Slc12a8, Slc35a1, Slc7a11, Slc8a1, Slitrk2, Smurf2, Snx27, Sort1, Sox11, Sri, Strbp, Synpr, Syp, Tbx4,

Tcf21, Tcf7l2, Tead3, Tet2, Tex12, Tfam, Timp3, Tmem100, Tmem161b, Tmem56, Trak2, Trpc7, Trps1, Ttc17, Ttc39b, Ugt2b35, Usp6nl, Whsc1l1, Wnt11, Wnt3, Wwtr1, Zbed6, Zc3hav1l, Zer1, Zfand5, Zfp148, Zfp384, Zfp518b, Zfp644, Zmynd8, Znrf1, Zwint

33. ENSMUSG00000065426

mmu-miR-134-5p

UGUGACUGGUUGACCAGAGGGG

***Targets*:** Ccdc91, Chrdl1, Cmtm3, Creb1, Cysltr1, Dcx, Ehbp1l1, Evi2b, Fadd, Gnao1, Limk1, Lrtm2, Nanog, Nr5a2, Prkd3, Prrc2b, Pum2, Runx1t1, Shc4, Snai2, Sox2, Tmem167, Trpm3

34. ENSMUSG00000070128

mmu-miR-485-5p

AGAGGCUGGCCGUGAUGAAUUC

Targets: Cd93, Foxk2, Gns, Pax2, Pigm

35. ENSMUSG00000065438

mmu-miR-377-3p

AUCACACAAAGGCAACUUUUGU

Targets: 2810407C02Rik, Acot11, Acvr1b, Adam19, Adamts9, Adra2b, Agpat4, Anln, Arf3, Arhgap23, Arhgef15, Arhgef9, Arid4a, Arnt, Arxes2, Atf2, Atg16l1, Atl2, Atm, Atxn7, BC068281, Bbc3, Bsn, Cadm2, Cadm3, Capza1, Cbln3, Ccdc138, Ccrl1, Cd1d1, Cd3d, Cisd2, Clasp1, Clec4a2, Clic5, Coa5, Cog7, Creb5, Cry2, Cstf3, D16Ertd472e, Dcp2, Dzank1, E2f1, Ehf, Ehmt1, Elovl7, Ephb3, Ephx3, F11r, Fam114a1, Fam160b2, Fam212b, Fam53c, Fbxl17, Frmd3, Fubp1, Gad2, Gmeb1, Gpd1l, Gxylt1, Gxylt2, Hexim1, Hnrnpd, Hook3, I830012O16Rik, Ifit3, Igdcc4, Igfbp3, Iglon5, Ikzf2, Ino80d, Insig2, Ints8, Iqsec3, Itgam, Itsn1, Jazf1, Kcna4, Krtap4-9, Lcorl, Lman2, Lonrf1, Lrp8, Mbtps2, Mmab, Mob3c, Myrip, Nceh1, Net1, Nfatc4, Ociad2, Pabpc4l, Pacs2, Pak4, Pappa, Pax3, Pck1, Pdpk1, Pfkfb2, Pgbd1, Phactr2, Pi15, Plcl1, Plekha2, Plekhg1, Ppp1r2, Ppp1r26, Prr3, Prr5, Prrg3, Prrg4, Pttg1, Rapgef3, Rasal2, Rcan2, Rictor, Rmi2, Rnf152, Rnf219, Rnf41, Rsf1, Scamp1, Sele, Sema4f, Sema6d, Sept11, Sfn, Shroom1, Sin3a, Slc1a2, Slc35a1, Slc36a1, Slc43a2, Slc7a11, Smarca4, Spock2, Spp1, Spryd3, Tbl1xr1, Tcf15, Tmem170b, Tmem201, Tmem65, Tmem8b, Tmlhe, Tnfrsf1b, Tnfsf10, Trak1, Trp53i11, Tulp3, Ubn2, Ubtf, Ulk3, Vdr, Wdr59, Xpnpep3, Zbtb7a, Zdhhc24, Zdhhc7, Zfp467, Zfp532, Zfp663, Zfp689, Zfp827, Zfp839

36. ENSMUSG00000097501

no

37. ENSMUSG00000099245

no

38. ENSMUSG00000097961

no

39. ENSMUSG00000084559

mmu-miR-1906

UGCAGCAGCCUGAGGCAGGGCU

Targets: 4930444A02Rik, Angel1, Armcx6, Cdc14b, Cox15, Ddx19b, Dhdh, Fam168b, Fbxo21, Fgd4, Irgq, Isoc1, Itgav, Itsn1, Klc1, Mapkap1, Mbtps2, Mtf1, Opa1, Otop1, Oxsm, Pacsin2, Sorcs2, Spen, Spsb4, Tacc1, Tln2

40. ENSMUSG00000084535

mmu-miR-1906

UGCAGCAGCCUGAGGCAGGGCU

Targets: 4930444A02Rik, Angel1, Armcx6, Cdc14b, Cox15, Ddx19b, Dhdh, Fam168b, Fbxo21, Fgd4, Irgq, Isoc1, Itgav, Itsn1, Klc1, Mapkap1, Mbtps2, Mtf1, Opa1, Otop1, Oxsm, Pacsin2, Sorcs2, Spen, Spsb4, Tacc1, Tln2

41. ENSMUSG00000104193

mmu-miR-1906

UGCAGCAGCCUGAGGCAGGGCU

Targets: 4930444A02Rik, Angel1, Armcx6, Cdc14b, Cox15, Ddx19b, Dhdh, Fam168b, Fbxo21, Fgd4, Irgq, Isoc1, Itgav, Itsn1, Klc1, Mapkap1, Mbtps2, Mtf1, Opa1, Otop1, Oxsm, Pacsin2, Sorcs2, Spen, Spsb4, Tacc1, Tln2

42. ENSMUSG00000098343

no

43. ENSMUSG00000098638

mmu-miR-3470a

UCACUUUGUAGACCAGGCUGG

Targets:
2010109I03Rik, A630033H20Rik, Abhd15, Acmsd, Acp2, Ap5m1, Arid4b, BC030476, Btaf1, Calm1, Camsap1, Cand2, Clmn, Crtc1, Csf3r, Cx3cr1, Denr, Dut, Elmod3, Epg5, Erich1, Fam134b, Fam86, Fbxl3, Fcer2a, Fktn, Ftsj1, Gad2, Gla, Gprc5c, Ikbip, Il5ra, Invs, Itih5, Jakmip2, Kctd14, Ky, Lgr5, Limd2, Lrrc14b, Lrrc8e, Lsm11, March1, Med16, Mettl2, Mfap1a, Mob4, Mobp, Mrpl19, Myo1e, Nek7, Npy1r, Nwd1, Pax2, Pof1b, Prex2, Prickle2, Prss43, Ptpn7, Rangap1, Raph1, Rhag, Rint1, Samd4, Scd2, Slc17a8, Slc35g1, Slc7a2, Slx1b, Smyd4, Snx30, Srgn, Tmed5, Tmem170b, Tmem178b, Tmem50a, Tmppe, Trim39, Ttbk2, Ung, Wbp1l, Wdr59, Zfp397, Zfp459, Zfp617, Zfp959, Zfyve27

44. ENSMUSG00000098638

mmu-miR-3470b

UCACUCUGUAGACCAGGCUGG

Targets:
2010012O05Rik, 2510002D24Rik, 4930579G24Rik, 5730455P16Rik, A130010J15Rik, AY074887, Abcd4, Adam28, Afg3l1, Aida, Aoc3, Ap5z1, Arhgef9, Asxl2, Bckdk, Camk4, Car5a, Ccdc78, Cd300a, Cd300lf, Cd8b1, Chek2, Chrnb4, Ciapin1, Clmn, Cog7, Crtc3, Ctsa, Dbt, Dcp2, Ddx55, Denr, Dkc1, Dlgap5, Dnal1, Dtx3l, Eci1, Elf1, F10, Fbxo22, Fsd1l, Fxn, Gabpb2, Gga2, Gimap1, Glrx2, Gm4951, Gnal, Gng2, Hccs, Il11, Inip, Iws1, Kcna4, Krr1, Lhx9, Lmbrd1, Lrrc3, Lypla1, Madd, Mau2, Mettl2, Mob3c, Mpv17, Mtrr, N6amt1, Nav1, Nbl1, Ncl, Ncoa7, Nfatc2ip, Nol10, Opa3, Pik3c2a, Pla2g2d, Pla2g7, Polr3f, Prdm2, Psmb11, Psmd9, Rab27a, Rad51c, Rapgef4, Rnf168, Rp2h, Rpl23, Rpl7l1, Rprd1a, Samd8, Sap18, Sass6, Serpine1, Sfn, Sgpp2, Slc10a1, Slc23a1, Slc2a10, Slc6a2, Smurf2, Sppl2a, Svopl, Taf1, Taf1a, Tcea1, Timm17b, Tmem245, Tns4, Tprkb, Tspyl5, Ttc9c, Uvssa, Veph1, Vps72, Vsx1, Was, Xcr1, Ybx1, Zfp111, Zfp553, Zfp78, Zfp882, Zfp941, Zmym2, Zyg11b

45. ENSMUSG00000021268

no

46. ENSMUSG00000076451

mmu-miR-770-5p

AGCACCACGUGUCUGGGCCACG

Targets: BC027231, Chm, Cisd2, Gm14420, Lsm6, Pigt, Tmem178b, Tmlhe, Ttc38

47. ENSMUSG00000076451

mmu-miR-770-3p

CGUGGGCCUGACGUGGAGCUGG

Targets: 2510002D24Rik, Cd8b1, Pctp, Syt7, Tmem176b, Zfp941

48. ENSMUSG00000076128

mmu-miR-686

AUUGCUUCCCAGACGGUGAAGA

Targets:
1810030O07Rik, 5730455P16Rik, Ammecr1l, Amy2a2, Amy2a3, Amy2a4, Apbb2, Arhgef2, Atp2b1, B3galt1, Ceacam20, Cln8, Ctdspl2, Cyth1, Gabrb2, Intu, Kcnc3, Lig4, Lmna, Map3k1, Mbnl1, Mfap1a, Nfatc4, Nol11, Prf1, Raph1, Rbbp4, Rdh10, Rsph4a, Rxfp3, Slc22a15, Slc7a14, Spag7, Tbx2, Tenm2, Terf2ip, Tfcp2l1, Tmem234, Ubxn2b, Utp23, Zfp641

49. ENSMUSG00000097431

no

50. ENSMUSG00000094702= ENSMUSG00000093302= ENSMUSG00000092840

mmu-miR-3471

UGAGAUCCAACUGUAAGGCAUU

Targets: 2310030G06Rik, 9930104L06Rik, Aak1, Adam19, Akr1d1, Anapc16, Ankrd28, B4galt6, Casp8, Cd300a, Cd8b1, Chrnb4, Cnih4, Commd7, Cpm, Cxcr7, Dbt, Dennd4c, Ggps1, Glrx2, Glyctk, Gmip, Gnal, H2-T24, Heatr5a, Idi2, Ifngr2, Il1rl1, Ints6, Iws1, Limd1, Llph, March5, Neu3, Pigh, Ppp1r16b, Psmb11, Ptcd1, Ran, Rapgef4, Rnf150, Rprd1a, Rragd, S1pr3, Slc12a8, Slc35d2, Tcaim, Tmem151a, Tmem69, Tmem88b, Tnfsf10, Trp53rk, Ubxn2a, Urb2, Uvssa, Whsc1, Ybey, Zbtb9, Zfp329

miRNA with more than 5 targets were used for further analyses

C: Changes of miRNA expression patterns over time after normal and extended hepatectomy.

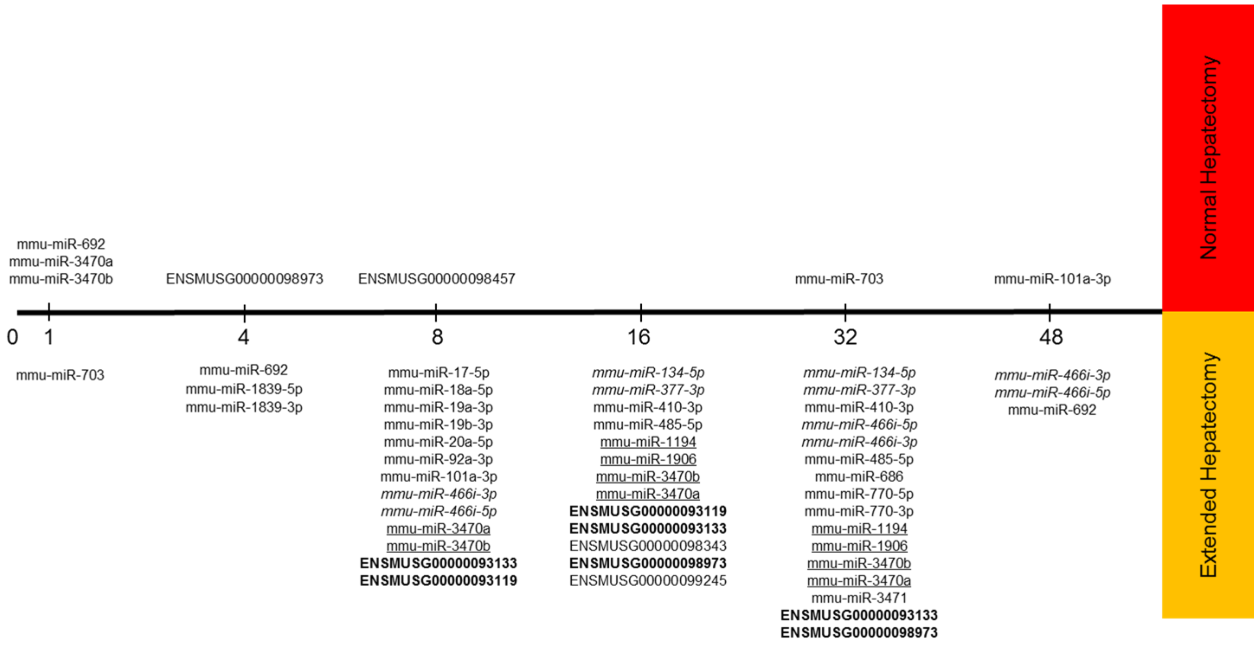

miRNA expression patterns over time after normal and extended hepatectomy.

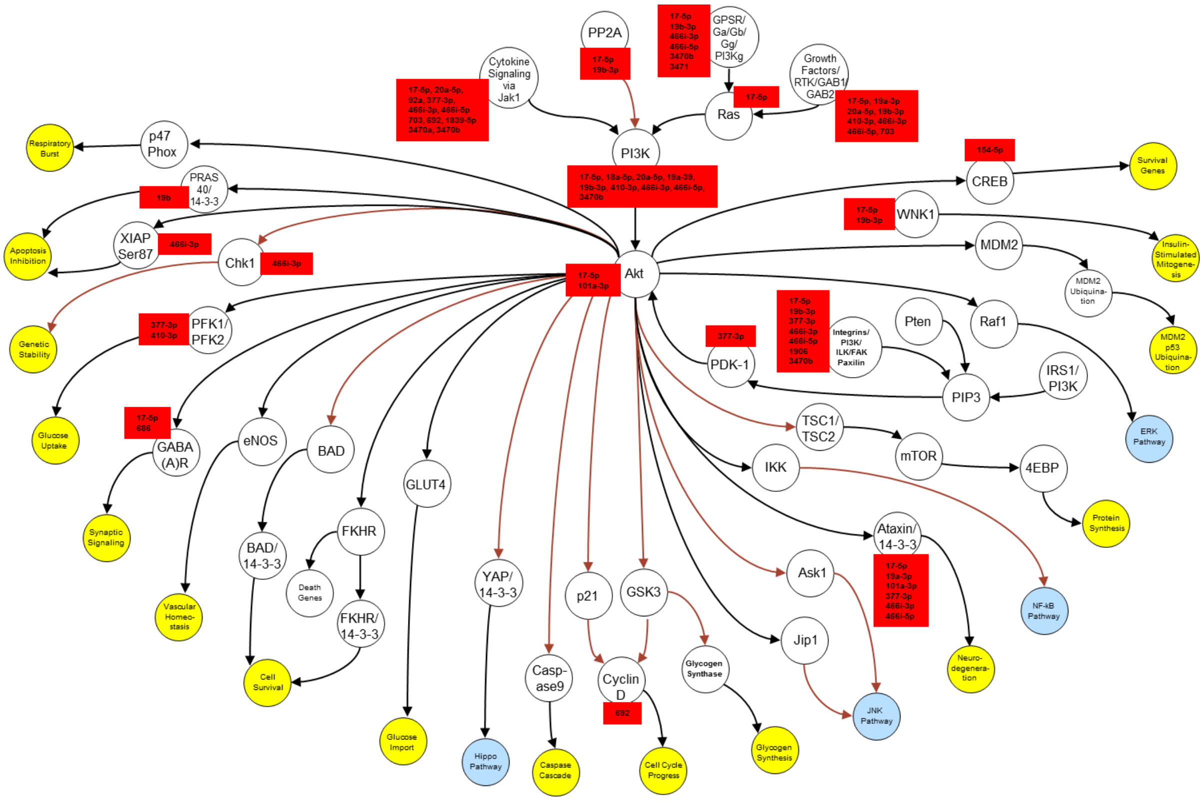

Borger et al
Supplemental Data

D. miRNA-based significantly differentially regulated ISPs

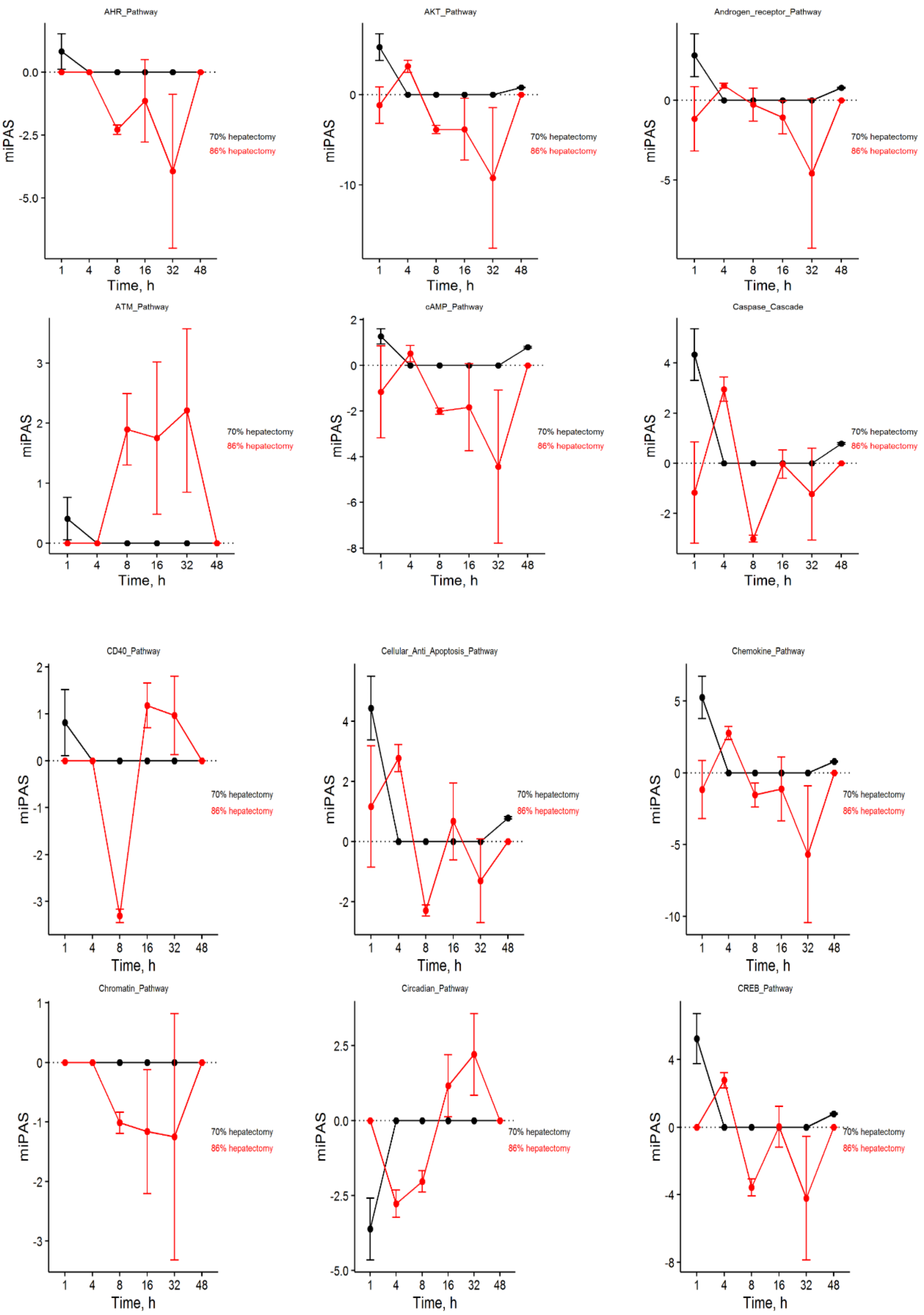

Borger et al
Supplemental Data

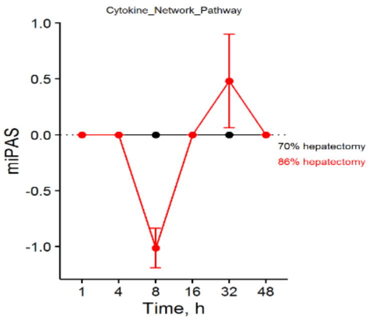

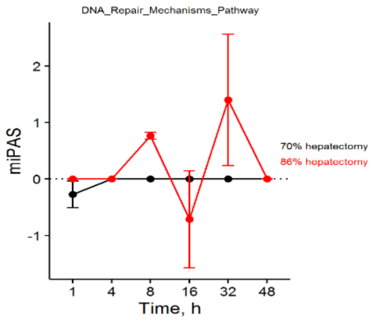

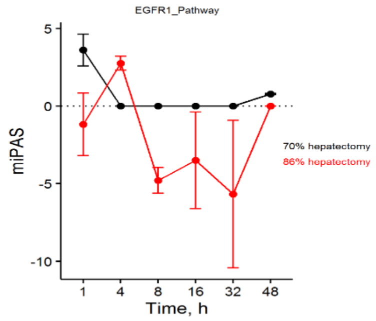

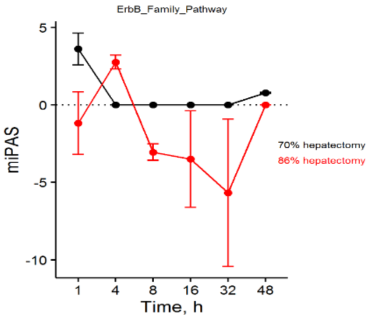

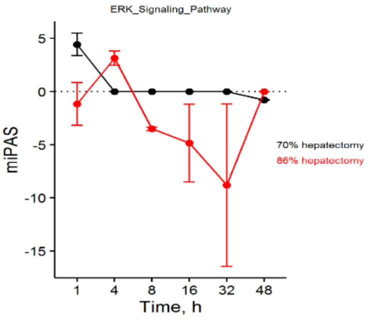

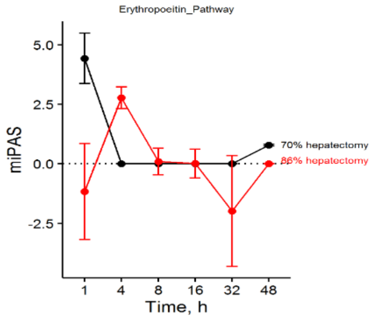

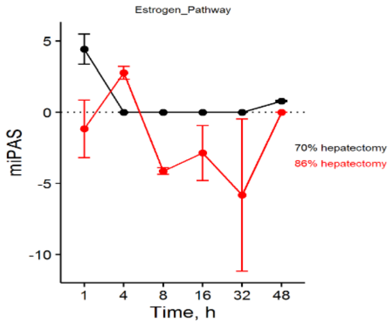

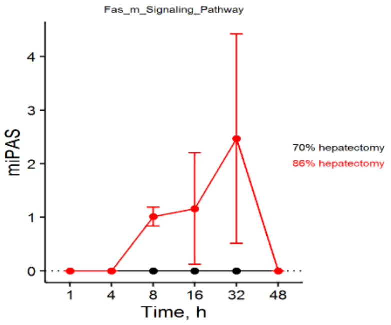

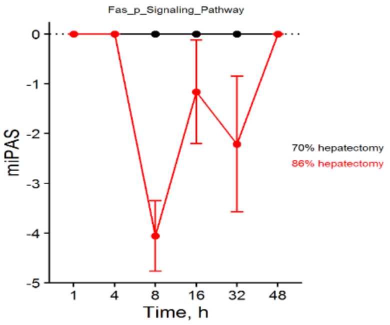

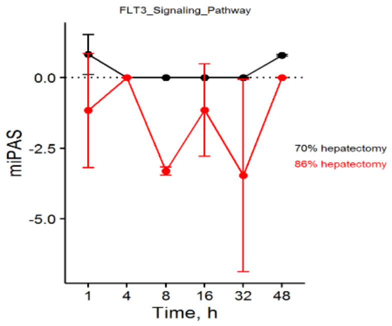

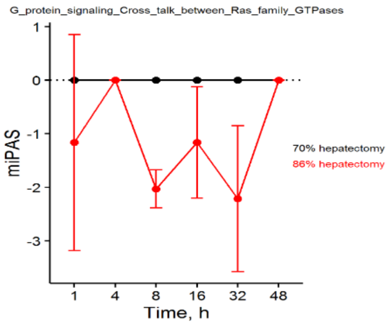

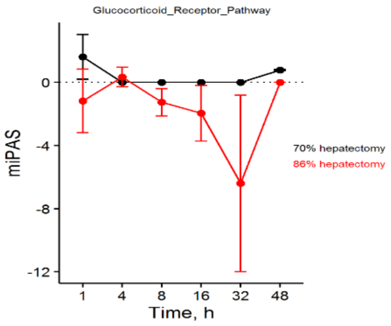

Borger et al
Supplemental Data

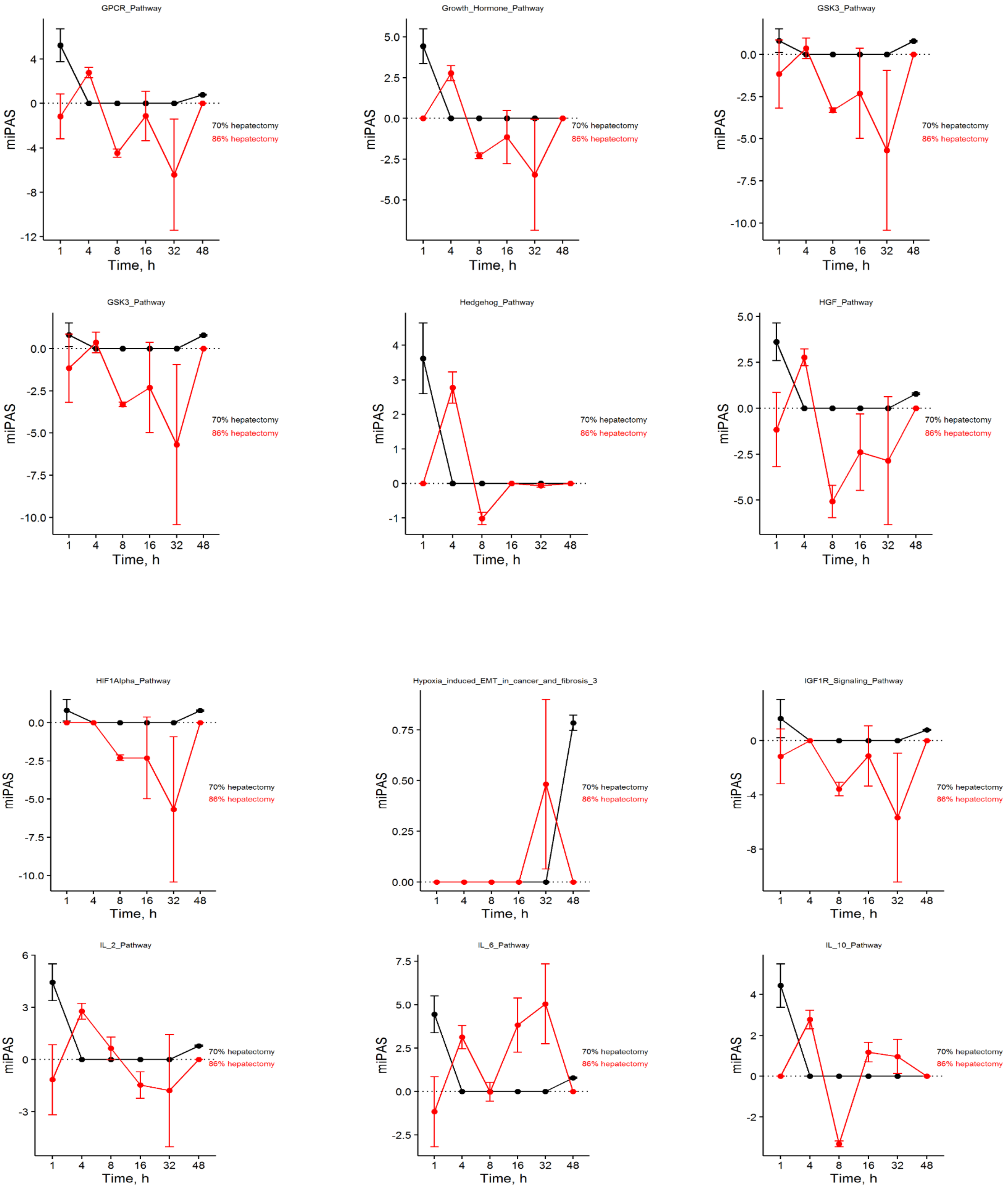

Borger et al
Supplemental Data

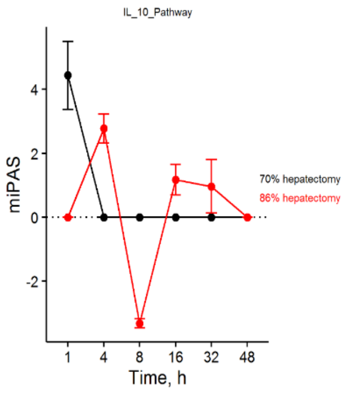

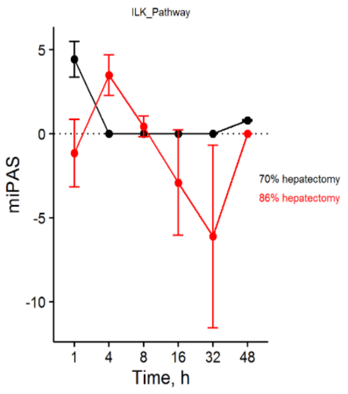

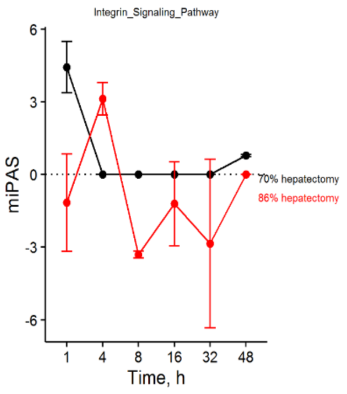

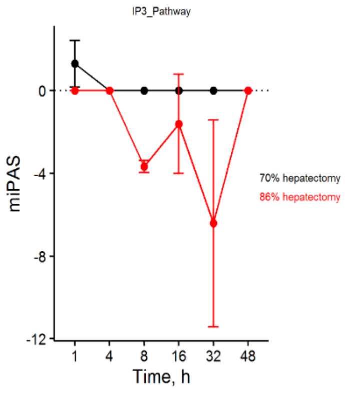

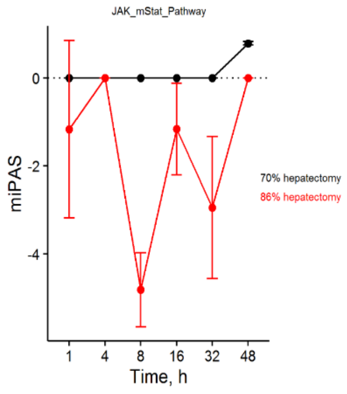

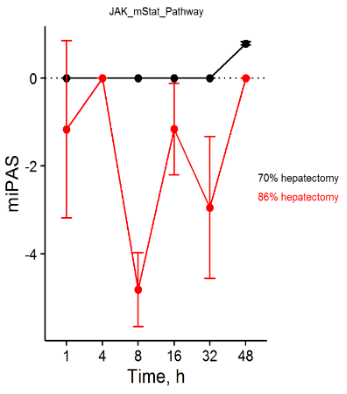

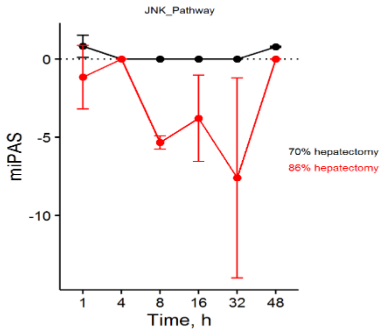

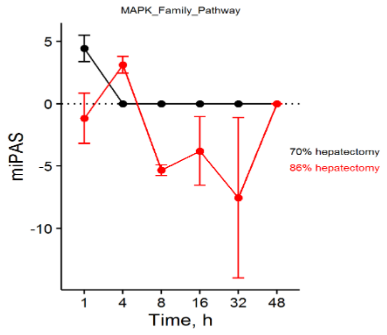

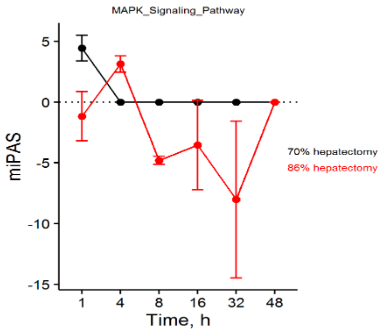

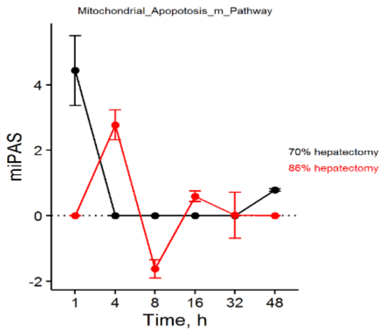

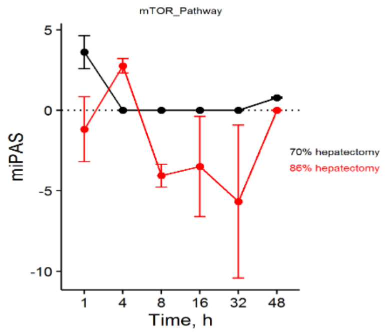

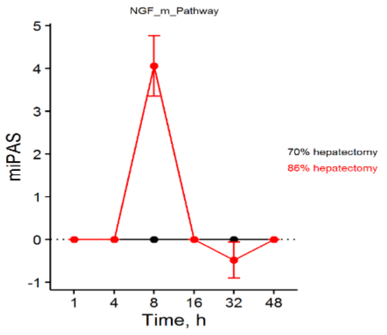

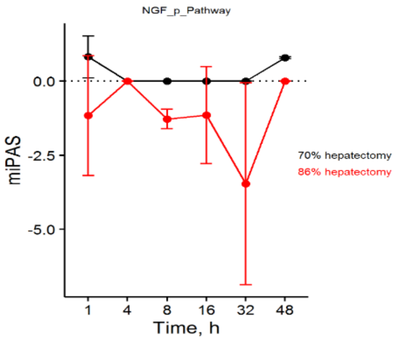
NGF_p_Pathway
miPAS
Time, h
70% hepatectomy
86% hepatectomy

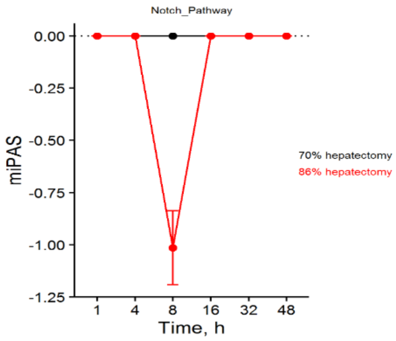
Notch_Pathway
miPAS
Time, h
70% hepatectomy
86% hepatectomy

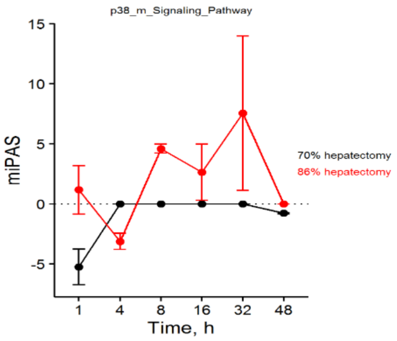
p38_m_Signaling_Pathway
miPAS
Time, h
70% hepatectomy
86% hepatectomy

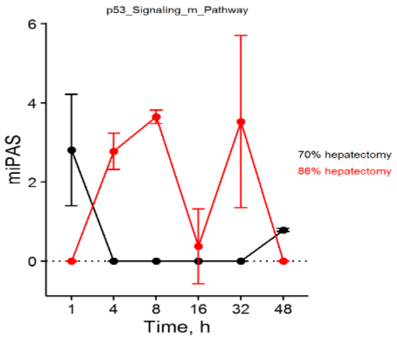
p53_Signaling_m_Pathway
miPAS
Time, h
70% hepatectomy
86% hepatectomy

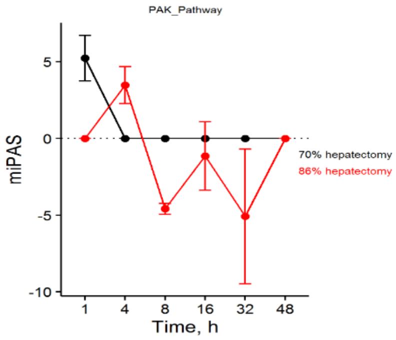
PAK_Pathway
miPAS
Time, h
70% hepatectomy
86% hepatectomy

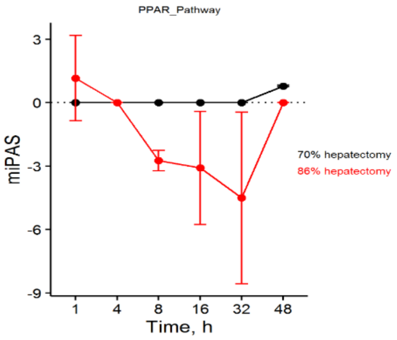
PPAR_Pathway
miPAS
Time, h
70% hepatectomy
86% hepatectomy

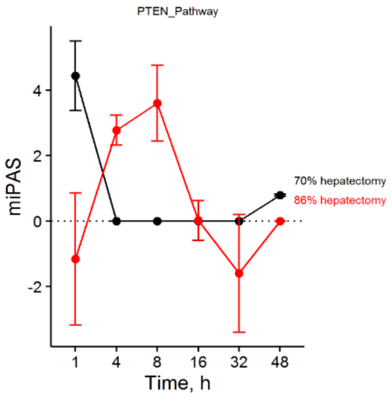
PTEN_Pathway
miPAS
Time, h
70% hepatectomy
86% hepatectomy

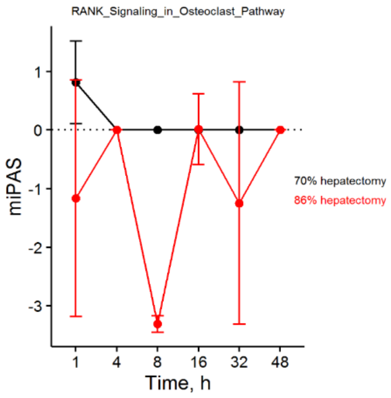
RANK_Signaling_in_Osteoclast_Pathway
miPAS
Time, h
70% hepatectomy
86% hepatectomy

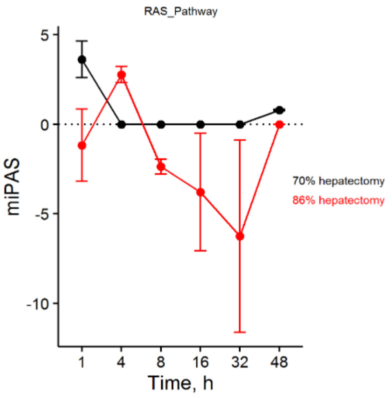
RAS_Pathway
miPAS
Time, h
70% hepatectomy
86% hepatectomy

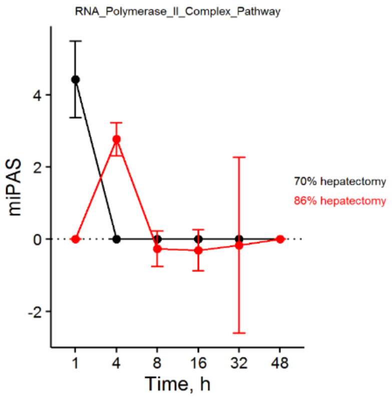
RNA_Polymerase_II_Complex_Pathway
miPAS
Time, h
70% hepatectomy
86% hepatectomy

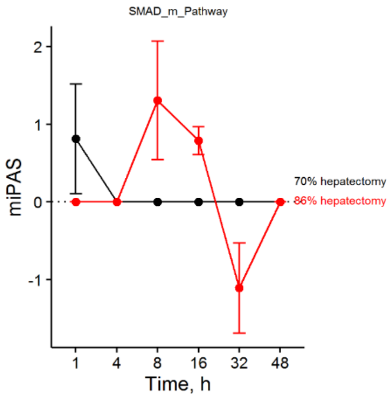
SMAD_m_Pathway
miPAS
Time, h
70% hepatectomy
86% hepatectomy

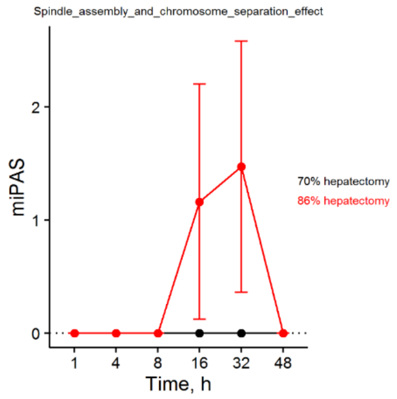
Spindle_assembly_and_chromosome_separation_effect
miPAS
Time, h
70% hepatectomy
86% hepatectomy

Borger et al
Supplemental Data

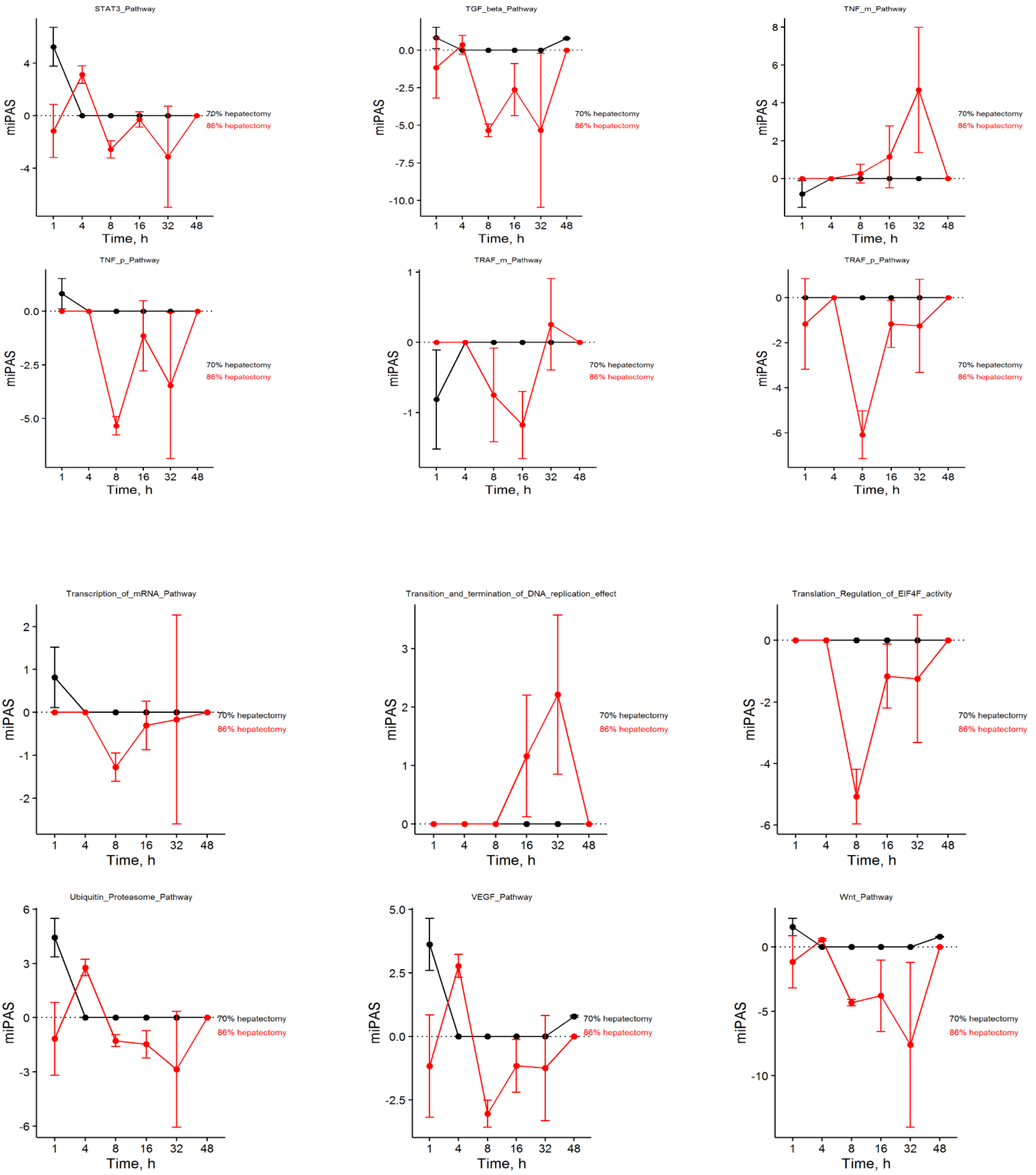

Supplement: Supplementary file 1 [file cells-09-01149-s001.pdf]
